# Efficacy and Acceptance of Cognitive Behavioral Therapy in Adults with Chronic Fatigue Syndrome: A Meta-analysis

**DOI:** 10.1007/s12529-023-10254-2

**Published:** 2024-01-16

**Authors:** Frederic Maas genannt Bermpohl, Ann-Cathrin Kucharczyk-Bodenburg, Alexandra Martin

**Affiliations:** Department of Clinical Psychology and Psychotherapy, University of Wuppertal, Gaußstraße 20, 42119 Wuppertal, Germany

**Keywords:** Chronic fatigue syndrome, Myalgic encephalomyelitis, Treatment evaluation, Adherence, Drop-out

## Abstract

**Background:**

The systematic aggregation of research on cognitive behavioral therapy (CBT) in chronic fatigue syndrome (CFS) needs an update. Although meta-analyses evaluating interventions typically focus on symptom reduction, they should also consider indicators of treatment acceptability, e.g., drop-out rates.

**Methods:**

Randomized controlled trials (RCTs) investigating CBT in adults with CFS compared to inactive and non-specific control groups were included. First, efficacy was examined, considering fatigue, depression, anxiety, and perceived health. Secondly, drop-out rates through different trial stages were analyzed: Non-completion of all mandatory sessions, drop-out (primary study definition), treatment refusal (non-starters), and average of sessions completed.

**Result:**

We included 15 RCTs with 2015 participants. CBT was more effective than controls in fatigue (*g* = -0.52, 95%CI -0.69 to -0.35), perceived health, depression, and anxiety at post-treatment. At long-term follow-up the effects were maintained for fatigue and anxiety. Rates of non-completion (22%, 95%CI 3–71), drop-out (15%, 95%CI 9–25), and treatment refusal (7%, 95%CI 3–15) were relatively low, with a high average proportion of sessions completed. Total time of therapy moderated the effect on fatigue, while the number of sessions moderated the effect on perceived health. Fatigue severity influenced adherence.

**Conclusion:**

The results indicate that CBT for CFS is effective in reducing fatigue, fatigue related impairment, and severity of depression and anxiety. Conclusions on efficacy at follow-ups are still limited. However, adherence is high in CBT. The results may help to inform clinical practice. Future research should focus on examining the maintenance of effects, while also emphasizing the importance of treatment acceptance.

**Supplementary Information:**

The online version contains supplementary material available at 10.1007/s12529-023-10254-2.

## Introduction

Chronic fatigue syndrome (CFS) is a debilitating disorder characterized by medically evaluated, unexplained, persistent or recurrent persistent fatigue that is not the result of current stress, not relieved by rest, results in significant activity limitations, and for which there is no clear organic explanation [1]. However, there is a broad array of possible diagnostic criteria that can be used for CFS. Hence, CFS according to the presented definition needs to be differentiated from newer classification approaches for myalgic encephalomyelitis/CFS [2]. While the etiology of CFS remains unclear, evidence suggests that not only biological but also psychosocial factors play an important role in the development and maintenance of the condition [3]. Cognitive behavioral therapy (CBT) derives from corresponding disorder models that assume interactions among biological/physical, psychological, and social factors [4]. CBT has been shown to be one of the most effective psychological treatments for CFS. In CFS, CBT is based on assumptions about the interaction of cognitive processes and behaviors, which contribute to the perpetuation of the ailments. It usually involves identifying the patient's negative thoughts, beliefs [5], and behaviors believed to contribute to the physical symptoms [6], most importantly, patients’ focus on perceived symptoms of fatigue is decreased [7]. The therapist helps the patient develop altered and more realistic views on their illness, and coping skills to manage their symptoms [8]. Thus, patients experience reversibility of symptoms, which results in enhanced self-efficacy [9]. The goal is to help patients gradually increase their activity levels and thereby to decrease impairments (e.g., [8]). Previous studies indicate short-term efficacy of CBT in reducing symptoms, enhancing quality of life, and improving physical functioning in patients with CFS. However, the evidence base for the long-term efficacy remains unclear [10].

Several previous meta-analyses have examined the efficacy of CBT in the treatment of CFS. Malouff et al.’s [11] meta-analysis (*k* = 13) found significant reductions in fatigue for CBT compared with inactive and non-specific control conditions (*d* = 0.54, 95%CI 0.26 to 0.81). Functioning was significantly reduced in subjective (*d* = 0.45, 95%CI 0.12 to 0.78) as well as objective measures (*d* = 0.52, 95%CI 0.28 to 0.76). However, not all evaluated interventions entailed cognitive components. The Cochrane review (*k* = 15) by Price et al. [8] resulted in small to moderate effects, e.g., for fatigue severity (*SMD* = -0.39, 95% CI -0.0.60 to -0.19), physical functioning (*SMD* = 0.11, 95%CI -0.32 to 0.54), depression (*SMD* = -0.24, 95%CI -0.53 to 0.05), and anxiety symptoms (*SMD* = -0.30, 95%CI -0.59 to -0.01) compared with inactive control conditions. Although meta-analytic results indicate significant efficacy in fatigue, functional impairment/quality of life, depression, and anxiety at short-term follow-up, long-term efficacy remains largely unclear [8, 12].

Despite these positive findings, the use of CBT as a treatment for CFS is controversial. The preceding version of the National Institute for Health and Care Excellence (NICE) guideline [13] recommended CBT for myalgic encephalomyelitis/CFS. However, the updated guideline published in 2021 [14] no longer recommends CBT as a curative, but only as an adjunctive treatment. This change has generated debate and criticism, in part due to the methods chosen by NICE to make the decision [15]. The debate highlights the need for up-to-date research on the efficacy of CBT in the treatment of CFS. Previous meta-analyses point to the short-term efficacy of CBT for CFS [8, 11, 12]. However, their publication dates back 10 to 15 years, so their results may now be outdated and not fully representative of the current state of research. A recent systematic review showed that several studies have been published since then, that could provide valuable information [16].

Furthermore, patient acceptance is an important factor in the evaluation of CFS treatment. In this context, patient acceptance of psychological treatments is of interest. If a treatment is not acceptable to patients despite demonstrated positive outcomes, it may not be effective in practice [17]. In research, drop-out might decrease the validity of the conclusions drawn from clinical trials. It is possible that it leads to a form of selection bias within randomized controlled trials [18], when people refuse to start or finish a therapy. Furthermore, psychotherapies might not work in full effect, when not adequately completed. Therefore, non-adherence can have negative consequences for patients and can increase health care costs [17]. For CFS, the review by Price et al. [8] reported an odds ratio of 1.77 (95%CI 1.13 to 2.75) for drop-out of the intervention arms compared to usual care. Malouff et al. [11] reported a mean CBT drop-out rate of 12% with a range of 0–42%. Castell et al. [12] found a median drop-out rate of 17% with a range of 0–46%. However, none of these meta-analyses differentiated between different stages of drop-out; they conferred to the authors’ definitions of drop-out which are not necessarily pre-defined or uniform. Hence, the implications that can be drawn from these results are limited.

One reason for the lack of willingness to engage in psychological therapy could be the discrepancy between the subjective explanatory concepts of some people with CFS and CBT models. That is, patients that suffer from CFS often have their own explanations about the causes and nature of their illness. When these subjective explanatory models are primarily physical and not related to psychological processes (such as appraisals and dysfunctional coping behavior), they may be hesitant to engage in a therapy that emphasizes these aspects [19]. Furthermore, patient characteristics like fatigue severity or comorbid psychopathology could determine whether a patient is able and willing to participate in CBT (e.g., [20]). Other aspects of the treatment itself, such as format in individual or group intervention, might influence the acceptability of treatments for patients as well [8].

Therefore, an updated meta-analysis should examine not only the efficacy of CBT, but also its acceptability to patients. To address these questions, first, a meta-analysis of randomized controlled trials (RCTs) to evaluate the efficacy of CBT in adults with CFS was conducted. Our objective was to evaluate the outcomes fatigue, depression, anxiety, and functional impairment post-treatment and to determine whether these effects persist in the long term. Secondly, we analyzed acceptance of CBT in a very differentiated way, i.e., drop-out rates at different stages of the trials. Here, the primary outcomes were non-completion of all mandatory sessions, and drop-out according to the primary study definition. Additionally, treatment refusal (non-starters), and the average number of sessions completed were included. Besides, for both parts of the project, differentiated moderator analyses served to identify the impact of study design, treatment format and participant characteristics on treatment efficacy and acceptance, e.g., regarding the different diagnostic criteria and control groups used in the studies, intervention related variables (treatment intensity and therapy setting), and – for the acceptance analyses – clinical variables (fatigue severity and duration of fatigue symptoms).

## Methods

The reporting of this meta-analysis followed the Preferred Reporting Items for Systematic Reviews and Meta-Analyses (PRISMA) 2020 statement [21]. A protocol for this project is available under: https://osf.io/2je7u. Furthermore, the data and R codes are available: https://osf.io/wq4gj/.

### Eligibility Criteria

Studies were considered eligible for inclusion if they met the following criteria: (a) participants were adults (≥ 18 years old) diagnosed with CFS according to any of the recognized diagnostic criteria (e.g., Oxford criteria [22] or Fukuda definition [1]; see Supplement for a complete list of eligible diagnostic criteria) or a score above a certain threshold on a validated symptom scale; (b) the study was a randomized controlled trial that compared CBT to inactive or non-specific control groups; (c) each arm relevant to this project was *n* ≥ 10 participants [23]; (d) the study was published in English or German.

### Literature Search

A systematic literature search was conducted in six databases, including PubMed, PsycINFO, PSYNDEX, CENTRAL, CDSR, and CCA; the final search was on 1st of June 2022. Results were filtered for *randomized controlled trial*, *meta-analysis*, and *systematic review* in PubMed*,* and for *clinical trial*, *meta-analysis*, and *systematic review* in PsycINFO. The search in PSYNDEX was limited to articles published in *academic journals*; consequently, grey literature, i.e., unpublished reports, was not included. The search algorithm for PubMed in the Supplement provides an example for the search strategies used.

### Study Selection

For study selection, abstracts were scanned for eligibility after the removal of duplicates by two independent researchers. Afterwards, the full articles of the reports that had not been excluded in the screening process were assessed for eligibility. During an additional backwards search, the references of meta-analyses and systematic reviews found during the first phase were searched for further eligible reports. The final selection was discussed between all authors. None of the independent researchers were blinded to any aspects of the studies at any time during the process.

### Data Extraction

Data were extracted from eligible studies by two independent reviewers using a standardized sheet. The extracted data was then again checked by ACKB. A complete list of extracted variables is provided in the Supplement. When reported, results of intent-to-treat-analyses were preferred; as were validated measures, if multiple measures were used for the assessment of the same outcome. Furthermore, two independent researchers assessed the risk of bias (RoB) using the revised Cochrane risk of bias tool for randomized trials for the efficacy outcomes (RoB 2.0, [24]). As the tool was not developed for acceptance outcomes, we did not use it for these.

### Outcome Variables and Data Analysis

We used a random effects model to estimate the effect sizes, and heterogeneity was assessed using the *I*^2^ statistic. The primary analysis was a meta-analysis of the efficacy of CBT in the treatment of CFS. The primary outcome was *fatigue* (physical and mental). Secondary outcomes were *depression*, *anxiety*, and *perceived health status* (including functional disability and quality of life). Outcomes were examined for post-treatment assessment, for short-term (up to 3 months) and long-term follow-up assessments (3–12 months) [25], as well as any follow-up intervals over a year. The standardized mean difference was calculated using Hedges’ *g*, with negative effects indicating a larger symptom reduction in treatment groups for fatigue, depression, and anxiety compared to the control groups; while positive effects on perceived health status suggest a larger improvement in treatment groups.

In the meta-analyses of the acceptance of CBT in CFS, we distinguished different forms of drop-out: The outcomes of interest were *non-completion* of all mandatory sessions, *drop-out* according to the primary study definition, *treatment refusal* (non-starters), and *average number of sessions completed*. On the one hand, non-completion was defined as all-cause discontinuation [26] after the treatment has been started, i.e., a unilateral termination of treatment despite therapeutic need [27] and against therapeutic advice. Thus, non-completion can be considered as the proportion of participants who started the treatment and completed at least one treatment module, but did not complete all sessions: (*n* participants not completing all sessions) / (*n* participants starting intervention). On the other hand, treatment refusal was defined as the proportion of participants in each group that did not start the intervention after being allocated and thus did not complete any module: (*n* participants not starting treatment) / (*n* participants allocated to intervention group) [28].

In accordance with former meta-analyses examining acceptance, we also included the primary authors’ definition of drop-out: (*n* participants dropping out according to authors’ definition) / (*n* participants starting intervention). Lastly, the average proportions of sessions completed was examined to estimate the amount of therapy participants, who started therapy, received: (average number of sessions completed) / (total number of sessions).

The meta-analyses for non-completion, treatment refusal, and for the average proportion of sessions completed in the intervention groups was calculated using weighted rates. For studies in which a rate of non-completers or drop-outs was reported, but not the number of participants who started treatment, we “imputed” the number of participants, i.e., we used the number of participants allocated as an estimation of starters. Non-completion and drop-out were also assessed for the control groups, and compared to the intervention groups using Relative Risk (*RR*). However, distinguishing these forms of drop-out was not possible in most inactive control groups. Lastly, the reasons for discontinuations were extracted for all acceptance outcomes, if available.

### Moderator Analyses

We conducted moderator analyses to explore the influence of study-related, intervention-related variables, and clinical variables on efficacy and acceptance of CBT in CFS: The choice of the control group was examined by comparing the effects of non-specific (treatment as usual, TAU; psychological and attention placebo groups) and inactive control groups (wait-list, WL). Furthermore, the therapy setting, therapy dosage (i.e., total therapy time in minutes), number of sessions, and duration of treatment (in weeks) were examined as potential moderators to judge the effect of intervention characteristics. As in former meta-analyses, we planned to examine differences between study outcomes considering the diagnostic criteria used for inclusion. We also examined the influence of fatigue severity and the duration of fatigue symptoms at baseline on non-completion, drop-outs, and average proportion of sessions completed. Regarding the other outcomes this was not feasible. For both, meta-regressions and subgroup analyses, we used a mixed-effects model with a true overall effect for each subgroup and random effects within subgroups. Subgroups had to be at least *k* ≥ 3 to be included in the analyses. Variables were dummy coded to dichotomize categories if necessary.

### Sensitivity Analyses

First, outlier studies were identified and every study’s individual influence on effect sizes and heterogeneity was analyzed. Further, to evaluate the use of the random effects model, results in the meta-analyses using fixed effect and random effects models were compared. Lastly, we recalculated the analyses excluding those studies for which we “imputed” the number of starters in the calculation of non-completion and drop-out rates.

### Publication Bias

Publication bias was assessed for every efficacy outcome using contour-enhanced funnel plots and Egger's regression test. Furthermore, using the *p*-curve method the distribution of statistically significant *p*-values for anomalies was examined.

## Results

### Study Selection and Characteristics

**Fig. 1:**
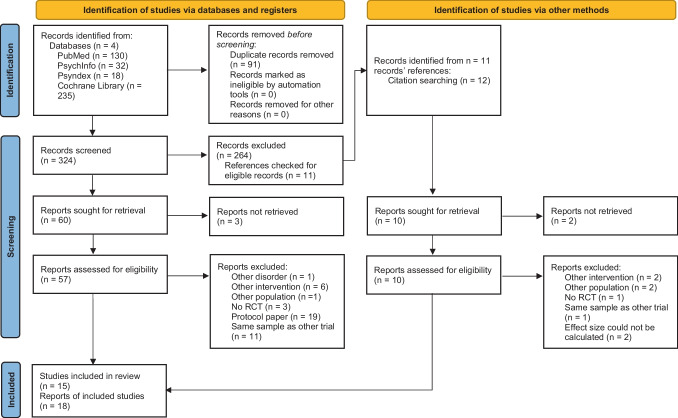
PRISMA 2020 flow diagram (adapted from Page et al. [21])

The initial search yielded a total of 415 articles, of which 91 were duplicates. The remaining 324 articles’ titles and abstracts were screened for eligibility, and 253 articles were excluded as they did not meet inclusion criteria (Fig. 1). The full texts of the remaining 57 articles were assessed for eligibility, and 41 studies were excluded. Through other methods, like backwards search, two relevant reports were identified. Finally, *k* = 15 studies with *n* = 2015 participants were included in the meta-analysis (with data reported in 18 manuscripts). There was no indication that eligible RCTs published in other languages were excluded during the course of the systematic literature search.

The included studies were conducted in Europe (*k* = 6 in Great Britain, *k* = 5 in the Netherlands, *k* = 1 in Norway) and US (*k* = 3); they were published between 1996 and 2021. Most studies (*k* = 10) used the international definition criteria (Centers for Disease Control and Prevention, [1]) for inclusion of CFS patients, while the other studies either used the Oxford criteria ([22], *k* = 3), or cut-off values (*k* = 2). The sample sizes of the studies ranged from 37 to 321 participants that were allocated to treatment arms. The duration of treatments varied from 8 to 36 weeks (*M* = 18.85, *SD* = 8.52), with a range of two to 17 sessions (*M* = 10.71, *SD* = 4.83), and a total time of direct contact from 2 to 28 h (*M* = 12.75, *SD* = 7.82). One study used an internet-based self-help form of CBT [29], another used a video-telephone-based program [30], three studies implemented CBT in a group setting [31–33]. All studies compared CBT to inactive or non-specific control groups, namely *k* = 5 WL, *k* = 6 TAU, *k* = 3 psychological placebo (in the form of relaxation [34, 35] or a health promotion group [30]), and one study with an attention placebo control group (symptom monitoring [36]). That is, *k* = 5 studies used an inactive control group (WL), while the other *k* = 10 studies used non-specific controls (see Tables 1 and 2 for further study characteristics).

**Table 1:** Study Characteristics

Study	Conditions	*n1/n2*	Setting	Guidance	Criteria	Country	Specific Target Group	No. Sessions	Time total^a^	Duration of treatment (weeks)	Follow-ups^b^ (weeks)
Deale et al. [34]	CBT/Psy plac	30/30	Individual		OC	GB	Adults	13	900	20	24, 240
Friedberg et al. [36]	CBT/Att plac	37/38	Individual	Para	CDC	US	Adults	2	120	14	48
Gotaas et al. [40]	CBT/WL	78/80	Individual	Psy	CDC	NO	Adults	17		17	36
Huibers et al. [46]	CBT/TAU	76/75	Individual	GP	Cut-Off	NL	Workers	6	180	16	16, 32, 176
Janse et al. [29]	CBT/WL	160^c/^80	Individual	Psy	CDC	NL	Adults	7			
Jason et al. [35]	CBT/Psy plac	29/28	Individual	Para	CDC	US	Adults	13	585	26	48
Knoop et al. [37]	CBT/WL	85/86	Individual	Psy	CDC	NL	Adults				
Milrad et al. [30]	CBT/Psy plac	75/75	Individual		CDC	US	Couples	10	600	10	
O’Dowd et al. [31]	CBT/TAU	52/51	Group	Mixed	CDC	GB	Adults	8	960	16	32
O’Dowd et al. [43]	CBT/TAU	28/16	Individual	GP	Cut-Off	GB	Adults	4	120	10	14
Prins et al. [41]	CBT/TAU	93/91	Individual	Mixed	CDC	NL	Adults	16	960	32	24
Rimes and Wingrove [32]	MBCT/WL	18/19	Group	Psy	CDC	GB	Adults	9	1215	8	8
Sharpe et al. [44]	CBT/TAU	30/30	Individual	Psy	OC	GB	Adults	16	960	16	16, 32
White et al. [42]	CBT/TAU	161/160	Individual	Mixed	OC	GB	Adults	15	900	36	28, 96
Wiborg et al. [33]	CBT/WL	136/68	Group	Mixed	CDC	NL	Adults	14	1680	24	

*Att plac* attention placebo, *CBT* cognitive behavioural therapy, *CDC* international centers for disease contol and prevention criteria, *GB* United Kingdom, *GP* general practitioner, *MBCT* mindfulness-based cognitive therapy, *Mixed* guidance provided by psychologist and psychiatrist, *n1* intervention group, *n2* control group, *NL* Netherlands, *NO* Norway, *OC* oxford criteria, *Para* paraprofessional (study nurses, trained students), *Psy* trained clinician (licensed psychotherapist/trained psychologist), *Psy plac* psychological placebo, *TAU* treatment as usual, *US* United States of America, *WL* wait-list

^a^Total time of direct contact in minutes (was not applicable for Janse et al. [29] and Knoop et al. [37])

^b^After end of treatment (short-term follow-up = ≤ 12 weeks, long-term follow-up = 13 to 48 weeks, follow-up longer than 12 months = > 48 weeks)

^c^Two intervention groups (ICBT with feedback on demand & ICBT with protocol-driven feedback) were merged for conducting the analyses

**Table 2:** Additional information on acceptance outcomes

Study	Conditions	*n1/n2*	Drop-out definition	Prop. drop-outs	Prop. treatment refusal^a^	Prop. non-completion^a^	Prop. average sessions completed
Deale et al. [34]	CBT/Psy plac	30/30				0.10 [0.02; 0.27]	
Friedberg et al. [36]	CBT/Att plac	37/38		0.23 [0.10; 0.42]		0.19 [0.08; 0.35]	
Gotaas et al. [40]	CBT/WL	78/80		0.21[0.12; 0.32]	0.01 [0.00; 0.07]	0.23 [0.82; 0.93]	
Huibers et al. [46]	CBT/TAU	76/75	Not completing CBT/ Not completing post assessment	0.28[0.18; 0.40]	0.06 [0.02; 0.15]		0.88 [0.39; 0.99]
Janse et al. [29]	CBT/WL	160^b^/80	Opening all modules + emailing fortnightly/ Not completing post assessment	0.31 [0.24; 0.39]	0.06 [0.03; 0.11]	0.88 [0.82; 0.93]	
Jason et al. [35]	CBT/Psy plac	29/28	Completing < 4 sessions/ Completing < 4 sessions				0.77 [0.48; 0.92]
Knoop et al. [37]	CBT/WL	85/86			0.20 [0.12; 0.30]		
Milrad et al. [30]	CBT/Psy plac	75/75		0.05 [0.01; 0.13]			0.93 [0.54; 0.99]
O’Dowd et al. [31]	CBT/TAU	52/51					
O’Dowd et al. [43]	CBT/TAU	28/16			0.39 [0.22; 0.59]	0.41 [0.18; 0.67]	
Prins et al. [41]	CBT/TAU	93/91	Formally withdrawing CBT/ Not attending assessments	0.28 [0.18; 0.39]	0.11 [0.05; 0.19]		
Rimes and Wingrove [32]	MBCT/WL	18/19	“Discontinued MBCT”/ Not completing post assessment	0.06 [0.00; 0.29]	0.06 [0.00; 0.27]		0.81 [0.45; 0.96]
Sharpe et al. [44]	CBT/TAU	30/30	No drop-outs reported	0.00 [0.00; 0.12]	0.00 [0.00; 0.12]	0.00 [0.00; 0.12]	
White et al. [42]	CBT/TAU	161/160	Completing < 10 sessions/ Completing < 3 sessions	0.11 [0.06; 0.17]	0.02 [0.00; 0.05]		
Wiborg et al. [33]	CBT/WL	136/68	“Discontinued intervention”/ Not completing post assessment	0.15 [0.09; 0.22]	0.12 [0.07; 0.18]		

*Att plac* attention placebo, Average proportions of sessions completed = (average number of sessions completed) / (total number of sessions), *CBT* cognitive behavioural therapy, Drop-outs = primary authors’ definition of drop-out: (*n* participants dropping out according to authors’ definition) / (*n* participants starting intervention), *n* number of participants allocated to each group, *n1* intervention group, *n2* = control group, Non-completion = prop. of participants who started treatment and completed at least one session: (*n* participants not completing all sessions) / (*n* participants starting intervention), *Psy plac* psychological placebo, *TAU* treatment as usual, *WL* wait-list

^a^A table with additional information on reasons is provided in the Supplement

^b^Two intervention groups (ICBT with feedback on demand & ICBT with protocol-driven feedback) were merged for conducting the analyses

There were two stepped-care studies that built up on two included studies: [20] used the same sample as [37], while [38] recruited additional participants that were added to the sample in [29]. To avoid the problem of dependent samples, we did not include the second parts of these studies. Furthermore, [39] incorporated CBT in a multidisciplinary intervention, but graded exercise predominated. Therefore, we did not include the study in the analyses.

For studies with multiple arms we combined similar arms where possible: For [29] there were two similar internet-based CBT arms, one facilitated protocol-driven therapist feedback, in the other one, therapist feedback was provided on demand. In [33] the CBT conditions only differed in group sizes (either four participants and one therapist or eight participants and two therapists per group). For other studies, some additional arms could not be included in the analyses: The shorter CBT arm from [40] as well as the cognitive therapy arm from [35] relied on a different rational than the elaborated CBT arms in both studies and were therefore excluded. Since the control groups in [36], i.e., the symptom monitoring support and TAU, were not comparable, TAU was excluded. Additionally, ineligible control groups were excluded: Anaerobic activity therapy [35], education and support [31], (active) guided support groups [41], graded exercise, and adaptive pacing [42].

The primary outcome measure for the efficacy of CBT was fatigue, which was assessed in 14 of the trials. Most used either the Checklist Individual Strength – Fatigue subscale (*k* = 5), or the Chalder Fatigue Questionnaire (*k* = 5, one using an adapted version of the scale; see Supplement for more information on outcome measures). For [35] and [43] no means were reported at post-treatment, and for [44] no effect could be calculated as *SD*s were not reported. Depression was mostly assessed using the Hospital Anxiety and Depression Scale – depression subscale (*k* = 6), or the Beck Depression Inventory (*k* = 2). Anxiety was rated on the Hospital Anxiety and Depression Scale – anxiety subscale (*k* = 6) and the Beck Anxiety Inventory (*k* = 2). Perceived health status was mainly assessed using the Short Form Health Survey – physical functioning subscale (*k* = 9).

### Efficacy of CBT

**Fig. 2:**
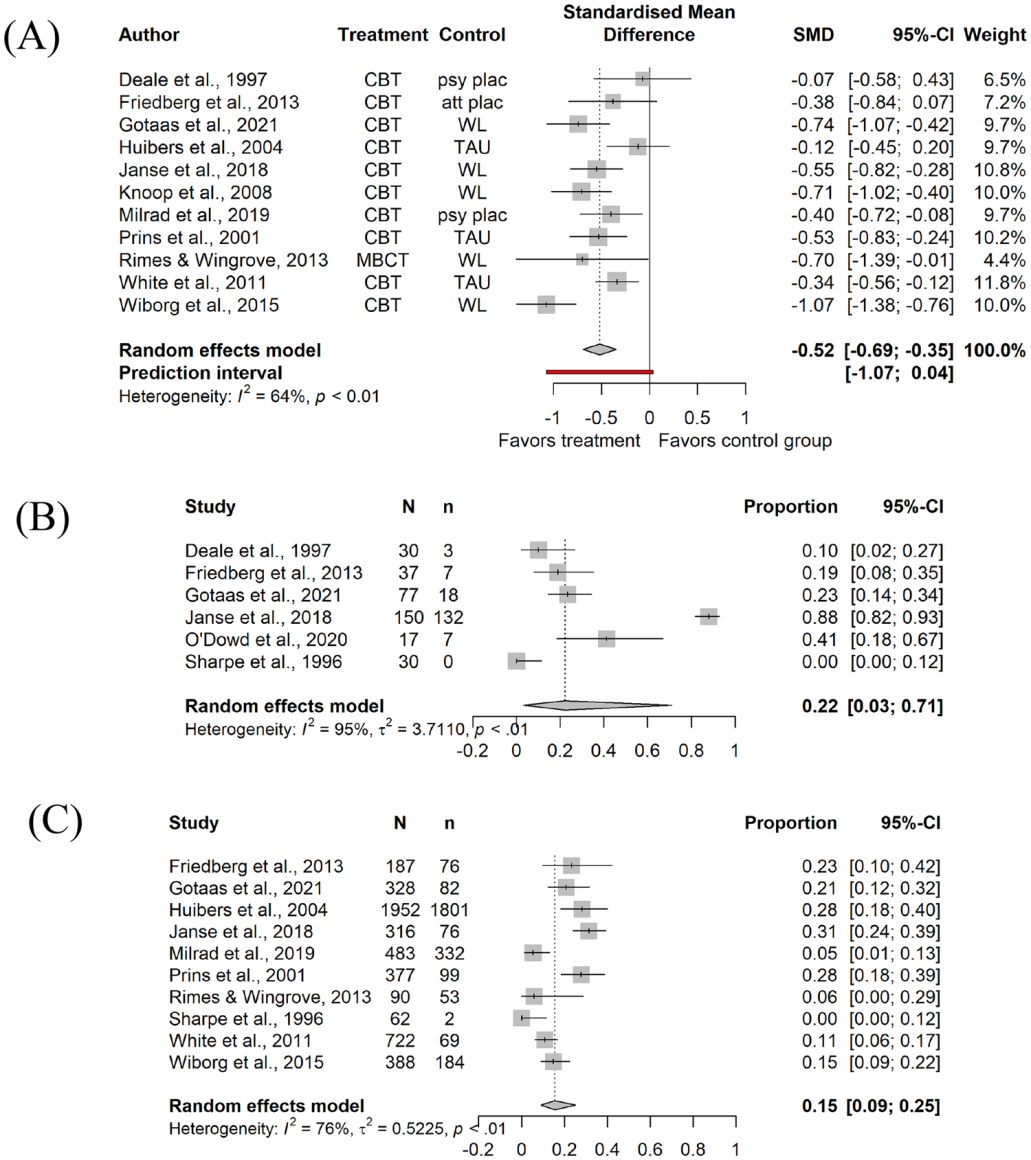
Forest plots for primary outcomes for fatigue, non-completion, and drop-out. **A** forest plot for fatigue (post-treatment); **B** forest plot for non-completion in CBT groups; **C** forest plot for drop-out in CBT groups

The meta-analytic results (*k* = 11) showed that CBT was significantly more effective in reducing fatigue than the control conditions at post-treatment *g* = -0.52 (95%CI -0.69 to -0.35; Fig. 2A). As only one study’s follow-up met our criterion for short-term follow-up [32], no results could be aggregated for any outcome. At long-term follow-up (*k* = 7), with a mean follow-up duration of 31.14 weeks (*SD* = 12.75), the effect on fatigue was also significant: *g* = -0.41 (95%CI -0.65 to -0.18). The heterogeneity across the studies was *I*^2^ = 64.4% (95%CI 32.1 to 81.3), indicating a moderate to high level of variability between the studies at post-treatment, and no to high heterogeneity at long-term follow-up (*I*^2^ = 57.9%, 95%CI 2.5 to 81.8). For follow-ups longer than 12 months post-treatment, two studies provided information on fatigue severity: [45] reported a ~ 3.5-year follow-up to [46] (*g* = 0.12, 95%CI -0.23 to 0.47), and [47] reported a two-year follow-up to [42] (*g* = -0.21, 95%CI -0.47 to 0.05).

For the secondary outcomes, CBT was significantly more effective in reducing depression and anxiety than the control conditions at post-treatment with small to moderate effects (Table 3). Furthermore, perceived health status was significantly improved in the CBT groups with a small effect. At long-term follow-up, neither the effects on perceived health status, nor on depression were significant (see Table 3; see Supplement for the forest plots). However, anxiety showed a significant small effect. It is to note, that the individual effects’ 95%CIs included 0, but the aggregated effect did not and was therefore significant. Of the secondary outcomes, only perceived health status was reported at follow-ups longer than a year post treatment: *g* = -0.35 (95%CI -0.7 to 0.0) for [46], and *g* = 0.17 (95%CI -0.08 to 0.43) for [42].

**Table 3:** Additional information on treatment effects on outcomes at post-treatment, at follow-up, and for indicators of acceptance

Outcome	*k*	*n*^a^	Pooled effect^b^	95%CI	*p*	*I*^2^	95%CI	95%PI
Post-treatment								
Fatigue	11	1727	-0.52	-0.69; -0.35	< 0.001	64.4%	32.1; 81.3	-1.07; 0.04
PHS	11	1679	0.29	0.11; 0.47	0.002	65.3%	34.2; 81.8	-0.30; 0.88
Depression	5	422	-0.36	-0.55; -0.17	< 0.001	0.0%	0.0%; 79.2%	-0.67; -0.05
Anxiety	3	212	-0.34	-0.62; -0.07	0.01	0.0%	0.0; 89.6	-2.11; 1.42
Long-term follow-up								
Fatigue	7	850	-0.41	-0.65; -0.18	< 0.001	57.9%	2.5; 81.8	-1.09; 0.26
PHS	8	1107	0.15	-0.18; 0.47	0.37	76.6%	53.5; 88.3	-0.93; 1.22
Depression	6	631	-0.15	-0.31; 0.00	0.06	0.0%	0.0; 74.6	-0.37; 0.07
Anxiety	5	571	-0.20	-0.36; -0.03	0.02	0.0%	0.0%; 79.2%	-0.46; 0.07
Acceptance								
Non-completion	6	341	0.22	0.03; 0.71		95.4%	92.2; 97.2	0.00; 0.99
Drop-out	10	812	0.15	0.09; 0.25		75.7%	54.9; 86.9	0.03; 0.52
Treatment refusal	10	865	0.07	0.03; 0.15		80.3%	64.6; 89.0	0.01; 0.52
Ave. prop. sessions	4	198	0.84	0.56; 0.96		0.0%	0.0; 84.7	0.44; 0.97

*95%CI* 95% confidence interval, *95%PI* 95% prediction interval, Ave. prop. sessions = (average number of sessions completed) / (total number of sessions); Drop-out = primary authors’ definition of drop-out: (*n* participants dropping out according to authors’ definition) / (*n* participants starting intervention), *g* Hedges’ *g*, *k* number of studies, Long-term follow-up = 13 to 48 weeks after post-treatment, *n* number of participants, Non-completion = prop. of participants who started treatment and completed at least one session: (*n* participants not completing all sessions) / (*n* participants starting intervention), *PHS* perceived health status; Treatment refusal = prop. of participants that did not start the intervention: (*n* participants not starting treatment) – (*n* participants allocated to intervention group)

^a^For the acceptance outcomes, only the participants in the intervention groups were considered

^b^Hedges’ *g* for efficacy outcomes, proportions for acceptance outcomes

### Acceptance of CBT

The results of the meta-analyses on acceptance showed that the non-completion in the CBT groups was 22% (0.22, 95%CI 0.03 to 0.71, Fig. 2B. Using each study’s drop-out definition (*k* = 10), the overall weighted rate was 15% (0.15, 95%CI 0.09 to 0.25, Fig. 2C). Four studies reported the average numbers of sessions completed, and for those the average proportion of modules was 84% (0.84, 95%CI 0.56 to 0.96). Ten studies reported the numbers of participants who were allocated, but did not start CBT. The weighted rate of treatment refusal was 7% (0.07, 95%CI 0.03 to 0.15). Reasons for drop-out were rarely reported (see Supplement for a list of all reasons).

### Relative Risks

In the calculations of the *RR* in the acceptance outcomes, the *RR* for non-completion (*k* = 5) was *RR* = 3.87 (95%CI 0.30 to 49.63) with a heterogeneity of *I*^2^ = 89.2% (95%CI 77.5 to 94.8). For drop-out (*k* = 9) the aggregated *RR* was 2.26 (95%CI 1.05 to 4.86; *I*^2^ = 67.6%, 95%CI 34.7 to 83.9). As one study [44] reported that there was neither drop-out nor non-completion in any group, it could not be incorporated in the analysis.

### Moderator Analyses

Moderator analyses were conducted to examine the effects of different variables on the efficacy and acceptance of CBT. The moderator analyses for efficacy outcomes were limited to post-treatment due to the small number of studies in the follow-up intervals. First, the subgroup analysis on control groups showed that the effects on fatigue between CBT and inactive control groups (*g* = -0.76, 95%CI -0.96; -0.56) were larger than the effects between CBT and non-specific control groups (*g* = -0.34, 95%CI -0.47; -0.21) at post-treatment (Table 4). There was no significant difference between subgroups for perceived health status or drop-out. Subgroups were too small for depression, anxiety, non-completion, and average proportion of sessions completed. The differences in therapy setting, i.e., individual and group setting, were not significant for perceived health status. The subgroups were too small to be analyzed for all other outcomes.

**Table 4:** Moderator analyses – subgroup analyses

Subgroup analysis	*k*	Pooled effect^a^	95%CI	*τ*^2^	*I*^2^	${p}_{subgroup}$
*Control condition*^b^						
Fatigue						0.0006
Non-specific	6	-0.34	-0.47; -0.21	0	0.0%	
Inactive	5	-0.76	-0.96; -0.56	0.02	36.7%	
Perceived Health Status						0.31
Non-specific	6	0.21	-0.09; 0.50	0.10	74.7%	
Inactive	5	0.39	0.20; 0.57	0.02	29.1%	
Drop-out						0.33
Non-specific	6	0.13	0.05; 0.31	0.82	77.8%	
Inactive	4	0.20	0.09; 0.37	0.17	76.2%	
*Therapy setting*^c^						
Perceived Health Status						0.98
Individual	8	0.29	0.08; 0.50	0.06	68.3%	
Group	3	0.29	-0.15; 0.72	0.10	70.1%	

Effects on efficacy outcomes at post-treatment

^a^Hedges’ *g* for efficacy outcomes, proportions for acceptance outcomes

^b^*k* < 3 for depression, anxiety, non-completion, and average proportion of sessions completed

^c^*k* < 3 for fatigue, depression, anxiety, non-completion, drop-out, treatment refusal, and average proportion of sessions completed

Therapy dosage was a significant moderator for fatigue (*R*^2^ = 91.17%, *p* = 0.0003), i.e., a higher dosage was associated with a greater reduction in fatigue. However, the meta-regressions for perceived health status, depression, anxiety, non-completion, drop-out, and average proportion of sessions completed were not significant. The meta-regression on number of sessions was significant for perceived health status (*R*^2^ = 49.21%, *p* = 0.03), indicating that more sessions are associated with a greater improvement in the perceived health status, but not significant for any other outcome. Duration of therapy in weeks was not a significant moderator for any outcome either. Subgroup analyses for diagnostic criteria ratings were not possible as there were not at least two groups with *k* ≥ 3.

Fatigue severity was a significant moderator for non-completion and drop-out. That is, higher fatigue prior to treatment was associated with greater non-completion and higher drop-out. However, it was not significant for the average proportion of sessions completed. Duration of fatigue symptoms was neither a significant moderator for non-completion nor for drop-out. As only two studies reported information on the average proportion of sessions completed and the duration of symptoms, no analysis was conducted for this outcome. The detailed results of the meta-regression analyses are presented in the Supplement.

### Sensitivity Analyses

We did not detect any outliers for depression, anxiety, drop-out, and the average proportion of sessions completed. For fatigue, there was one outlier [33] as well as for perceived health status [46], non-completion [29], and treatment refusal [43]. While the mean estimated effects did not change drastically after exclusion, heterogeneity decreased for all four outcomes.

When comparing the results using a random effects model (0.22, 95%CI 0.03 to 0.71) and a fixed effect model (0.49, 95%CI 0.44 to 0.54), there was only a difference in the estimate of the non-completion rate. Other than that, the confidence intervals tended to be wider in the analyses using a random effects model. The exclusion of the studies using the number of participants allocated to the intervention group when the number of treatment starters was not given, did not result in meaningful differences for non-completion or drop-out (see Supplement for detailed sensitivity analyses).

### Risk of Bias

**Fig. 3:**
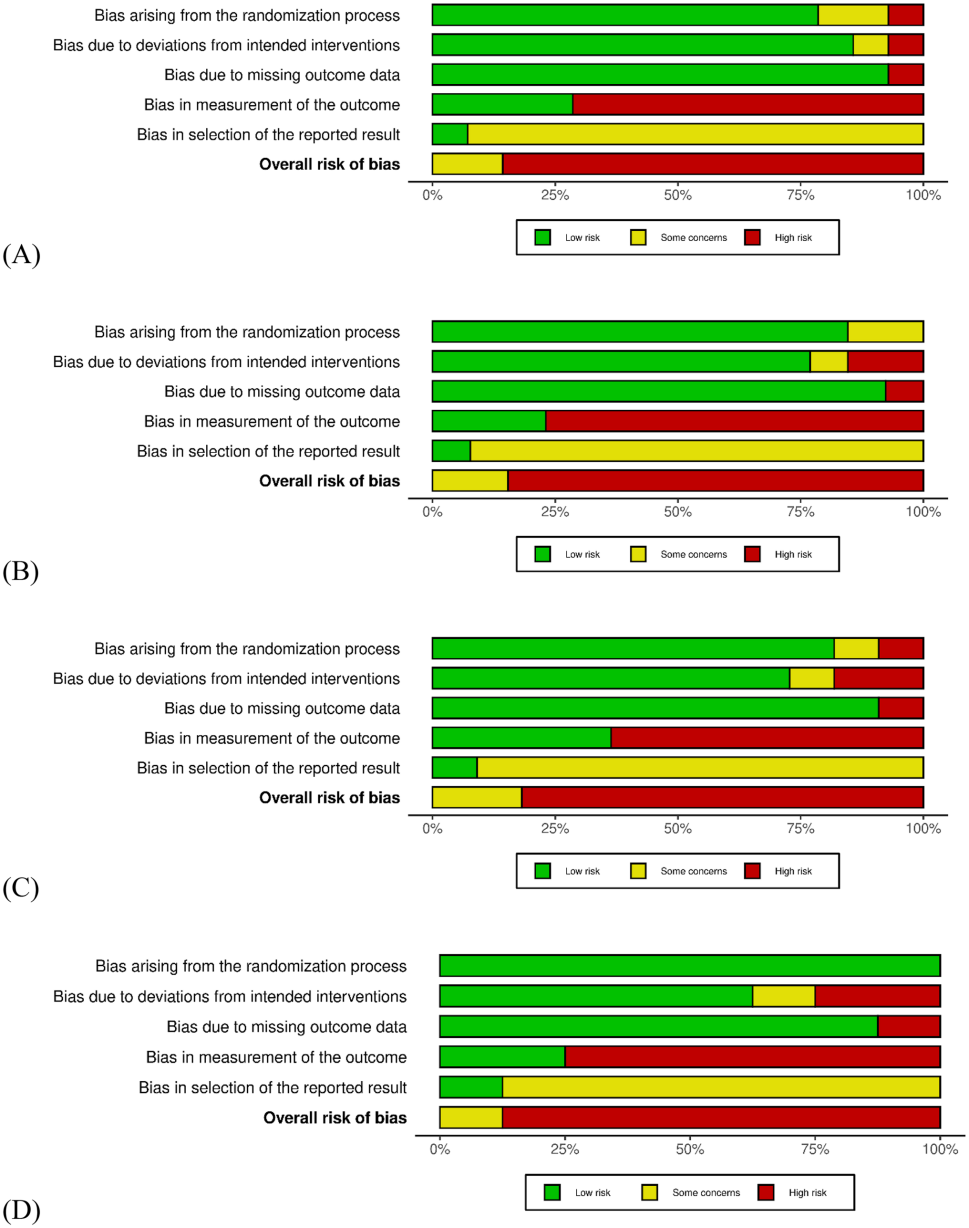
Risk of bias ratings for fatigue, perceived health status, depression, and anxiety at post-treatment. **A** summary plot for fatigue; **B** summary plot for perceived health status, **C** summary plot for depression, **D** summary plot for anxiety. Additional information is provided in the Supplement

Overall RoB across all studies was either rated *high* or *some concerns* (Fig. 3). However, the RoB 2.0 tool might lead to higher ratings of conventional psychotherapeutic trial designs (cf. [48]).

### Publication Bias

Egger’s regression test did not indicate a possible publication bias in any of the efficacy outcomes at post treatment. While there was a significant result for anxiety at long-term follow-up, the test was solely based on five studies. The *p*-curve analyses indicated that the evidence on fatigue and perceived health status at post-treatment and for fatigue at long-term follow-up was based on evidential value. The funnel plots and *p*-curves are provided in the Supplement.

### Deviations from the Protocol

There resulted some deviations from the protocol: The outcomes *mental fatigue* and *physical fatigue* could not be aggregated separately because the studies did not report fatigue divided into these domains. Additionally, the outcomes *participation refusal* and *total drop-out rate* were excluded from the analysis. It was found that there was no consistent reporting of participation refusal in studies, which could be attributed to different recruitment methods used that led to differently defined participant pools between studies. Thus, an aggregated proportion of participation refusal would have been difficult to interpret. Furthermore, the reporting of *treatment refusal* (non-starters) and *drop-out* in studies was not always clearly distinguishable, possibly leading to overlaps and an overestimation of the total drop-out rate. In contrast to the protocol, an interrater reliability was not applicable as the chosen procedure in this project relied on consensus within the author group and therefore some decisions were revised after collective discussion. No sensitivity analysis for risk of bias values was carried out either, as most ratings were *high* and an analysis was therefore not feasible. Lastly, moderator analyses were streamlined to enhance clarity in reporting, i.e., we unified the moderator analyses for efficacy and acceptance outcomes.

## Discussion

The results of this meta-analysis provide important insights into the efficacy and acceptance of CBT in the treatment of CFS in adults. Our findings confirm that CBT is an effective intervention for CFS. We found significant post-treatment effects of CBT on fatigue, depression, anxiety, and perceived health status. The effect on the primary efficacy outcome, fatigue, was moderate with a confidence interval ranging from small to moderate effects. The mean effects on the other efficacy outcomes were smaller, yet had wider confidence intervals. That is, there were small effects on depression, anxiety, and perceived health status in CBT compared to inactive and non-specific control groups. The follow-up effects suggest a partial maintenance, while the data base is slightly smaller than for post-treatment. The effect on fatigue was small with the upper end of the 95%CI ranging into moderate effects. For the secondary outcomes, neither the effect on depression nor on perceived health status was significant at long-term follow-up. In contrast, there was a small significant overall effect on anxiety. All in all, these results are in line with previous research [8, 11, 12, 16, 49], indicating that CBT can alleviate CFS symptoms and improve patients’ overall well-being. In fact, this analysis is based on a broader data base compared to previous meta-analyses. Yet, for some outcomes sample sizes are still relatively small. This is especially the case for depression and anxiety. Besides, this is the first project that provides meaningful aggregated long-term effects; in previous works, there were only short-term follow-up effects [8] or aggregation was not possible [12]. While there is a recently published individual patient data meta-analysis in CBT for CFS [49], the data base for that project was limited to a specific treatment-protocol (e.g., [50]) and therefore less generalizable.

In the light of the latest NICE guidelines [14], this meta-analysis sheds light on the acceptance of CBT in the treatment of CFS, which is an important aspect of treatment evaluation. The results showed that non-adherence rates in the CBT group were generally low, indicating that CBT was acceptable to patients. Over all studies, only 7% of participants refused participation after randomization. Of those who started the interventions, a mean of 15% dropped out of the treatment groups, and 22% did not complete all mandatory modules. That is, most participants completed all mandatory modules and even more received an adequate amount of treatment according to the individual study authors’ definitions. In line with this, some studies that reported the average proportion of sessions completed, and the aggregated rates indicate that patients on average complete most sessions. Although some clinicians argue that some patients discontinue treatment due to major improvements already made during the current path of treatment, this is rather unlikely [51]. While cases of clinically relevant worsening of symptoms due to CBT are rather rare in CFS [16], they might still have an increased risk of dropping out of treatment. In general, about one fifth of participants drop out of psychotherapy trials [28, 52]. That is, the findings on drop-out in this project are comparable to general reviews on drop-out in psychotherapy studies. Furthermore, the results on treatment refusal are comparable to treatment refusal rates in individual therapies for various psychological disorders as well [28].

In non-completion, the *RR* was not significant between intervention and control groups, but the confidence interval was wide, and heterogeneity was considerable. In contrast, the RR for drop-out was significantly larger in intervention groups, i.e., participants were more than twice as likely to drop-out of the intervention groups compared to the controls. Heterogeneity was moderate to considerable. However, in the non-specific, and especially in the inactive control groups, the definitions of dropping out of the control group and non-completion in the control group were not always clearly defined. Moreover, in most control groups, participation was low-threshold due to the inactive nature. Hence, the *RR*s cannot be adequately interpreted as they are most likely biased by this methodological artifact.

The reported analyses on efficacy and acceptance provide average effects, thereby complementing another recent review [16] that reported the proportion of clinically improved or worsened cases. Furthermore, the results of most analyses in this project showed a relevant amount of heterogeneity. This suggests that not every patient benefits from CBT, which is why an array of moderators was examined: Using moderator and sensitivity analyses, the effects on fatigue were larger when only compared to inactive control groups (moderate to large effect). However, the effects were still small to moderate, if CBT was compared to an intervention that controls for some factors such as therapist support, attention, or expectancy variables. Within the subgroup analyses, the 95%CIs of both groups did not overlap and heterogeneity was drastically reduced in both groups. There was a similar pattern for perceived health status, as the effect was only significant for inactive control groups, but not for non-specific control groups with a larger 95%CI for the latter (from no effect to a moderate effect). Yet, the subgroups did not differ significantly. For perceived health status, the therapy setting (group vs. individual) did not affect the outcome, but it’s potential impact on other outcomes could not be evaluated. Although it was not possible to calculate subgroup analyses based on the different diagnostic criteria used for inclusion of CFS patients, most included studies used well-established criteria [2]. For the two studies that used cut-off values as inclusion criteria, sensitivity analyses did not indicate a significant impact on results.

Furthermore, the influence of three variables of treatment intensity was examined: treatment dosage (total time in minutes), the number of sessions, and the duration of treatments in weeks. There was an effect of treatment dosage, as expected, for fatigue (higher dosage predicts a higher symptom reduction), and the moderator accounted for almost all heterogeneity (*R*^2^ = 91.17%). Although the included studies evaluated relatively short therapies with a range from 2 to 17 sessions, the number of sessions was a significant moderator for the effect in perceived health status. Duration of treatment (in weeks) neither affected efficacy nor acceptance. That is, treatment intensity may be a relevant moderator in CFS. Consequently, two indicators of treatment intensity each showed a relevant association with one of the two most frequently studied efficacy outcome measures. In contrast, they were not associated with the measures of treatment acceptance. Fatigue severity was associated with a higher non-completion rate – that is, if the mean on the fatigue scale rises one point, the proportion of non-completion rises 0.1. This was also true for drop-out, but to a lesser extent. That is, some individuals might not deem themselves able to participate in regular sessions due to their severe fatigue symptoms. Duration of symptoms at baseline neither moderated non-completion nor drop-out. The differentiated sensitivity analyses, which considered the meta-analytic model choice and the influence of specific studies indicated that the project’s results are robust. Additionally, there was no substantial indication of publication bias for the efficacy outcomes.

### Limitations and Future Directions

As this project’s focus was on investigating the absolute efficacy of CBT, which allows for a more accurate assessment of the efficacy and acceptance of the intervention [53], the interpretation of results is limited to this pool of studies. Consequently, the statements made apply primarily to CFS according to the Oxford criteria [22] and Centers for Disease Control and Prevention (CDC) definition [1], but not for myalgic encephalomyelitis/CFS according to the definition by the NICE guidelines [14]. However, a recently published individual patient data meta-analysis did not find evidence suggesting that patients meeting different case definitions or reporting additional symptoms benefited less from CBT [49]. Nonetheless, the selection of studies might have led to an overestimation of effects as effects are usually larger when compared to inactive and non-specific control groups [54]. Additionally, in line with Kim et al.’s [55] findings on measures used in CFS trials, the measures of fatigue and perceived health status are self-report ratings. This has partly been criticized [56]. However, this line of argument neglects the fact that those affected primarily report subjective suffering. The assessment of self-ratings is in line with the recommendations by the EURONET-SOMA group [57]. The same applies to comorbid psychopathology, which can be a predictor [58], a concomitant factor or a consequence of CFS [12]. Therefore, subjective experienced fatigue, comorbid psychopathology, and resulting disabilities have been chosen as key outcome measures. However, post-exertional malaise – which has been proposed as a cardinal symptom for myalgic encephalomyelitis/CFS in the latest NICE-guidelines [14]– could not be systematically aggregated as it was seldom assessed and not uniformly reported [29, 35, 42]. Future studies should take this into account. Furthermore, this project – as previous meta-analyses in the field – can neither answer which components of CBT are effective nor does it allow for therapy comparisons. Since, studies were considered here if they were based on a cognitive-behavioral rational rather than a purely behavioral rational, we cannot draw conclusions on differences between behavioral and cognitive components or the like.

Since the overall RoB was rated at least *some concerns*, but mostly *high* for the included studies, this should be taken into account when interpreting the results. Nonetheless, the high rating can partly be explained by the nature of conventional psychotherapeutic trial designs used for these studies. This does not imply that these designs are without flaw, however, these ratings do not render the results irrelevant. For example, one major criticism of psychotherapy trials in CFS is a lack of blinding [56]. However, this overemphasizes the assumed effect of blinding in trials, which is not reflected in clinical data [59].

Furthermore, there might be a form of selection bias in the examined sample since the included studies were conducted mainly in Europe and the US, which may limit the generalizability of the findings to other regions. More importantly, the included participants in the studies already agreed to participate in a RCT on CBT for CFS. That is, participants who are not willing to partake in CBT might simply refuse participation. Moreover, as most studies included at least some face-to-face contact, it might disadvantage those CFS patients who, due to their pronounced symptom severity, are housebound [16]. Therefore, conclusions on this subset of CFS patients cannot be drawn. Thus, it remains an open question as to which treatments or forms of treatment are appropriate for patients who are this severely impaired. Future studies should continue to systematically assess the reported efficacy outcomes, and should additionally be supplemented with objective measurement instruments. Thus, other observable indicators would also be of interest, such as behavior changes and sickness leave days. In particular, the catamnestic effects in CBT should be further investigated in large-scale trials. Currently, in study reports, specific forms of drop-out rates are seldom reported [17], and a consistent definition of drop-out is lacking [28]. While it is understandable that authors define an adequate amount of treatment received as the main indicator of acceptance, this leads to a certain amount of variance between studies’ definitions of adequate treatment. However, it is noticeable that across all studies, only a small proportion of studies reported uptake and discontinuation; which could indicate attrition bias [16]. Especially, the (non-)completion of all mandatory modules and information on the mandatory modules completed were only reported in some studies. In line with former meta-analyses on adherence, treatment refusal (e.g., [28]), and drop-outs (e.g., [17]) have been systematically aggregated here. Additionally, in this project, the analysis of non-completion and the average proportion of mandatory modules provided a new perspective on and operationalized measures of adherence and hence acceptance. Differentiated information on adherence is essential. Preferably, future studies should report the described variants of acceptance outcomes. Although there is now a broader database compared with the meta-analyses from 10 to 15 years ago, the subgroup analyses and, especially, the regression analyses to identify possible moderators of efficacy and acceptance require further primary studies.

## Conclusion

In conclusion, this meta-analysis provides further evidence for the efficacy of CBT in the treatment of CFS in adults and maintenance of the effects, while also highlighting the importance of considering the acceptance of treatments. Acceptance of CBT in CFS does not seem to be lower when compared with other patient groups with various mental disorders – this also applies if the stricter criterion of non-completion is taken into account. The results may help inform clinical practice and future research in this area. Hence, this project contributes to a better understanding of the potential benefits and limitations of CBT in the treatment of CFS and supports clinical decision-making. One reason why some people with CFS are reluctant to undergo psychological therapy could be the lack of willingness to engage in psychological therapy among people with CFS. This may stem from a mismatch between their personal beliefs about their condition and the foundational principles of therapies like CBT. Recognizing and addressing this disconnect is crucial for providing effective support and treatment for individuals with CFS, potentially by tailoring therapy to their unique needs and perspectives. Considering that initial fatigue severity was associated with a slightly increased risk of discontinuation of treatment, one could consider acceptance facilitating interventions – as shown in pain [60]. Similarly, stepped-care approaches might be promising in the field of CFS: first, the participants receive low-threshold intervention (e.g., internet-based self-help program), and then, if needed, a more intense face-to-face therapy [20].

## Supplementary Information

Below is the link to the electronic supplementary material.

Supplementary file1 (DOCX 5.97 MB)

## Data Availability

The data that support the findings of this project are openly available in OSF at https://osf.io/wq4gj, 10.17605/OSF.IO/WQ4GJ.

## References

[1] Fukuda K, Straus SE, Hickie I, Sharpe M, Dobbins JG, Komaroff A. The chronic fatigue syndrome: a comprehensive approach to its definition and study. International Chronic Fatigue Syndrome Study Group. Ann Intern Med. 1994;121:953–9. 10.7326/0003-4819-121-12-199412150-00009. 10.7326/0003-4819-121-12-199412150-00009 7978722

[2] Lim E-J, Son C-G. Review of case definitions for myalgic encephalomyelitis/chronic fatigue syndrome (ME/CFS). J Transl Med. 2020;18:289. 10.1186/s12967-020-02455-0. 32727489 10.1186/s12967-020-02455-0 PMC7391812

[3] Kleinstäuber M, Schröder A, Daehler S, et al. Aetiological understanding of fibromyalgia, irritable bowel syndrome, chronic fatigue syndrome and classificatory analogues: A systematic umbrella review. Clin Psychol Eur. 2023. 10.32872/cpe.11179. 38356902 10.32872/cpe.11179 PMC10863637

[4] Harvey SB, Wessely S. Chronic fatigue syndrome: identifying zebras amongst the horses. BMC Med. 2009;7:58. 10.1186/1741-7015-7-58. 19818158 10.1186/1741-7015-7-58 PMC2766380

[5] Wiborg JF, Knoop H, Frank LE, Bleijenberg G. Towards an evidence-based treatment model for cognitive behavioral interventions focusing on chronic fatigue syndrome. J Psychosom Res. 2012;72:399–404. 10.1016/j.jpsychores.2012.01.018. 22469284 10.1016/j.jpsychores.2012.01.018

[6] Wessely S, David A, Butler S, Chalder T. Management of chronic (post-viral) fatigue syndrome. J R Coll Gen Pract. 1989;39:26–9. 2553945 PMC1711569

[7] Wiborg JF, Knoop H, Prins JB, Bleijenberg G. Does a decrease in avoidance behavior and focusing on fatigue mediate the effect of cognitive behavior therapy for chronic fatigue syndrome? J Psychosom Res. 2011;70:306–10. 10.1016/j.jpsychores.2010.12.011. 21414449 10.1016/j.jpsychores.2010.12.011

[8] Price JR, Mitchell E, Tidy E, Hunot V. Cognitive behaviour therapy for chronic fatigue syndrome in adults. Cochrane Database Syst Rev. 2008:CD001027. 10.1002/14651858.CD001027.pub2. 10.1002/14651858.CD001027.pub2 PMC7028002 18646067

[9] Janse A, Bleijenberg G, Knoop H. Prediction of long-term outcome after cognitive behavioral therapy for chronic fatigue syndrome. J Psychosom Res. 2019;121:93–9. 10.1016/j.jpsychores.2019.03.017. 31006534 10.1016/j.jpsychores.2019.03.017

[10] Martin A, Härter M, Henningsen P, Hiller W, Kröner-Herwig B, Rief W. Evidenzbasierte Leitlinie zur Psychotherapie somatoformer Störungen und assoziierter Syndrome [Evidence-based guideline for psychotherapy for somatoform disorders and associated syndromes]. Göttingen: Hogrefe; 2013.

[11] Malouff JM, Thorsteinsson EB, Rooke SE, Bhullar N, Schutte NS. Efficacy of cognitive behavioral therapy for chronic fatigue syndrome: a meta-analysis. Clin Psychol Rev. 2008;28:736–45. 10.1016/j.cpr.2007.10.004. 18060672 10.1016/j.cpr.2007.10.004

[12] Castell BD, Kazantzis N, Moss-Morris RE. Cognitive behavioral therapy and graded exercise for chronic fatigue syndrome: A meta-analysis. Clin Psychol (New York). 2011;18:311–24. 10.1111/j.1468-2850.2011.01262.x.

[13] Turnbull N, Shaw EJ, Baker R, et al. Chronic fatigue syndrome/myalgic encephalomyelitis (or encephalopathy): diagnosis and management of chronic fatigue syndrome/myalgic encephalomyelitis (or encephalopathy) in adults and children. London: Royal College of General Practitioners; 2007. p. 2007. 21563329

[14] National Institute for Health and Care Excellence. Myalgic encephalomyelitis (or encephalopathy)/chronic fatigue syndrome: diagnosis and management. https://www.nice.org.uk/guidance/ng206. Accessed 23 Jan 2023. 35438859

[15] Busse JW, Vandvik PO, Akl EA, et al. Re: Updated NICE guidance on chronic fatigue syndrome. [Rapid response to Turner-Stokes L, et al.]. BMJ. 2020. 10.1136/bmj.m4774 33328173

[16] Ingman T, Smakowski A, Goldsmith K, Chalder T. A systematic literature review of randomized controlled trials evaluating prognosis following treatment for adults with chronic fatigue syndrome. Psychol Med. 2022;52:2917–29. 10.1017/S0033291722002471. 36059125 10.1017/S0033291722002471 PMC9693680

[17] Swift JK, Greenberg RP, Tompkins KA, Parkin SR. Treatment refusal and premature termination in psychotherapy, pharmacotherapy, and their combination: A meta-analysis of head-to-head comparisons. Psychotherapy (Chic). 2017;54:47–57. 10.1037/pst0000104. 28263651 10.1037/pst0000104

[18] Eysenbach G. The law of attrition. J Med Internet Res. 2005;7:e11. 10.2196/jmir.7.1.e11. 15829473 10.2196/jmir.7.1.e11 PMC1550631

[19] Deale A, Chalder T, Wessely S. Illness beliefs and treatment outcome in chronic fatigue syndrome. J Psychosom Res. 1998;45:77–83. 10.1016/s0022-3999(98)00021-x. 9720857 10.1016/s0022-3999(98)00021-x

[20] Tummers M, Knoop H, Bleijenberg G. Effectiveness of stepped care for chronic fatigue syndrome: a randomized noninferiority trial. J Consult Clin Psychol. 2010;78:724–31. 10.1037/a0020052. 20873907 10.1037/a0020052

[21] Page MJ, McKenzie JE, Bossuyt PM, et al. The PRISMA 2020 statement: an updated guideline for reporting systematic reviews. BMJ. 2020;2021:n71. 10.1136/bmj.n71. 10.1136/bmj.n71 PMC8005924 33782057

[22] Sharpe MC, Archard LC, Banatvala JE, et al. A report - chronic fatigue syndrome: guidelines for research. J R Soc Med. 1991;84:118–21. 1999813 10.1177/014107689108400224 PMC1293107

[23] Wissenschaftlicher Beirat Psychotherapie. Methodenpapier des Wissenschaftlichen Beirats Psychotherapie: Verfahrensregeln zur Beurteilung der wissenschaftlichen Anerkennung von Methoden und Verfahren der Psychotherapie [Methods Paper of the Scientific Advisory Board for Psychotherapy: Rules of Procedure for the Assessment of the Scientific Recognition of Methods and Procedures of Psychotherapy]. https://www.wbpsychotherapie.de/fileadmin/user_upload/downloads/pdf-Ordner/WBP/Methodenpapier.pdf. Accessed 22 Dec 2022.

[24] Sterne JAC, Savović J, Page MJ, et al. RoB 2: a revised tool for assessing risk of bias in randomised trials. BMJ. 2019;366:l4898. 10.1136/bmj.l4898. 31462531 10.1136/bmj.l4898

[25] Chowdhury S, Burton C. Associations of treatment effects between follow-up times and between outcome domains in interventions for somatoform disorders: Review of three Cochrane reviews. J Psychosom Res. 2017;98:10–8. 10.1016/j.jpsychores.2017.04.013. 28554364 10.1016/j.jpsychores.2017.04.013

[26] Cuijpers P, Noma H, Karyotaki E, Vinkers CH, Cipriani A, Furukawa TA. A network meta-analysis of the effects of psychotherapies, pharmacotherapies and their combination in the treatment of adult depression. World Psychiatry. 2020;19:92–107. 10.1002/wps.20701. 31922679 10.1002/wps.20701 PMC6953550

[27] Tehrani E, Krussel J, Borg L, Munk-Jørgensen P. Dropping out of psychiatric treatment: a prospective study of a first-admission cohort. Acta Psychiatr Scand. 1996;94:266–71. 10.1111/j.1600-0447.1996.tb09859.x. 8911562 10.1111/j.1600-0447.1996.tb09859.x

[28] Fernandez E, Salem D, Swift JK, Ramtahal N. Meta-analysis of dropout from cognitive behavioral therapy: Magnitude, timing, and moderators. J Consult Clin Psychol. 2015;83:1108–22. 10.1037/ccp0000044. 26302248 10.1037/ccp0000044

[29] *Janse A, Worm-Smeitink M, Bleijenberg G, Donders R, Knoop H. Efficacy of web-based cognitive-behavioural therapy for chronic fatigue syndrome: randomised controlled trial. Br J Psychiatry. 2018;212:112–8. 10.1192/bjp.2017.22. 10.1192/bjp.2017.22 29436329

[30] *Milrad SF, Hall DL, Jutagir DR, et al. Relationship satisfaction, communication self-efficacy, and chronic fatigue syndrome-related fatigue. Soc Sci Med. 2019;237:112392. 10.1016/j.socscimed.2019.112392. 10.1016/j.socscimed.2019.112392 PMC8088125 31377502

[31] *O'Dowd H, Gladwell P, Rogers CA, Hollinghurst S, Gregory A. Cognitive behavioural therapy in chronic fatigue syndrome: a randomised controlled trial of an outpatient group programme. Health Technol Assess. 2006;10:iii–iv, ix–x, 1–121. 10.3310/hta10370. 10.3310/hta10370 17014748

[32] *Rimes KA, Wingrove J. Mindfulness-based cognitive therapy for people with chronic fatigue syndrome still experiencing excessive fatigue after cognitive behaviour therapy: a pilot randomized study. Clin Psychol Psychother. 2013;20:107–17. 10.1002/cpp.793. 10.1002/cpp.793 21983916

[33] *Wiborg JF, van Bussel J, van Dijk A, Bleijenberg G, Knoop H. Randomised controlled trial of cognitive behaviour therapy delivered in groups of patients with chronic fatigue syndrome. PPS. 2015;84:368–76. 10.1159/000438867. 10.1159/000438867 26402868

[34] *Deale A, Chalder T, Marks I, Wessely S. Cognitive behavior therapy for chronic fatigue syndrome: a randomized controlled trial. Am J Psychiatry. 1997;154:408–14. 10.1176/ajp.154.3.408. 10.1176/ajp.154.3.408 9054791

[35] *Jason LA, Torres-Harding S, Friedberg F, Corradi K, Njoku MG, Donalek J, et al. Non-pharmacologic Interventions for CFS: A Randomized Trial. J Clin Psychol Med Settings. 2007;14:275–96. 10.1007/s10880-007-9090-7.

[36] *Friedberg F, Napoli A, Coronel J, et al. Chronic fatigue self-management in primary care: a randomized trial. Psychosom Med. 2013;75:650–7. 10.1097/PSY.0b013e31829dbed4. 10.1097/PSY.0b013e31829dbed4 PMC3785003 23922399

[37] *Knoop H, van der Meer JWM, Bleijenberg G. Guided self-instructions for people with chronic fatigue syndrome: randomised controlled trial. Br J Psychiatry. 2008;193:340–1. 10.1192/bjp.bp.108.051292. 10.1192/bjp.bp.108.051292 18827302

[38] Worm-Smeitink M, Janse A, van Dam A, et al. Internet-Based Cognitive Behavioral Therapy in Stepped Care for Chronic Fatigue Syndrome: Randomized Noninferiority Trial. J Med Internet Res. 2019;21:e11276. 10.2196/11276. 30869642 10.2196/11276 PMC6437617

[39] Núñez M, Fernández-Solà J, Nuñez E, Fernández-Huerta J-M, Godás-Sieso T, Gomez-Gil E. Health-related quality of life in patients with chronic fatigue syndrome: group cognitive behavioural therapy and graded exercise versus usual treatment. A randomised controlled trial with 1 year of follow-up. Clin Rheumatol. 2011;30:381–9. 10.1007/s10067-010-1677-y. 10.1007/s10067-010-1677-y 21234629

[40] *Gotaas ME, Stiles TC, Bjørngaard JH, Borchgrevink PC, Fors EA. Cognitive Behavioral Therapy Improves Physical Function and Fatigue in Mild and Moderate Chronic Fatigue Syndrome: A Consecutive Randomized Controlled Trial of Standard and Short Interventions. Front Psychiatry. 2021;12: 580924. 10.3389/fpsyt.2021.580924. 10.3389/fpsyt.2021.580924 PMC8071989 33912079

[41] *Prins JB, Bleijenberg G, Bazelmans E, et al. Cognitive behaviour therapy for chronic fatigue syndrome: a multicentre randomised controlled trial. Lancet. 2001;357:841–7. 10.1016/S0140-6736(00)04198-2. 10.1016/S0140-6736(00)04198-2 11265953

[42] *White PD, Goldsmith KA, Johnson AL, et al. Comparison of adaptive pacing therapy, cognitive behaviour therapy, graded exercise therapy, and specialist medical care for chronic fatigue syndrome (PACE): a randomised trial. Lancet. 2011;377:823–36. 10.1016/S0140-6736(11)60096-2. 10.1016/S0140-6736(11)60096-2 PMC3065633 21334061

[43] *O’Dowd H, Beasant L, Ingram J, et al. The feasibility and acceptability of an early intervention in primary care to prevent chronic fatigue syndrome (CFS) in adults: randomised controlled trial. Pilot Feasibility Stud. 2020;6:65. 10.1186/s40814-020-00595-0. 10.1186/s40814-020-00595-0 PMC7216523 32426159

[44] *Sharpe M, Hawton K, Simkin S, et al. Cognitive behaviour therapy for the chronic fatigue syndrome: a randomized controlled trial. BMJ. 1996;312:22–6. 10.1136/bmj.312.7022.22. 10.1136/bmj.312.7022.22 PMC2349693 8555852

[45] Leone SS, Huibers MJH, Kant I, et al. Long-term efficacy of cognitive-behavioral therapy by general practitioners for fatigue: a 4-year follow-up study. J Psychosom Res. 2006;61:601–7. 10.1016/j.jpsychores.2006.04.010. 17084137 10.1016/j.jpsychores.2006.04.010

[46] *Huibers MJH, Beurskens AJHM, van Schayck CP, et al. Efficacy of cognitive-behavioural therapy by general practitioners for unexplained fatigue among employees: Randomised controlled trial. Br J Psychiatry. 2004;184:240–6. 10.1192/bjp.184.3.240. 10.1192/bjp.184.3.240 14990522

[47] Sharpe M, Goldsmith KA, Johnson AL, Chalder T, Walker J, White PD. Rehabilitative treatments for chronic fatigue syndrome: long-term follow-up from the PACE trial. Lancet Psychiatry. 2015;2:1067–74. 10.1016/S2215-0366(15)00317-X. 26521770 10.1016/S2215-0366(15)00317-X

[48] Maas genannt Bermpohl F, Hülsmann L, Martin A. Efficacy of mindfulness- and acceptance-based cognitive-behavioral therapies for bodily distress in adults: a meta-analysis. Front Psychiatry. 2023. 10.3389/fpsyt.2023.1160908. 10.3389/fpsyt.2023.1160908 PMC10157071 37151971

[49] Kuut TA, Buffart LM, Braamse AMJ, et al. Does the effect of cognitive behavior therapy for chronic fatigue syndrome (ME/CFS) vary by patient characteristics? A systematic review and individual patient data meta-analysis. Psychol Med. 2023;1–10. 10.1017/S0033291723003148. 10.1017/S0033291723003148 37927223

[50] Heins MJ, Knoop H, Prins JB, Stulemeijer M, van der Meer JWM, Bleijenberg G. Possible detrimental effects of cognitive behaviour therapy for chronic fatigue syndrome. PPS. 2010;79:249–56. 10.1159/000315130. 10.1159/000315130 20502065

[51] Melville KM, Casey LM, Kavanagh DJ. Dropout from Internet-based treatment for psychological disorders. Br J Clin Psychol. 2010;49:455–71. 10.1348/014466509X472138. 19799804 10.1348/014466509X472138

[52] Swift JK, Greenberg RP. Premature discontinuation in adult psychotherapy: a meta-analysis. J Consult Clin Psychol. 2012;80:547–59. 10.1037/a0028226. 22506792 10.1037/a0028226

[53] Karlsson P, Bergmark A. Compared with what? An analysis of control-group types in Cochrane and Campbell reviews of psychosocial treatment efficacy with substance use disorders. Addiction. 2015;110:420–8. 10.1111/add.12799. 25393504 10.1111/add.12799 PMC4374442

[54] Gold SM, Enck P, Hasselmann H, et al. Control conditions for randomised trials of behavioural interventions in psychiatry: a decision framework. Lancet Psychiatry. 2017;4:725–32. 10.1016/S2215-0366(17)30153-0. 28396067 10.1016/S2215-0366(17)30153-0

[55] Kim D-Y, Lee J-S, Son C-G. Systematic Review of Primary Outcome Measurements for Chronic Fatigue Syndrome/Myalgic Encephalomyelitis (CFS/ME) in Randomized Controlled Trials. J Clin Med. 2020. 10.3390/jcm9113463. 33126460 10.3390/jcm9113463 PMC7692998

[56] Vink M, Vink-Niese A. The Updated NICE Guidance Exposed the Serious Flaws in CBT and Graded Exercise Therapy Trials for ME/CFS. Healthcare (Basel). 2022. 10.3390/healthcare10050898. 35628033 10.3390/healthcare10050898 PMC9141828

[57] Rief W, Burton C, Frostholm L, et al. Core Outcome Domains for Clinical Trials on Somatic Symptom Disorder, Bodily Distress Disorder, and Functional Somatic Syndromes: European Network on Somatic Symptom Disorders Recommendations. Psychosom Med. 2017;79:1008–15. 10.1097/PSY.0000000000000502. 28691994 10.1097/PSY.0000000000000502

[58] Kitselaar WM, van der Vaart R, Perschl J, Numans ME, Evers AWM. Predictors of Persistent Somatic Symptoms in the General Population: A Systematic Review of Cohort Studies. Psychosom Med. 2023;85:71–8. 10.1097/PSY.0000000000001145. 36315905 10.1097/PSY.0000000000001145

[59] Moustgaard H, Clayton GL, Jones HE, et al. Impact of blinding on estimated treatment effects in randomised clinical trials: meta-epidemiological study. BMJ. 2020;368:l6802. 10.1136/bmj.l6802. 10.1136/bmj.l6802 PMC7190062 31964641

[60] Baumeister H, Seifferth H, Lin J, Nowoczin L, Lüking M, Ebert D. Impact of an Acceptance Facilitating Intervention on Patients’ Acceptance of Internet-based Pain Interventions: A Randomized Controlled Trial. Clin J Pain. 2015;31:528–35. 10.1097/AJP.0000000000000118. 24866854 10.1097/AJP.0000000000000118

