# Supplementary material for: Efficacy and Acceptance of Cognitive Behavioral Therapy in Adults with Chronic Fatigue Syndrome: A Meta-analysis
Source: Int J Behav Med. 2024 Jan 16;31(6):895–910. doi: 10.1007/s12529-023-10254-2 (PMC11588766; doi:10.1007/s12529-023-10254-2)
Supplement: Supplementary file 1 — Supplementary file1 (DOCX 5.97 MB) [file 12529_2023_10254_MOESM1_ESM.docx]

***Supplement - Efficacy and Acceptance of Cognitive Behavioral Therapy in Adults with Chronic Fatigue Syndrome: A Meta-analysis***

Frederic Maas genannt Bermpohl, Ann-Cathrin Kucharczyk-Bodenburg, Alexandra Martin

**Table of contents – Supplement**

[***Supplement - Efficacy and Acceptance of Cognitive Behavioral Therapy in Adults with Chronic Fatigue Syndrome: A Meta-analysis*** 1](#_Toc151292374)

[**1.** **Eligible Diagnostic Criteria** 2](#_Toc151292375)

[**2.** **Search Strategy** 2](#_Toc151292376)

[**3.** **Risk of Bias Assessment** 3](#_Toc151292377)

[**4.** **R Packages** 6](#_Toc151292378)

[**5.** **Study Characteristics** 7](#_Toc151292379)

[**6.** **Outcome Measures** 19](#_Toc151292380)

[**7.** **Results Moderator Analyses – Meta-regressions** 20](#_Toc151292381)

[**8.** **Results Sensitivity Analyses** 39](#_Toc151292382)

[**9.** **Publication Bias** 62](#_Toc151292383)

[**10.** **P-curve Analyses** 67](#_Toc151292384)

[**References** 68](#_Toc151292385)

# **Eligible Diagnostic Criteria**

The following criteria were considered eligible for inclusion:

- Holmes Criteria [1]
- Oxford criteria [2]
- Fukuda definition/Centers for Disease Control and Prevention (CDC) definition [3]
- Canadian consensus criteria [4]
- international consensus criteria [5]
- NICE guidelines [6]
- ICD-10 [7]: Neurasthenia (F48.0), unspecified Chronic fatigue (R53.82), ME/postviral fatigue syndrome (G.93.3)
- Systemic Exertion Intolerance Disease [8, 9]

# **Search Strategy**

Exemplary search algorithm for PubMed:

("fatigue syndrome, chronic" [MeSH] OR "chronic fatigue syndrome" [All Fields] OR "fatigue syndrome*" [All Fields] OR CFS [All Fields] OR "ME/CFS" [All Fields] OR "CFS/ME" [All Fields] OR "psychogenic fatigue" [All Fields] OR "myalgic encephalomyelit*" [All Fields] OR "myalgic encephalopath*" [All Fields] OR "encephalopath*" [All Fields] OR "benign myalgic encephalomyelitis" [All Fields] OR "postviral fatigue syndrome*" [All Fields] OR "unspecified chronic fatigue" [All Fields] OR "post-infectious fatigue syndrome*" [All Fields] OR "chronic fatigue immune dysfunction syndrome*" [All Fields] OR "neurasthenia" [All Fields] OR "psychasthenia" [All Fields] OR "systemic exertion intolerance*" [All Fields] OR "post-exertional malaise"[All Fields]) AND (cbt [All Fields] OR "cognitive behavioral therap*" [All Fields] OR "cognitive behavioural therap*" [All Fields] OR "cognitive behavioral treatment*" [All Fields] OR "cognitive behavioural treatment*" [All Fields] OR "cognition therap*"[All Fields] OR "cognition treatment*"[All Fields] OR "cognitive therap*"[All Fields] OR "cognitive treatment*"[All Fields] OR "cognitive restructuring" [All Fields] OR (cognitive AND "perceptual change*") OR "cognitive reappraisal" [All Fields] OR "exposure therap*" [All Fields] OR "behavior modification*" OR "behaviour modification*" OR "resource activation" [All Fields] OR ("problem?solving" AND (therap* OR treatment*)) OR "behavioral activation" [All Fields] OR "behavioural activation" [All Fields] OR "behavioral activation therap*" [All Fields] OR "behavioural activation therap*" [All Fields] OR ("third wave" AND (therap* OR treatment*)) OR mindful* [All Fields] OR MBT [All Fields] OR MBCT [All Fields] OR MBSR [All Fields] OR (acceptance AND commitment) OR (dialectic* AND (behavioural OR behavioral)) OR DBT [All Fields] OR "metacognitive therap*" [All Fields] OR "metacognitive treatment*" [All Fields] OR "meta?cognitive therap*" [All Fields] OR "meta?cognitive treatment*" [All Fields] OR MCT [All Fields] OR ((Schematherap* OR "schema therap*") AND Young) OR "emotion?regulation skills training*" [All Fields] OR "emotion?regulation training*" [All Fields] OR "unified protocol*" OR "compassion focused therap*" [All Fields] OR "compassionate mind training*" [All Fields] OR "well-being therap*" [All Fields] OR "functional analytic psychotherap*" [All Fields] OR "positive psychotherap*" [All Fields] OR "integrative behavioural couples therap*" [All Fields] OR "integrative behavioural couple therap*" [All Fields] OR "mode deactivation therap*" [All Fields] OR "assertiveness training" [All Fields] OR (("compassion-focused" [All Fields] OR "compassion-focussed" [All Fields]) AND (therapy [SH] OR therapies [All Fields] OR therapy [All Fields] OR therape*[All Fields] OR therapis*[All Fields] OR Therapeutics [All Fields] OR treatment*[All Fields])) OR "solution-focused therap*"[All Fields] OR "self-control therap*"[All Fields] OR "self-control training*"[All Fields] OR "self control therap*"[All Fields] OR "self control training*"[All Fields])

# **Risk of Bias Assessment**

**Figure S1**

*Risk of bias ratings for fatigue at post-treatment*

1.
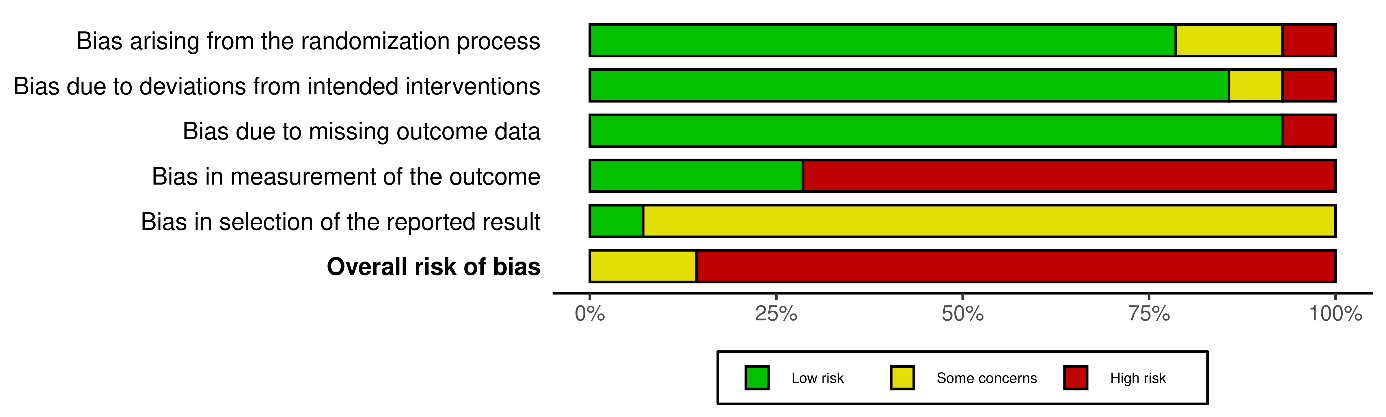

2.
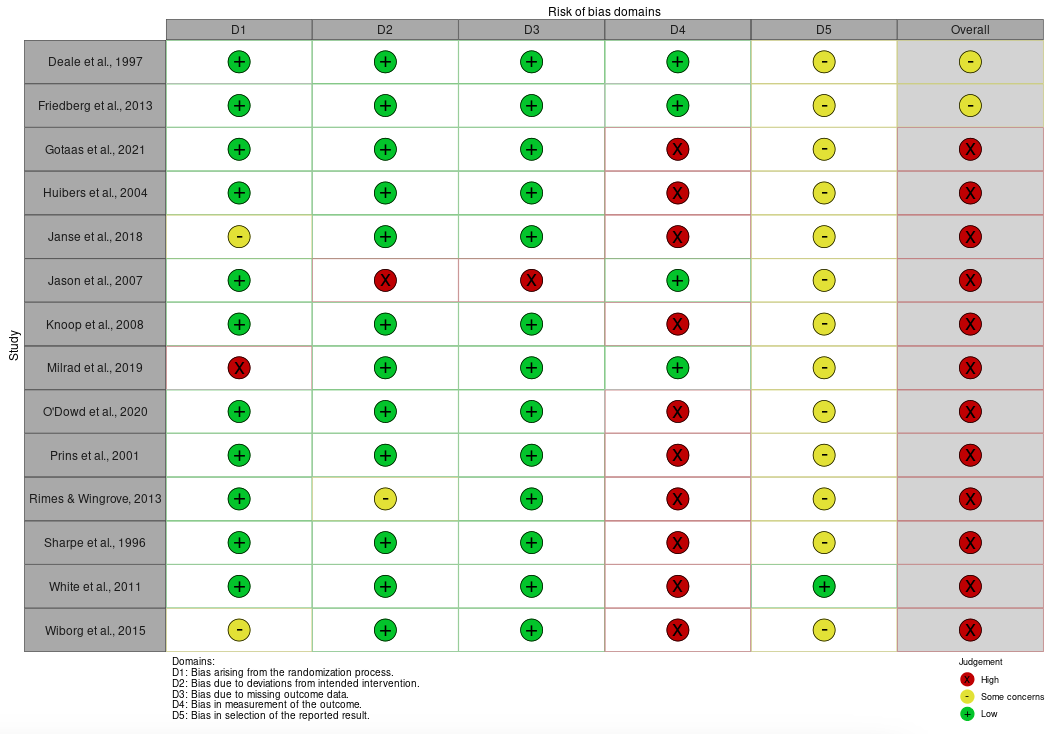


*Note.* A: summary plot; B: traffic light plot.

**Figure S2**

*Risk of bias ratings for perceived health status at post-treatment*

1.
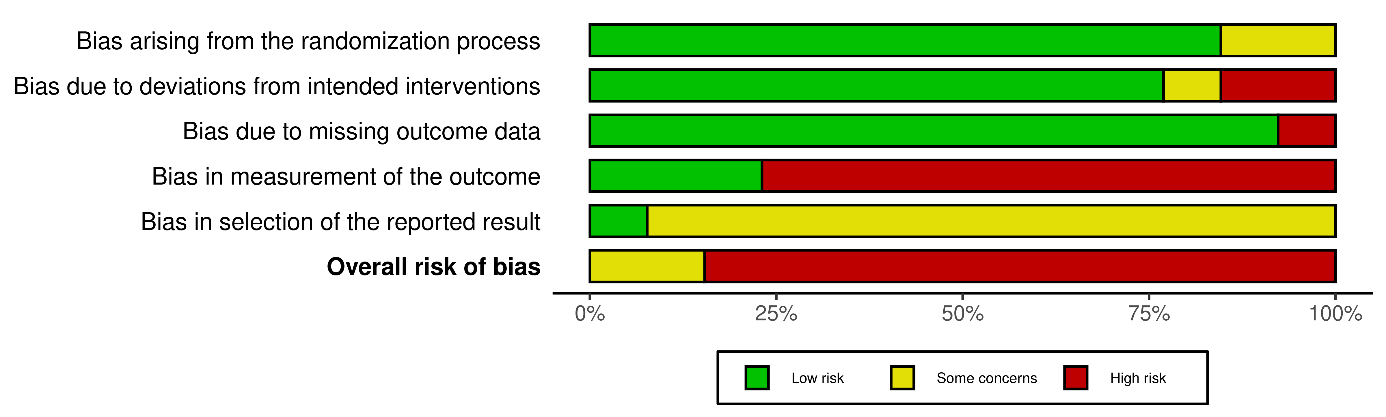

2.
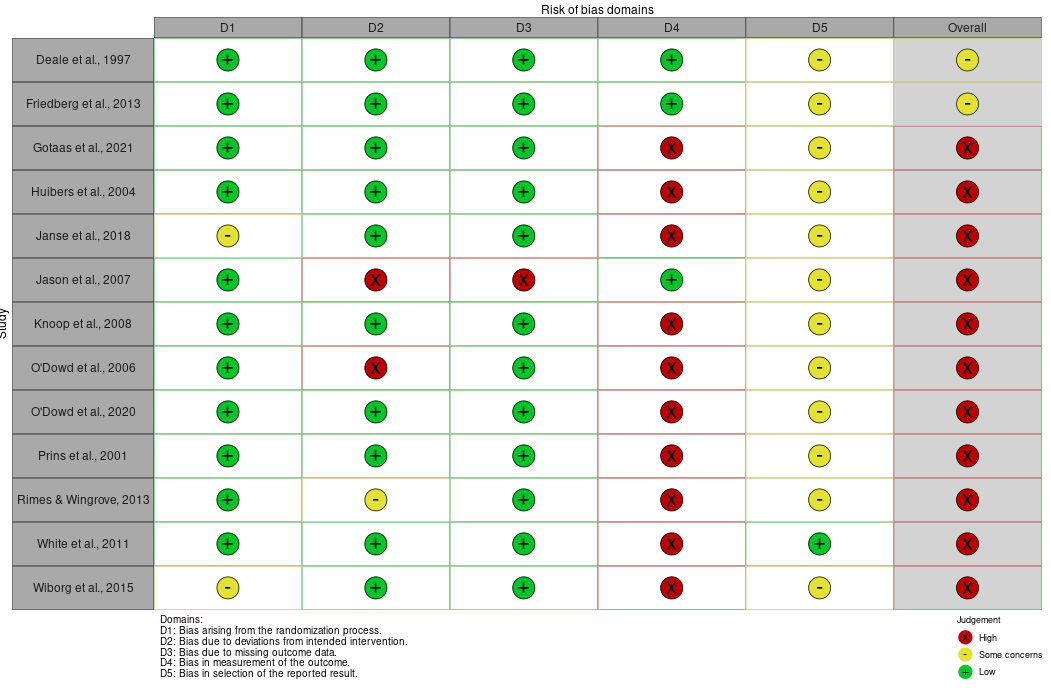


*Note.* A: summary plot; B: traffic light plot.

**Figure S3**

*Risk of bias ratings for depression at post-treatment*

1.
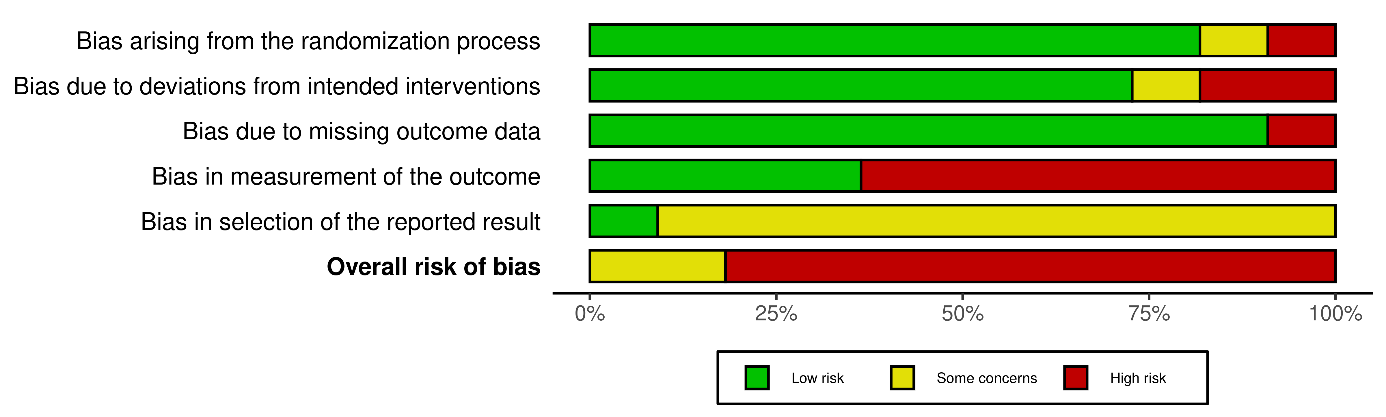

2.
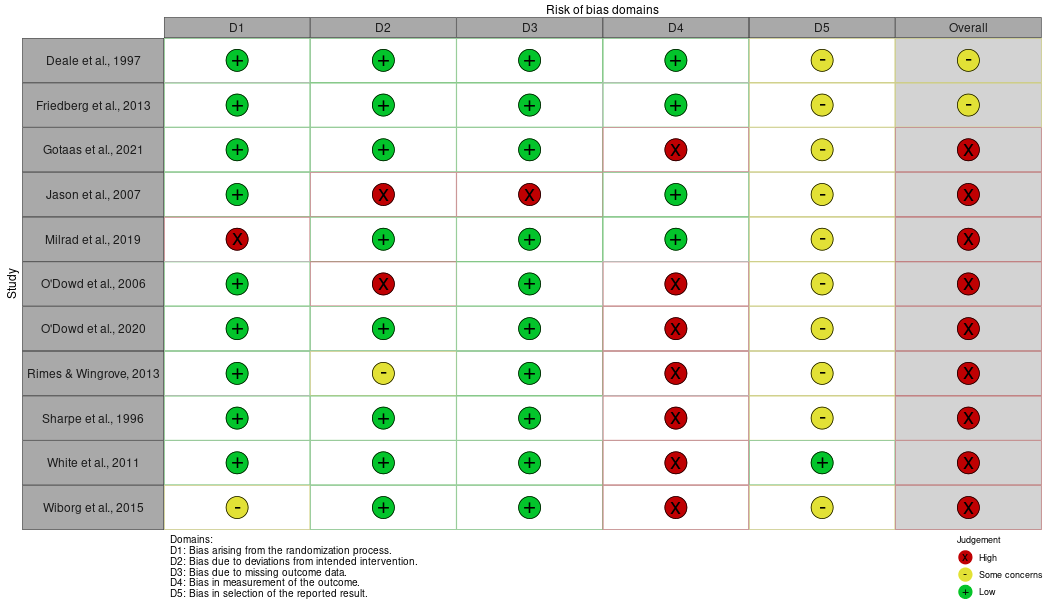


*Note.* A: summary plot; B: traffic light plot.

**Figure S4**

*Risk of bias ratings for anxiety at post-treatment*

1.
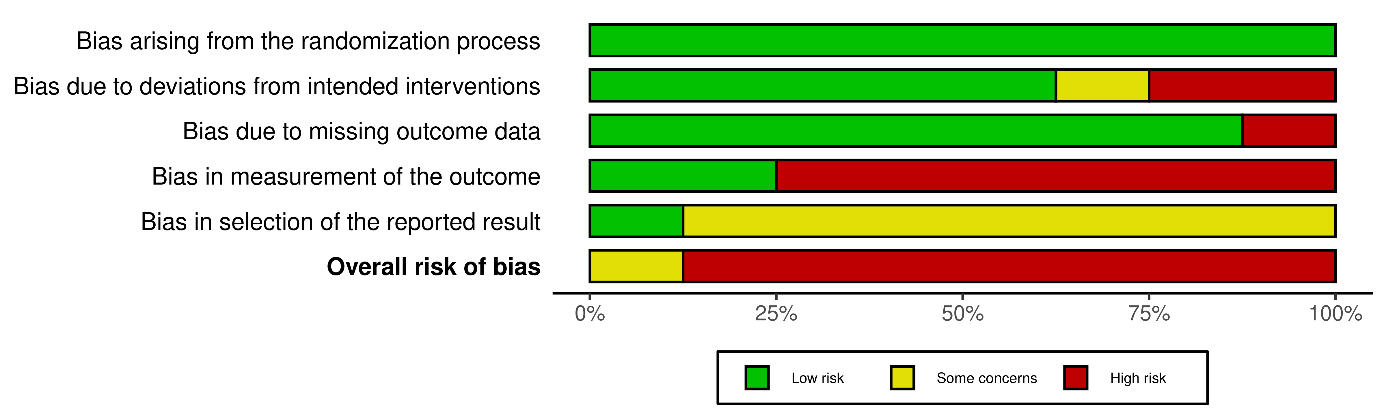

2.
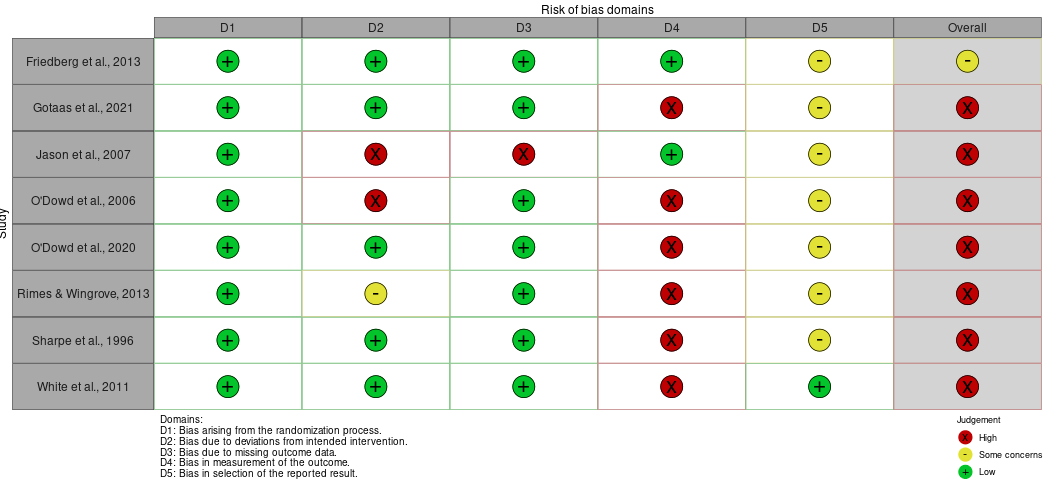


*Note.* A: summary plot; B: traffic light plot.

# **R Packages**

We used the packages {meta} [10], {metafor} [11], and {dmetar} [12] to aggregate effect sizes and compute associated analyses, and {tidyverse} [13] to visualize data in R version 4.2.1 [14]. For additional functions, we used the packages {esc} [15], and {car} [16]. Furthermore, data was imported using {readxl} [17]. To depict the risk of bias assessment, the package {robvis} [18] as well as the corresponding web application (<https://mcguinlu.shinyapps.io/robvis/>) were utilized.

# **Study Characteristics**

**Table S1**

*Study Characteristics regarding acceptance*

| Study | Con-ditions | *n* | Drop-out definition | Prop. drop-outs | Prop. treatment refusal | Reasons treatment refusal | Prop. non-completion | Reasons non-completion | Prop. average sessions completed |
| --- | --- | --- | --- | --- | --- | --- | --- | --- | --- |
| Deale et al., 1997 | CBT | 30 |  |  |  |  | 0.10  [0.02; 0.27] | Found it ineffective (*n* = 3), felt too ill to attend as an outpatient (*n* = 1), improved and wanted no further treatment (*n* = 1) |  |
|  | Psy plac | 30 |  |  |  |  |  | Too ill to continue attending  (*n* = 1), no reason (*n* = 1), found the relaxation exercises overly tiring  (*n* = 2) |  |
| Friedberg et al., 2013 | CBT | 37 |  | 0.23  [0.10; 0.42] |  |  | 0.19  [0.08; 0.35] | Too much work to do in study, didn't feel that the type of treatment was appropriate, no longer interested, “unknown” for those who did not return phone calls |  |
|  | Att plac | 38 |  |  |  |  |  |  |  |
|  |  |  |  |  |  |  |  |  |  |
| Gotaas et al., 2021 | CBT | 78 |  | 0.21 [0.12; 0.32] | 0.01  [0.00; 0.07] |  | 0.23  [0.82; 0.93] | Due to fatigue (*n* = 3), poor economy (*n* = 2), psychosocial strain (*n* = 2), long distance to treatment centre (*n* = 2), lack of motivation for the treatment (*n* = 4), change of therapist (*n* = 1), current infection (*n* = 1), need of further psychiatric evaluation (*n* = 1) |  |
|  | WL | 80 |  |  |  |  |  |  |  |
| Huibers et al., 2004 | CBT | 76 | Not completing CBT | 0.28 [0.18; 0.40] | 0.06  [0.02; 0.15] |  |  | Too busy/ work resumption (*n* = 6), not satisfied (*n* = 5), psychiatric complications (*n* = 2), unknown reason (*n* = 7) | 0.88  [0.39; 0.99] |
|  | TAU | 75 | Not completing post assessment |  |  |  |  |  |  |
| Janse et al., 2018 | CBT | 160^a^ | Opening all modules + emailing fortnightly | 0.31  [0.24; 0.39] | 0.06  [0.03; 0.11] |  | 0.88  [0.82; 0.93] |  |  |
|  | WL | 80 | Not completing post assessment |  |  |  |  |  |  |
| Jason et al., 2007 | CBT | 29 | Completing  < 4 sessions |  |  |  |  |  | 0.77  [0.48; 0.92] |
|  | Psy plac | 28 | Completing  < 4 sessions |  |  |  |  |  |  |
| Knoop et al., 2008 | CBT | 85 |  |  | 0.20  [0.12; 0.30] |  |  |  |  |
|  | WL | 86 |  |  |  |  |  |  |  |
| Milrad et al., 2019 | CBT | 75 |  | 0.05  [0.01; 0.13] |  |  |  |  | 0.93  [0.54; 0.99] |
|  | Psy plac | 75 |  |  |  |  |  |  |  |
| O’Dowd et al., 2006 | CBT | 52 |  |  |  |  |  | Moved away (*n* =2), work pressures (*n* = 1), dropped out following an argument (*n* = 1), transport problems (*n* = 1), unable to attend owing to a bereavement (*n* = 2) |  |
|  | TAU | 51 |  |  |  |  |  | Withdrew from the study (*n* = 3), on holiday (*n* = 1), unable to attend (*n* = 1) |  |
| O’Dowd et al., 2020 | CBT | 28 |  |  | 0.39  [0.22; 0.59] |  | 0.41  [0.18; 0.67] | Did not relate to the therapeutic model, disliked telephone consultations, found the concept of self-reflection challenging |  |
|  | TAU | 16 |  |  |  |  |  |  |  |
| Prins et al., 2001 | CBT | 93 | Formally withdrawing CBT | 0.28  [0.18; 0.39] | 0.11  [0.05; 0.19] |  |  |  |  |
|  | TAU | 91 | Not attending assessments |  |  |  |  |  |  |
| Rimes & Wingrove, 2013 | MBCT | 18 | “Discontinued MBCT” | 0.06  [0.00; 0.29] | 0.06  [0.00; 0.27] | Withdrew due to family illness (*n* =1) |  | Did not like group nature of MBCT (*n* = 1) | 0.81  [0.45; 0.96] |
|  | WL | 19 | Not completing post assessment |  |  |  |  |  |  |
| Sharpe et al., 1996 | CBT | 30 | No dropouts reported | 0.00  [0.00; 0.12] | 0.00  [0.00; 0.12] |  | 0.00  [0.00; 0.12] |  |  |
|  | TAU | 30 |  |  |  |  |  |  |  |
| White et al., 2011 | CBT | 161 | Completing < 10 sessions | 0.11  [0.06; 0.17] | 0.02  [0.00; 0.05] |  |  |  |  |
|  | TAU | 160 | Completing  < 3 sessions |  |  |  |  |  |  |
| Wiborg et al., 2015 | CBT | 136 | “Discontinued intervention” | 0.15  [0.09; 0.22] | 0.12  [0.07; 0.18] |  |  |  |  |
|  | WL | 68 | Not completing post assessment |  |  |  |  |  |  |

*Note.* Att plac = attention placebo; Average proportions of sessions completed = (average number of sessions completed) / (total number of sessions); CBT = cognitive behavioural therapy; Drop-Outs = primary authors’ definition of drop-out: (𝑛 participants dropping out according to authors’ definition) / (𝑛 participants starting intervention); *n* = number of participants allocated to each group; Non-completion = prop. of participants who started treatment and completed at least one session: (𝑛 participants not completing all sessions) / (𝑛 participants starting intervention); Psy plac = psychological placebo; TAU = treatment as usual; WL = wait-list.

^a^ two intervention groups (ICBT with feedback on demand & ICBT with protocol-driven feedback) were merged for conducting the analyses.

**Figure S5**


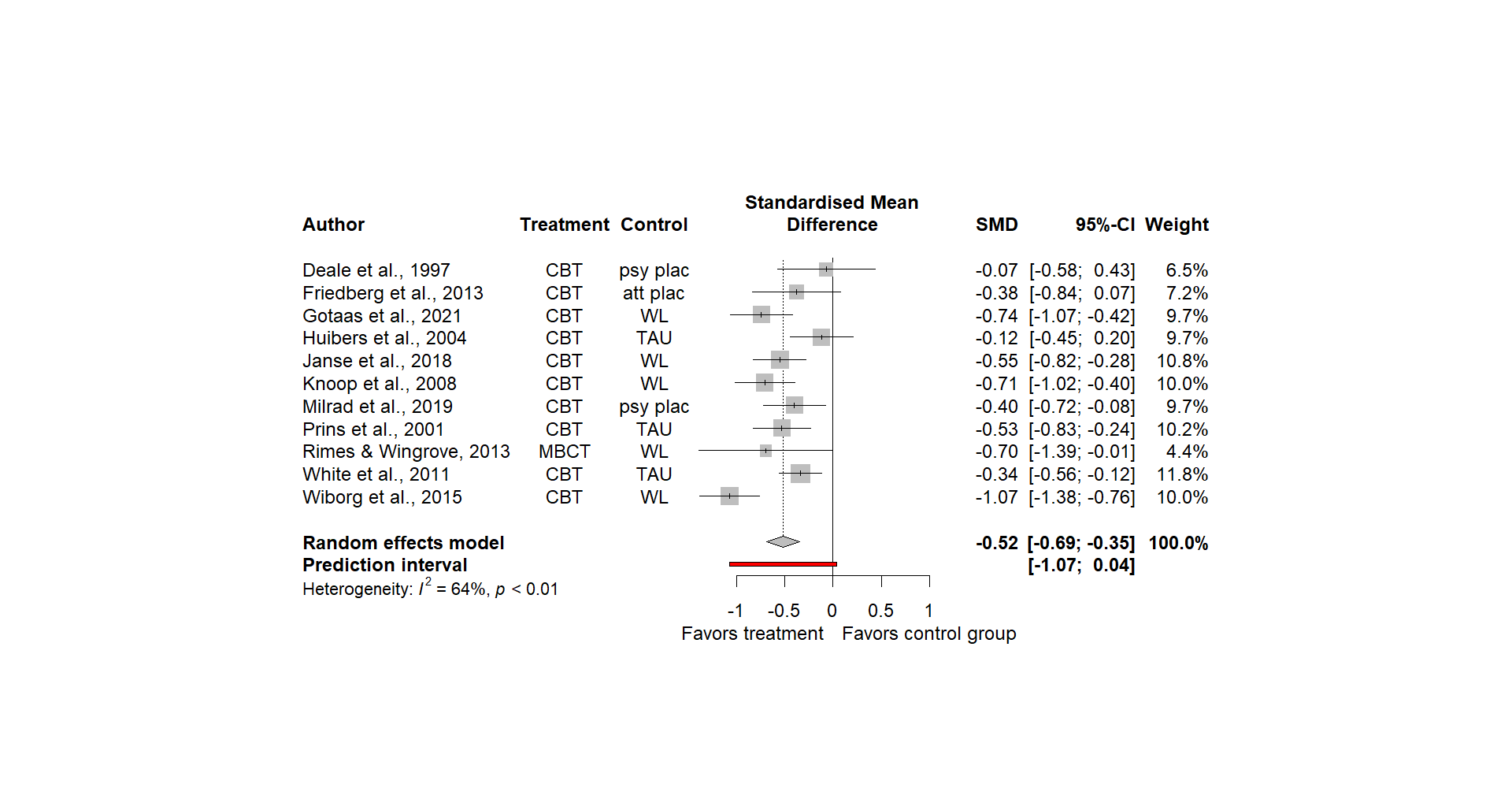
*Forest plots – Post-treatment*

(A)


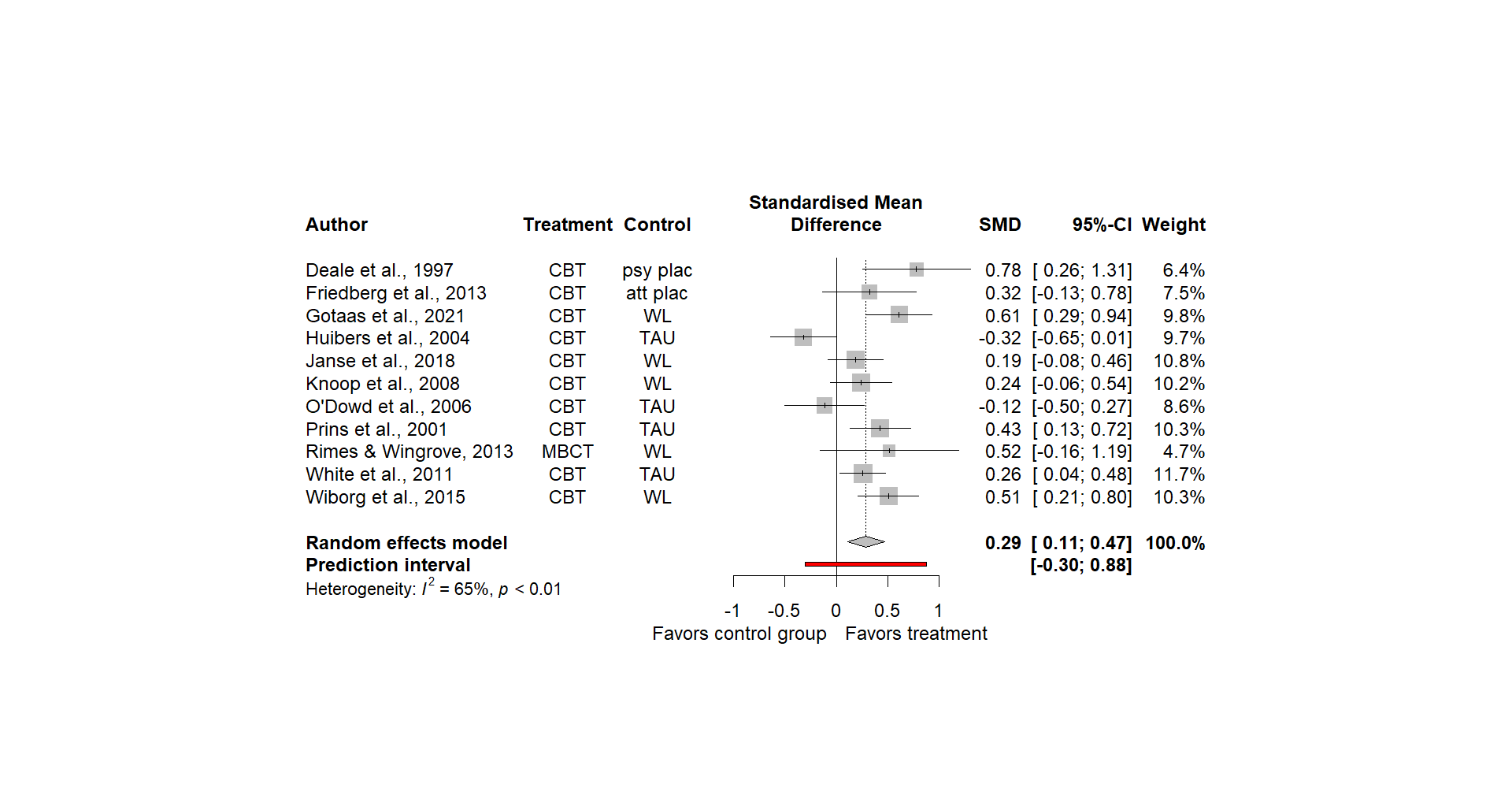
(B)


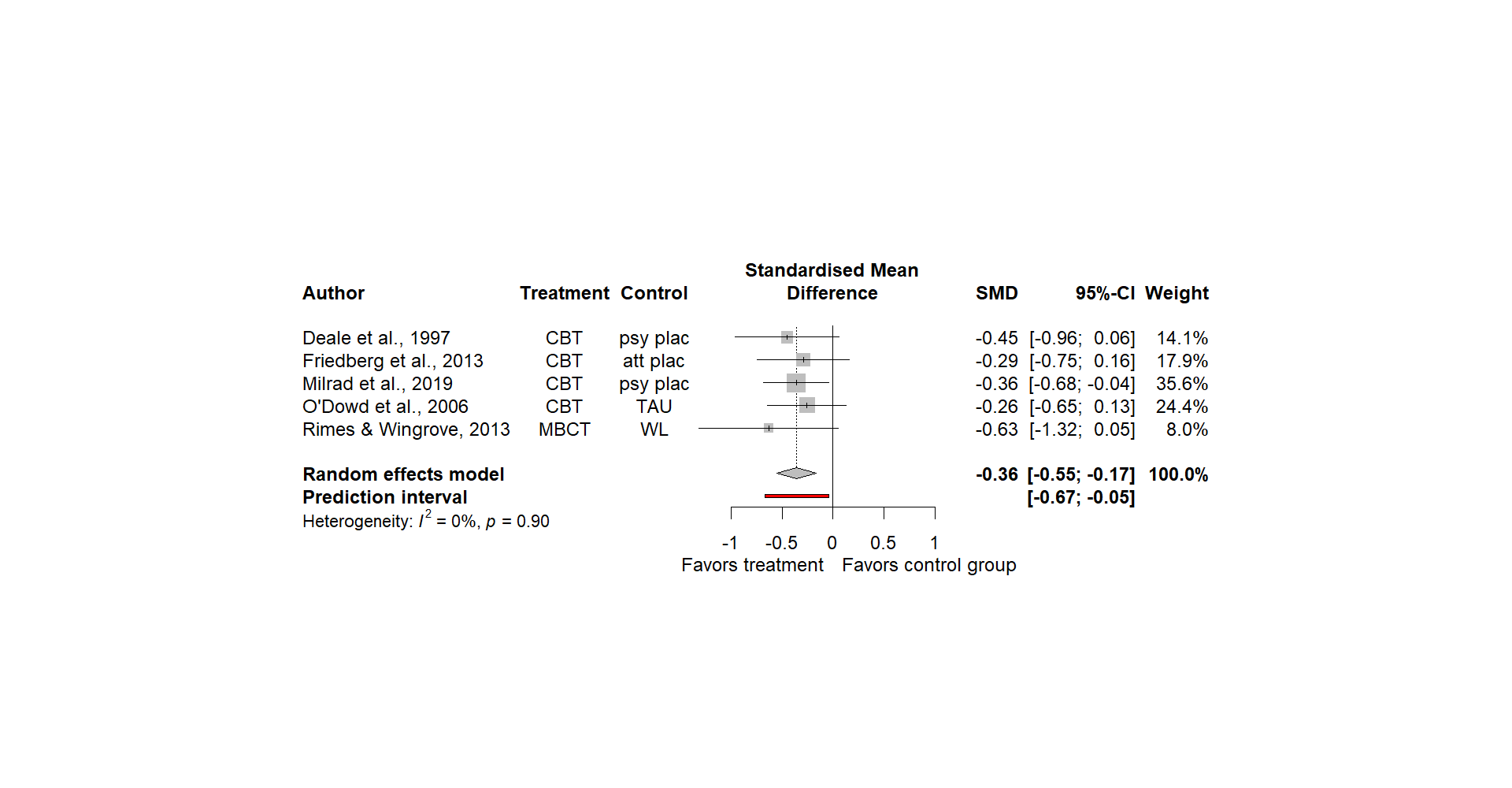


(C)


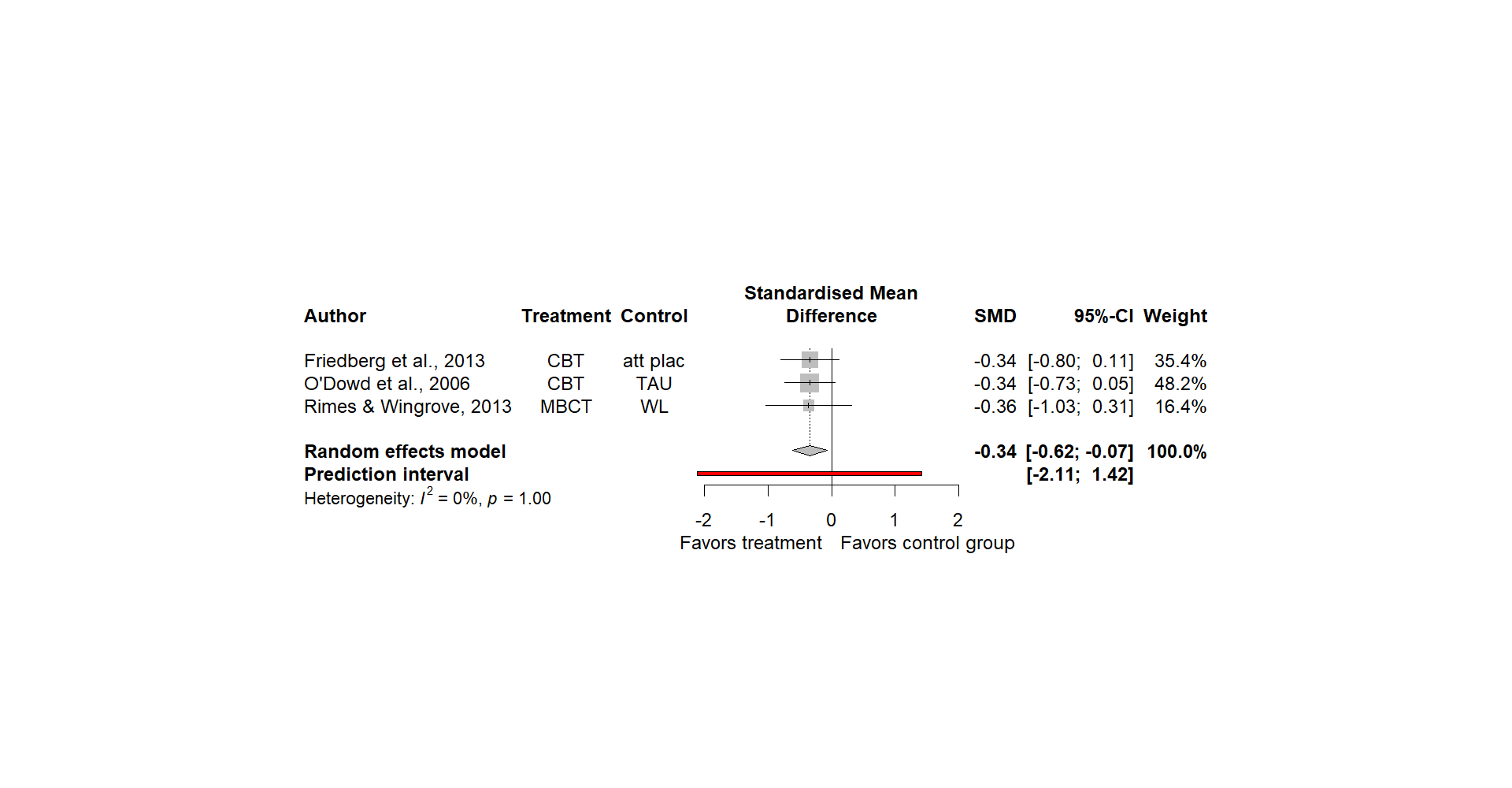


(D)

*Note.* A: fatigue at post-treatment; B: perceived health status at post-treatment; C: depression at post-treatment; D: anxiety at post-treatment. Note: For perceived health status positive effects indicate efficacy of the treatments, for all other outcomes negative effects indicate efficacy of the treatments

**Figure S6**

*Forest plots – Long-term follow-up*


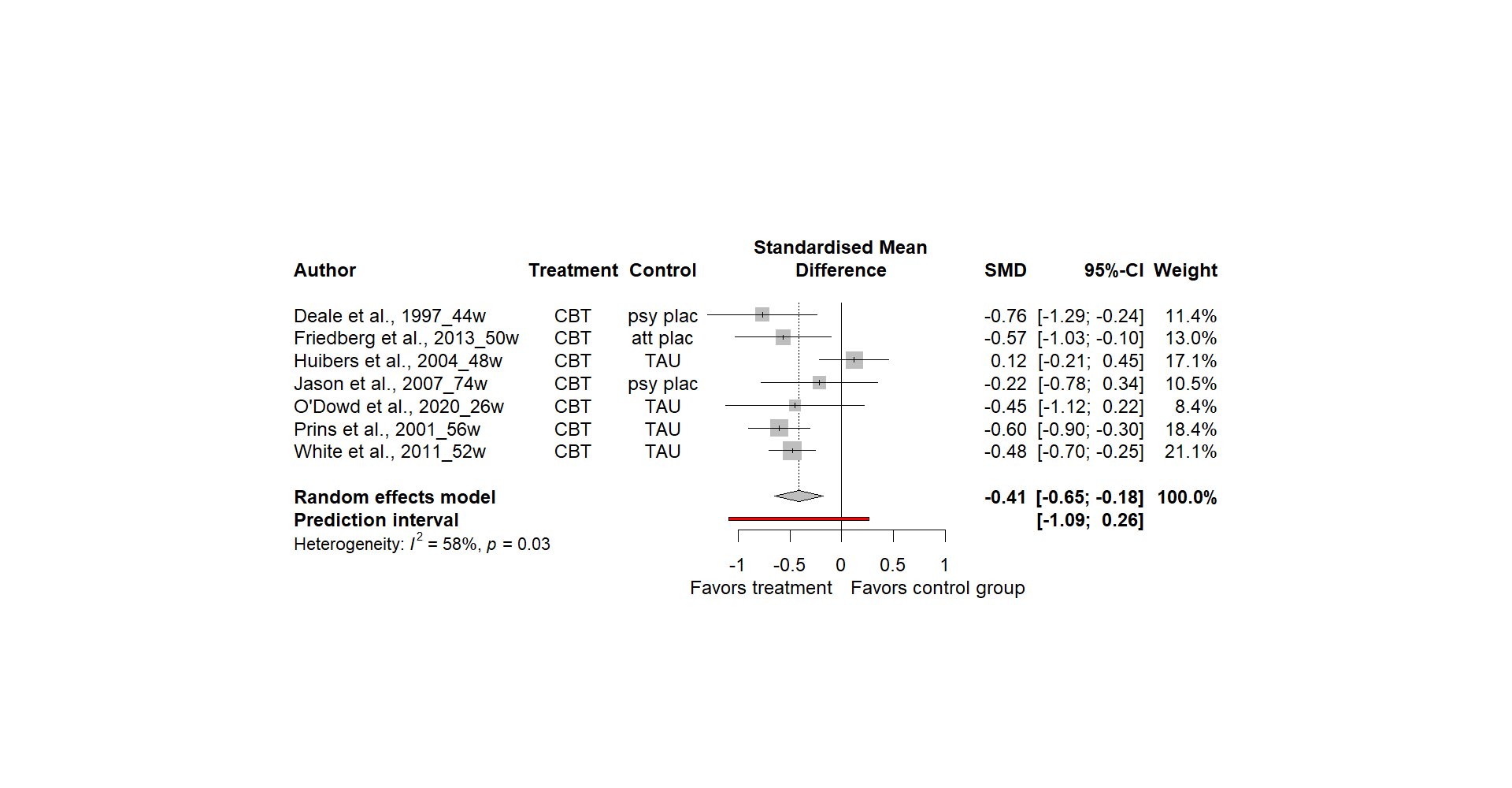


(A)


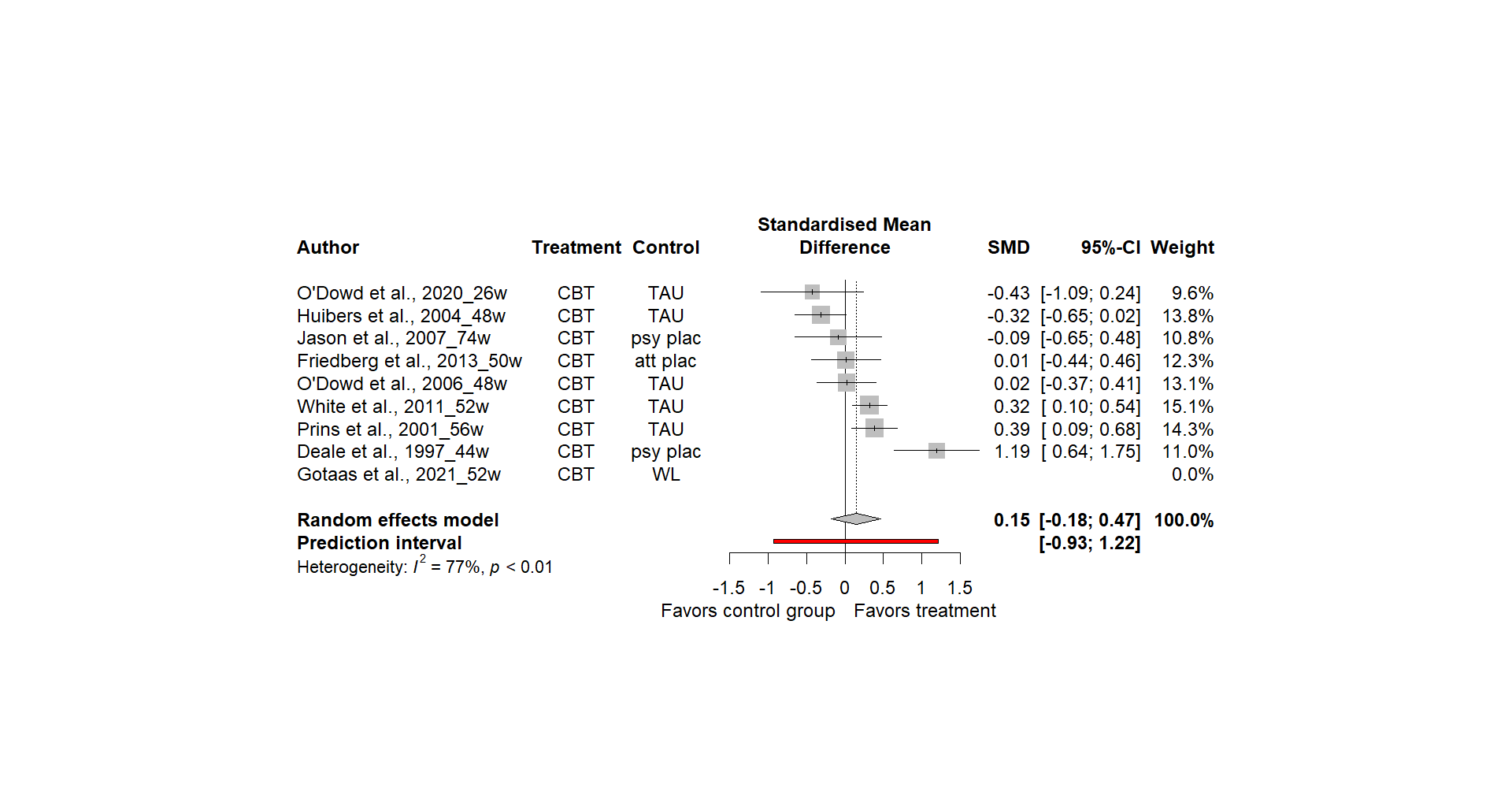


(B)


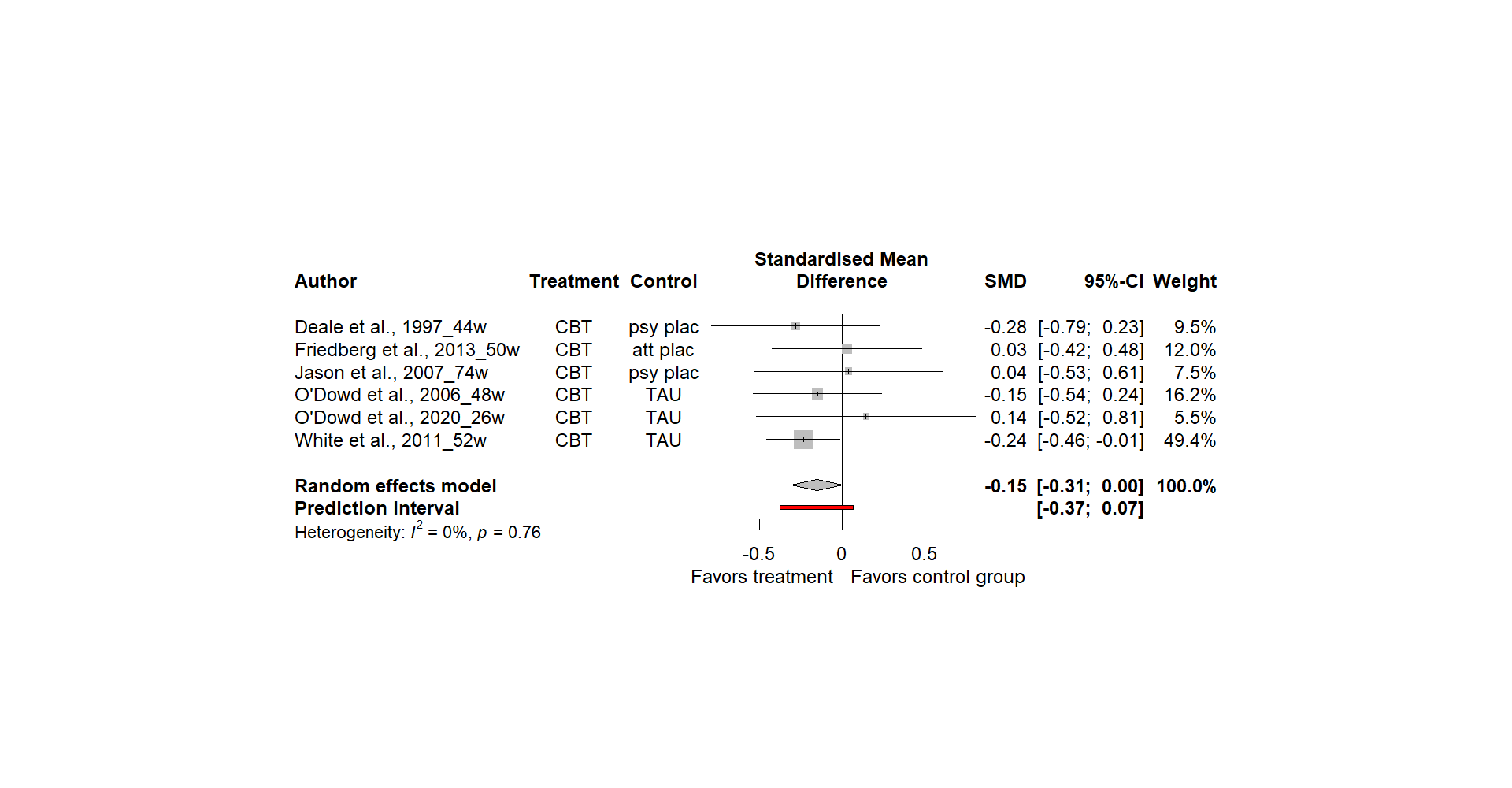


(C)


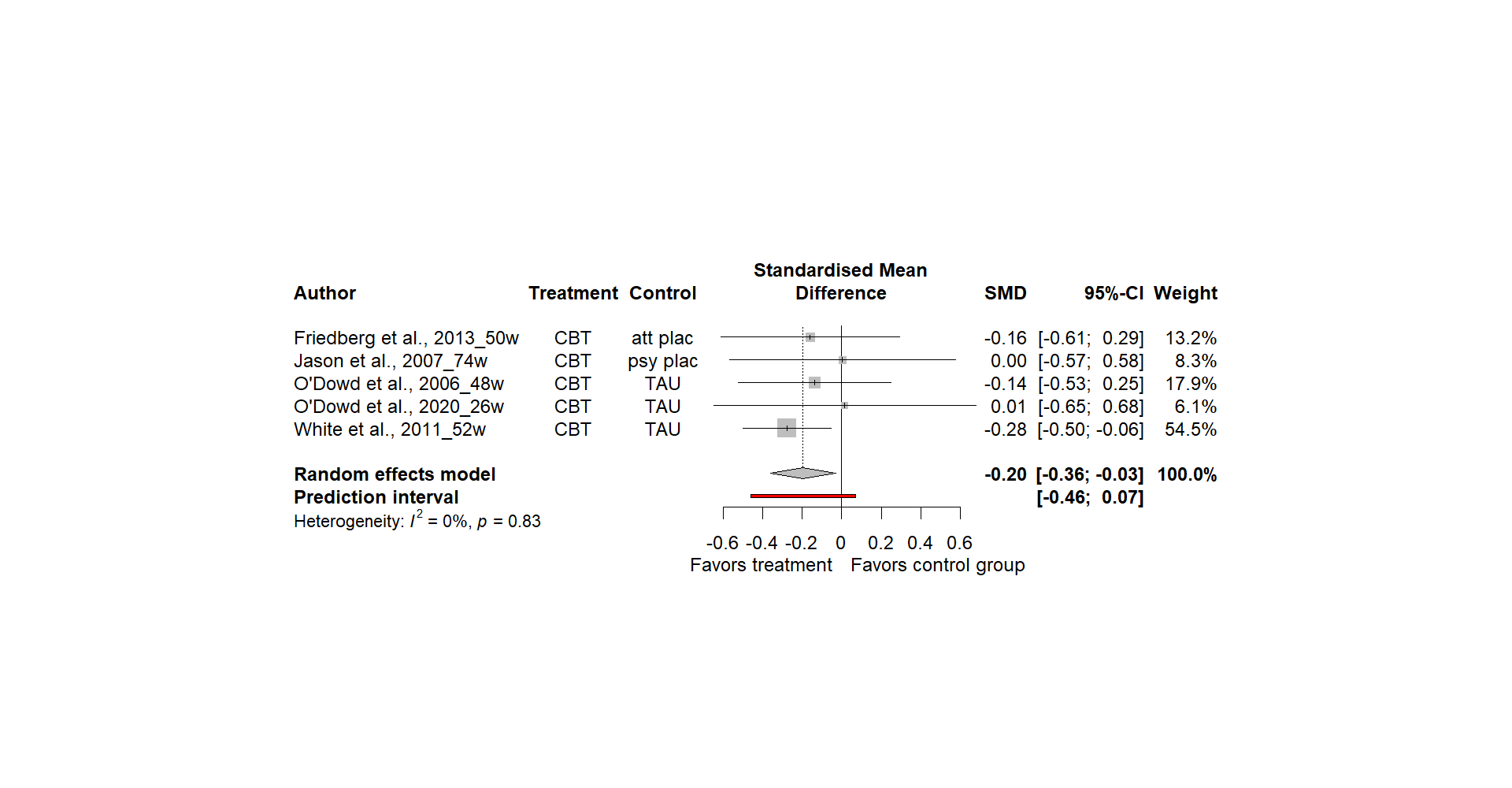


(D)

*Note.* A: fatigue at long-term follow-up; B: perceived health status at long-term follow-up; C: depression at long-term follow-up; D: anxiety at long-term follow-up. Note: For perceived health status positive effects indicate efficacy of the treatments, for all other outcomes negative effects indicate efficacy of the treatments.

**Figure S7**

*Forest plots – Acceptance (proportions in intervention groups)*
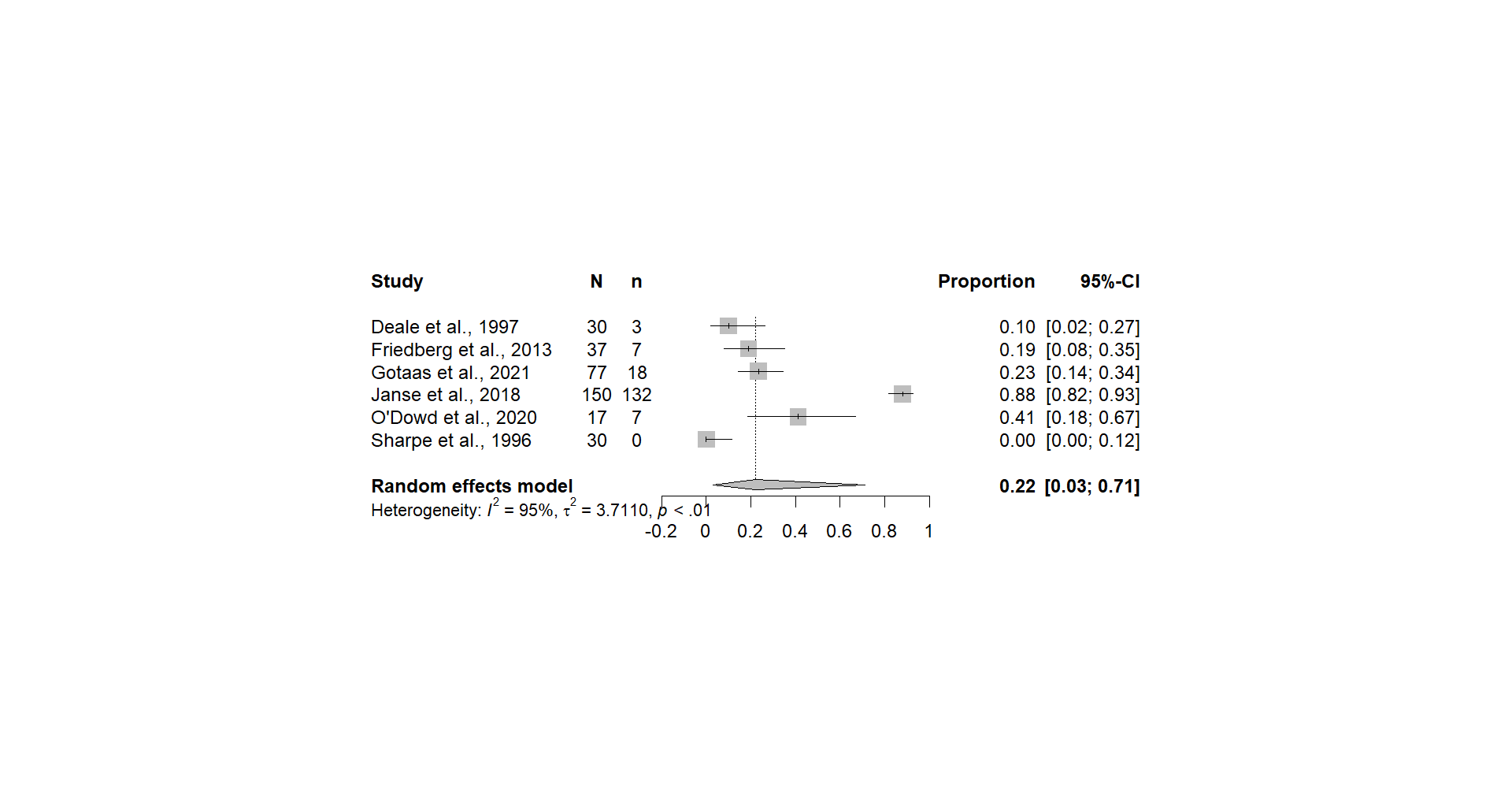


(A)


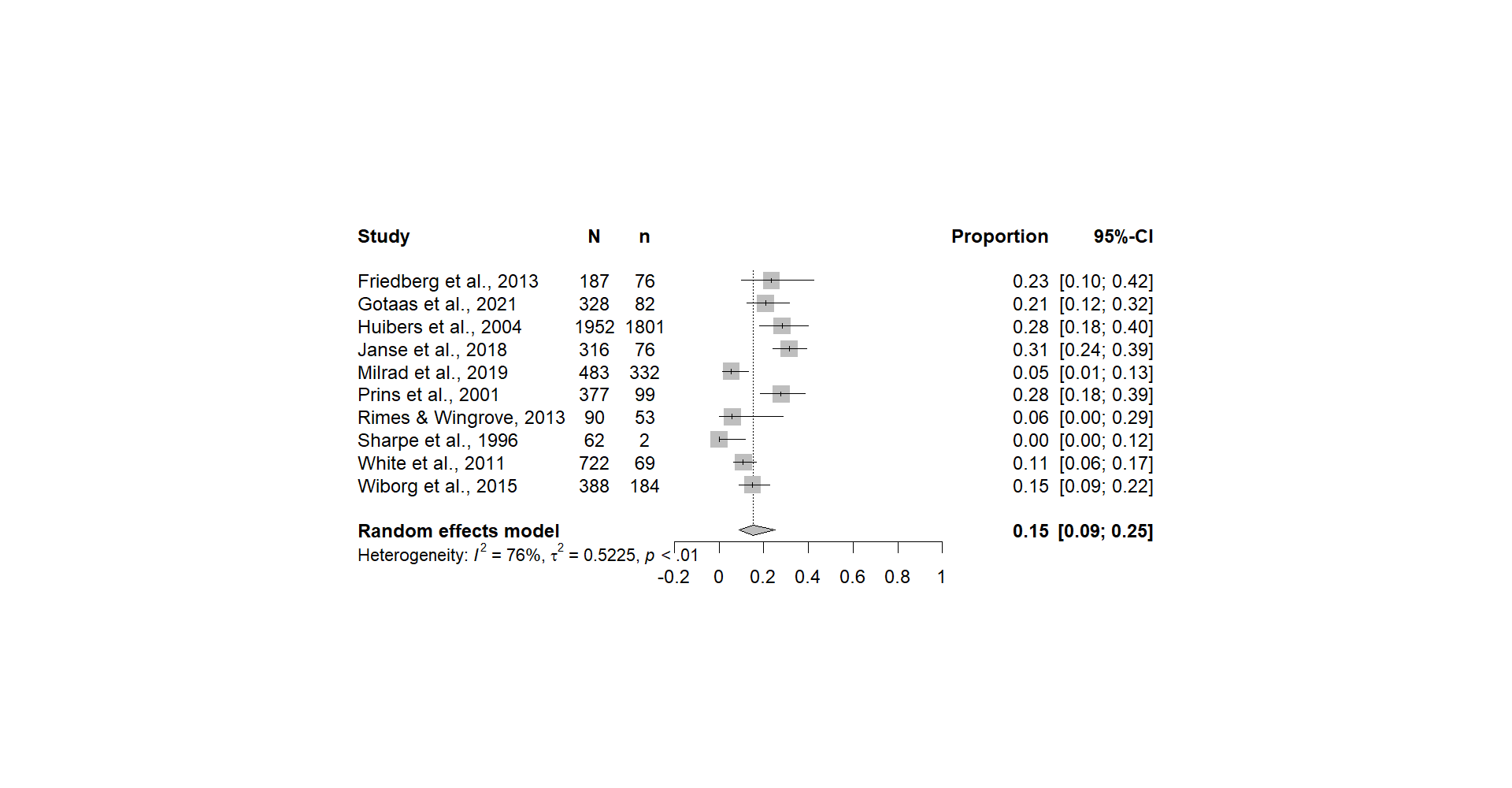


(B)


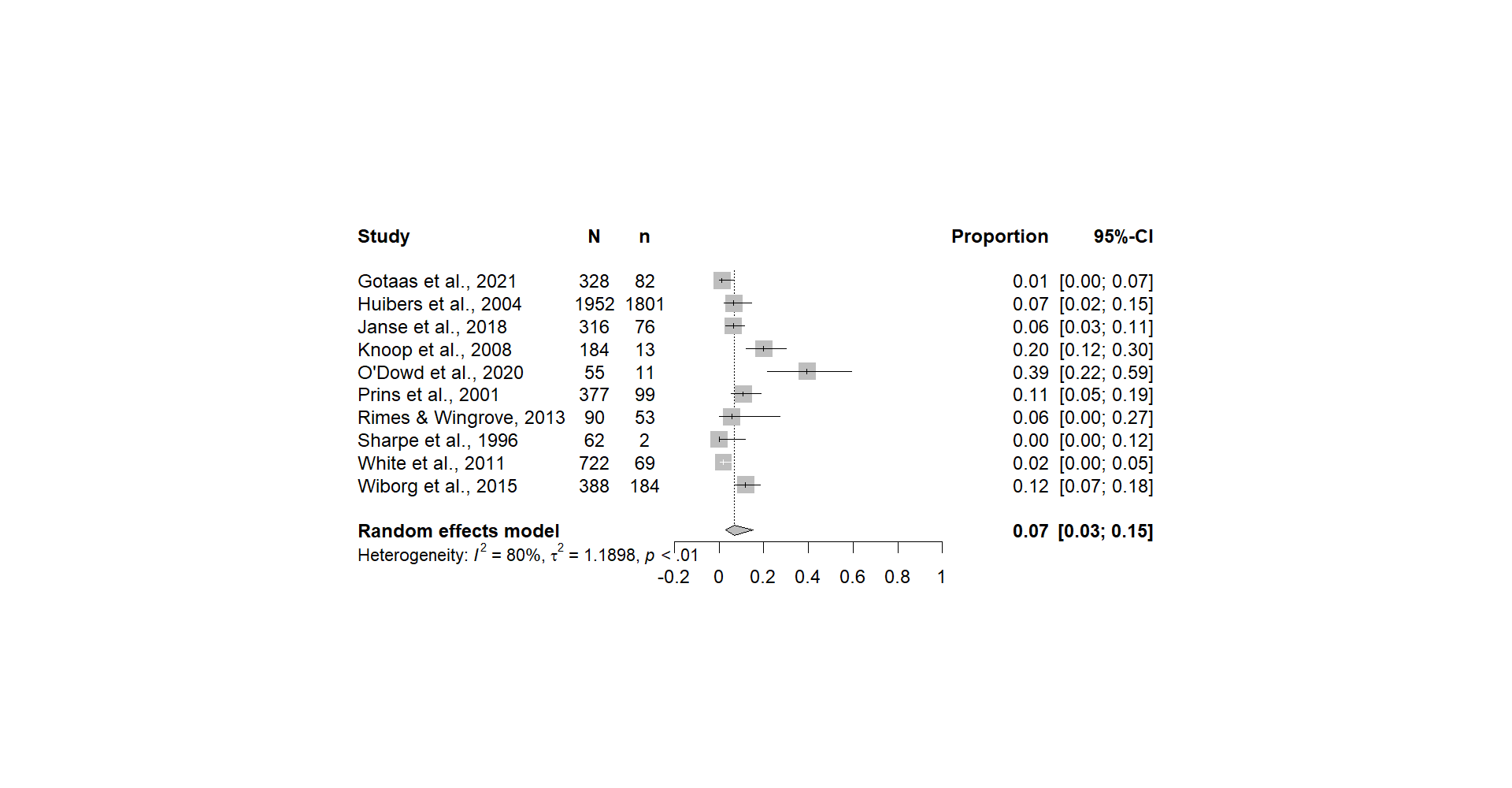


(C)


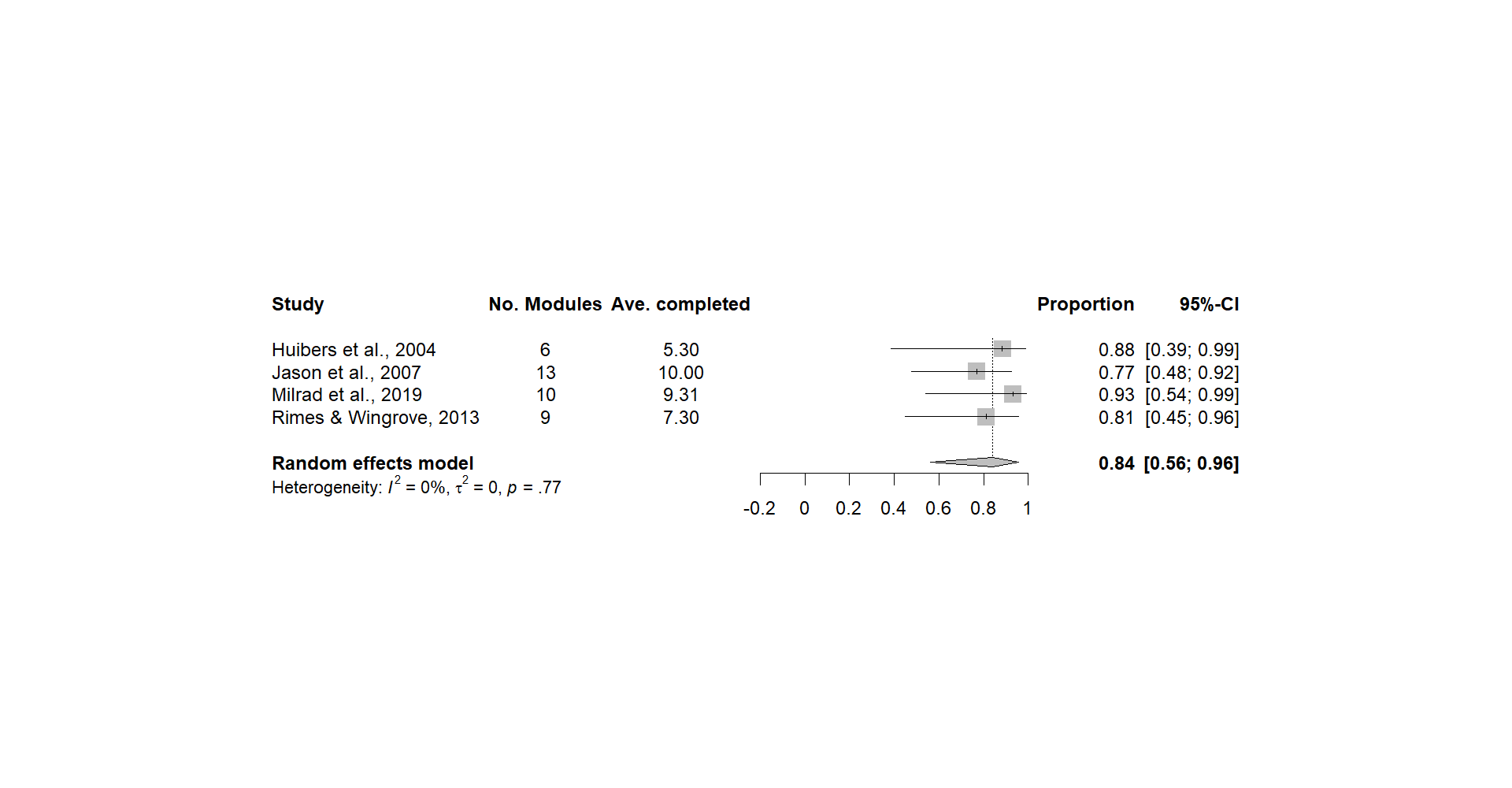


(D)

*Note.* A: non-completion; B: drop-out; C: treatment refusal; D: average proportion of sessions completed.

**Figure S8**

*Forest plots – Acceptance (relative risks)*


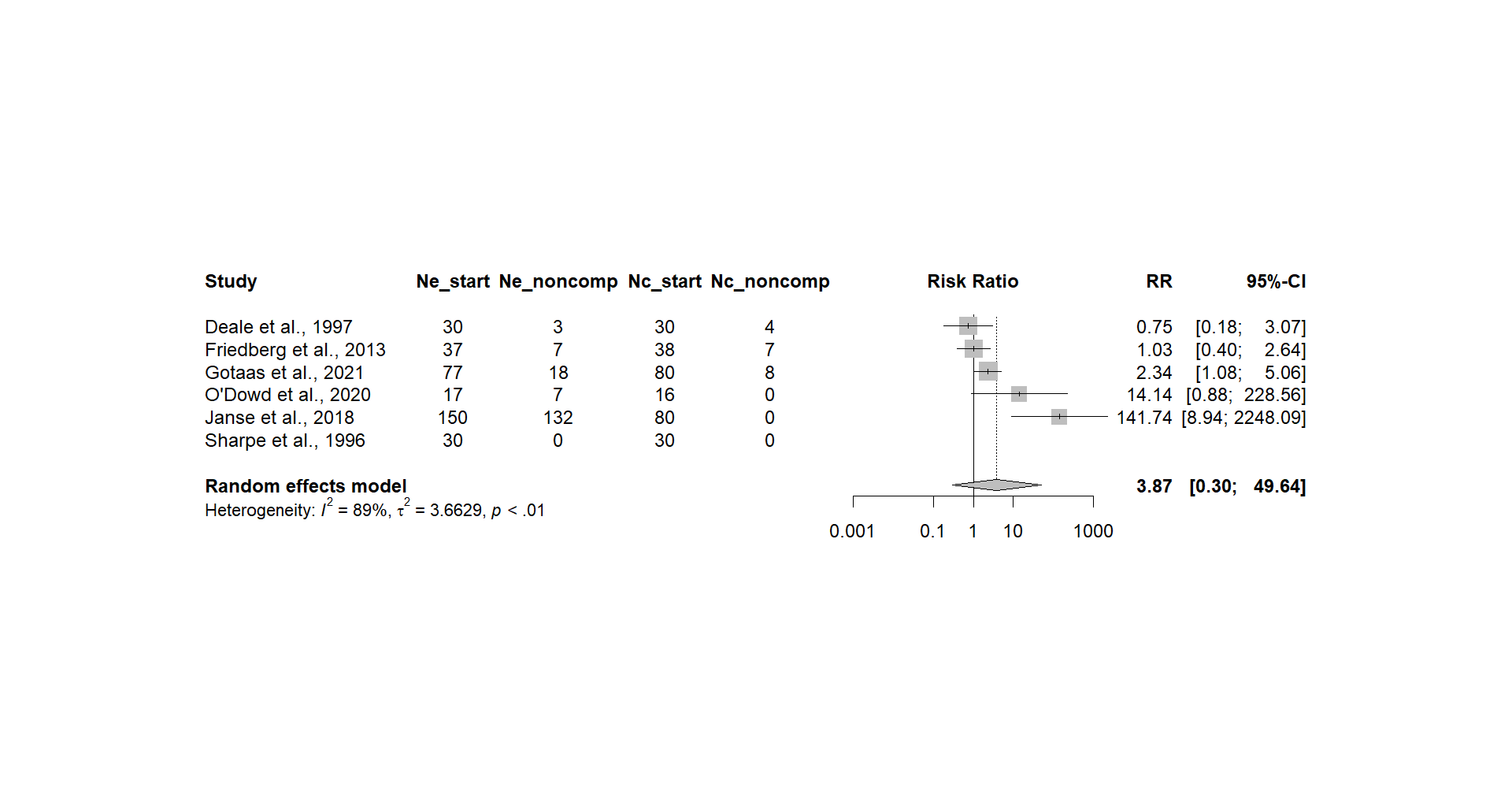


(A)


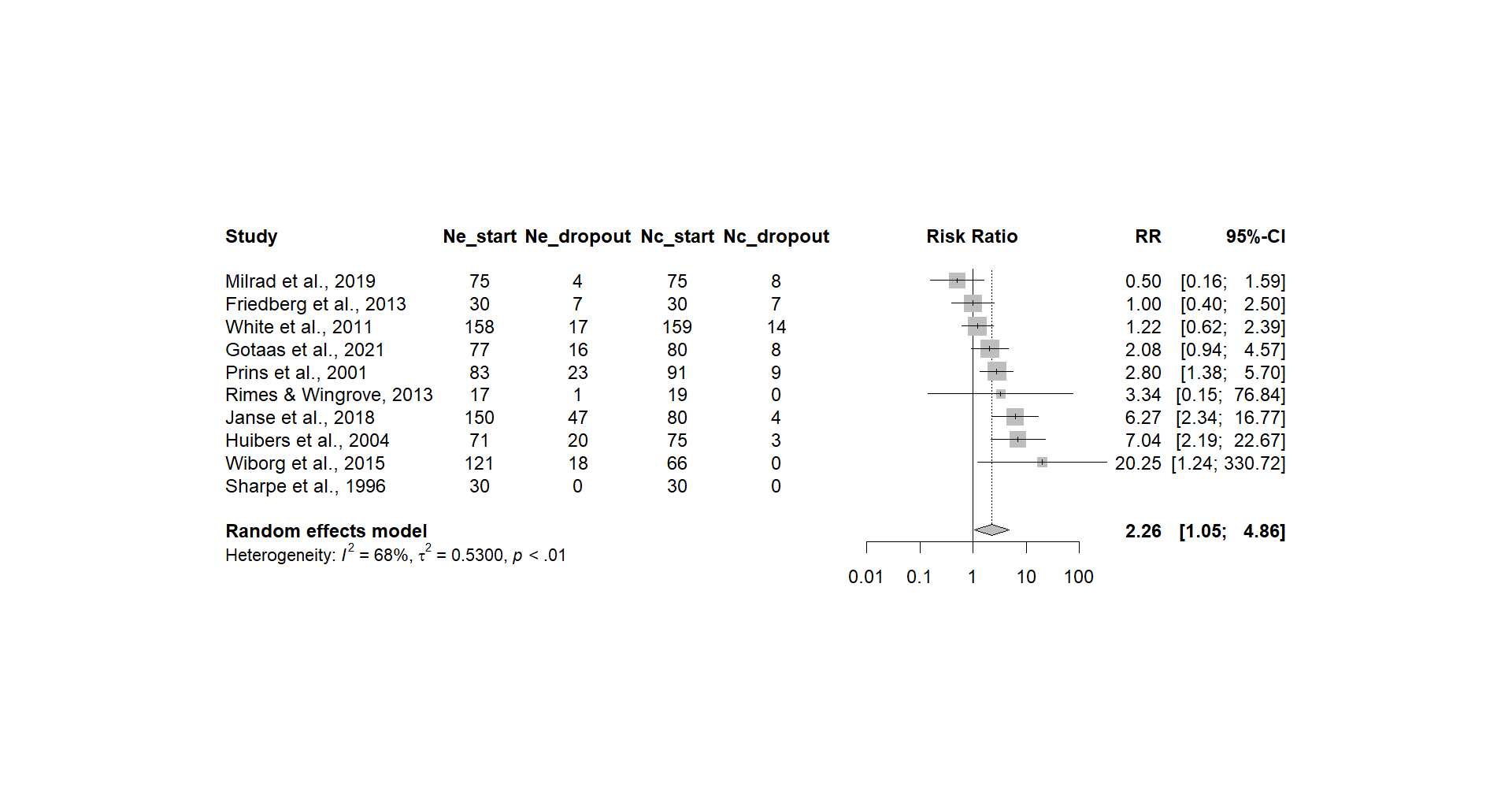


(B)

*Note.* A: non-completion; B: drop-out.

# **Outcome Measures**

**Table S2**

*List of outcome measures*

| Study | Fatigue | Depression | Anxiety | Perceived Health Status |
| --- | --- | --- | --- | --- |
| Deale et al., 1997 | CFQ/CFS | BDI |  | MOS |
| Friedberg et al., 2013 | FSS | BDI | BAI | SF-36-PF |
| Gotaas et al., 2021 | CFQ/CFS | HADS-D^a^ | HADS-A^a^ | SF-36-PF |
| Huibers et al., 2004 | CIS |  |  | SF-36-PF |
| Janse et al., 2018 | CIS |  |  | SF-36-PF |
| Jason et al., 2007 | FSS^a^ | BDI-II^a^ | BAI^a^ | MOS^a^ |
| Knoop et al., 2008 | CIS |  |  | SF-36-PF |
| Milrad et al., 2019 | FSI | CES-D |  |  |
| O’Dowd et al., 2006 |  | HADS-D | HADS-A | SF-36-PF |
| O’Dowd et al., 2020 | CFQ/CFS ^a^ | HADS-D^a^ | HADS-A^a^ | SF-36-PF^a^ |
| Prins et al., 2001 | CIS |  |  | EQ-5D |
| Rimes & Wingrove, 2013 | CFQ/CFS | HADS-D | HADS-A | PF-10 |
| Sharpe et al., 1996 | NRS^a^ | HADS-D^a^ | HADS-A^a^ |  |
| White et al., 2011 | CFQ/CFS | HADS-D^a^ | HADS-A^a^ | SF-36-PF |
| Wiborg et al., 2015 | CIS |  |  | SF-36-PF |

*Note.* BAI = Beck Anxiety Inventory; BDI = Beck Depression Inventory; BDI-II = Beck Depression Inventory-II; CES-D = Center for Epidemiologic Studies Depression Scale; CFQ/CFS = Chalder Fatigue Scale; CIS = Checklist Individual Strength – Fatigue subscale; EQ-5D = EuroQol Five-Dimensional Questionnaire; FSI = Fatigue Symptom Inventory; FSS = Fatigue Severity Scale; HADS-A = Hospital Anxiety and Depression Scale – Anxiety; HADS-D = Hospital Anxiety and Depression Scale – Depression; MOS = Medical Outcomes Study Short-Form General Health Survey physical functioning scale (previous version of the SF-36); PF-10 = Physical Functioning scale; SF-36-PF = Short Form Health Survey – Physical Functioning Subscale.
^a^ no effect sizes could be calculated at post-treatment due to missing values

# **Results Moderator Analyses – Meta-regressions**

**Table S3**

*Meta-regression analyses for therapy dosage*

| Outcome | $\tau_{unexplained}^{2}$ | *I*² | *R*² | $p_{moderator}$ | Regression weight | Intercept |
| --- | --- | --- | --- | --- | --- | --- |
| Fatigue | 0.01 | 17.37% | 91.17% | 0.0003 | -0.0005 | -0.02 |
| Perceived Health Status | 0.06 | 63.91% | 33.38% | 0.10 | 0.0004 | -0.05 |
| Depression | 0 | 0.00% | 0.00% | 0.66 | -0.0001 | -0.27 |
| Anxiety | 0 | 0.00% | 0.00% | 0.99 | -0.00 | -0.34 |
| Non-completion | 0.12 | 25.55% |  | 0.13 | -0.0025 | -0.68 |
| Drop-out | 0.52 | 79.51% |  | 0.40 | -0.0005 | -1.47 |
| Ave. prop. sessions | 0 | 0.00% |  | 0.77 | -0.0004 | 1.96 |

*Note.* Effect on efficacy outcomes at post-treatment; $\tau_{unexplained}^{2}$ = estimated amount of residual heterogeneity; *I*² = residual heterogeneity/unaccounted variability; *R*² = amount of heterogeneity accounted for (could only be calculated for efficacy outcomes)

**Figure S9**

*Bubble plots – meta-regression analysis for therapy dosage (total time of therapy in minutes)*


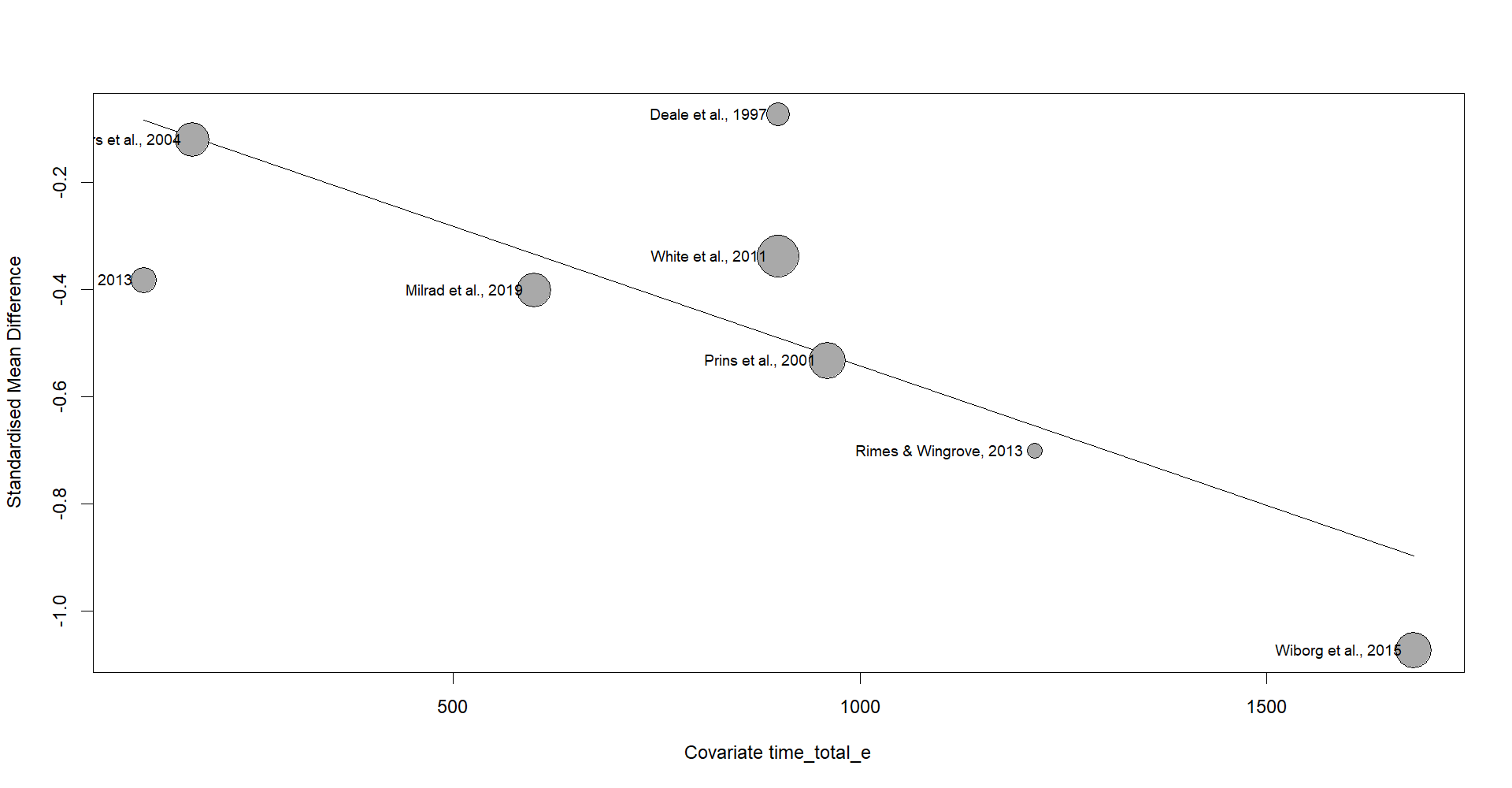


(A)


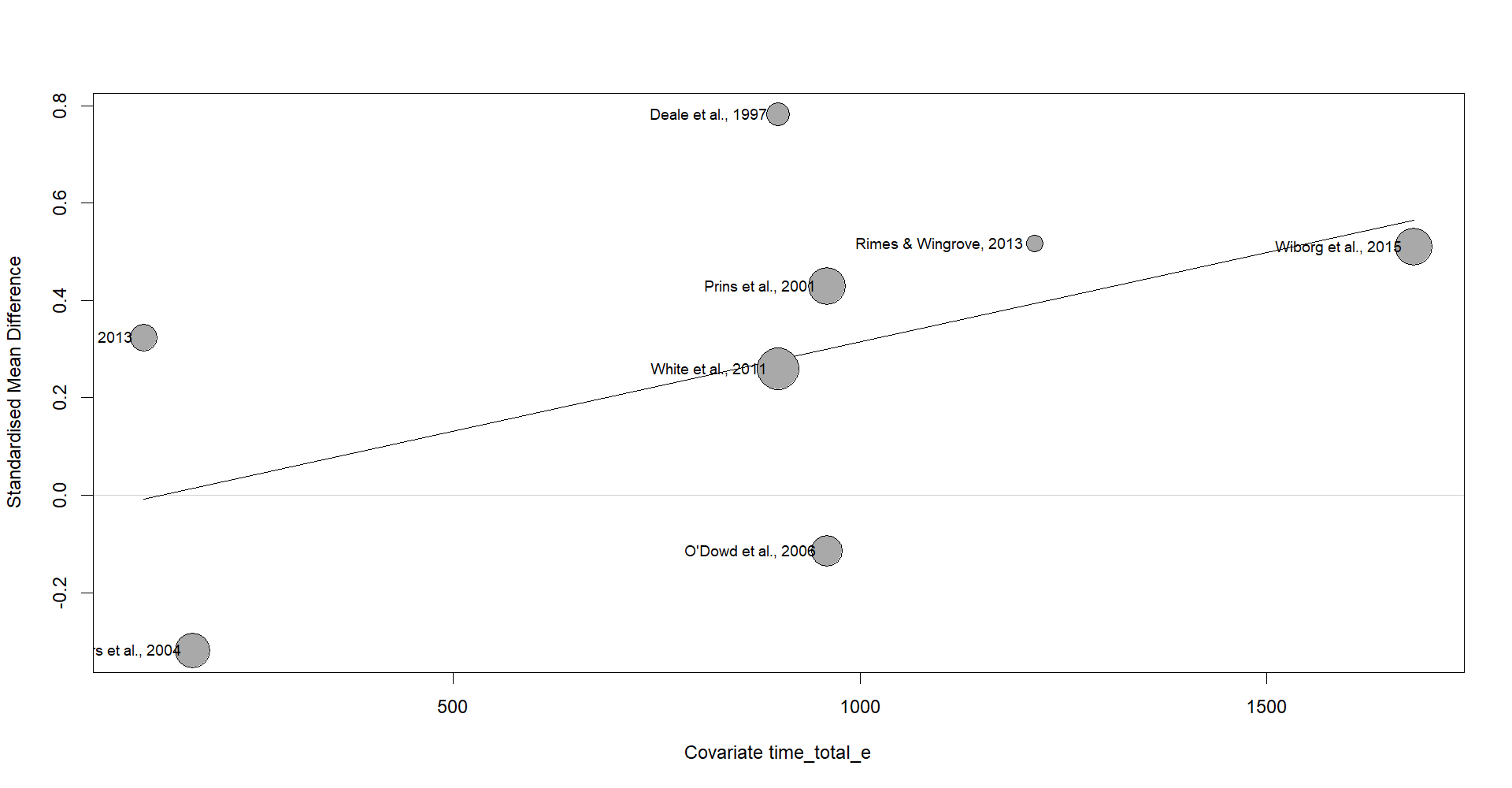


(B)


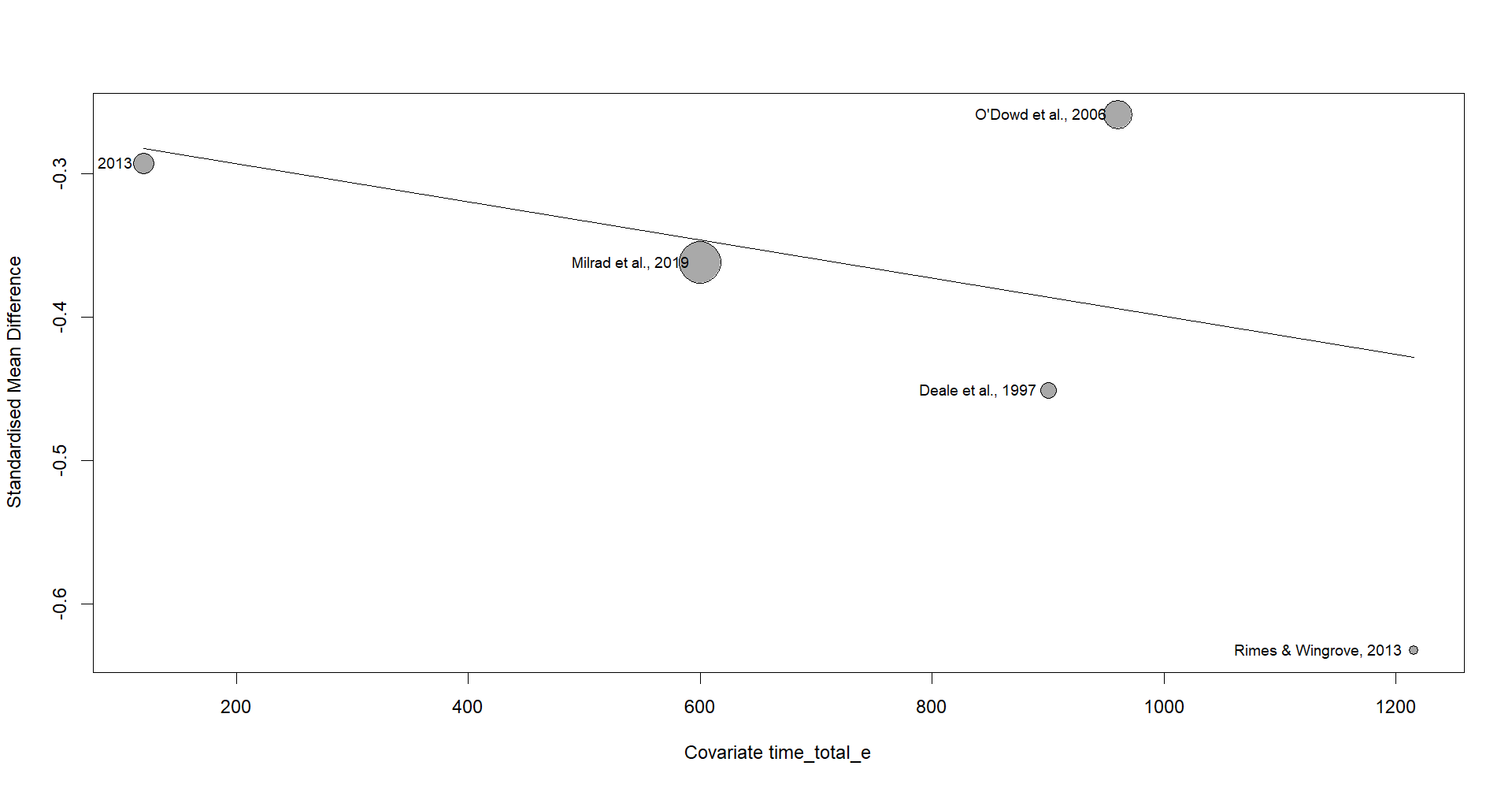


(C)


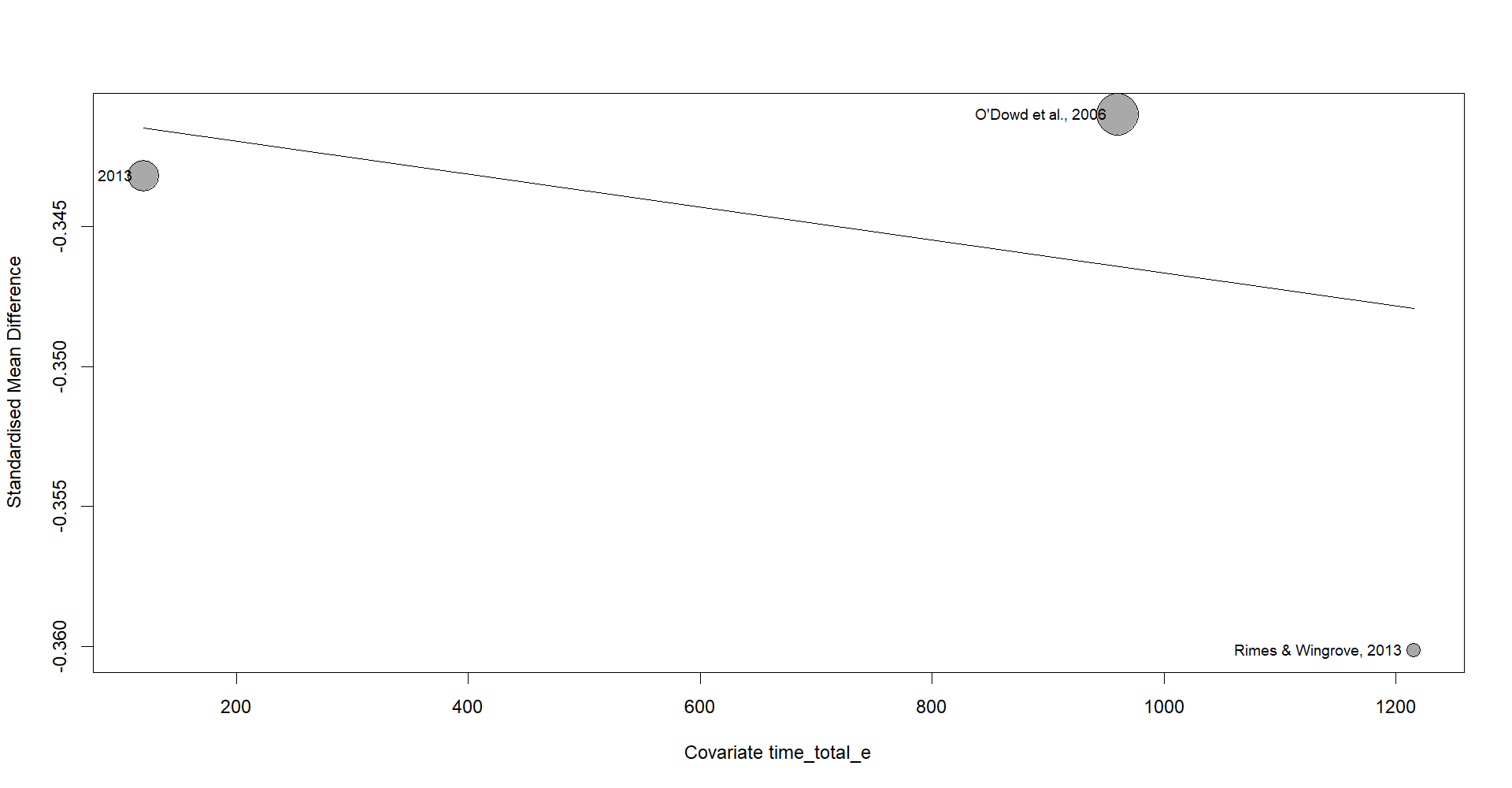


(D)


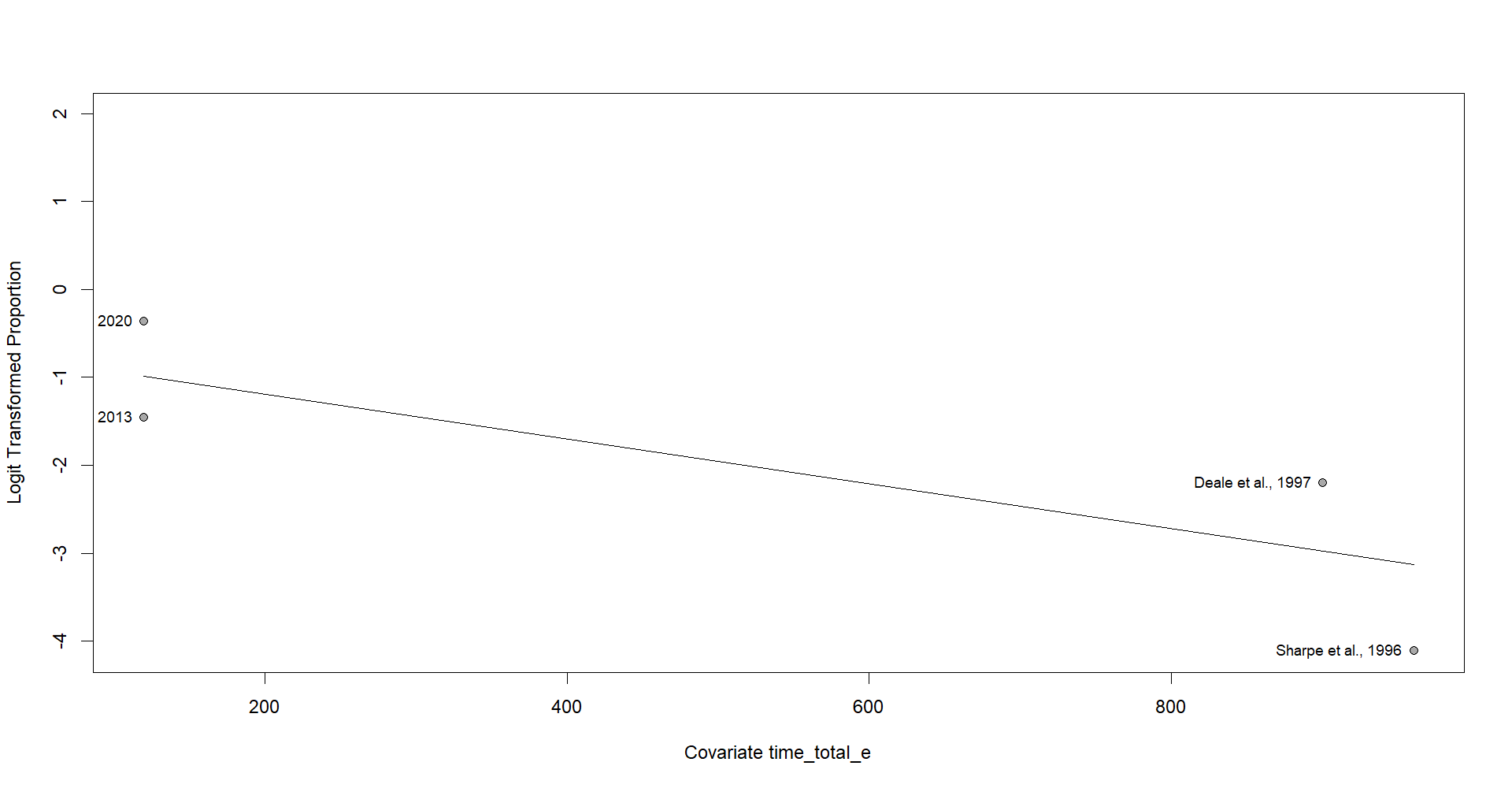


(E)


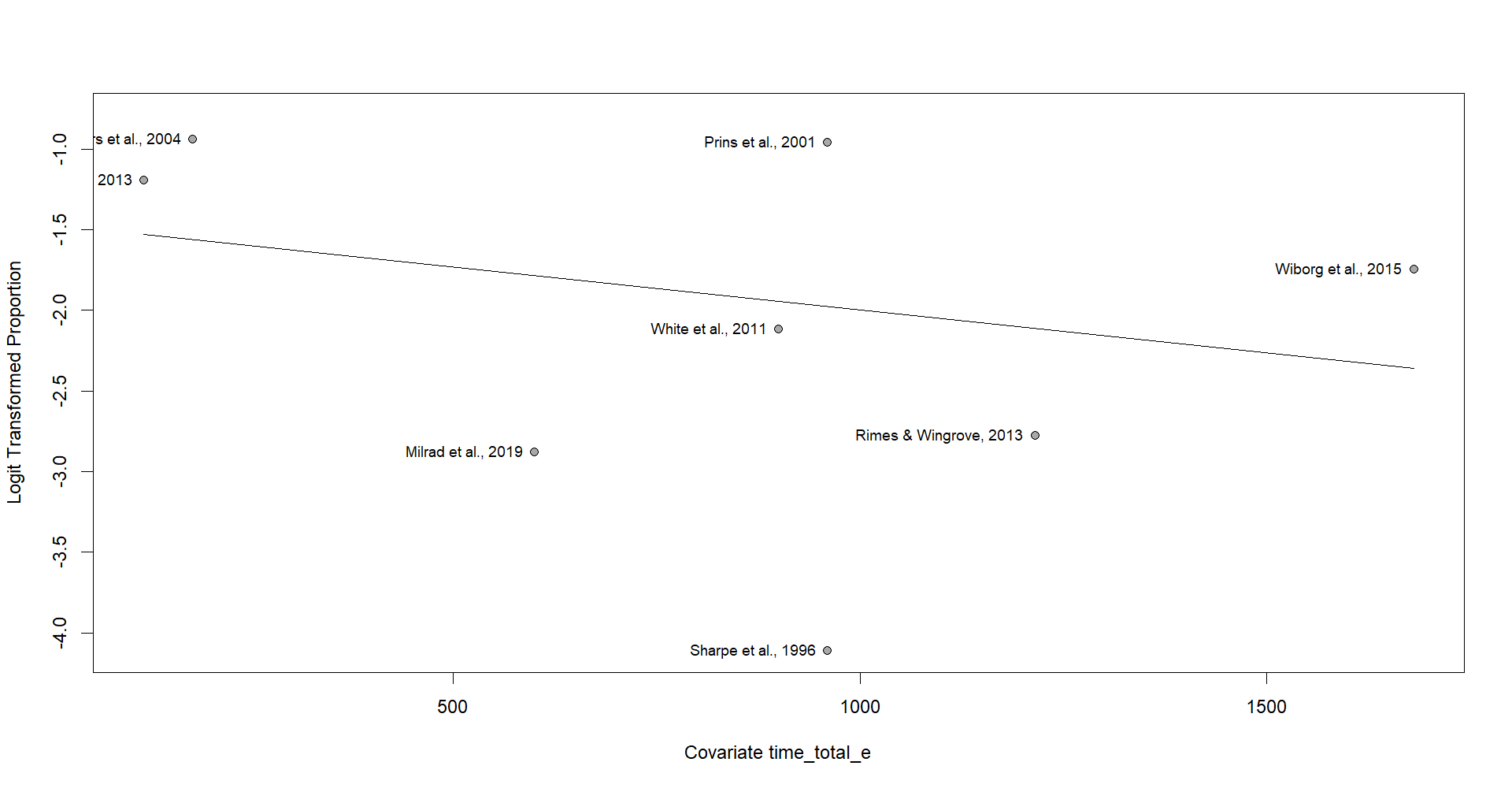


(F)


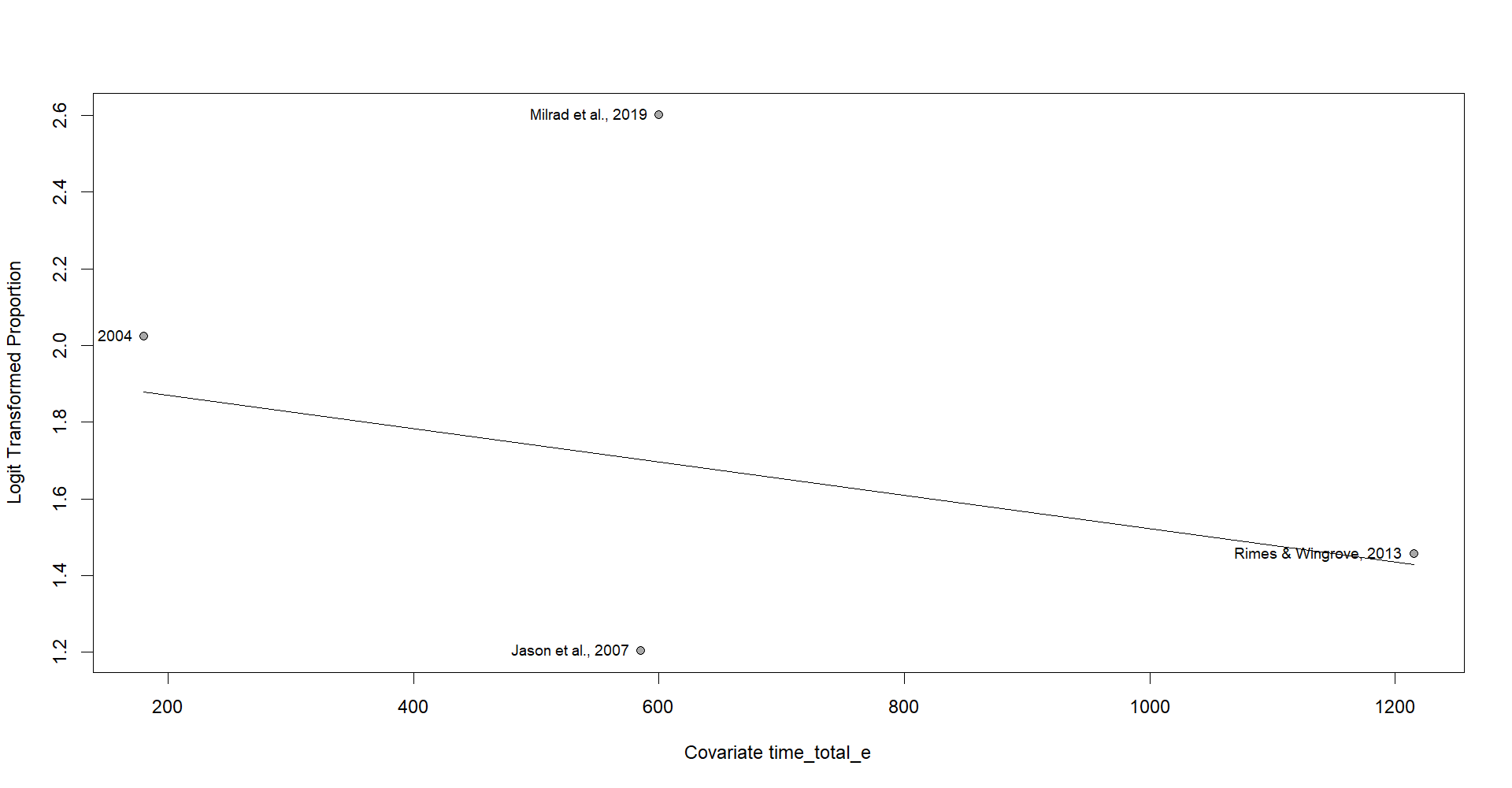


(G)

*Note.* A: bubble plot for the effect of therapy dosage on fatigue at post-treatment; B: bubble plot for the effect of therapy dosage on perceived health status at post-treatment; C: bubble plot for the effect of therapy dosage on depression at post-treatment; D: bubble plot for the effect of therapy dosage on anxiety at post-treatment; E: bubble plot for the effect of therapy dosage on non-completion; F: bubble plot for the effect of therapy dosage on drop-out; G: bubble plot for the effect of therapy dosage on the average proportion of sessions completed.

**Table S4**

*Meta-regression analyses for number of sessions*

| Outcome | $\tau_{unexplained}^{2}$ | *I*² | *R*² | $p_{moderator}$ | Regression weight | Intercept |
| --- | --- | --- | --- | --- | --- | --- |
| Fatigue | 0.05 | 65.44% | 5.11% | 0.27 | -0.0222 | -0.25 |
| Perceived Health Status | 0.04 | 55.37% | 49.21% | 0.03 | 0.0387 | -0.14 |
| Depression | 0 | 0.00% | 0.00% | 0.63 | -0.0142 | -0.24 |
| Anxiety | 0 | 0.00% | 0.00% | 0.99 | -0.0007 | -0.34 |
| Non-completion | 2.88 | 93.61% |  | 0.26 | -0.1704 | 0.41 |
| Drop-out | 0.45 | 81.95% |  | 0.32 | -0.0529 | -1.09 |
| Ave. prop. sessions | 0 | 0.00% |  | 0.59 | -0.1241 | 2.95 |

*Note.* Effect on efficacy outcomes at post-treatment; $\tau_{unexplained}^{2}$ = estimated amount of residual heterogeneity; *I*² = residual heterogeneity/unaccounted variability; *R*² = amount of heterogeneity accounted for (could only be calculated for efficacy outcomes)

**Figure S10**

*Bubble plots – meta-regression analysis for number of sessions*


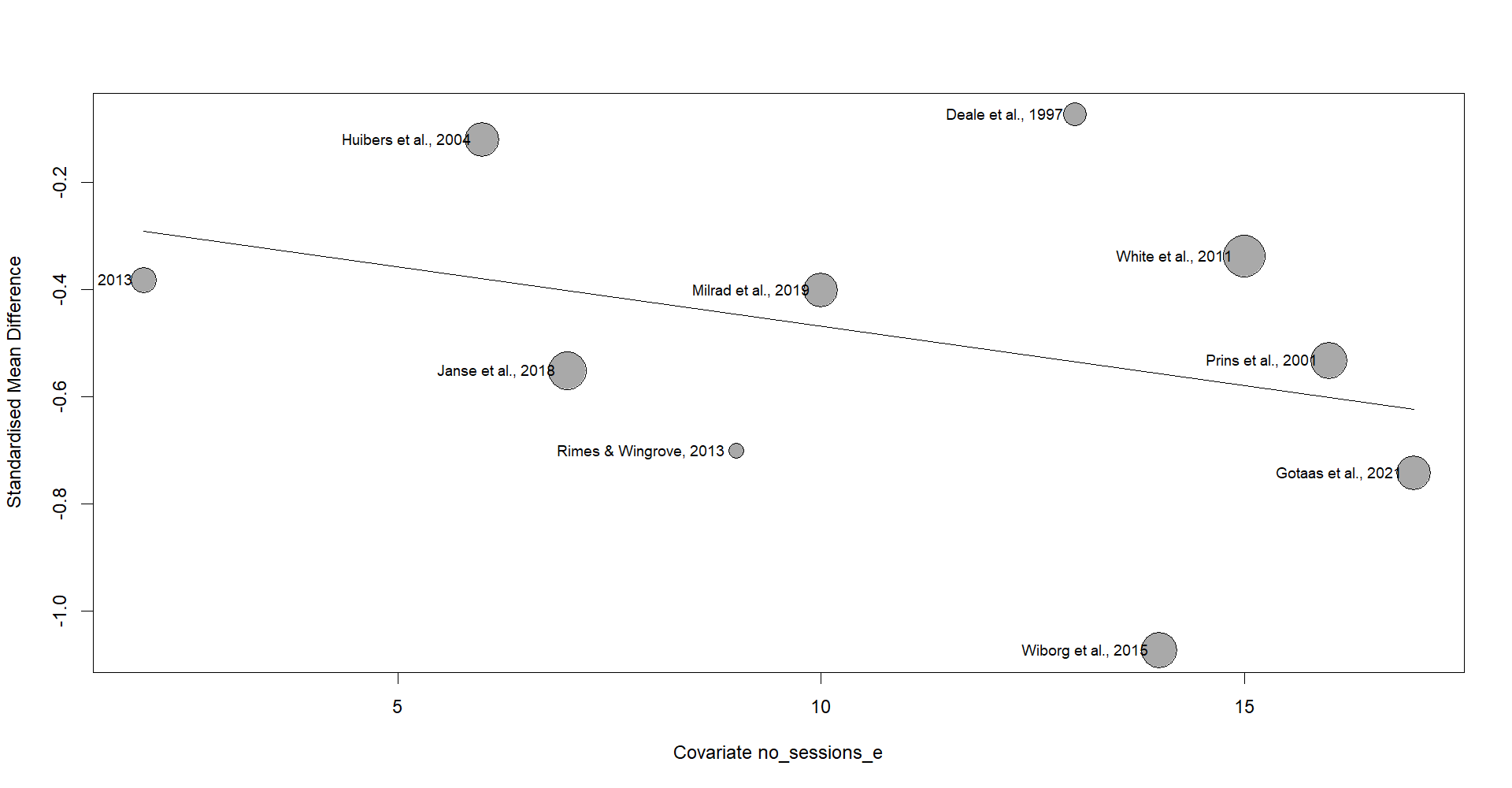


(A)


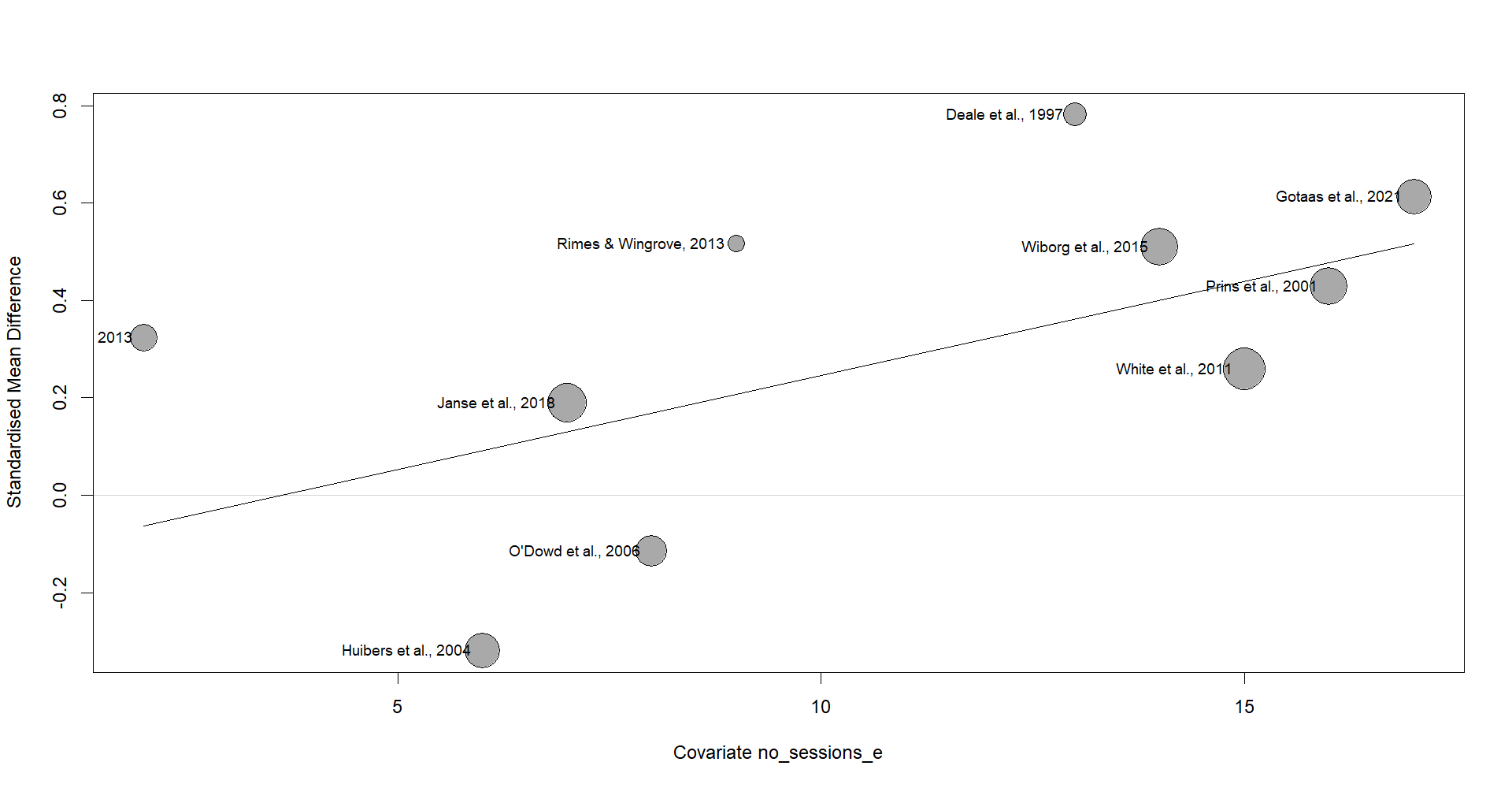


(B)


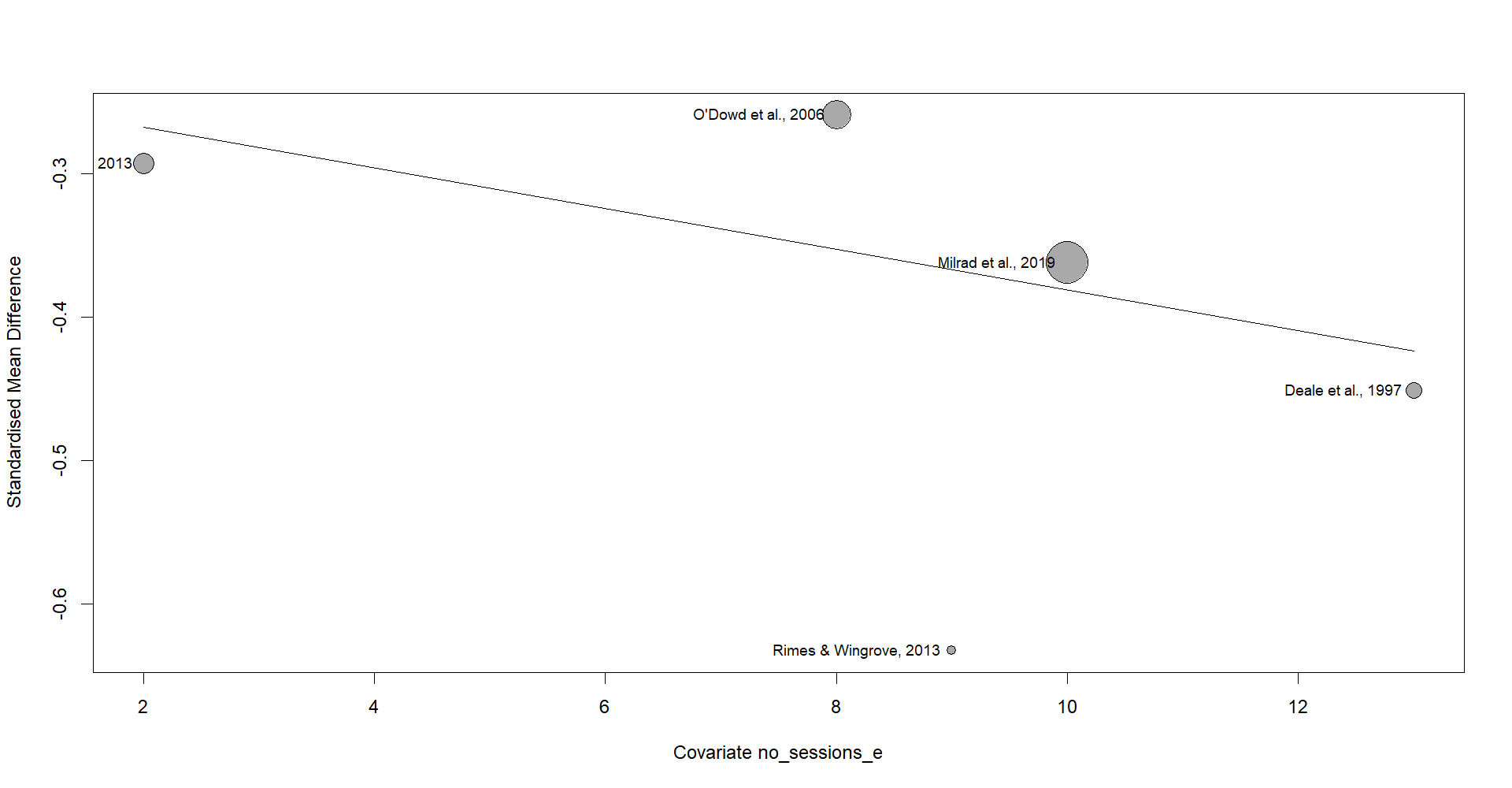


(C)


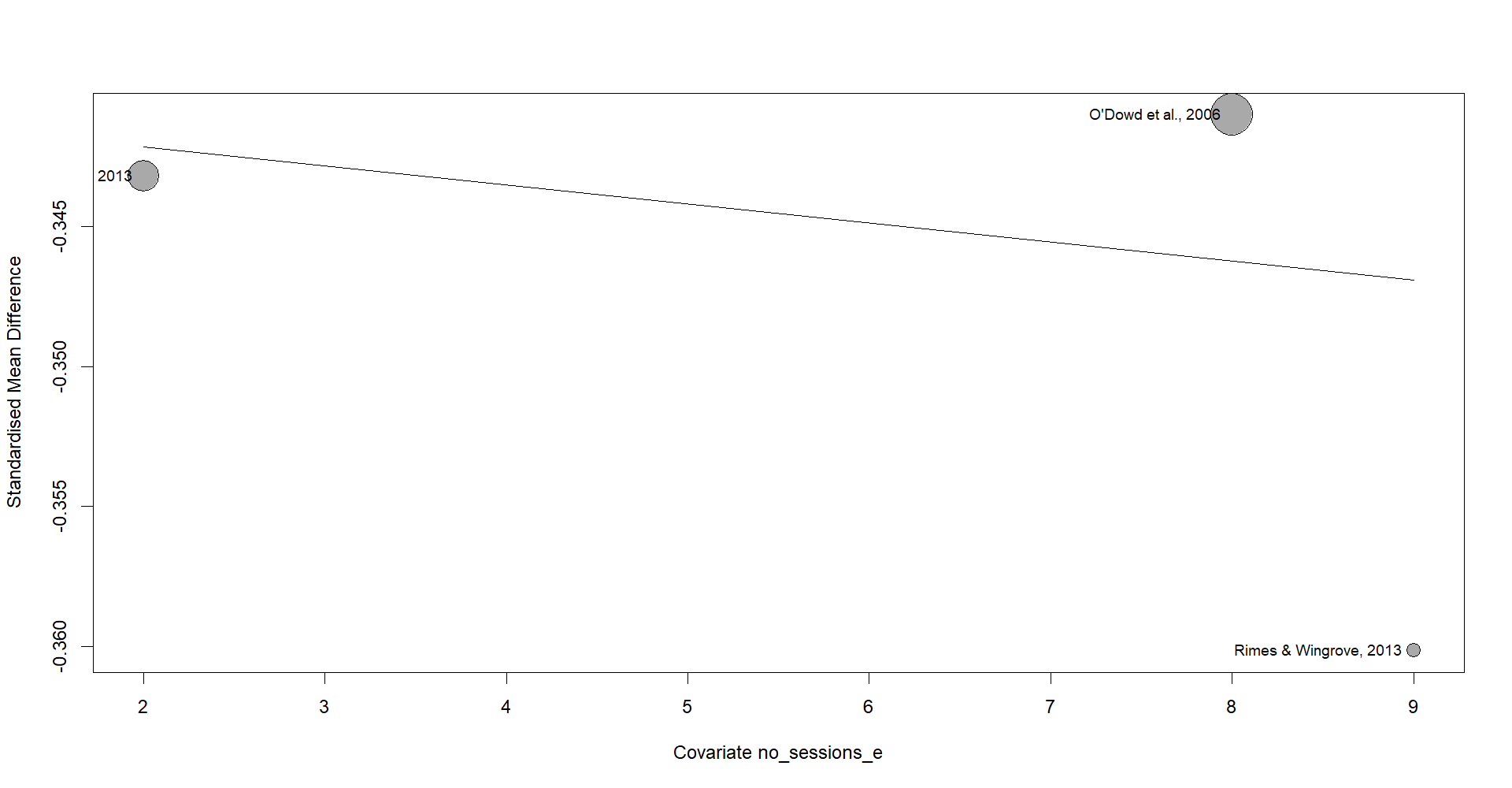


(D)


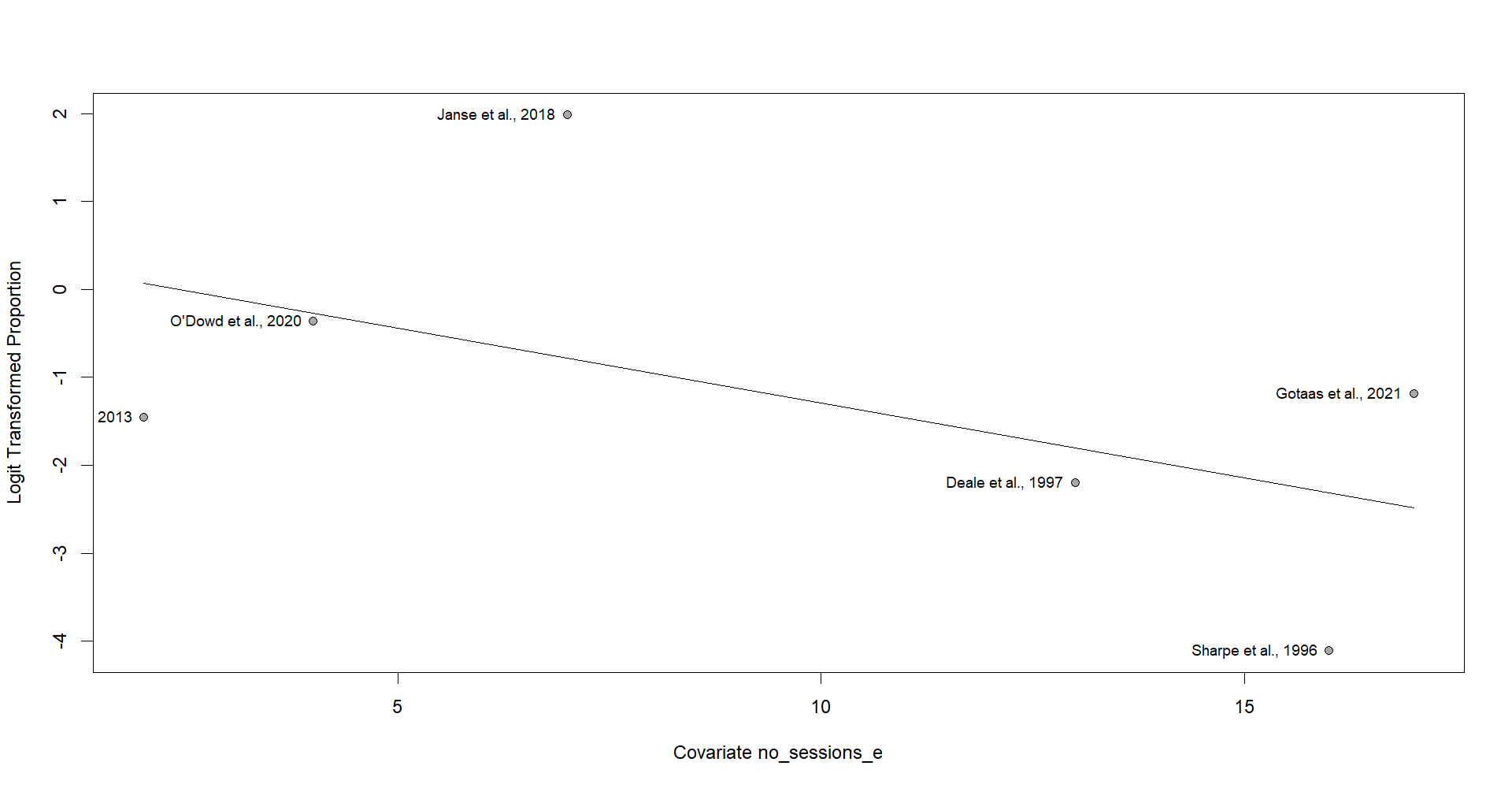


(E)


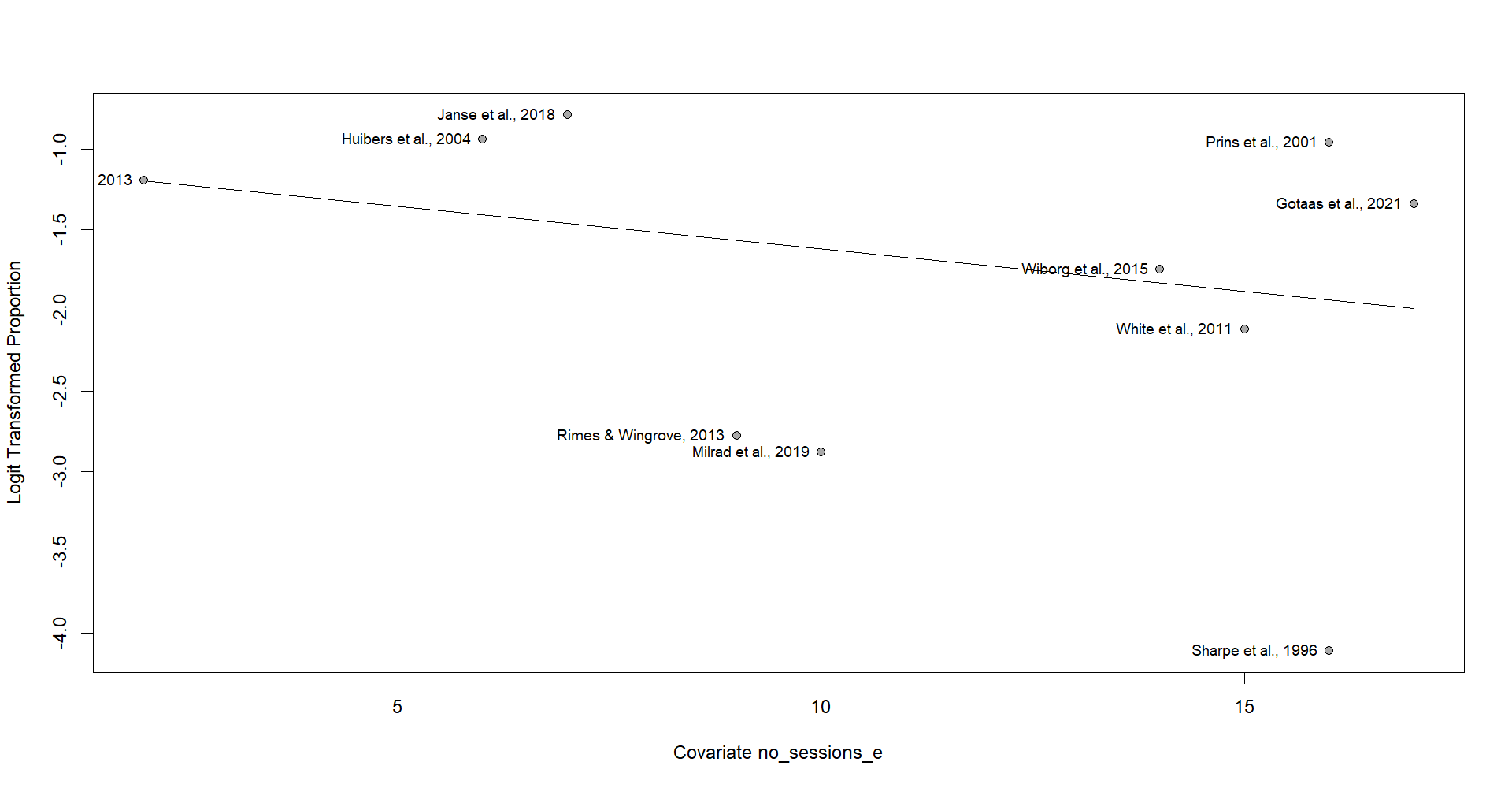


(F)


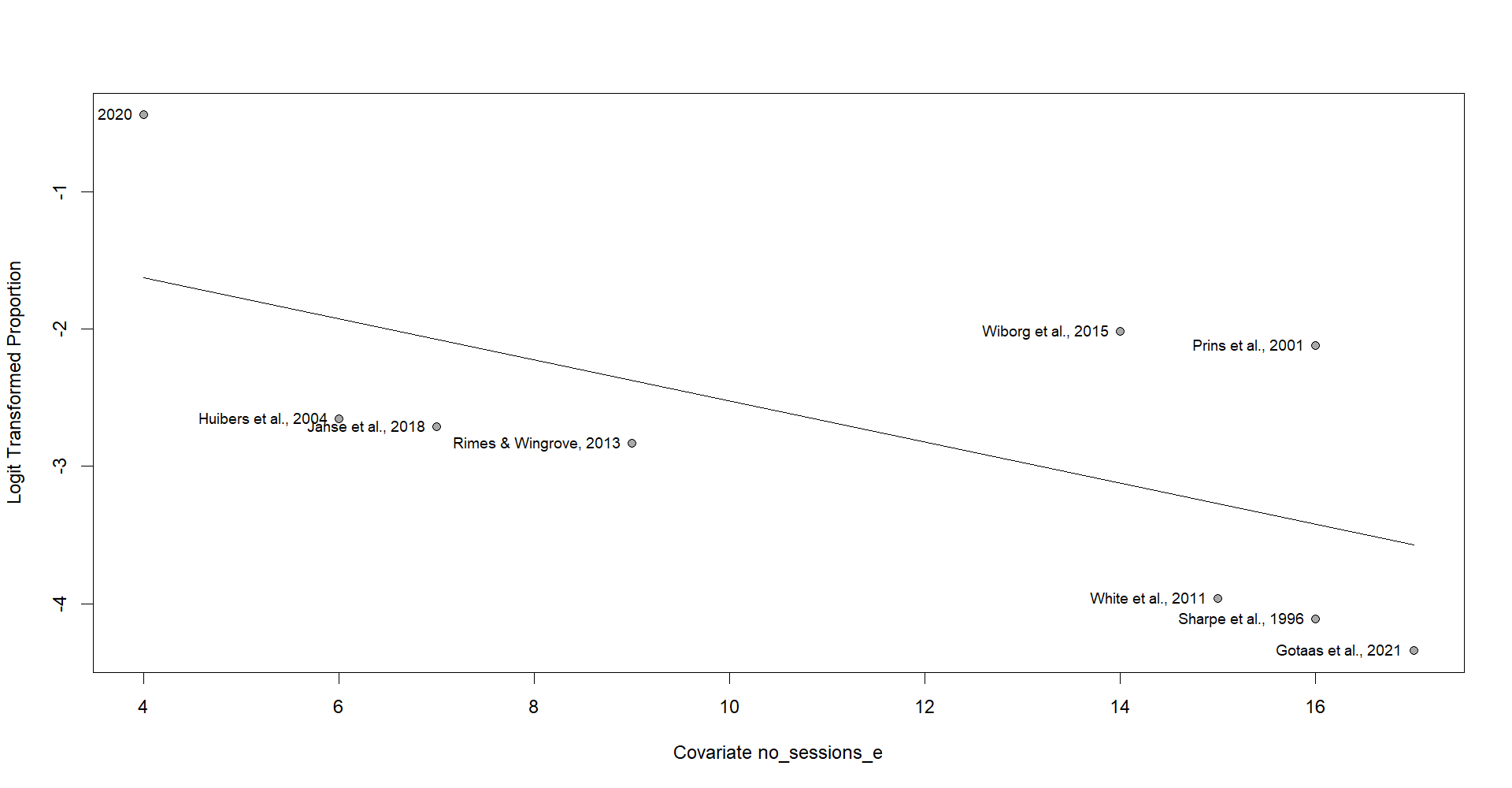


(G)


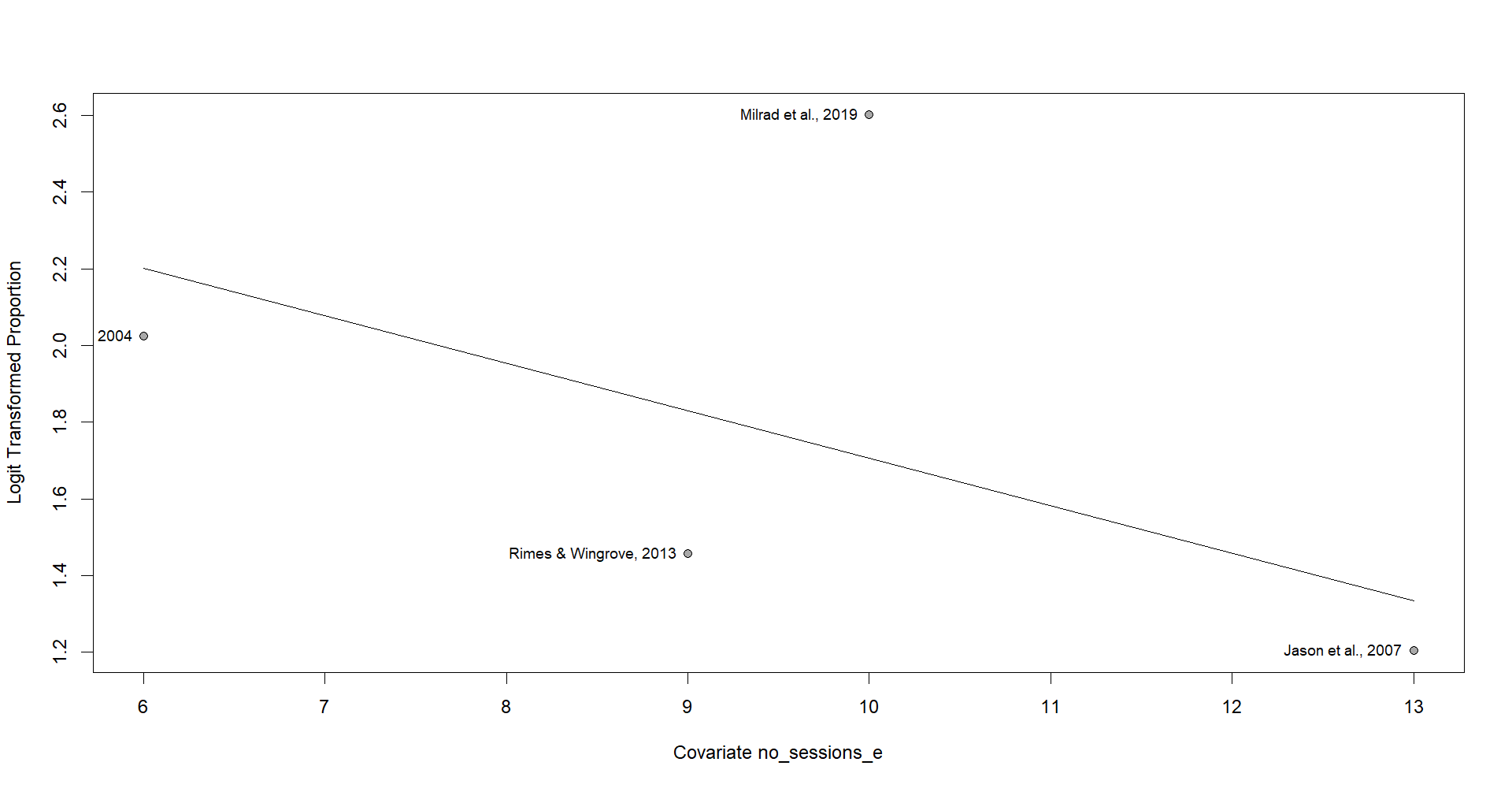


(H)

*Note.* A: bubble plot for the effect of number of sessions on fatigue at post-treatment; B: bubble plot for the effect of number of sessions on perceived health status at post-treatment; C: bubble plot for the effect of number of sessions on depression at post-treatment; D: bubble plot for the effect of number of sessions on anxiety at post-treatment; E: bubble plot for the effect of number of sessions on non-completion; F: bubble plot for the effect of number of sessions on drop-out; G: bubble plot for the effect of number of sessions on treatment refusal; H: bubble plot for the effect of number of sessions on the average proportion of sessions completed.

**Table S5**

*Meta-regression analyses for duration of therapy in weeks*

| Outcome | $\tau_{unexplained}^{2}$ | *I*² | *R*² | $p_{moderator}$ | Regression weight | Intercept |
| --- | --- | --- | --- | --- | --- | --- |
| Fatigue | 0.08 | 72.29% | 0.00% | 0.92 | -0.0013 | -0.46 |
| Perceived Health Status | 0.10 | 75.19% | 0.00% | 0.73 | 0.005 | 0.21 |
| Depression | 0 | 0.00% | 0.00% | 0.78 | 0.0074 | -0.46 |
| Anxiety | 0 | 0.00% | 0.00% | 0.96 | 0.0024 | -0.38 |
| Non-completion | 0.39 | 61.53% |  | 0.21 | -0.1795 | 1.11 |
| Drop-out | 0.46 | 79.48% |  | 0.42 | 0.0257 | -2.35 |
| Ave. prop. sessions | 0 | 0.00% |  | 0.55 | -0.0409 | 2.34 |

*Note.* Effect on efficacy outcomes at post-treatment; $\tau_{unexplained}^{2}$ = estimated amount of residual heterogeneity; *I*² = residual heterogeneity/unaccounted variability; *R*² = amount of heterogeneity accounted for (could only be calculated for efficacy outcomes)

**Figure S11**

*Bubble plots – meta-regression analysis for duration of therapy (weeks)*


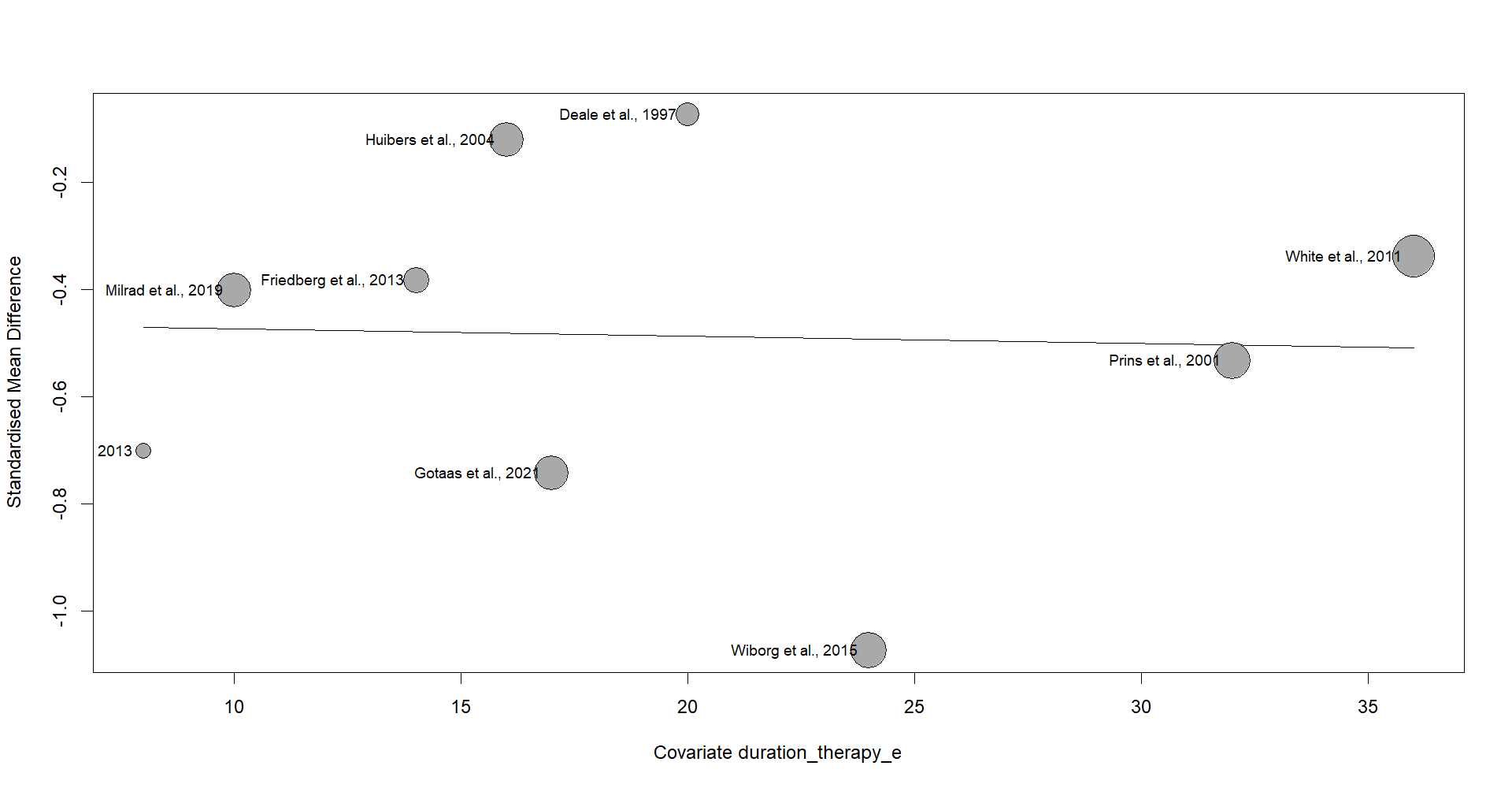


(A)


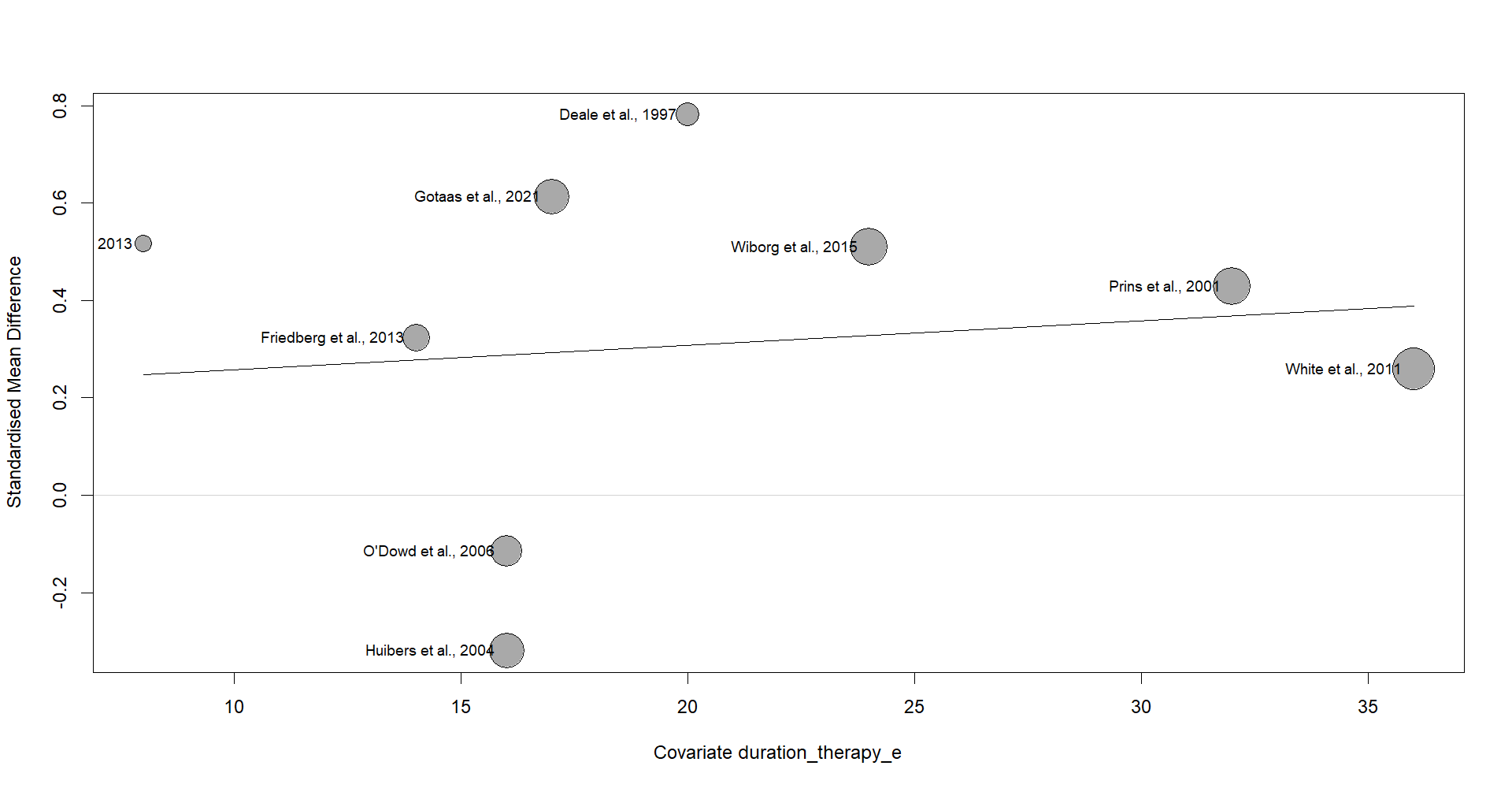


(B)


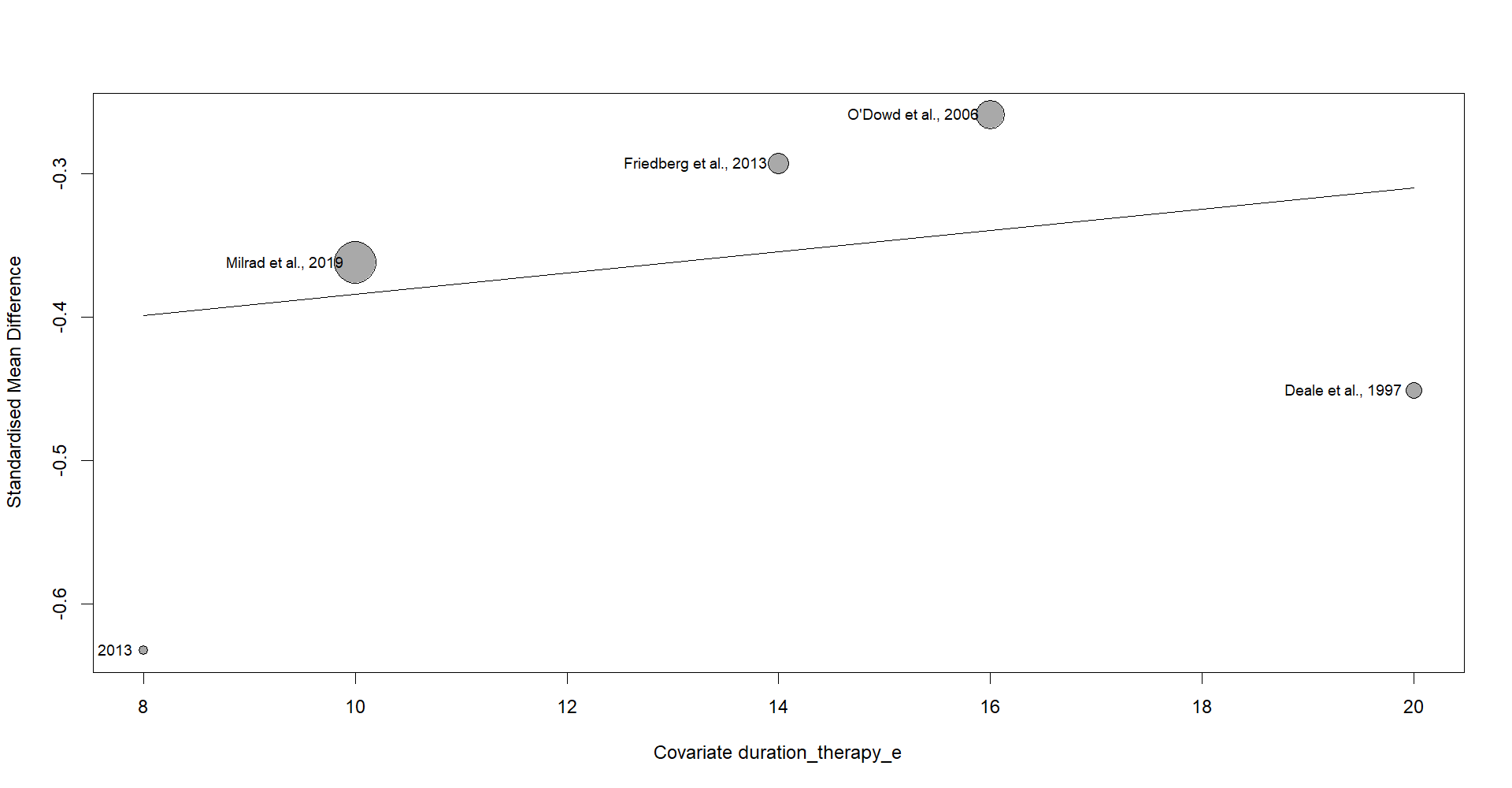


(C)


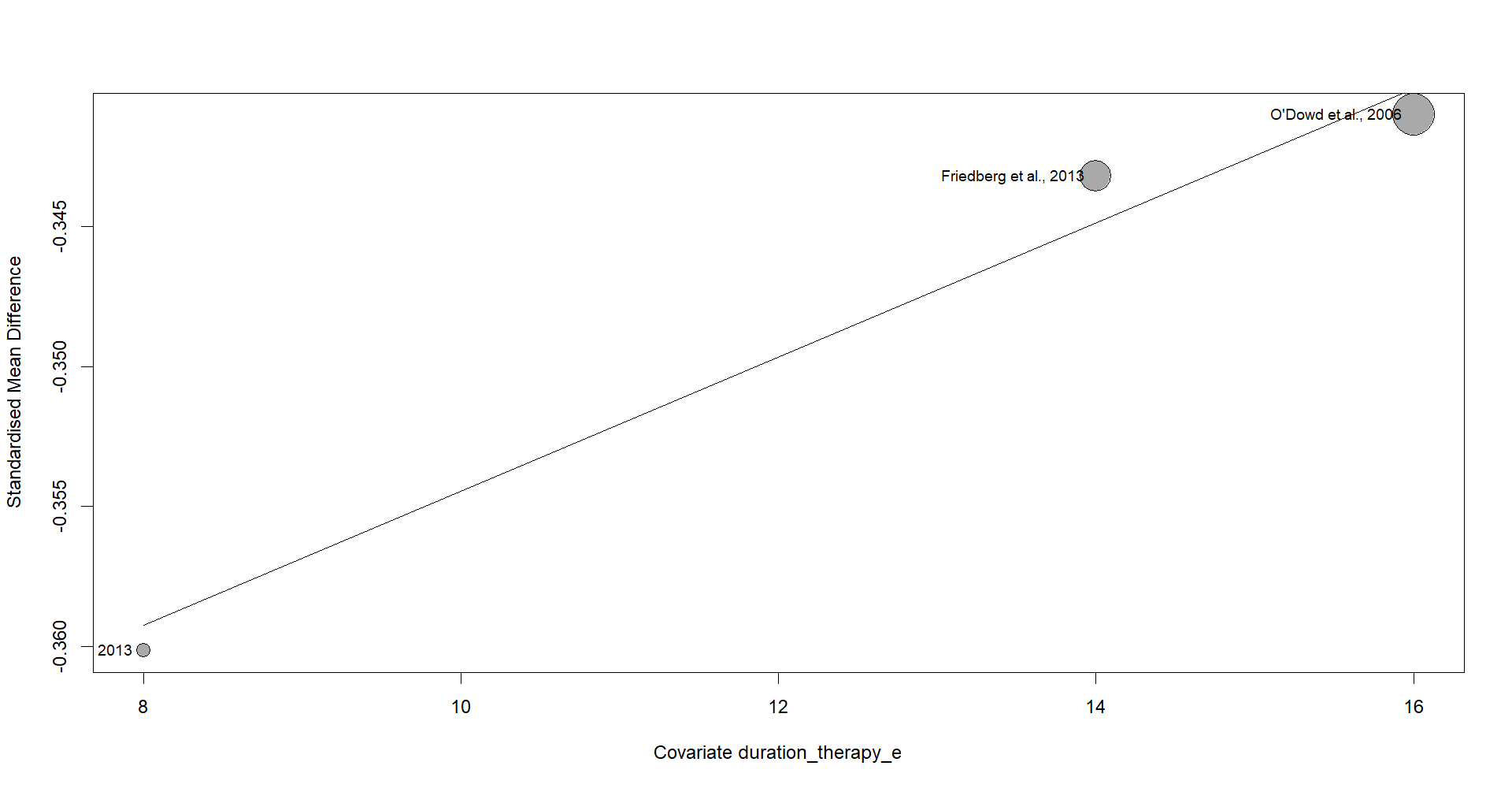


(D)


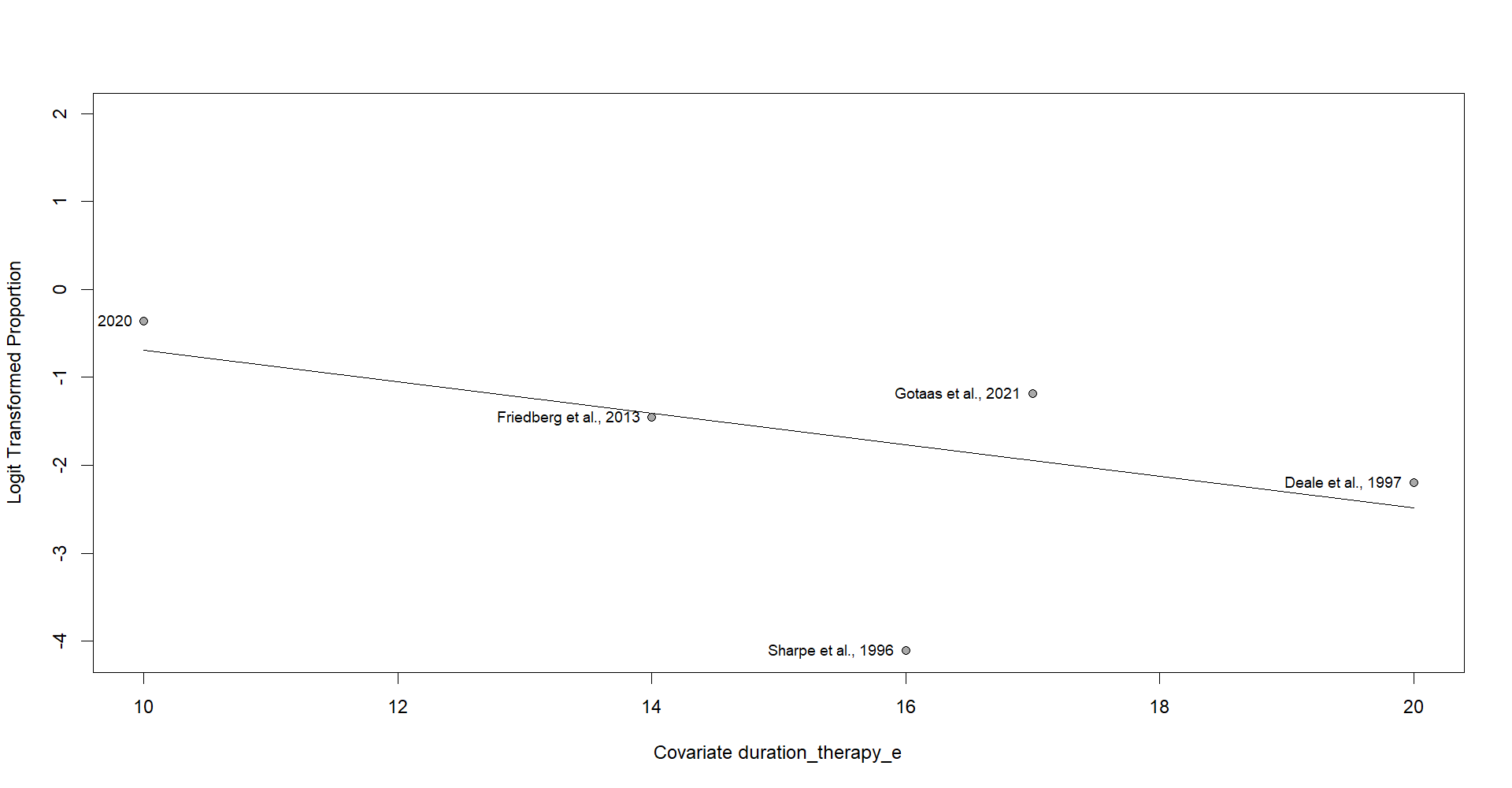


(E)


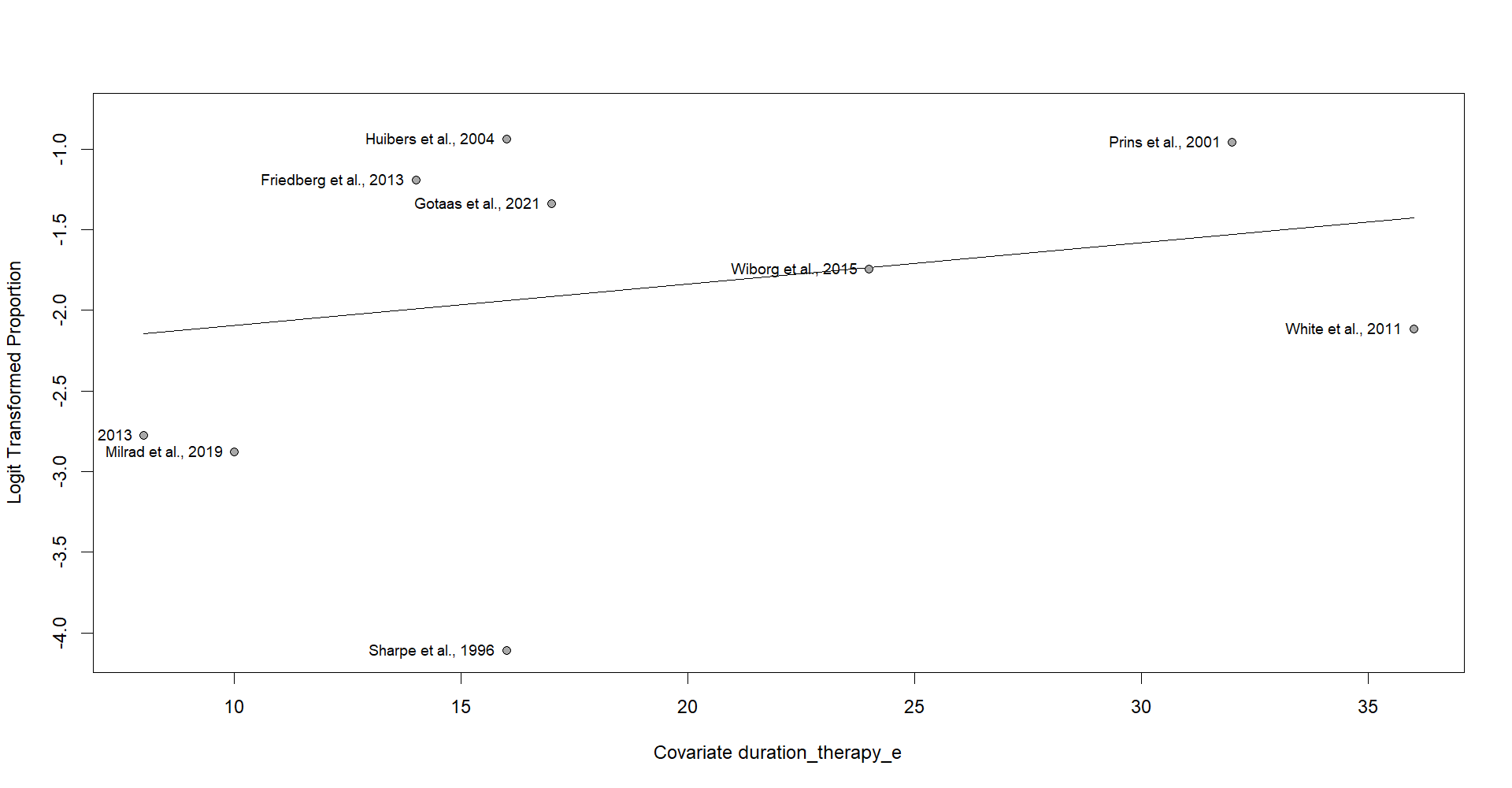


(F)


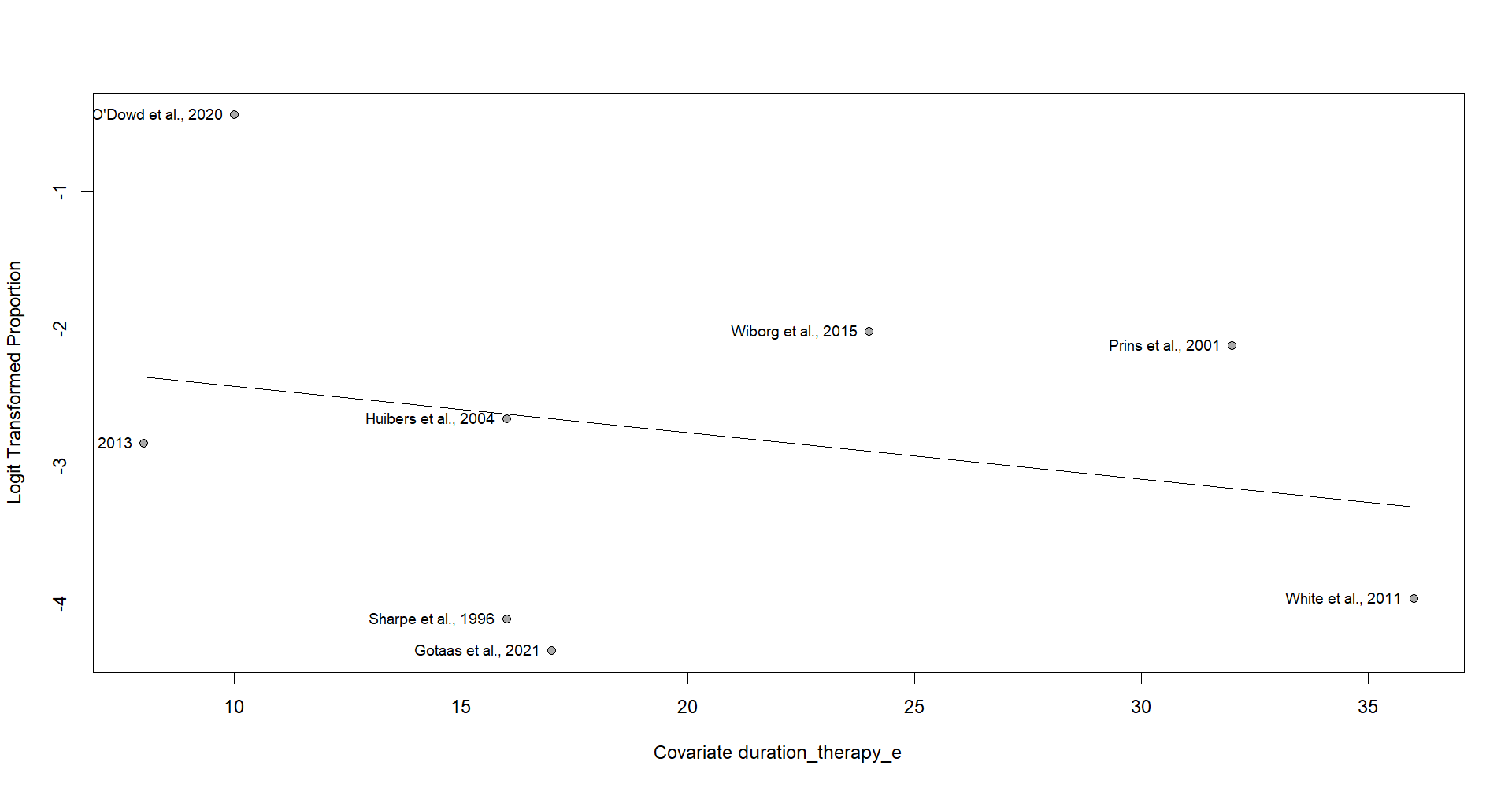


(G)


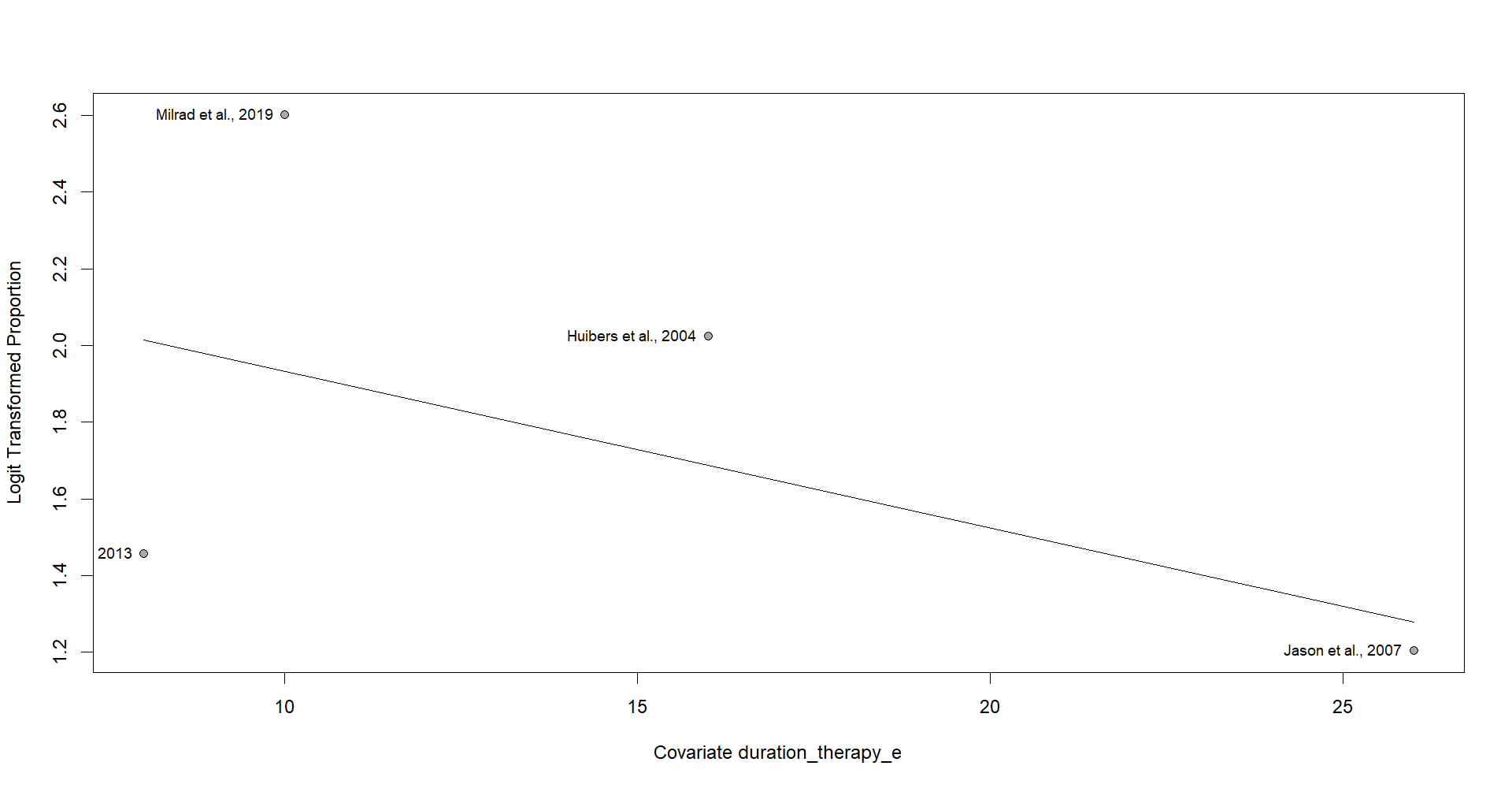


(H)

*Note.* A: bubble plot for the effect of duration of therapy on fatigue at post-treatment; B: bubble plot for the effect of duration of therapy on perceived health status at post-treatment; C: bubble plot for the effect of duration of therapy on depression at post-treatment; D: bubble plot for the effect of duration of therapy on anxiety at post-treatment; E: bubble plot for the effect of duration of therapy on non-completion; F: bubble plot for the effect of duration of therapy on drop-out; G: bubble plot for the effect of duration of therapy on treatment refusal; H: bubble plot for the effect of duration of therapy on the average proportion of sessions completed.

**Table S6**

*Meta-regression analyses for fatigue severity at baseline*

| Outcome | $\tau_{unexplained}^{2}$ | *I*² | $p_{moderator}$ | Regression weight | Intercept |
| --- | --- | --- | --- | --- | --- |
| Non-completion | 0.26 | 56.58% | 0.005 | 0.0988 | -3.13 |
| Drop-out | 0.17 | 64.30% | 0.02 | 0.0290 | -2.60 |
| Ave. prop. sessions | 0 | 0.00% | 0.95 | 0.002 | 1.62 |

*Note.* $\tau_{unexplained}^{2}$ = estimated amount of residual heterogeneity; *I*² = residual heterogeneity/unaccounted variability

**Figure S12**

*Bubble plots – meta-regression analysis for fatigue severity*


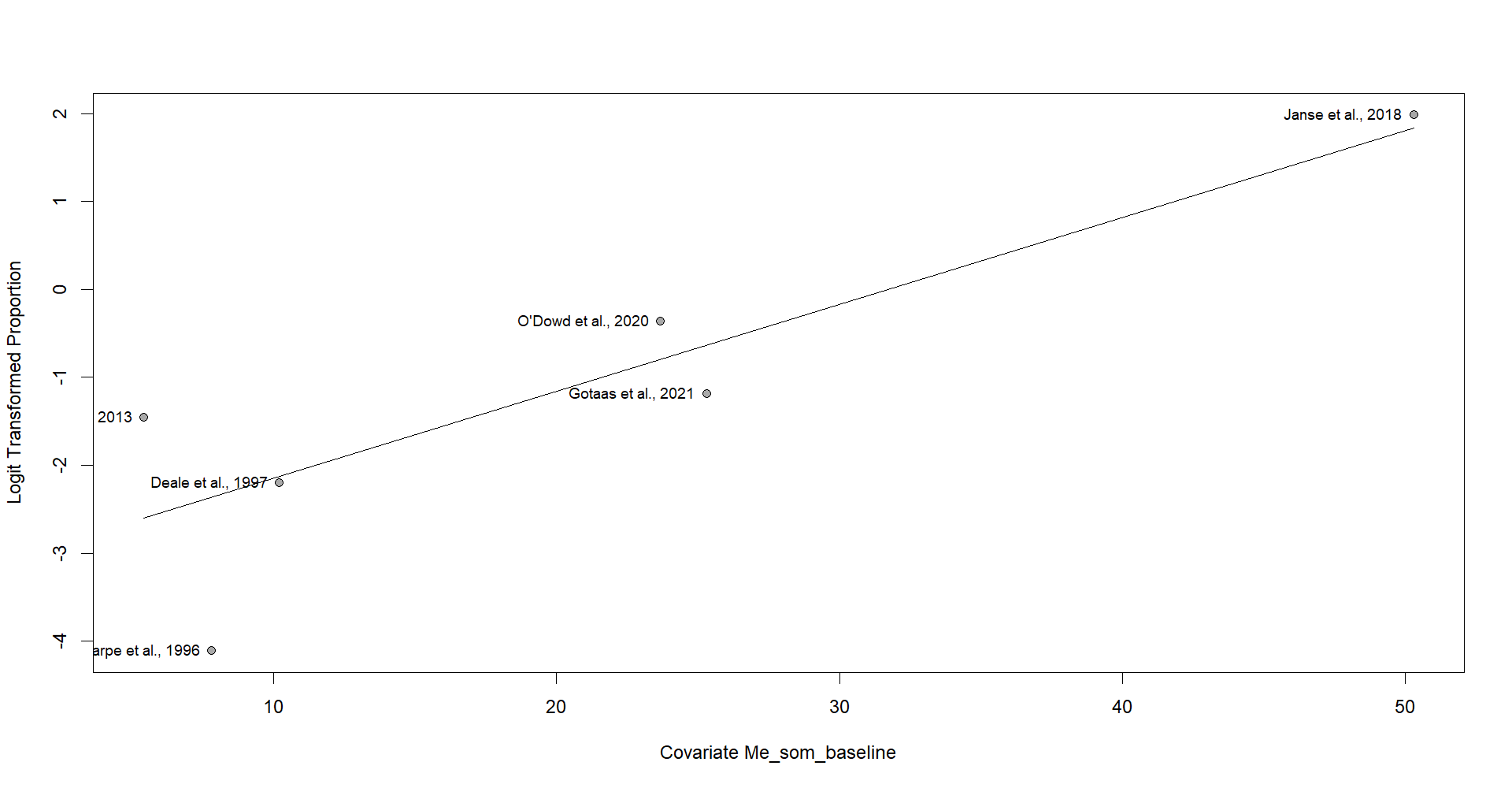


(A)


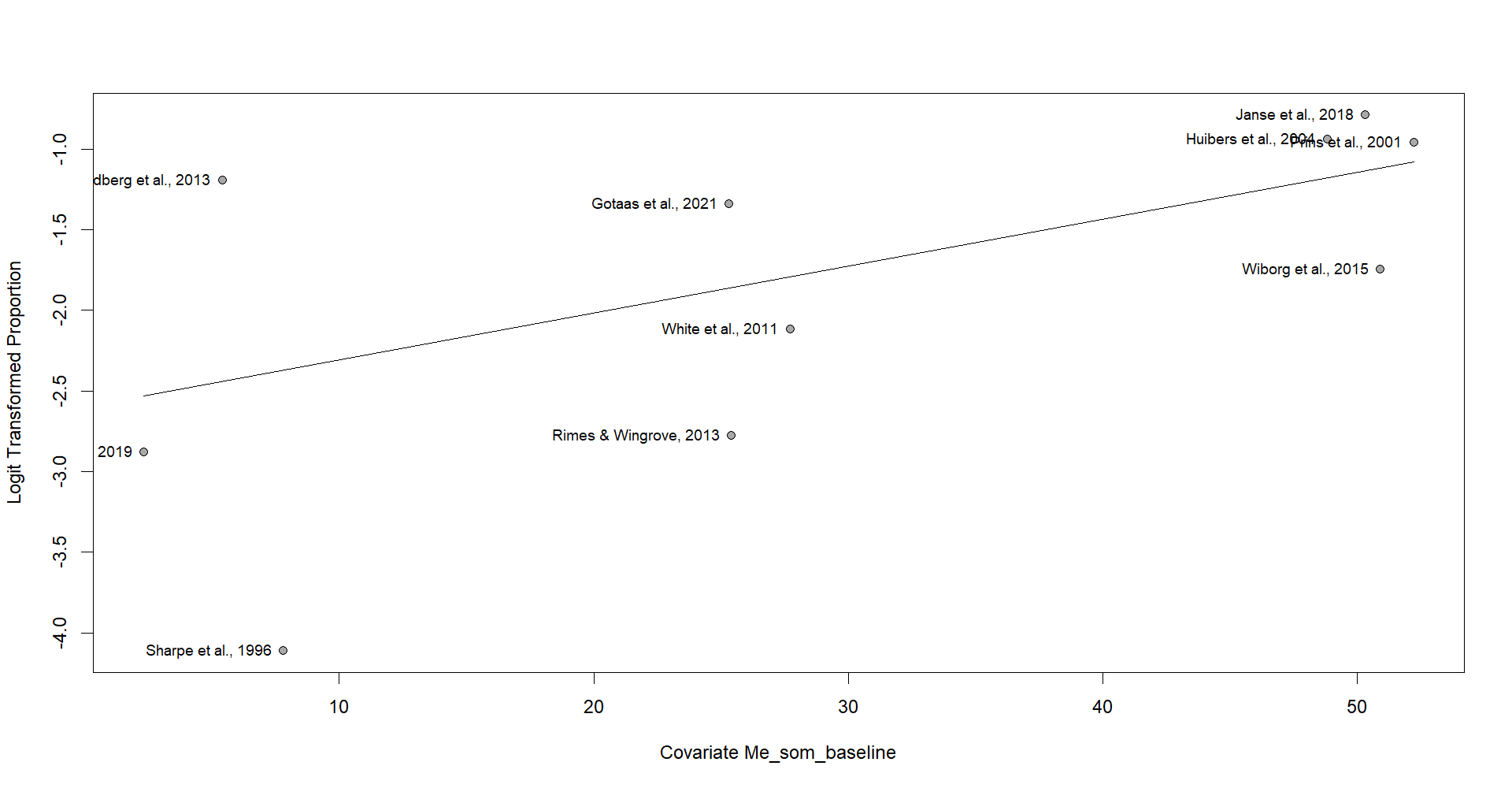


(B)


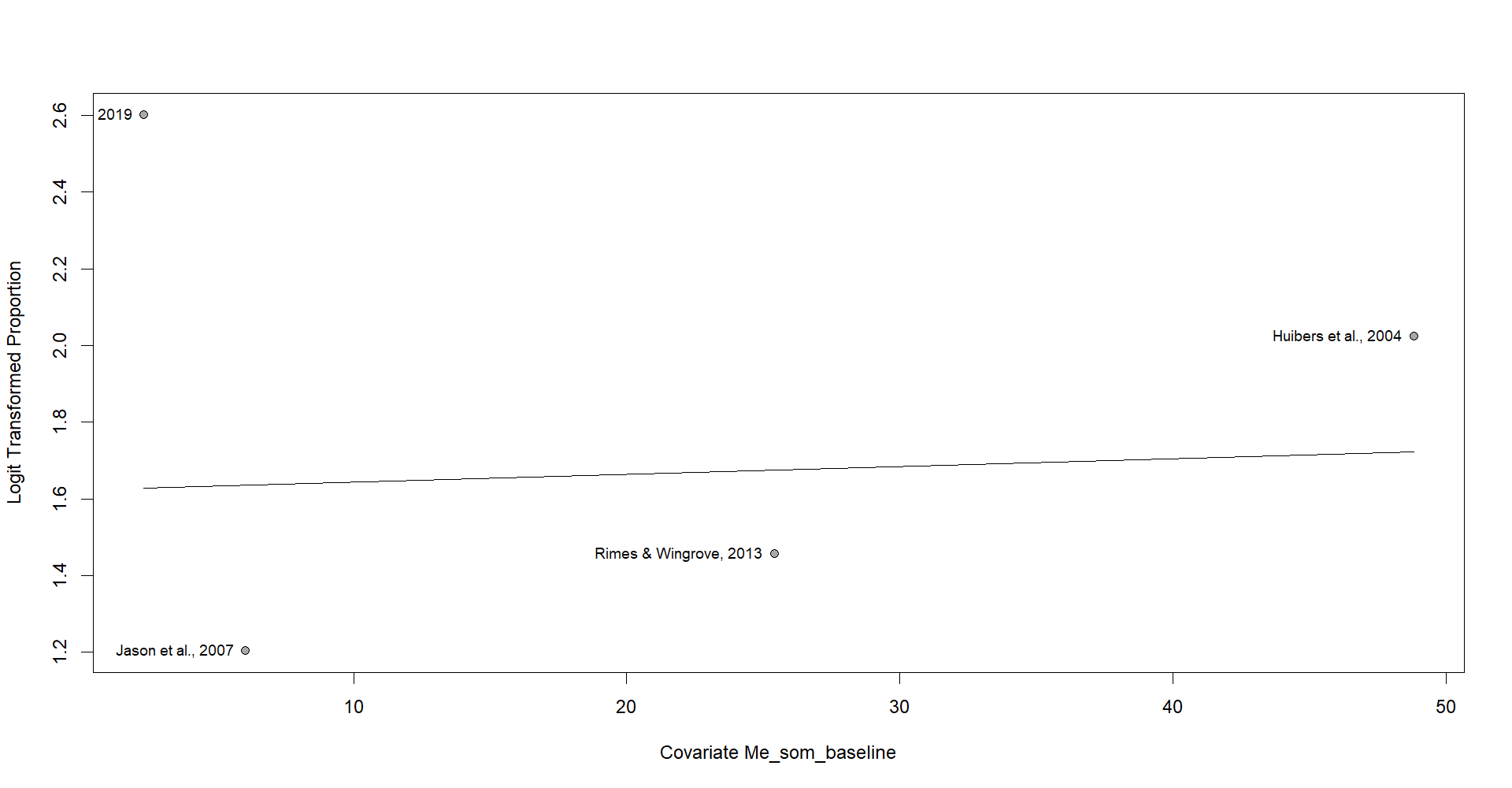


(C)

*Note.* A: bubble plot for the effect of fatigue severity on non-completion; B: bubble plot for the effect of fatigue severity on drop-out; C: bubble plot for the effect of fatigue severity on the average proportion of sessions completed.

**Table S7**

*Meta-regression analyses for duration of fatigue symptoms up to baseline*

| Outcome | $\tau_{unexplained}^{2}$ | *I*² | $p_{moderator}$ | Regression weight | Intercept |
| --- | --- | --- | --- | --- | --- |
| Non-completion | 0.43 | 58.04% | 0.37 | 0.0123 | -2.90 |
| Drop-out | 0.32 | 74.72% | 0.76 | 0.0022 | -1.80 |

*Note.* $\tau_{unexplained}^{2}$ = estimated amount of residual heterogeneity; *I*² = residual heterogeneity/unaccounted variability; only *k* = 2 studies reported information on the average proportion of sessions completed and the duration of symptoms

**Figure S13**


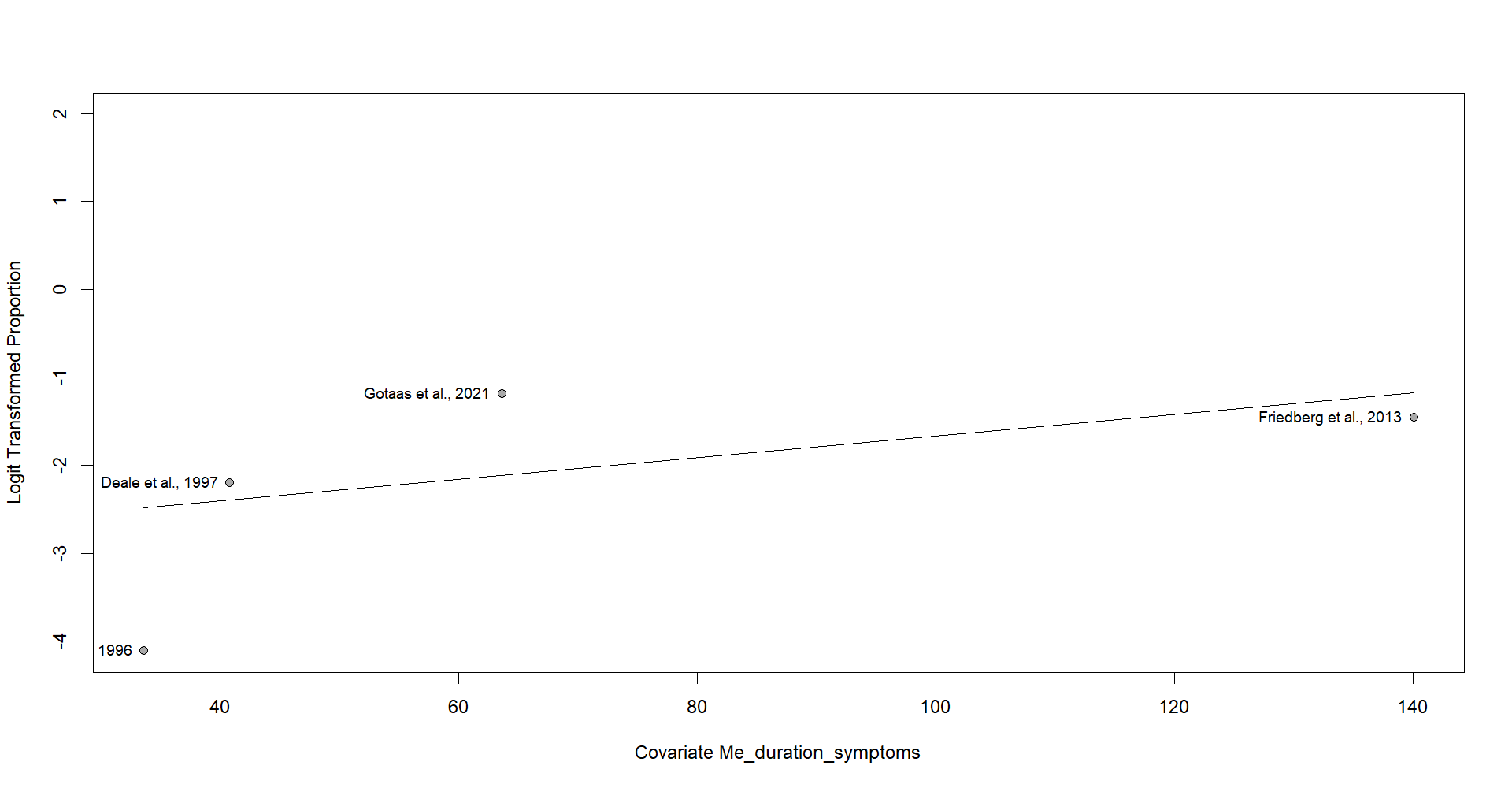


(A)


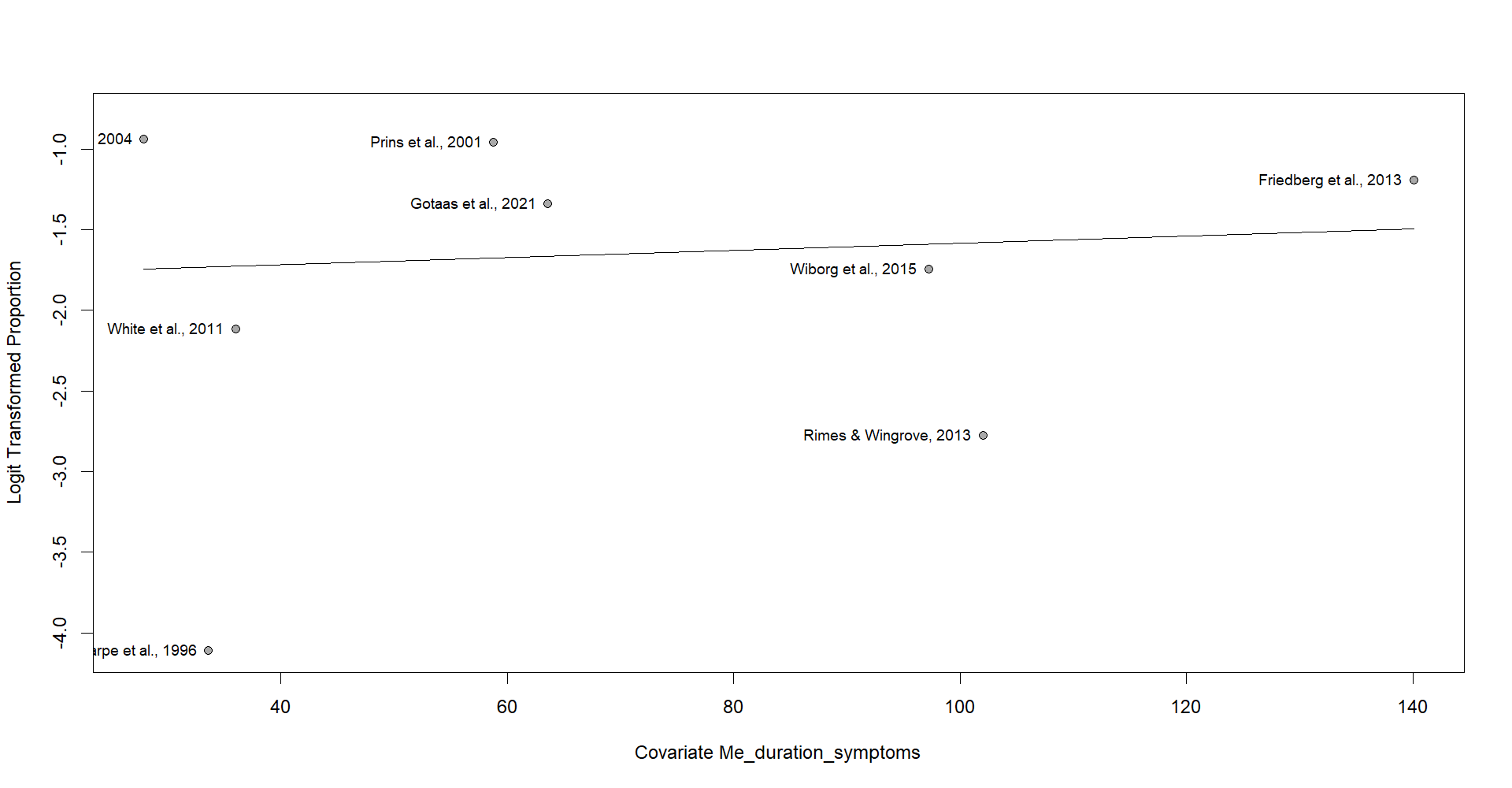


(B)

*Note.* A: bubble plot for the effect of duration of fatigue symptoms on non-completion; B: bubble plot for the effect of duration of fatigue symptoms on drop-out.

# **Results Sensitivity Analyses**

**Table S8**

*Sensitivity analyses comparing fixed effect and random effects models*

| Outcome | *k* | Model | Pooled effect^a^ | 95%CI |
| --- | --- | --- | --- | --- |
| Fatigue | 11 | Random effects | -0.52 | -0.69; -0.35 |
|  |  | Fixed effect | -0.52 | -0.62; -0.42 |
| Perceived health status | 11 | Random effects | 0.29 | 0.11; 0.47 |
|  |  | Fixed effect | 0.28 | 0.18; 0.38 |
| Depression | 5 | Random effects | -0.36 | -0.55; -0.17 |
|  |  | Fixed effect | -0.36 | -0.55; -0.17 |
| Anxiety | 3 | Random effects | -0.34 | -0.62; -0.07 |
|  |  | Fixed effect | -0.34 | -0.62; -0.07 |
| Non-completion | 6 | Random effects | 0.22 | 0.03; 0.71 |
|  |  | Fixed effect | 0.49 | 0.44; 0.54 |
| Drop-out | 10 | Random effects | 0.15 | 0.09; 0.25 |
|  |  | Fixed effect | 0.19 | 0.16; 0.22 |
| Treatment refusal | 10 | Random effects | 0.07 | 0.03; 0.15 |
|  |  | Fixed effect | 0.09 | 0.07; 0.11 |
| Average proportion of sessions completed | 4 | Random effects | 0.84 | 0.56; 0.96 |
|  |  | Fixed effect | 0.84 | 0.69; 0.93 |

*Note.* Effect on efficacy outcomes at post-treatment; *k* = number of studies.

^a^ Hedges’ *g* for efficacy outcomes, proportions for acceptance outcomes

**Table S9**

*“Imputed” starters*

| Outcome | Sample | *k* | Proportion | 95%CI^a^ | *I*² | 95%CI^b^ |
| --- | --- | --- | --- | --- | --- | --- |
| Non-completion | With imputation | 6 | 0.22 | 0.03; 0.71 | 95.4% | 92.2; 97.2 |
|  | Without imputation | 4 | 0.26 | 0.01; 0.96 | 96.1% | 92.7; 97.9 |
| Drop-out | With imputation | 10 | 0.15 | 0.09; 0.25 | 75.7% | 54.9; 86.9 |
|  | Without imputation | 8 | 0.17 | 0.09; 0.28 | 74.3% | 47.9; 87.3 |

^a^ 95%CI for the proportion, ^b^ 95%CI for *I*²

**Table S10**

*Outlier analyses*

|  | *k* | Pooled effect^a^ | 95%CI^b^ | *I*² | 95%CI^c^ | 95%PI |
| --- | --- | --- | --- | --- | --- | --- |
| Fatigue |  |  |  |  |  |  |
| With outliers | 11 | -0.52 | -0.69; -0.35 | 64.4% | 32.1; 81.3 | -1.07; 0.04 |
| Without outliers | 10 | -0.46 | -0.59; -0.32 | 37.2% | 0.0; 70.1 | -0.80; -0.12 |
| Perceived Health Status |  |  |  |  |  |  |
| With outliers | 11 | 0.29 | 0.11; 0.47 | 65.3% | 34.2; 81.8 | -0.30; 0.88 |
| Without outliers | 10 | 0.35 | 0.21; 0.48 | 38.9% | 0.0; 70.9 | 0.01; 0.69 |
| Non-completion |  |  |  |  |  |  |
| With outliers | 6 | 0.22 | 0.03; 0.71 | 95.4% | 92.2; 97.2 | 0.00; 0.99 |
| Without outliers | 5 | 0.15 | 0.04; 0.42 | 33.1% | 0.0; 74.6 | 0.01; 0.85 |
| Treatment refusal |  |  |  |  |  |  |
| With outliers | 10 | 0.07 | 0.03; 0.15 | 80.3% | 64.6; 89.0 | 0.01; 0.52 |
| Without outliers | 9 | 0.06 | 0.03; 0.12 | 70.4% | 41.2; 85.1 | 0.01; 0.34 |

^a^ Hedges’ *g* for efficacy outcomes, proportions for acceptance outcomes, ^b^ 95%CI for the proportion, ^c^ 95%CI for *I*²

**Figure S14**

*Results for the influence analyses for fatigue at post-treatment*


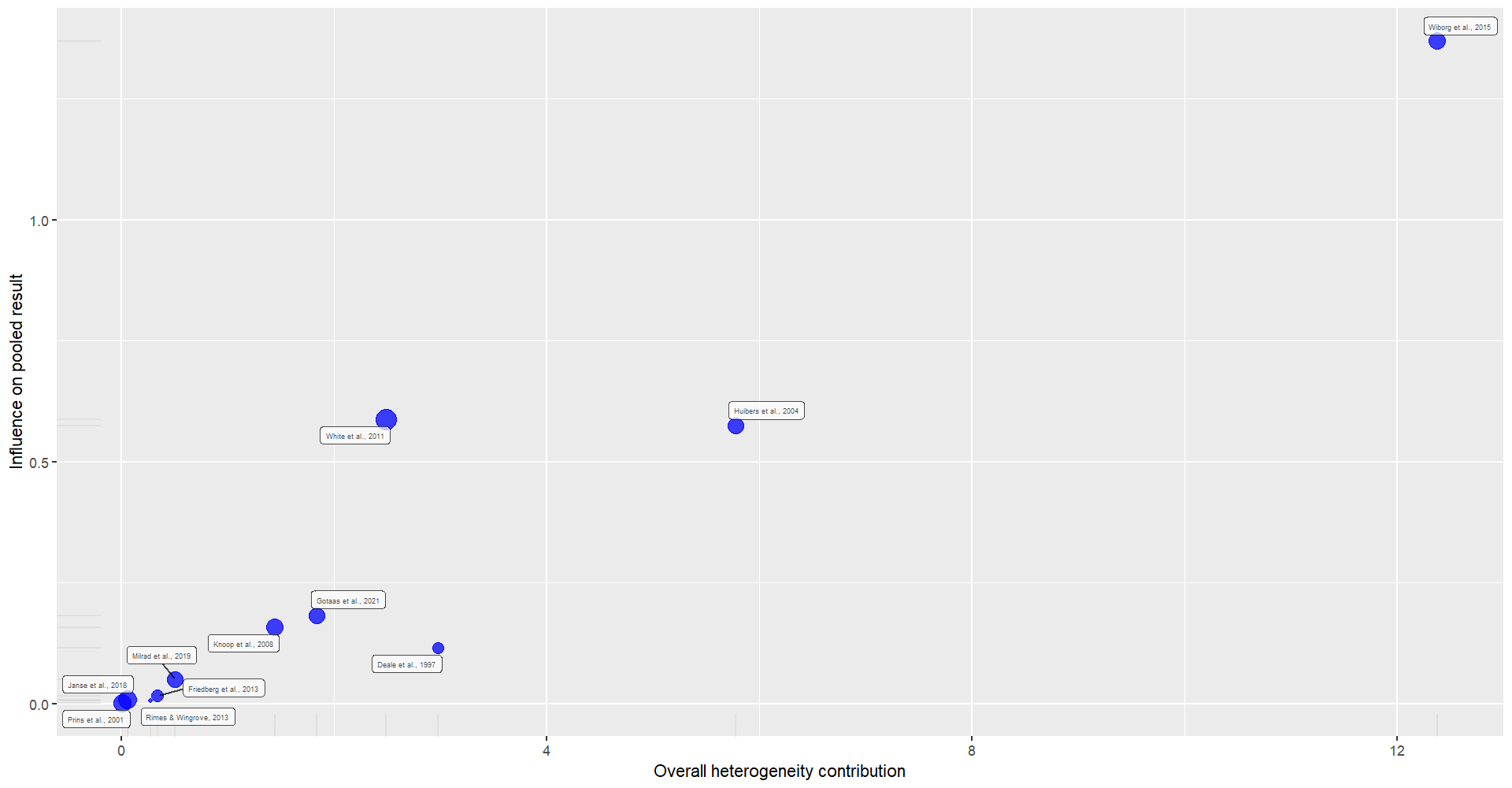


(A)


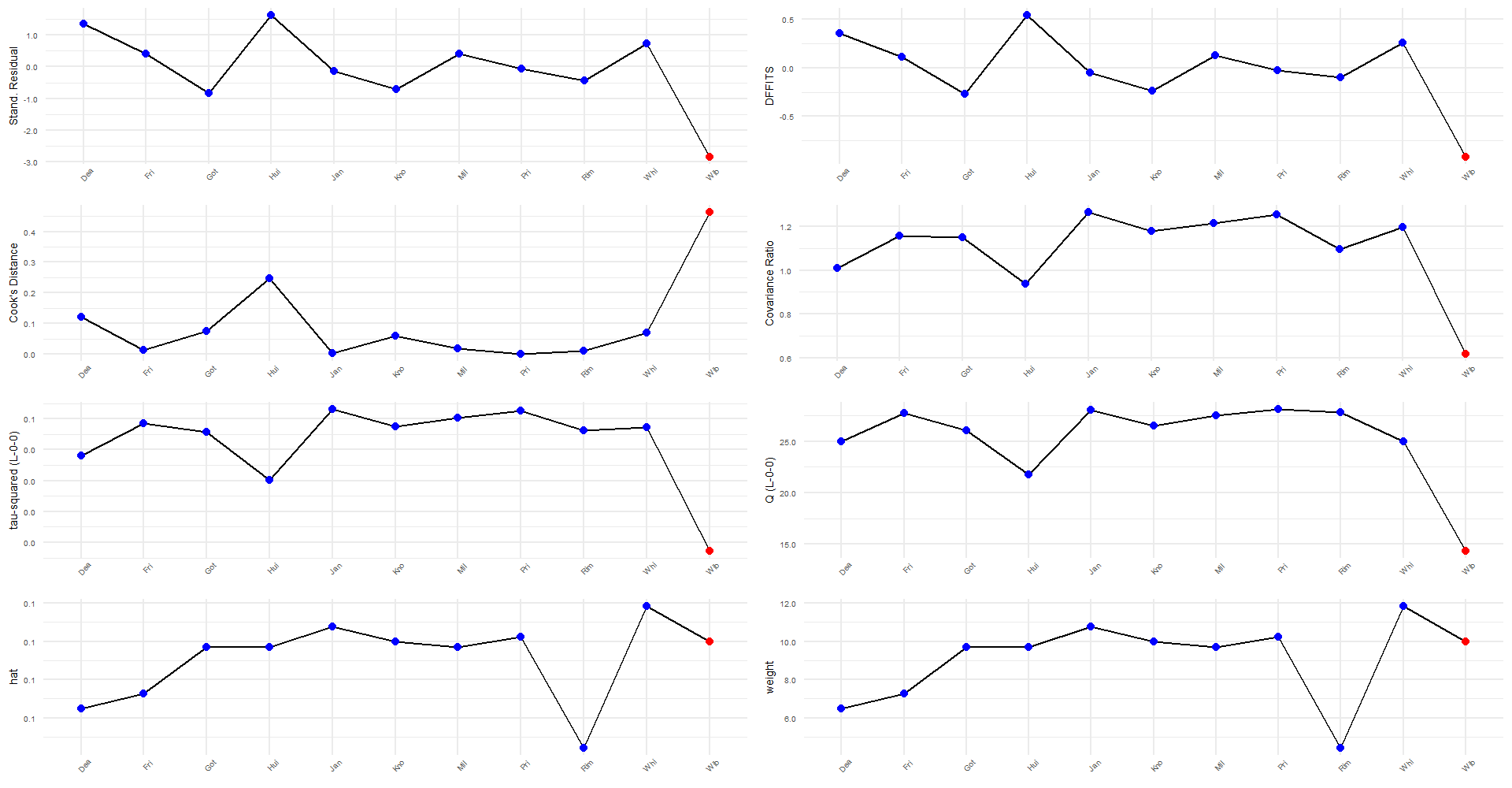


(B)


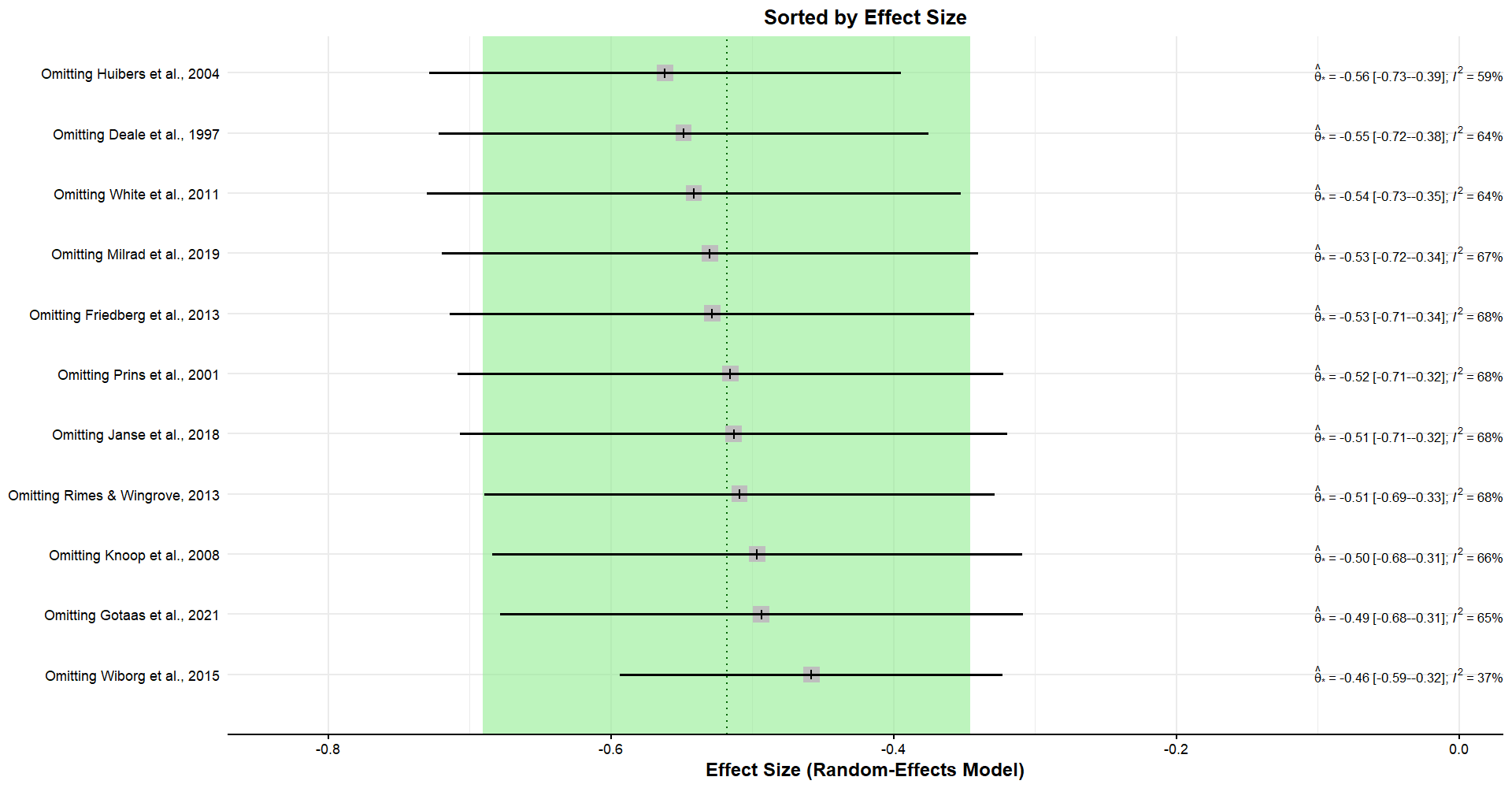


(C)


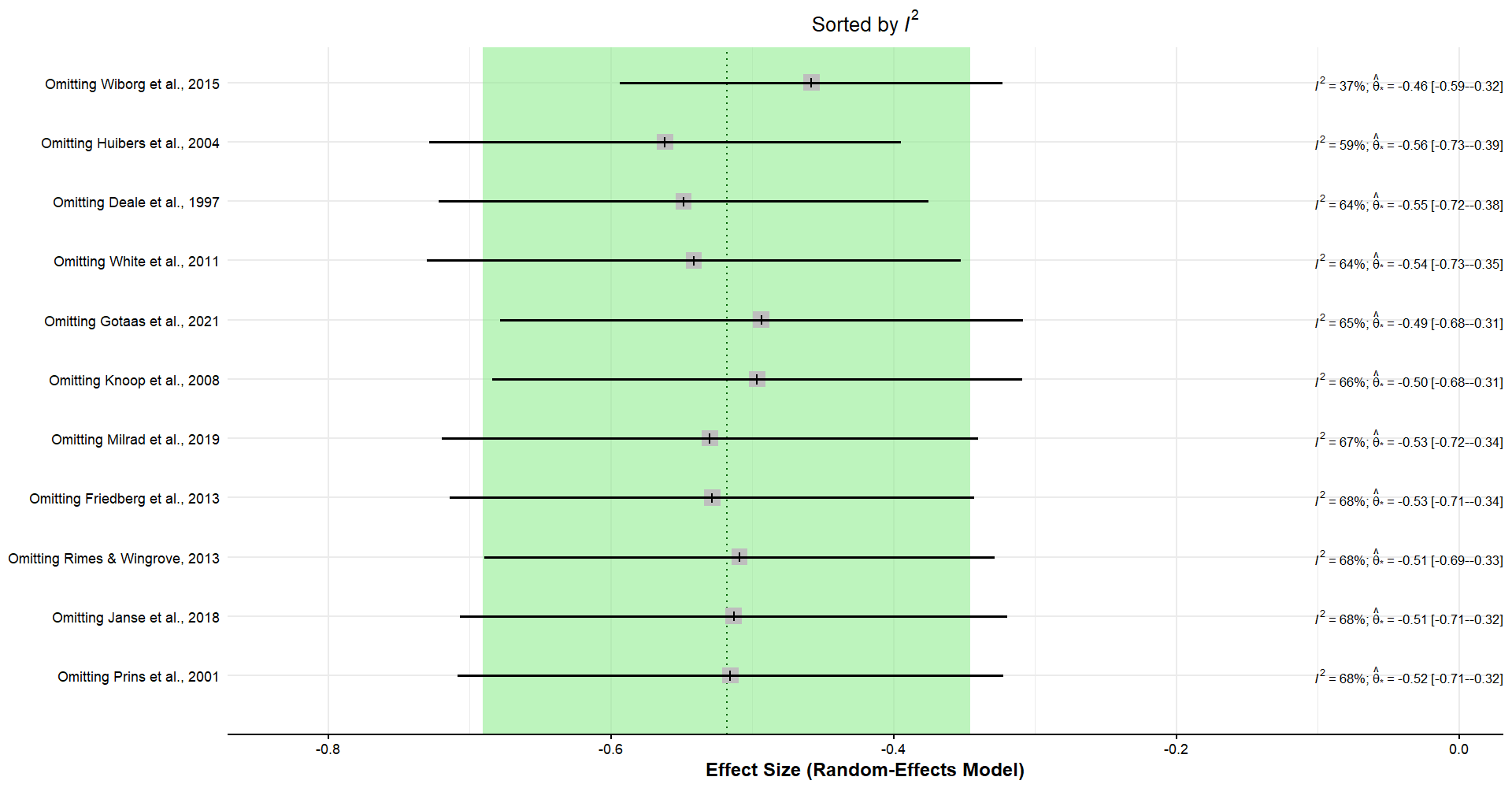


(D)


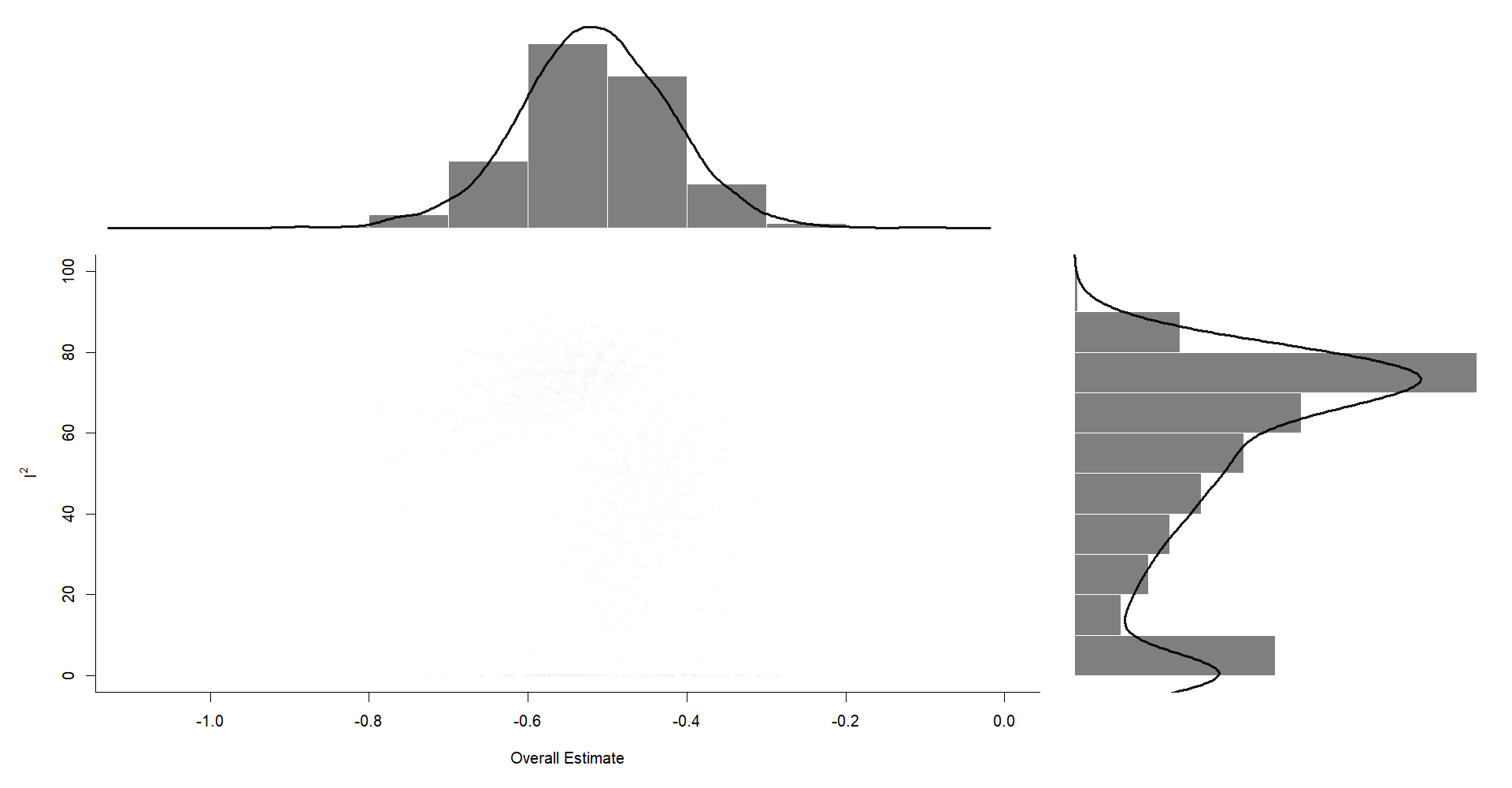


(E)

*Note.* A: Baujat plot; B: influence plot; C: leave one out method, influence on effect size; D: leave one out method, influence on heterogeneity; E: Graphic Display of Heterogeneity (GOSH) plot.

**Figure S15**

*Results for the influence analyses for perceived health status at post-treatment*


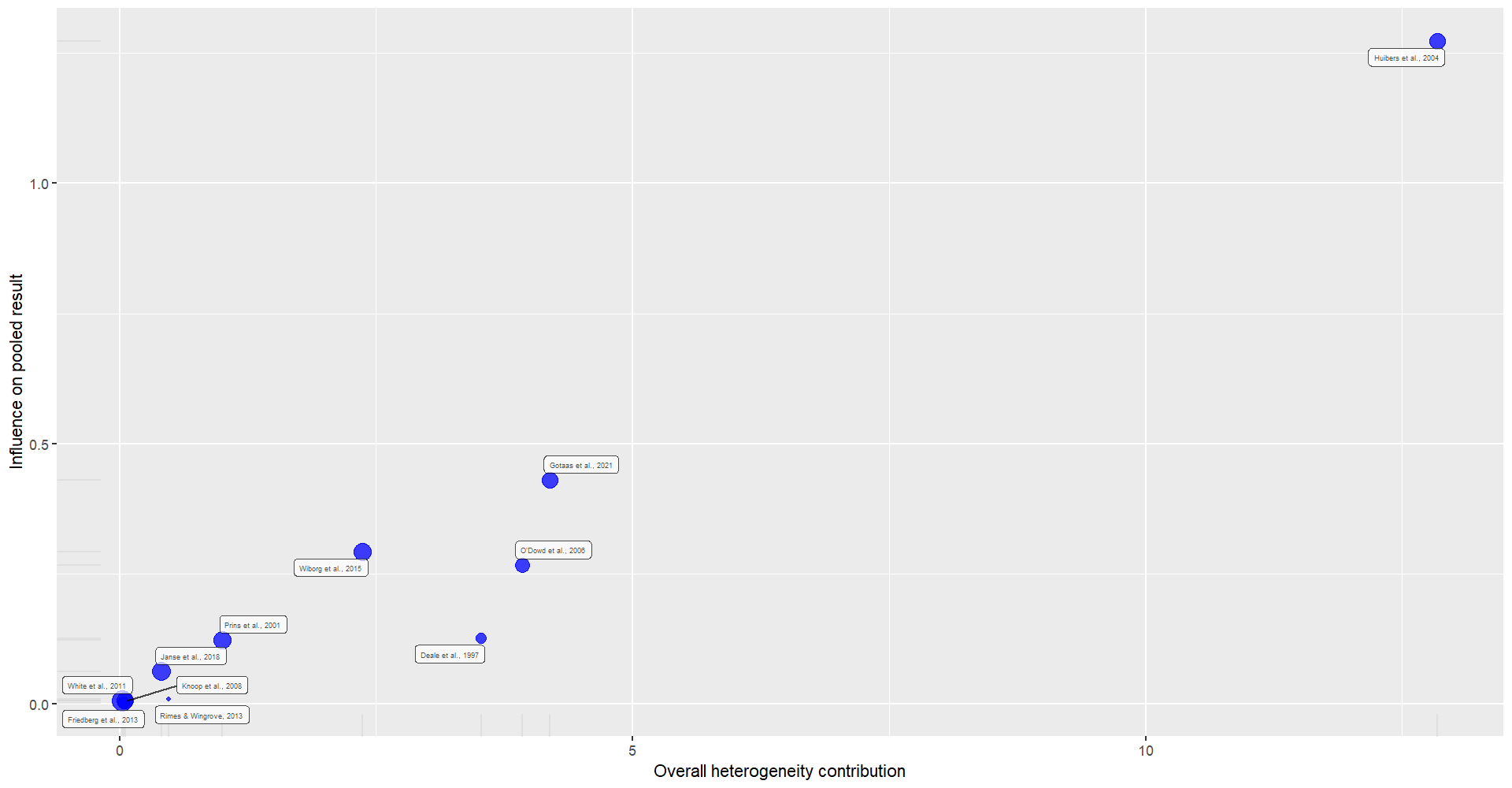


(A)


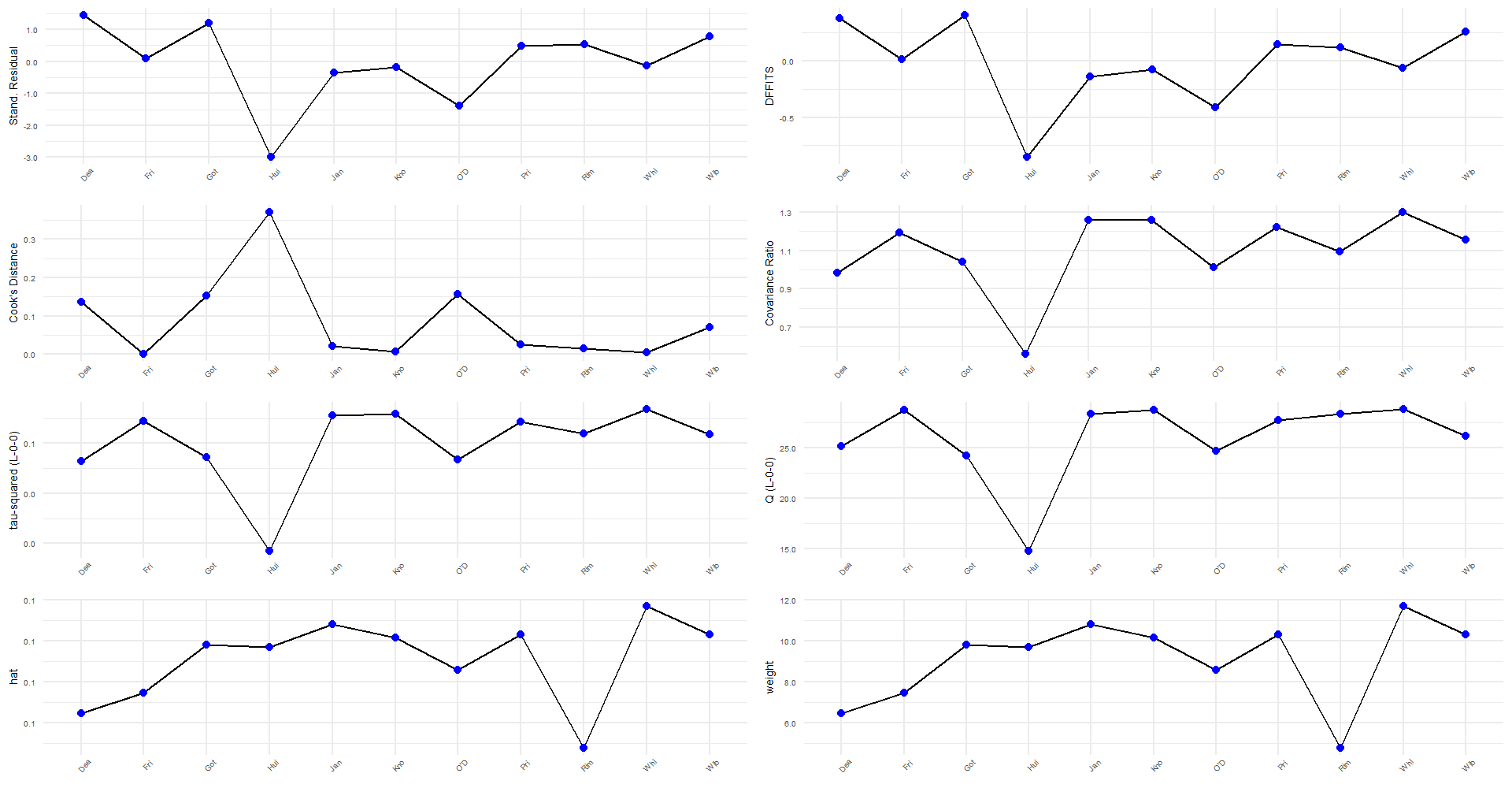


(B)


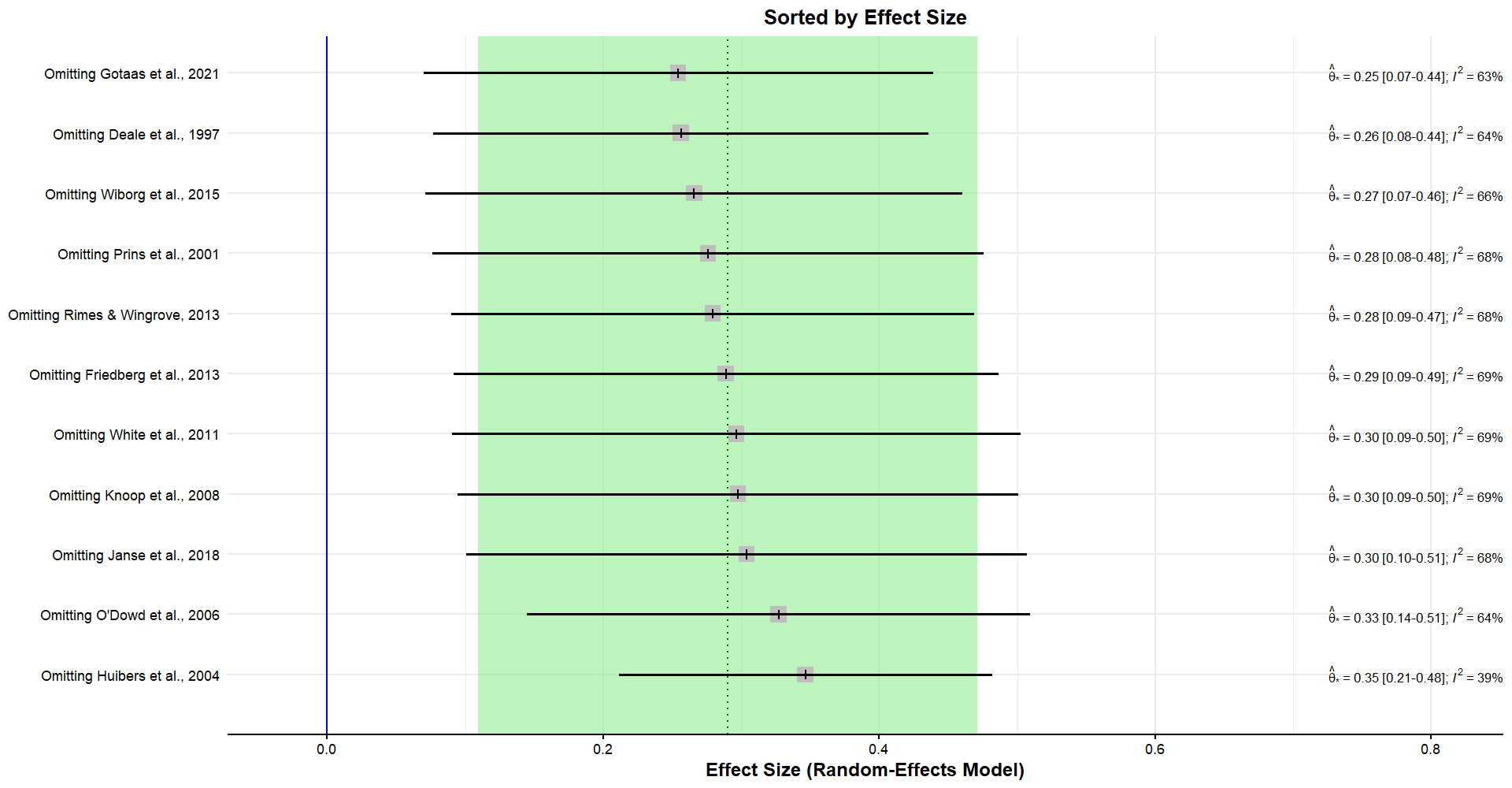


(C)


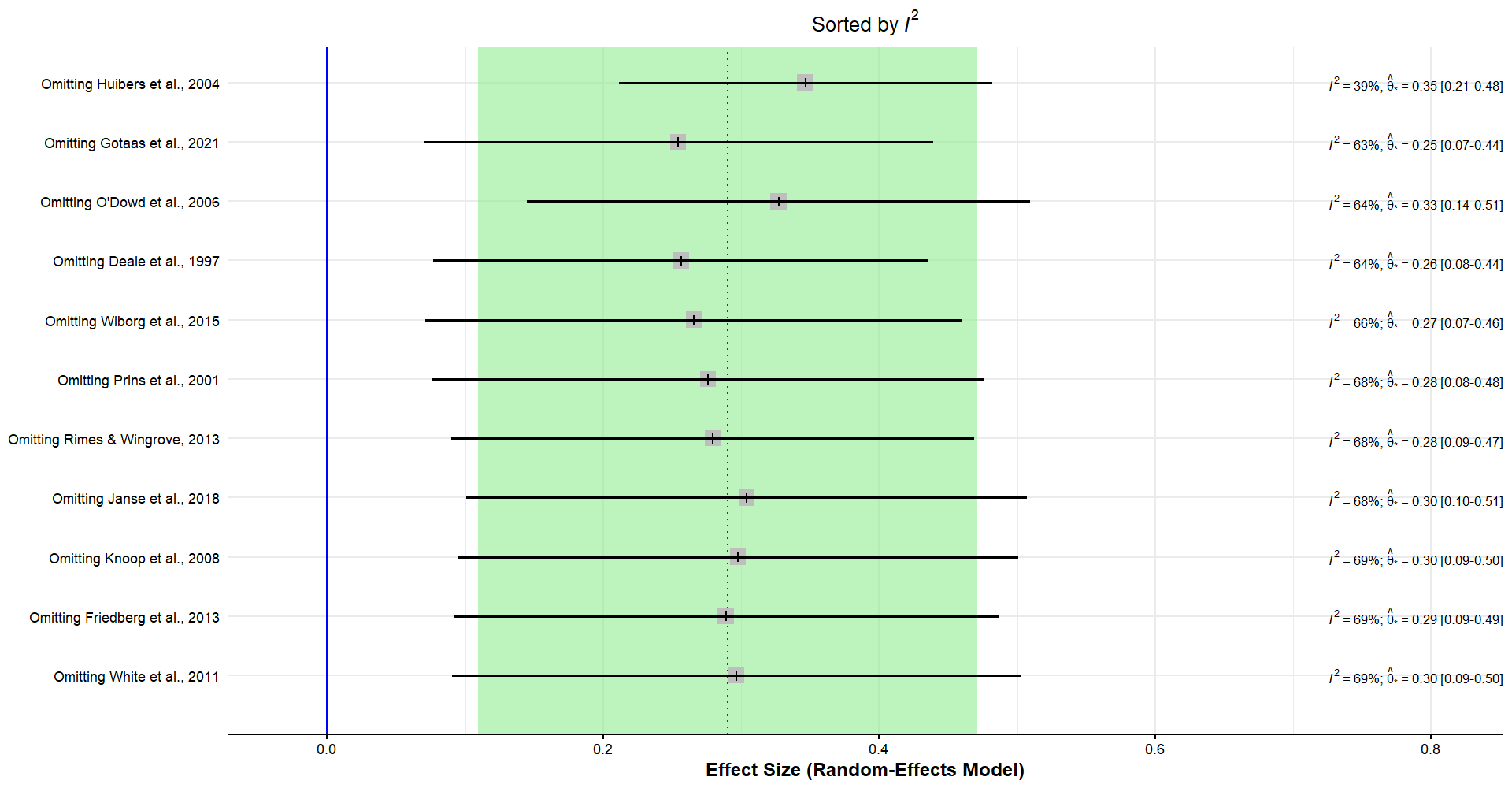


(D)


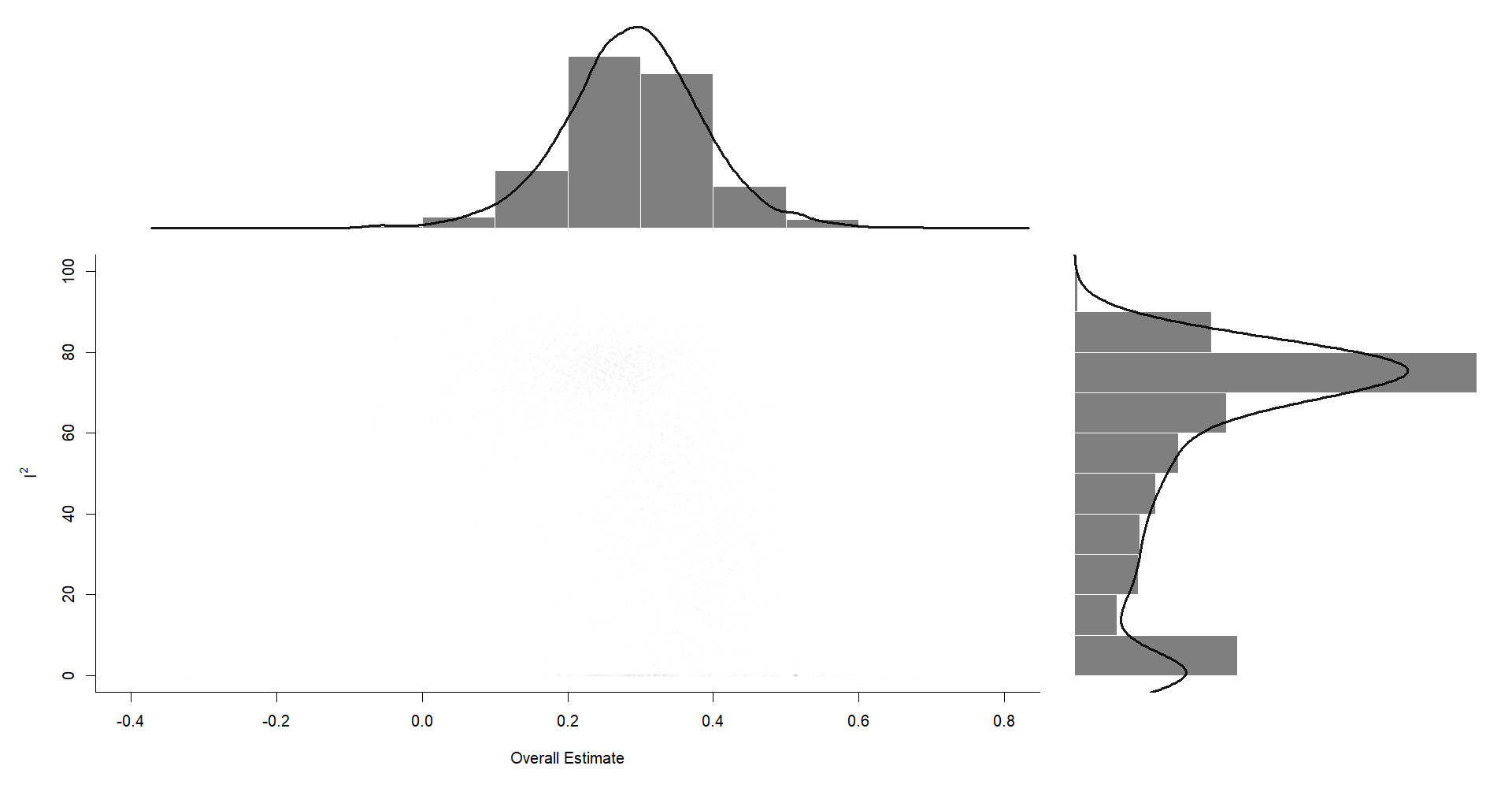


(E)

*Note.* A: Baujat plot; B: influence plot; C: leave one out method, influence on effect size; D: leave one out method, influence on heterogeneity; E: Graphic Display of Heterogeneity (GOSH) plot.

**Figure S16**

*Results for the influence analyses for depression at post-treatment*


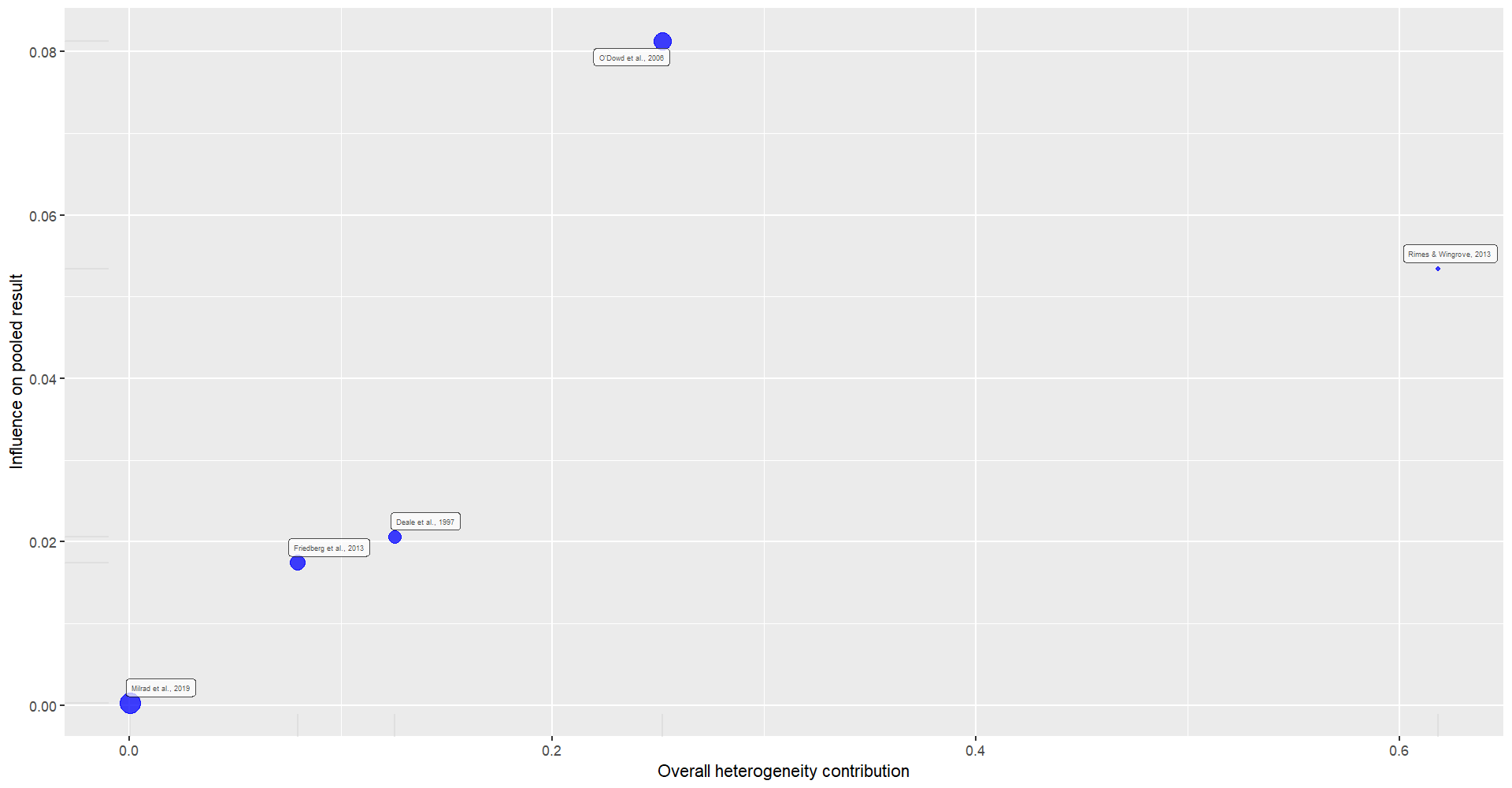


(A)


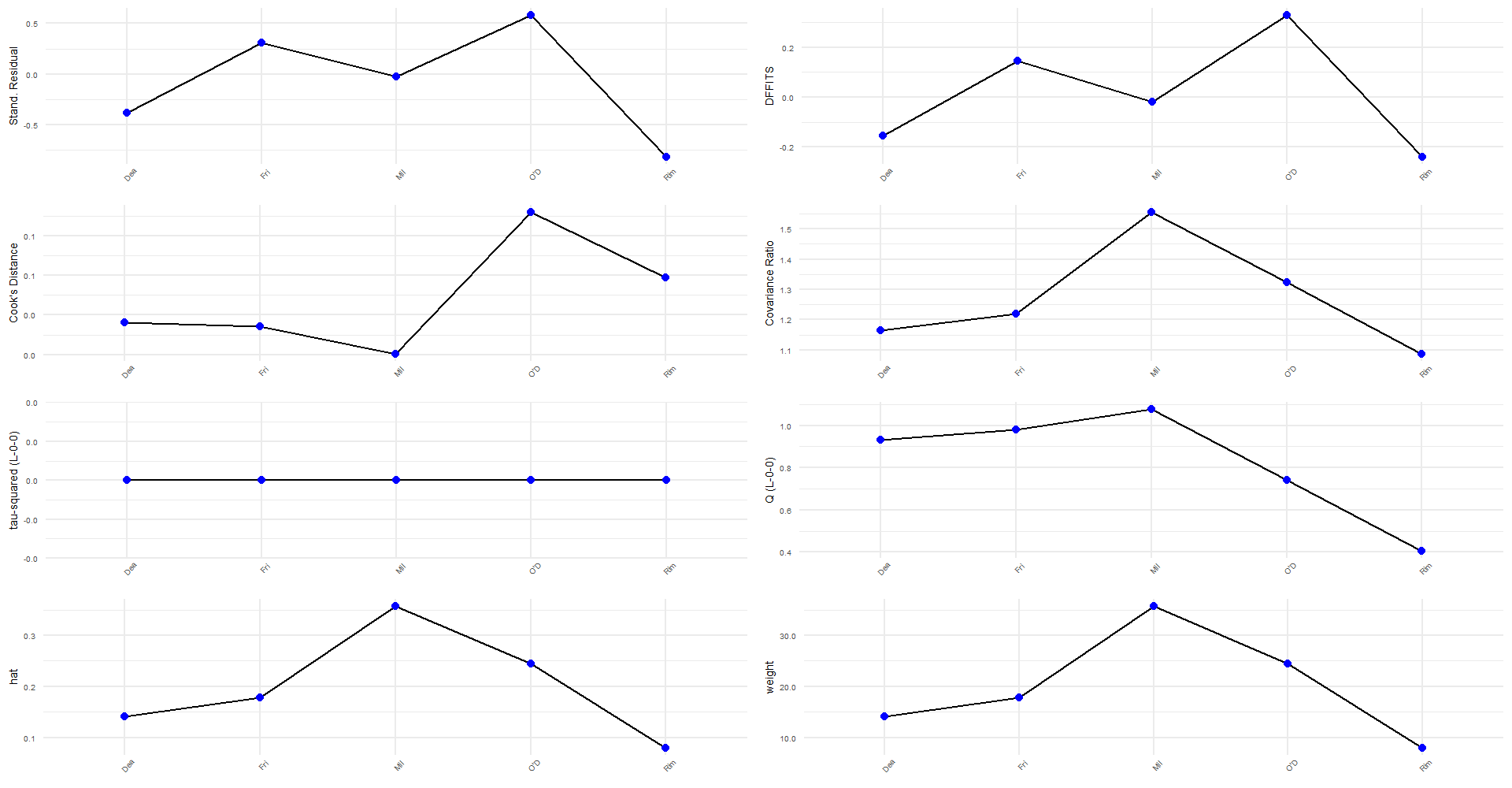


(B)


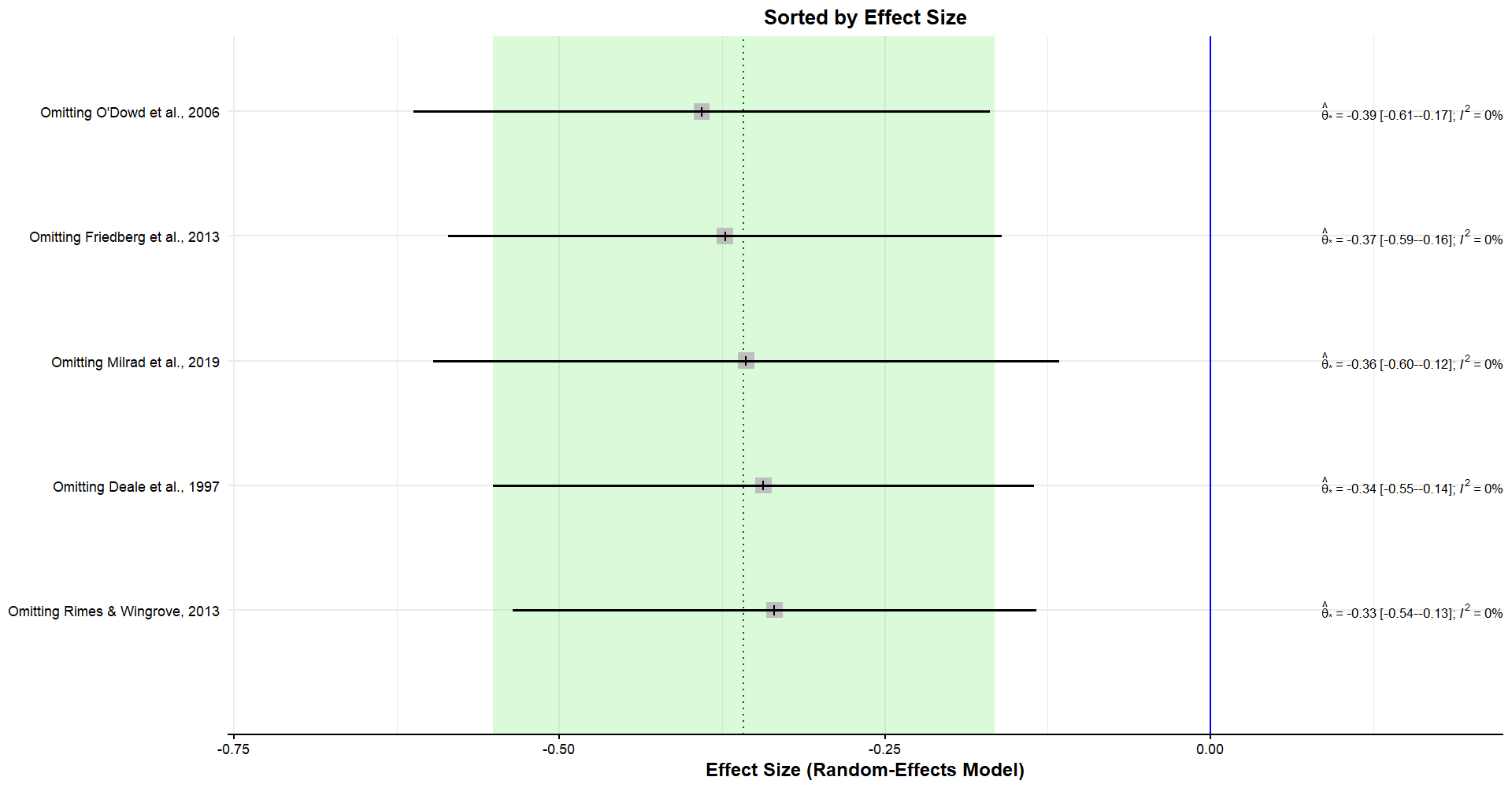


(C)


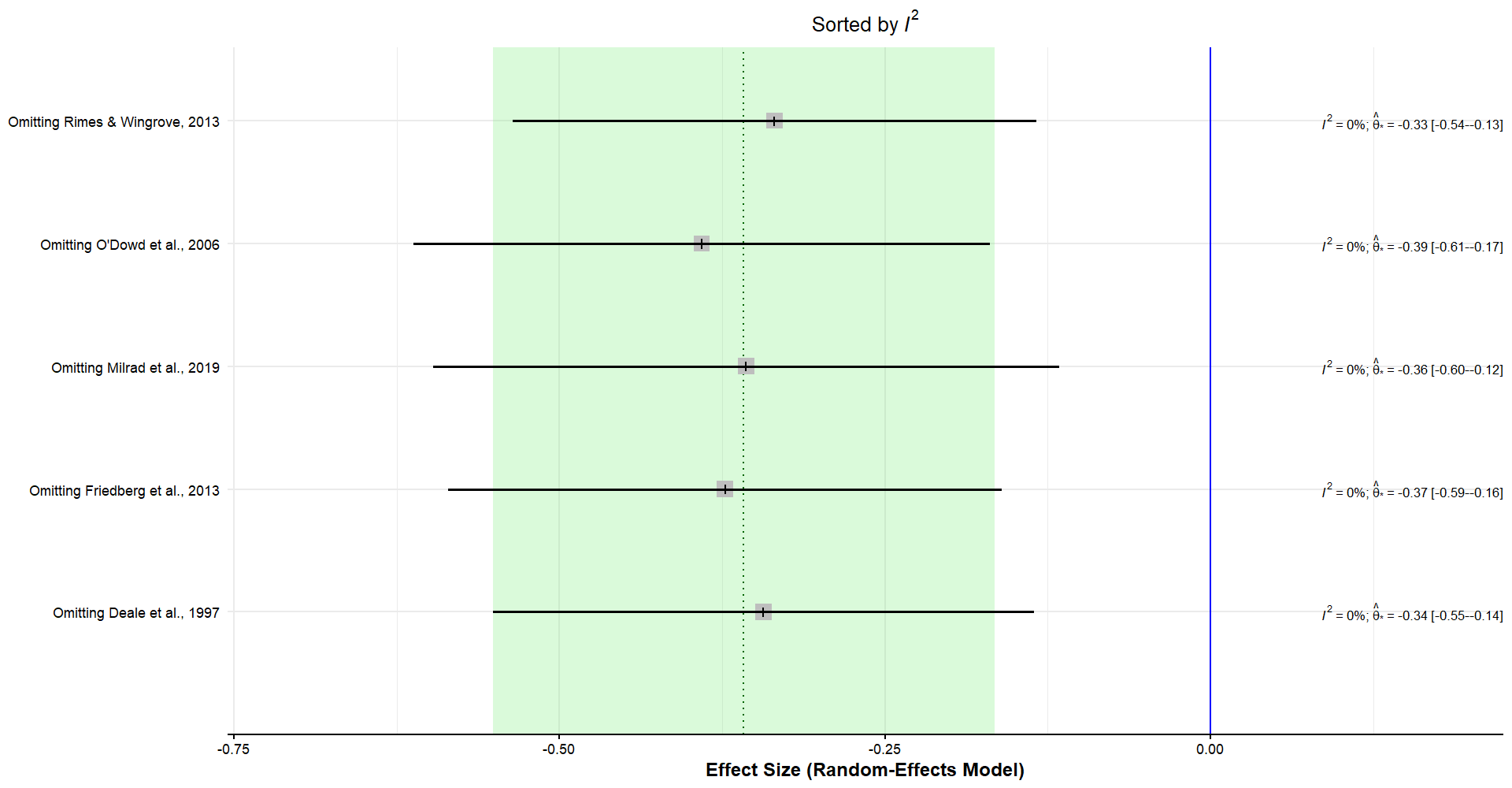


(D)


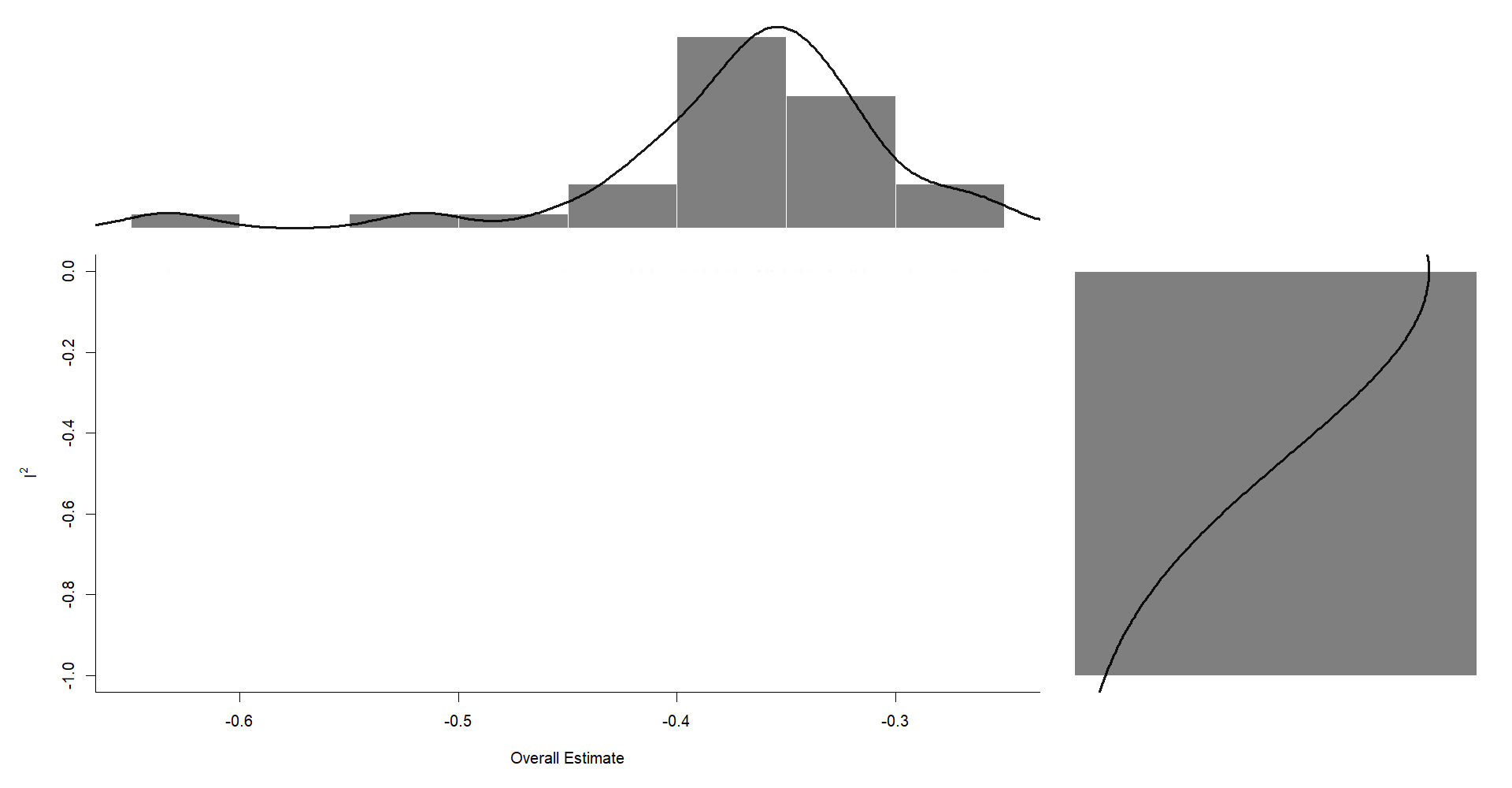


(E)

*Note.* A: Baujat plot; B: influence plot; C: leave one out method, influence on effect size; D: leave one out method, influence on heterogeneity; E: Graphic Display of Heterogeneity (GOSH) plot.

**Figure S17**

*Results for the influence analyses for anxiety at post-treatment*


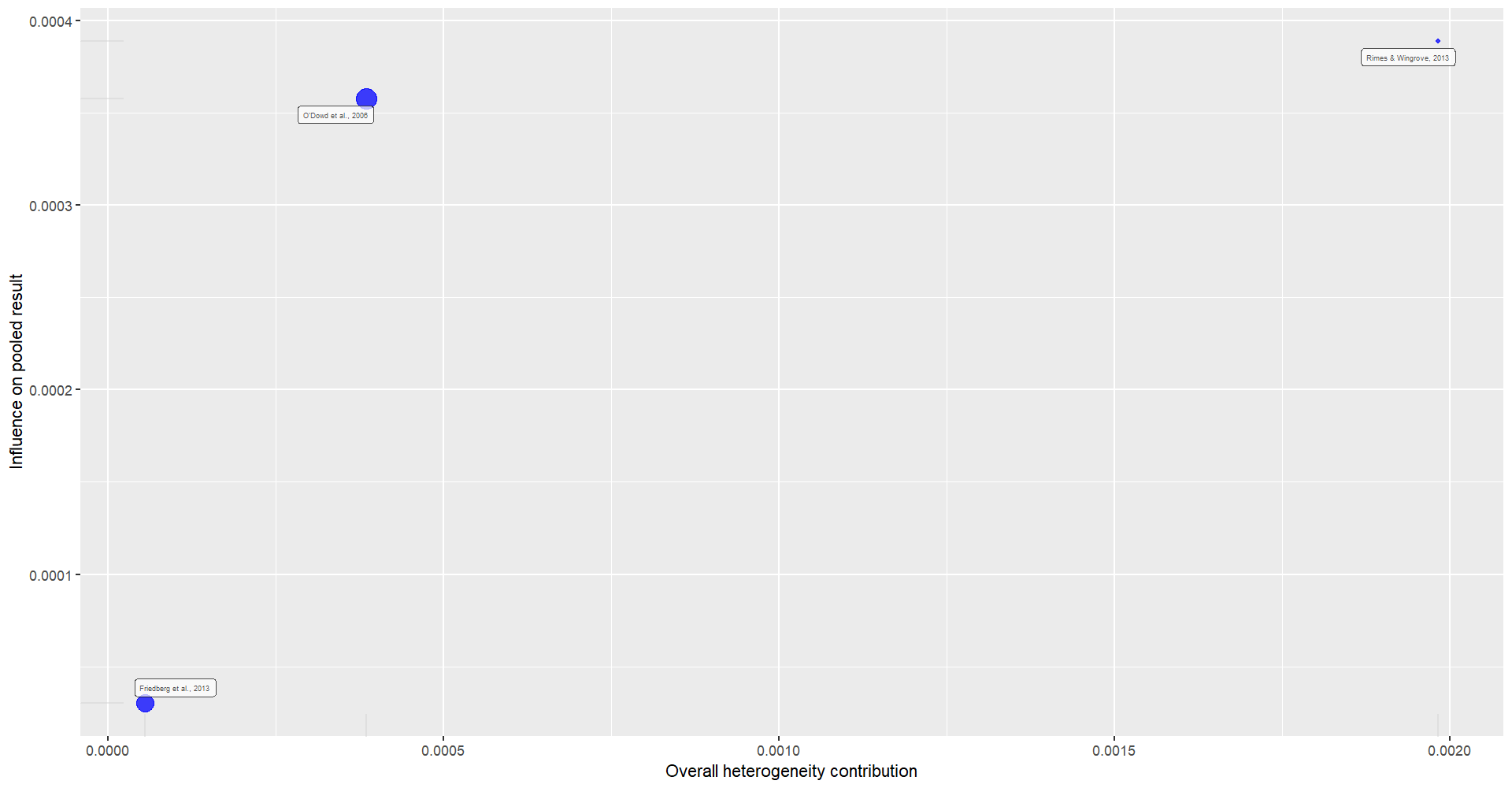


(A)


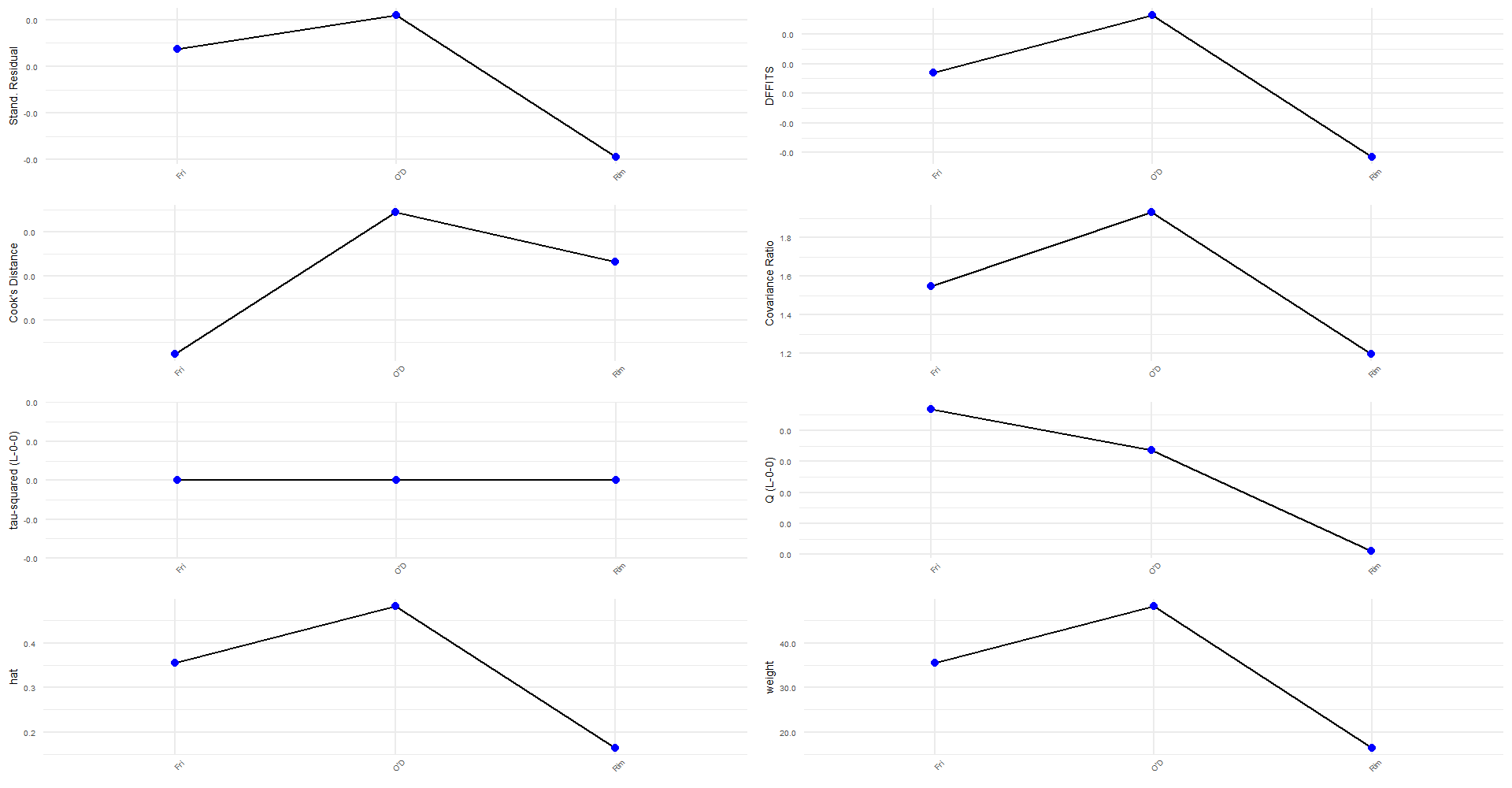


(B)


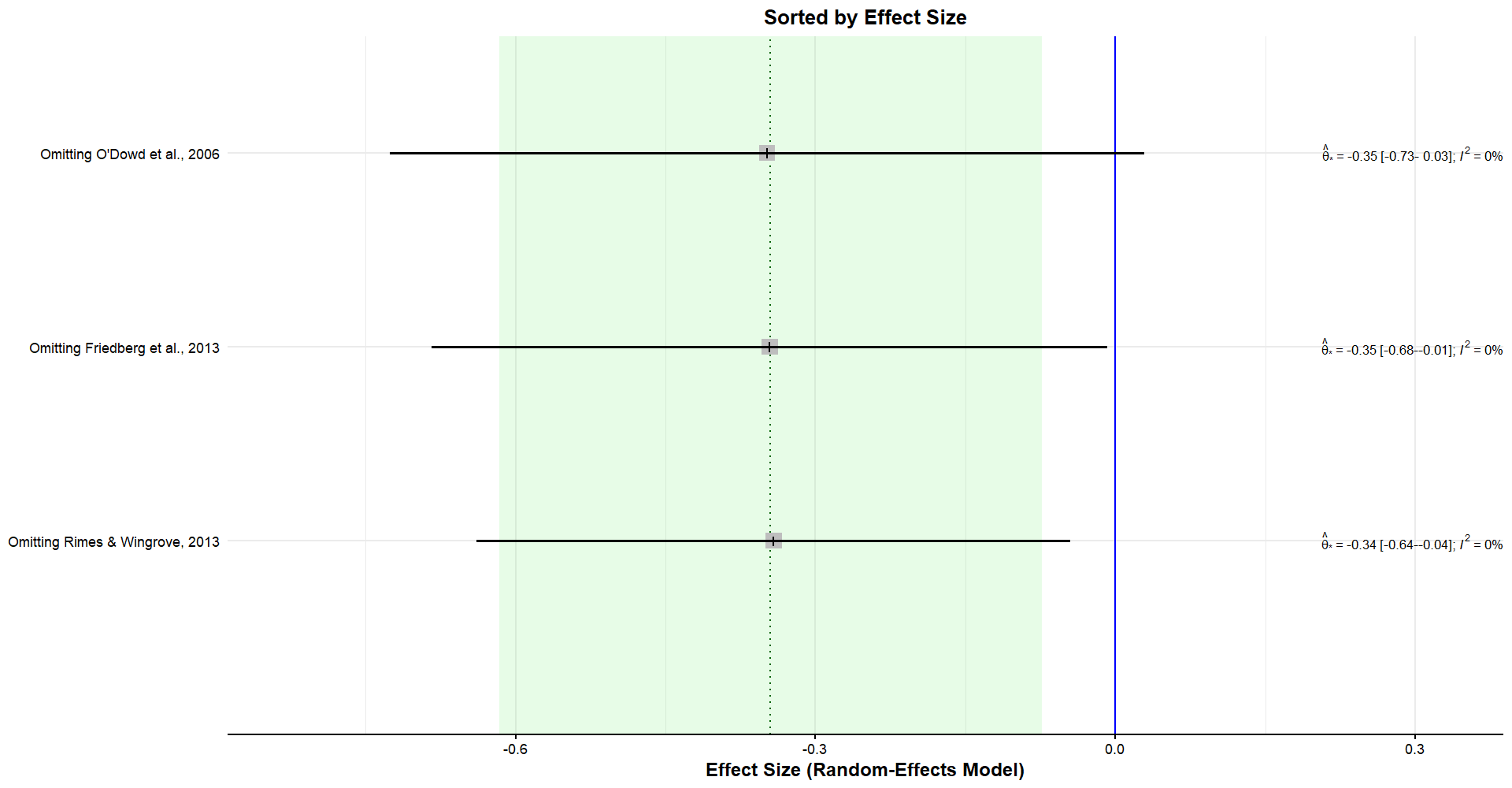


(C)


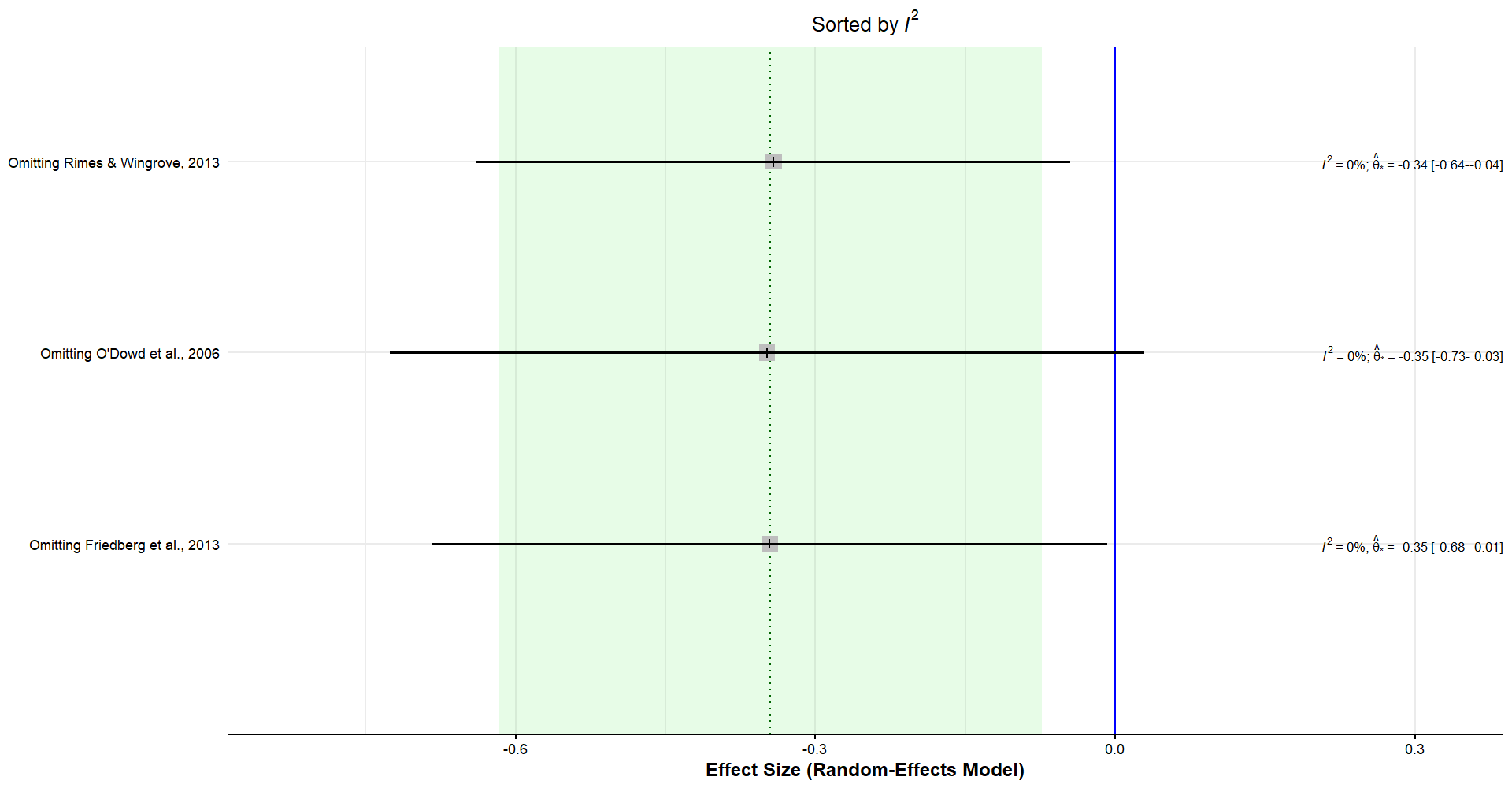


(D)


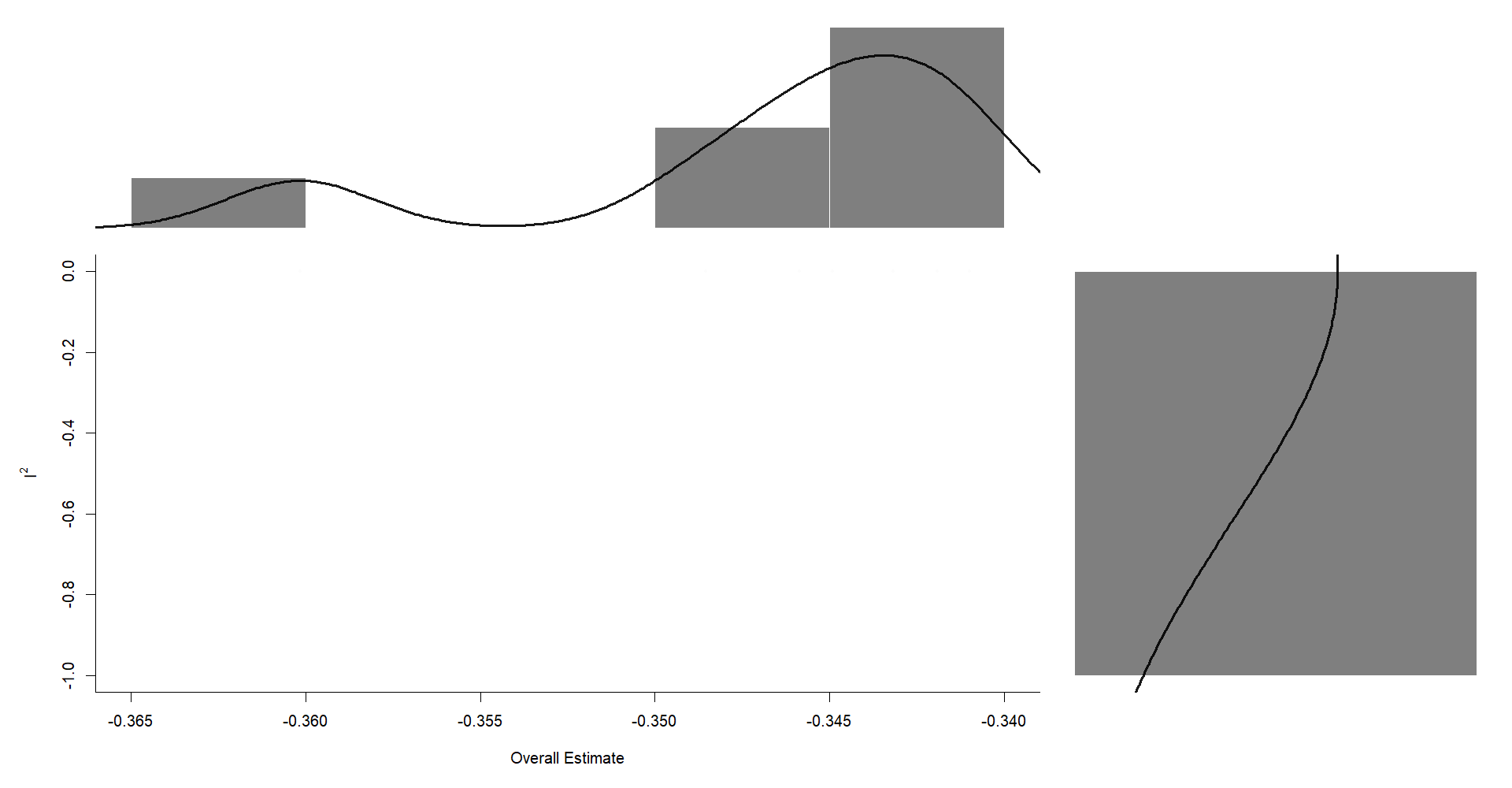


(E)

*Note.* A: Baujat plot; B: influence plot; C: leave one out method, influence on effect size; D: leave one out method, influence on heterogeneity; E: Graphic Display of Heterogeneity (GOSH) plot.

**Figure S18**

*Results for the influence analyses for non-completion*


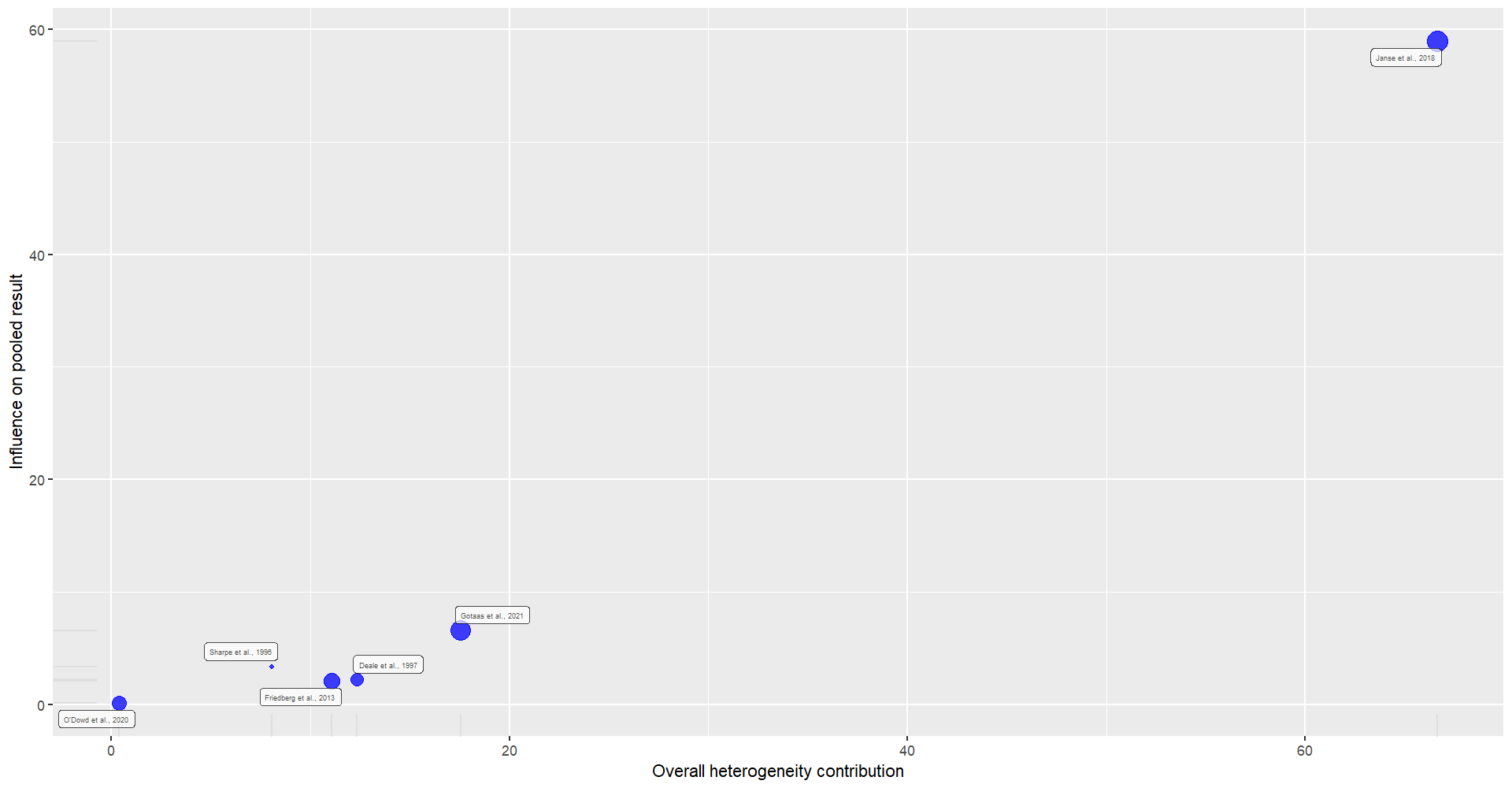


(A)


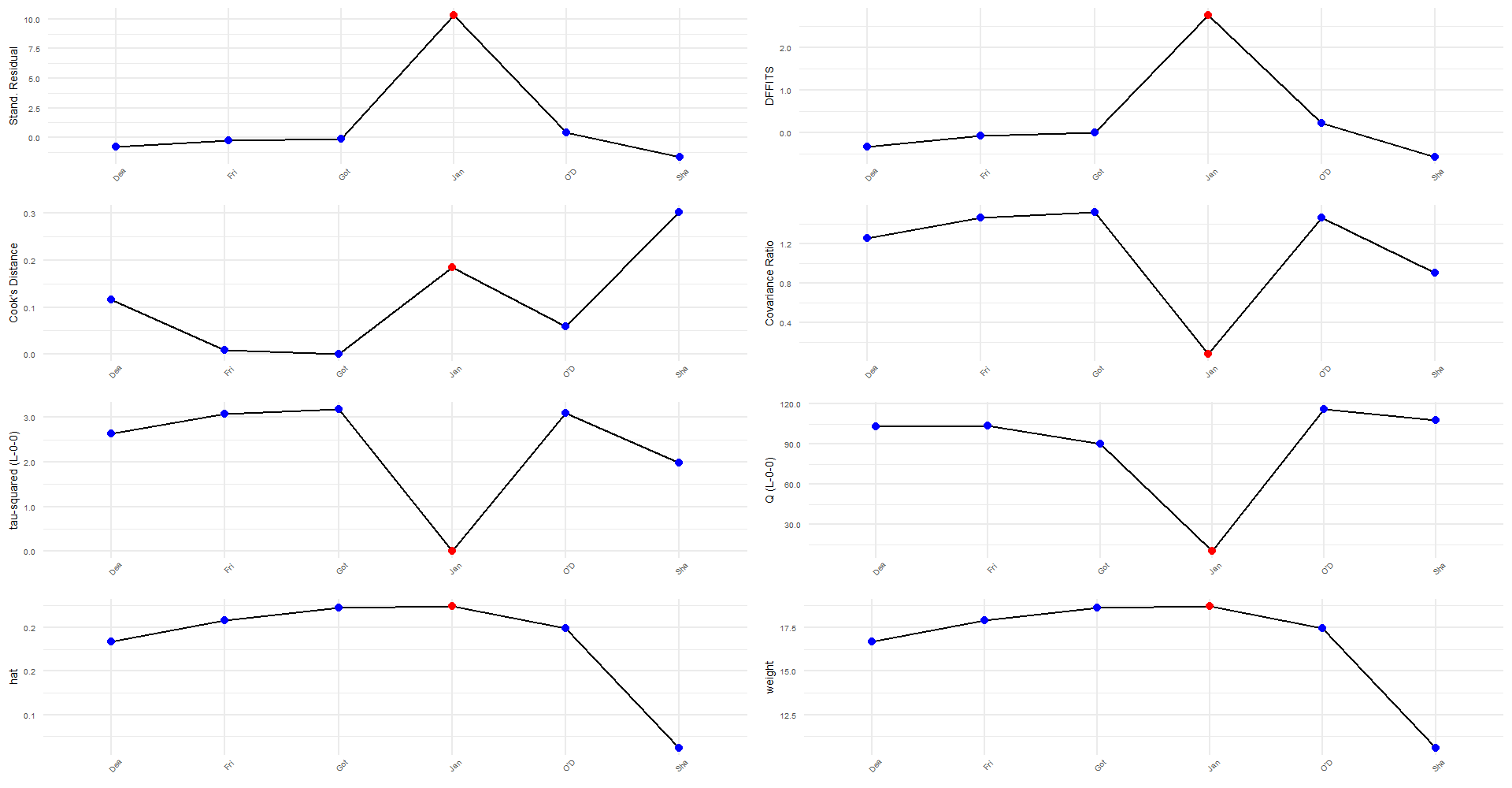


(B)


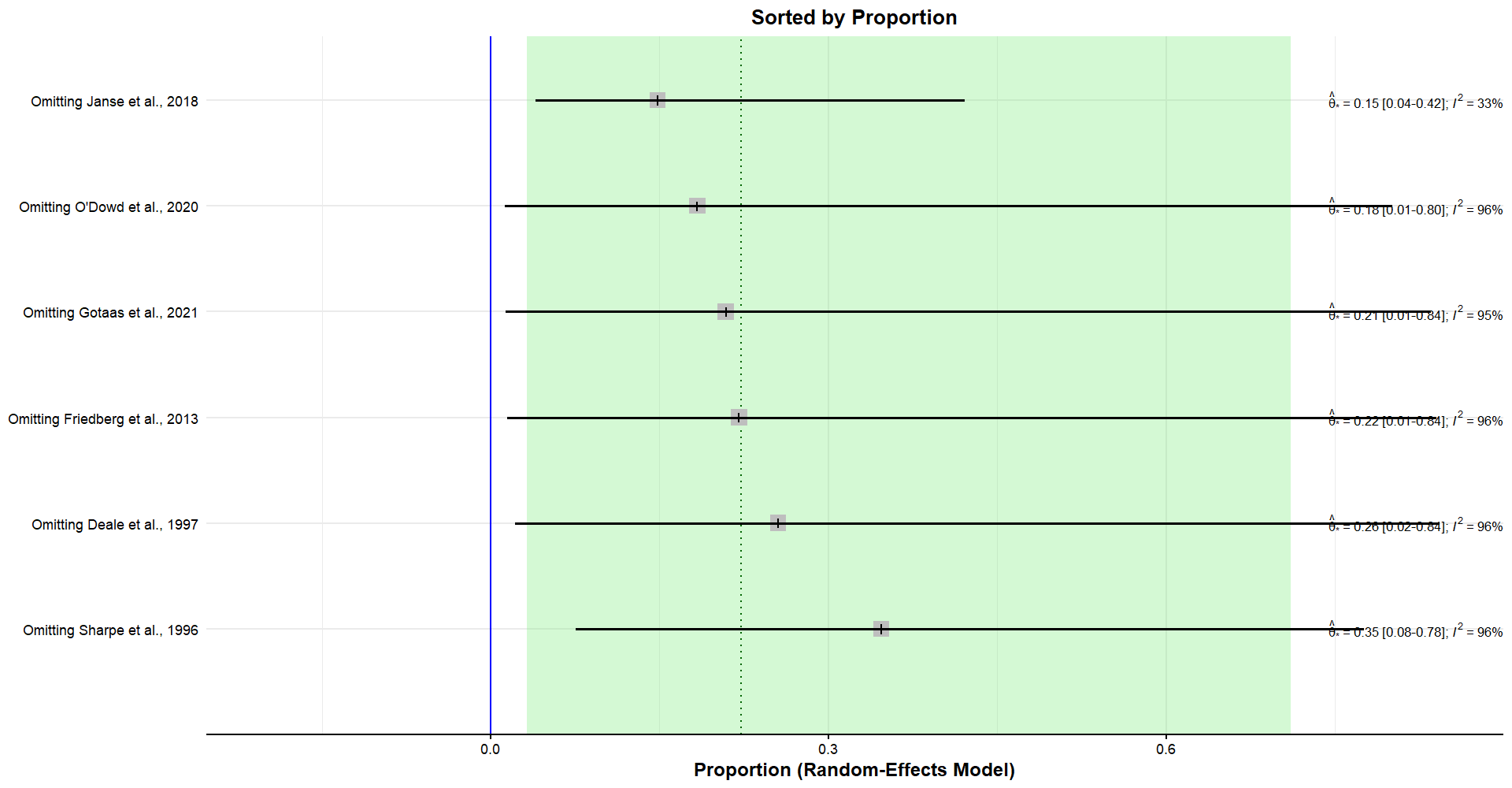


(C)


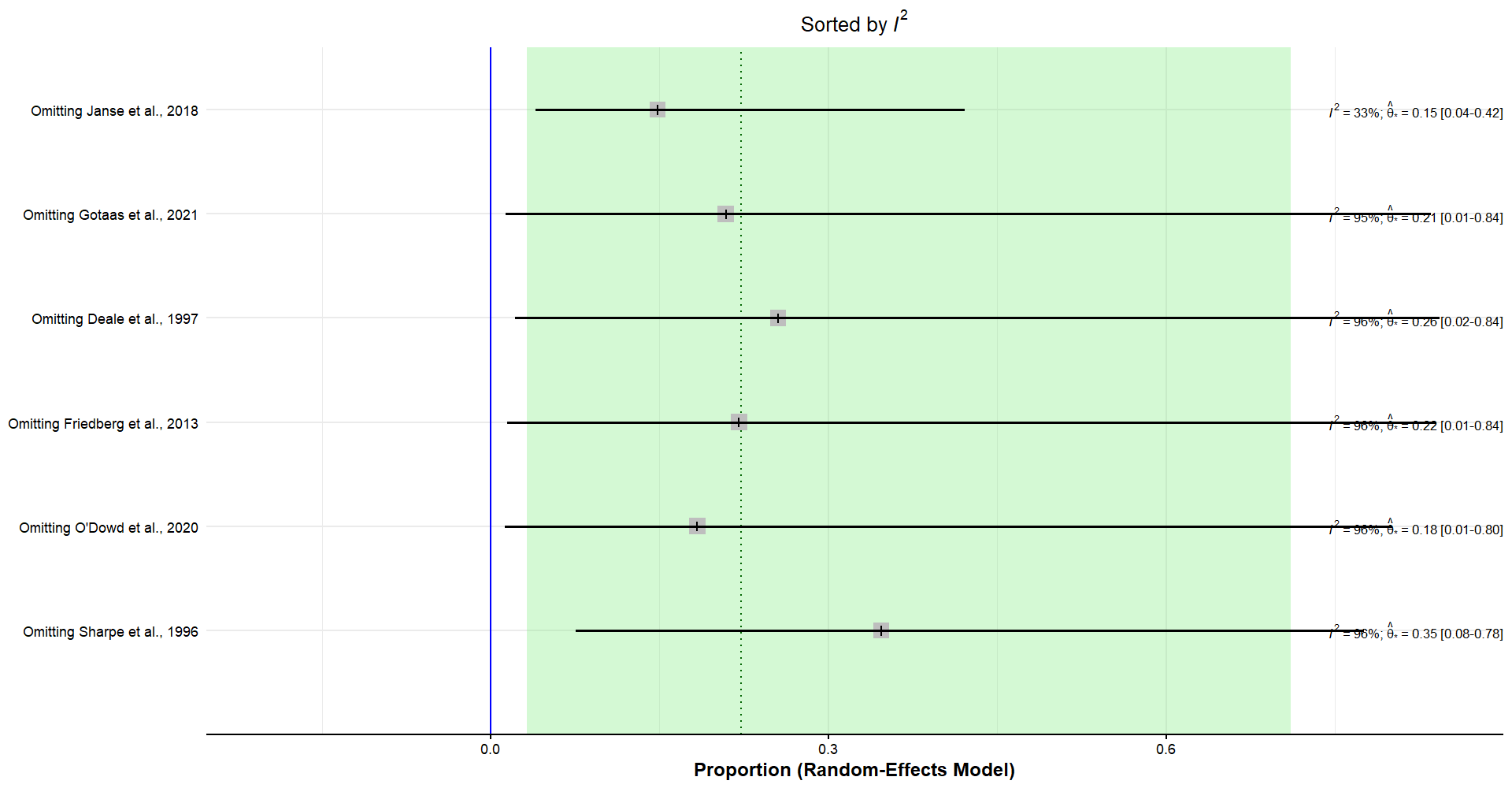


(D)

*Note.* A: Baujat plot; B: influence plot; C: leave one out method, influence on effect size; D: leave one out method, influence on heterogeneity.

**Figure S19**

*Results for the influence analyses for drop-out*


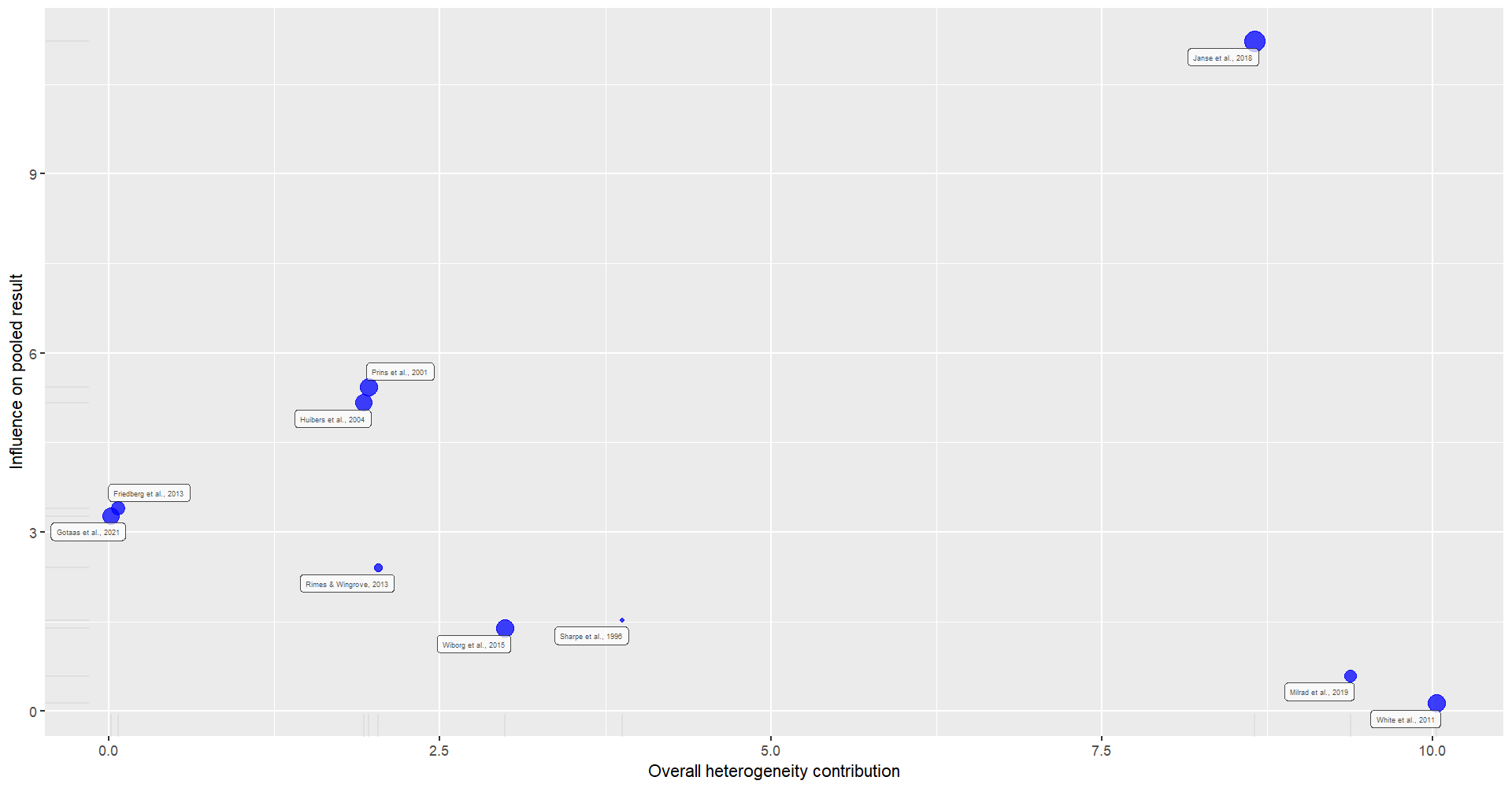


(A)


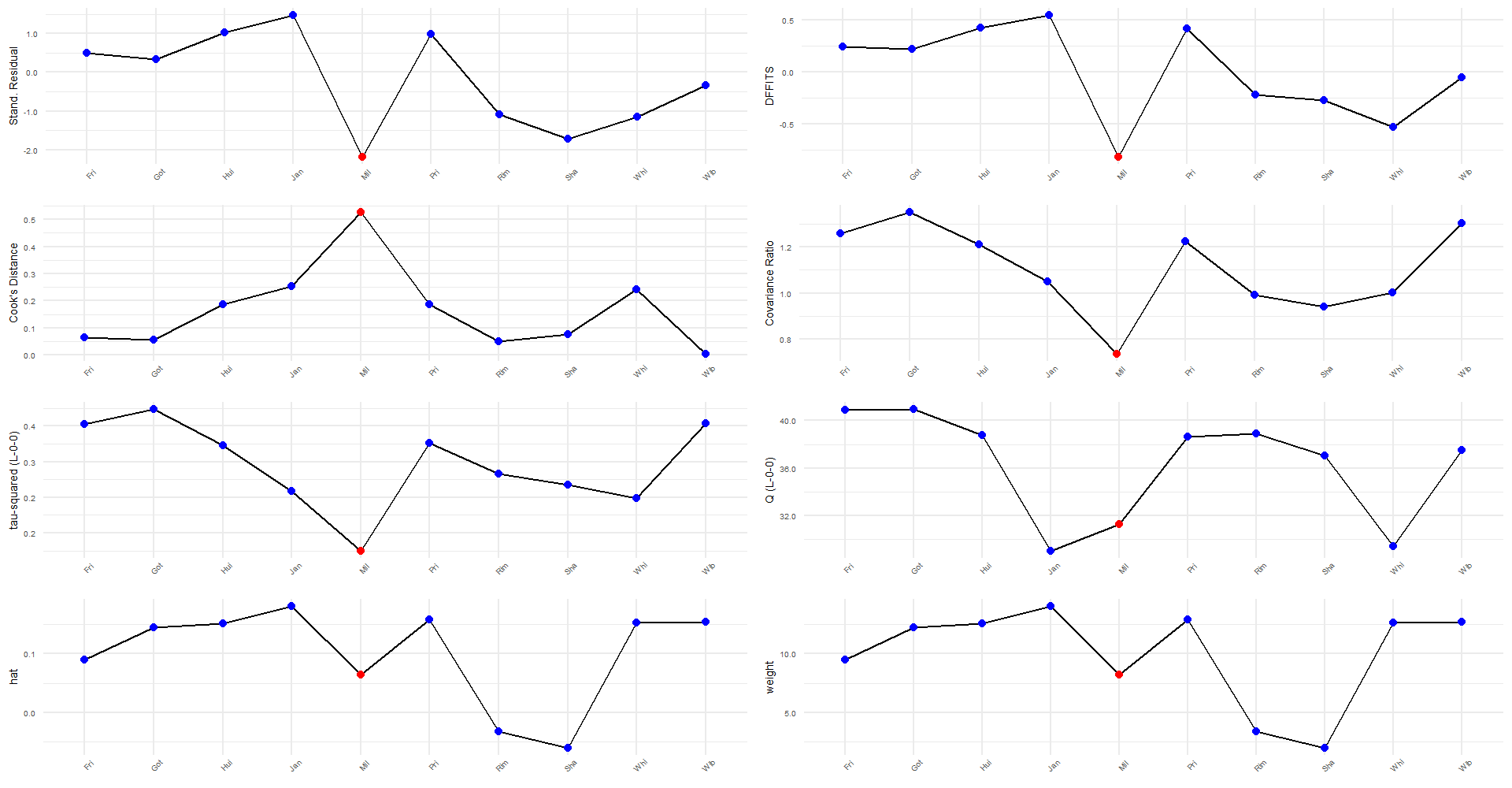


(B)


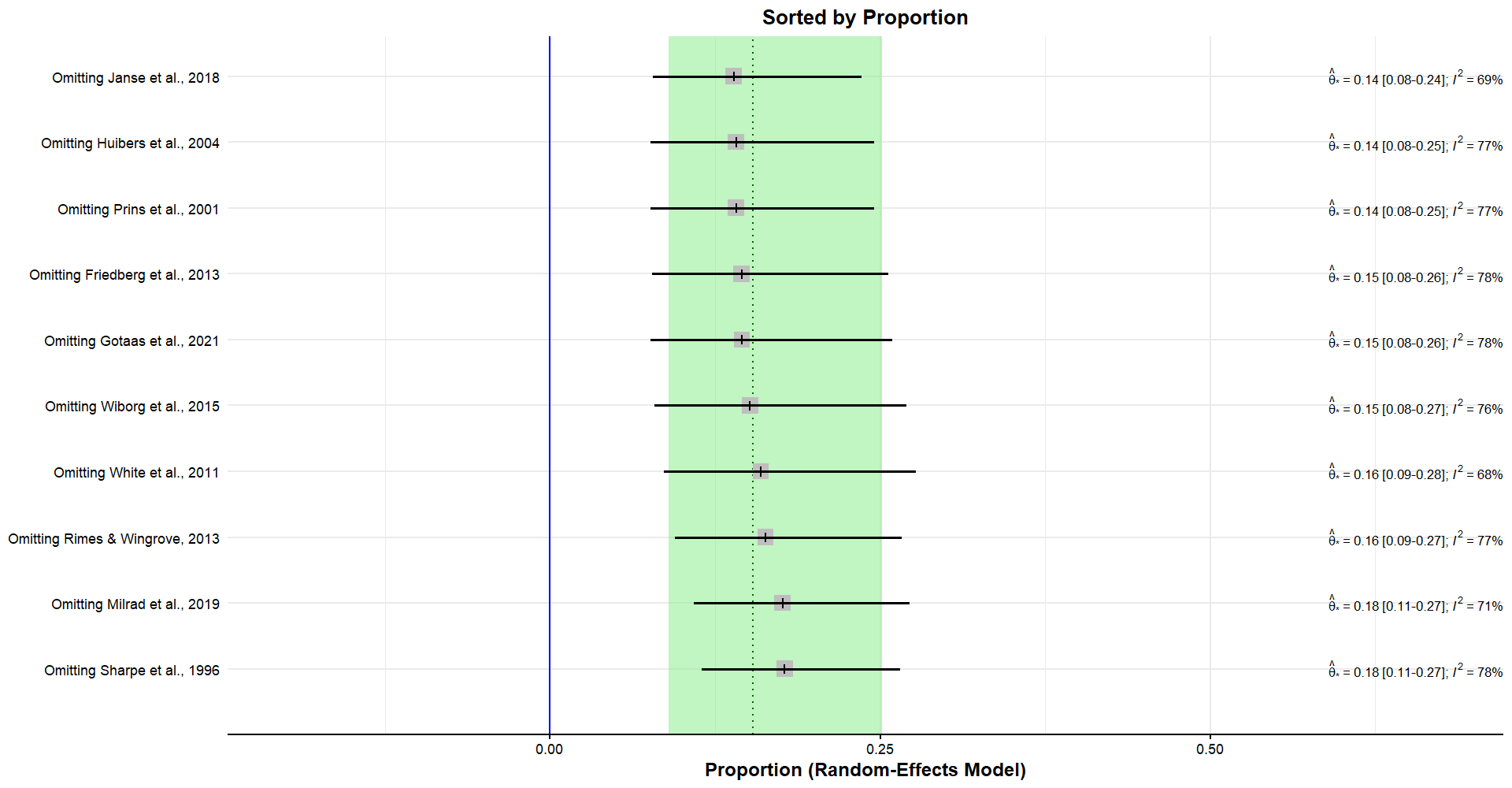


(C)


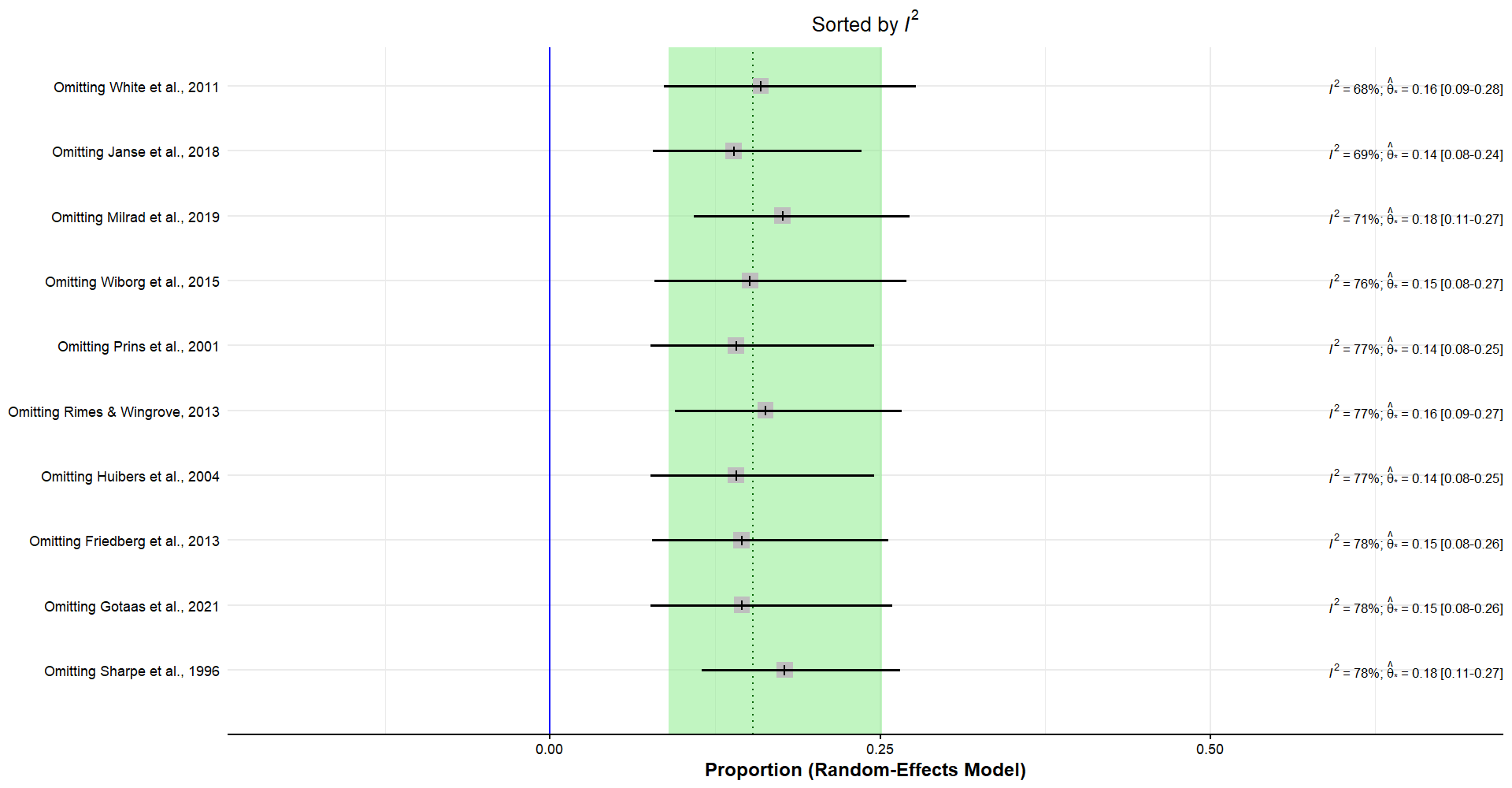


(D)

*Note.* A: Baujat plot; B: influence plot; C: leave one out method, influence on effect size; D: leave one out method, influence on heterogeneity.

**Figure S20**

*Results for the influence analyses for treatment refusal*


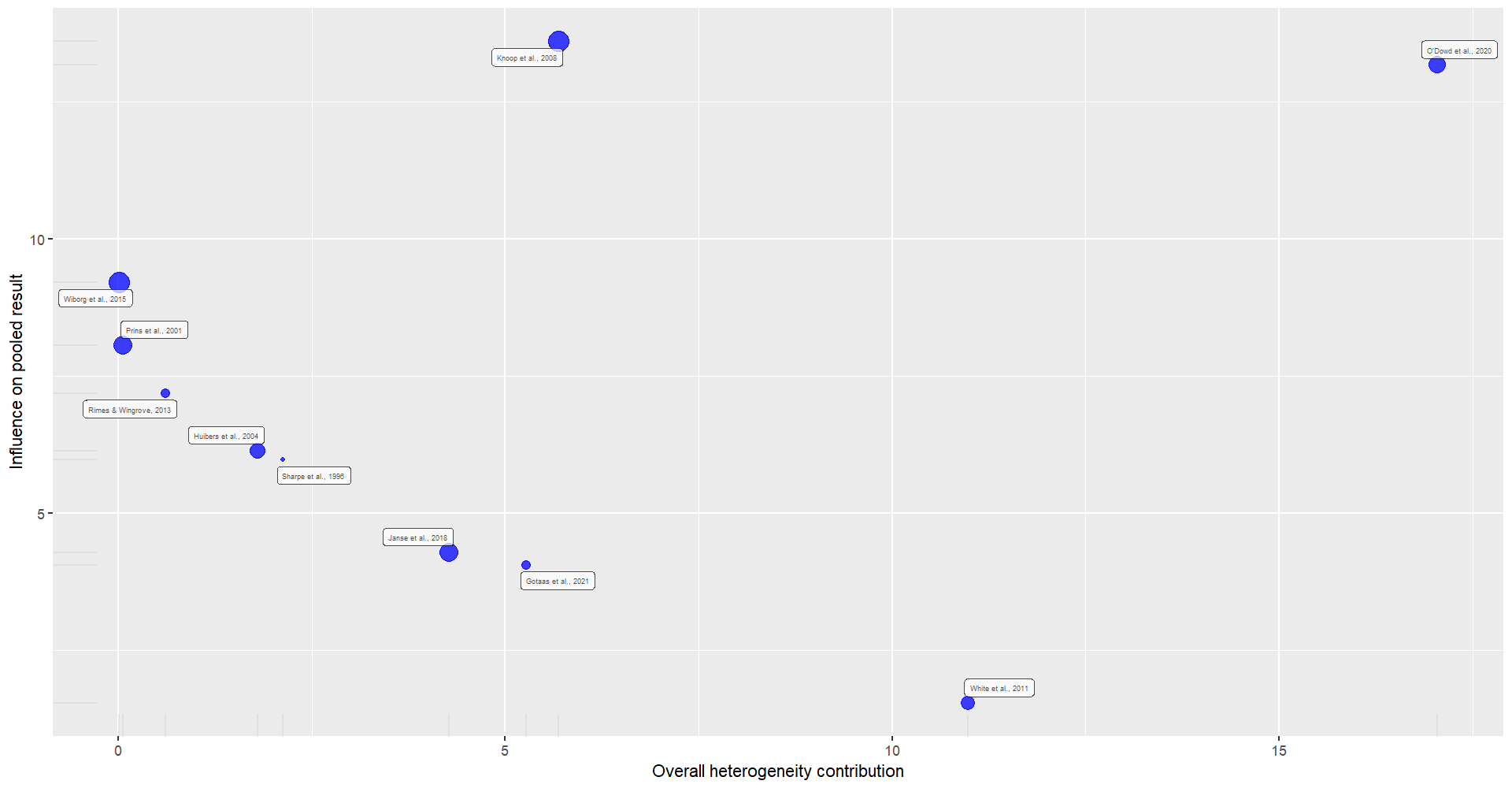


(A)


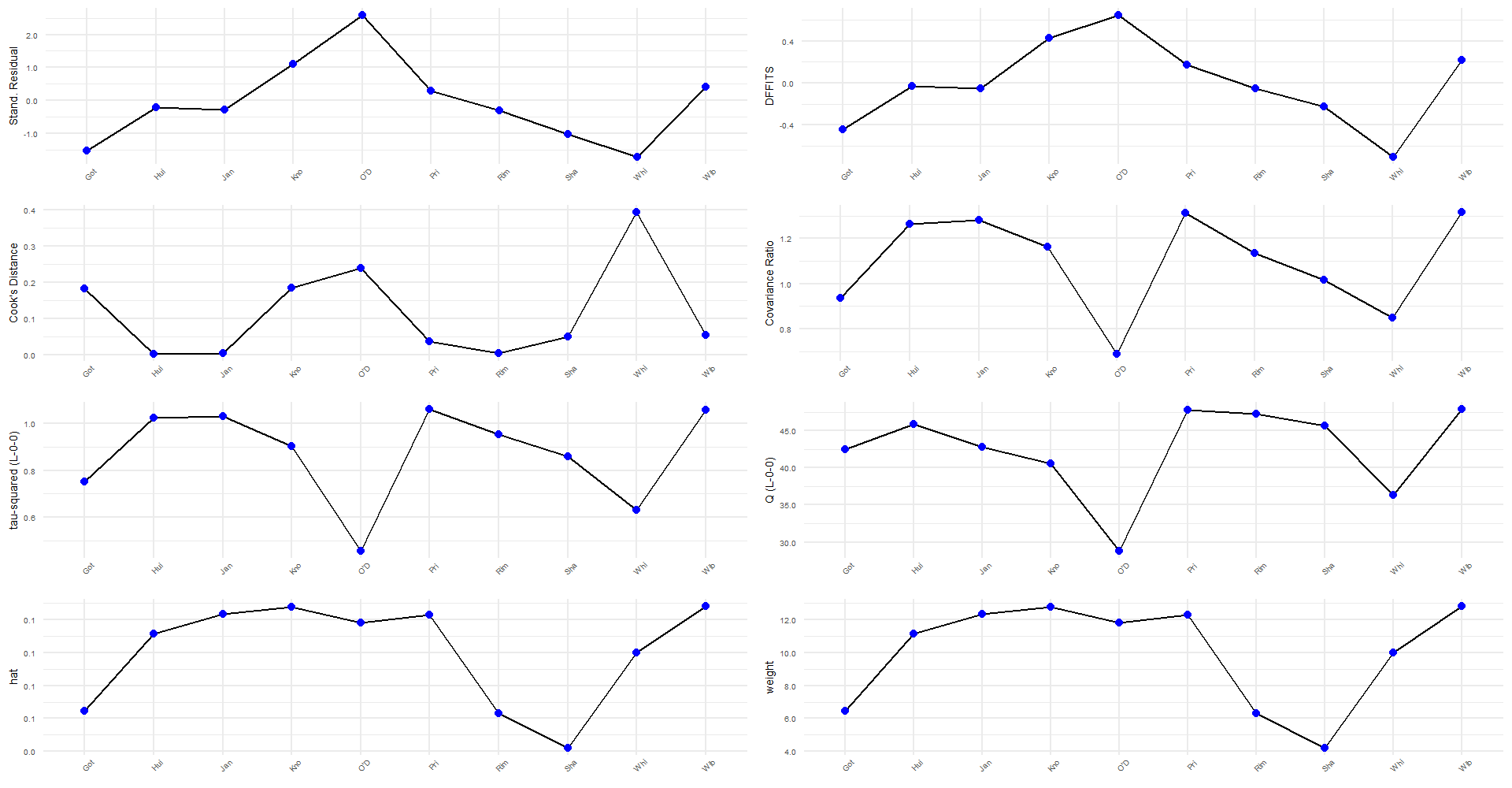


(B)


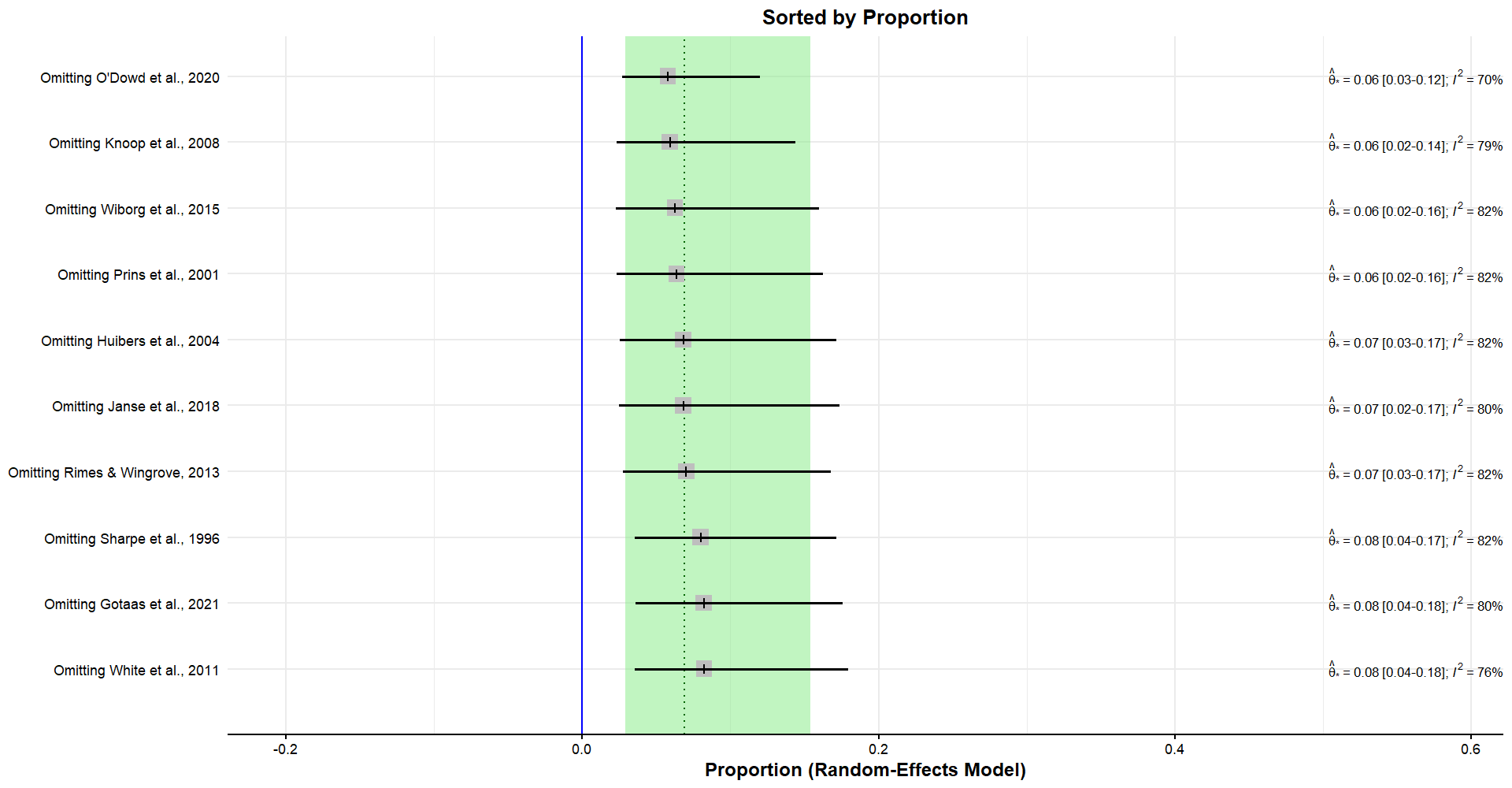


(C)


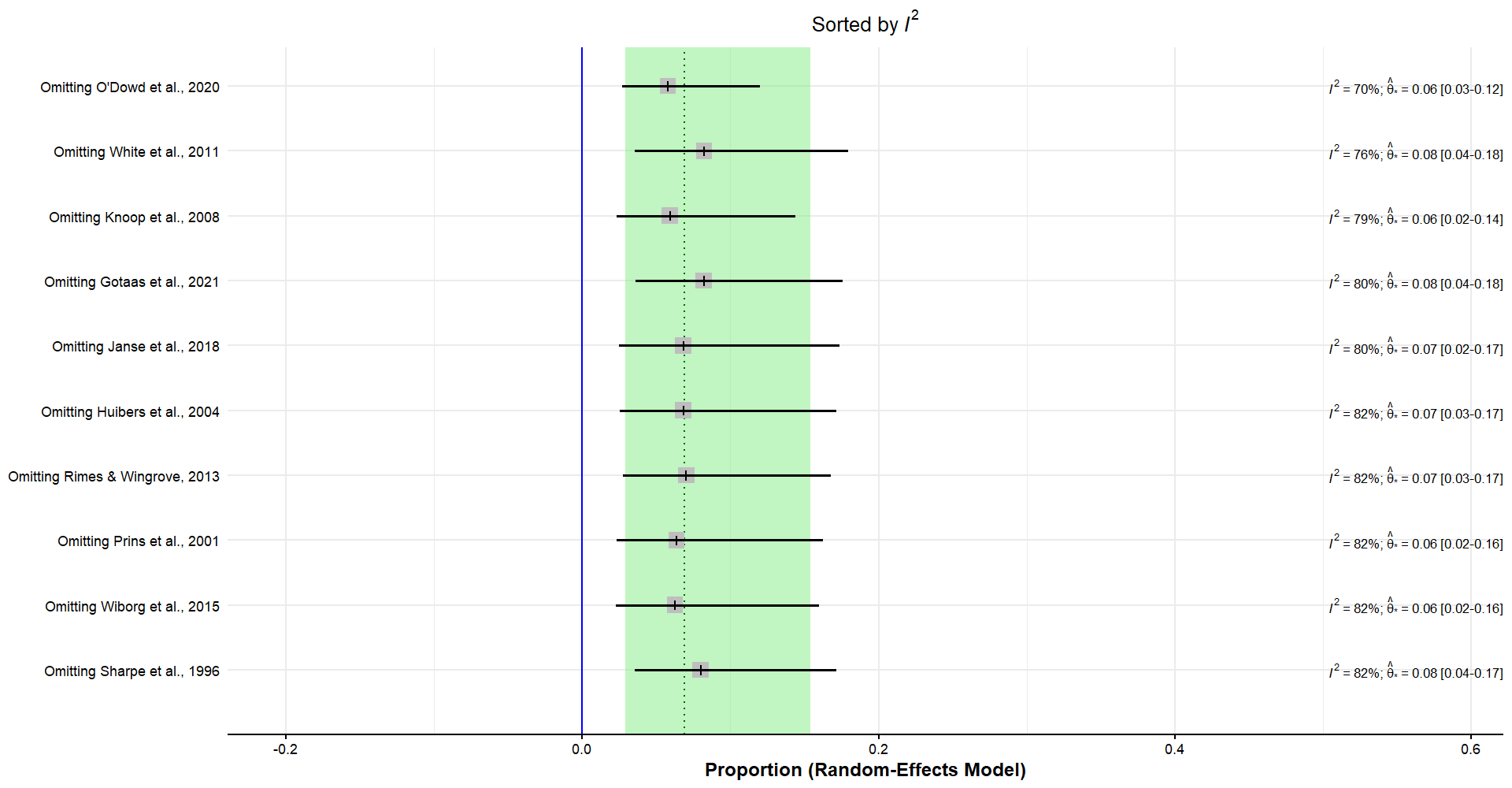


(D)

*Note.* A: Baujat plot; B: influence plot; C: leave one out method, influence on effect size; D: leave one out method, influence on heterogeneity.

**Figure S21**

*Results for the influence analyses for average proportion of session completed*


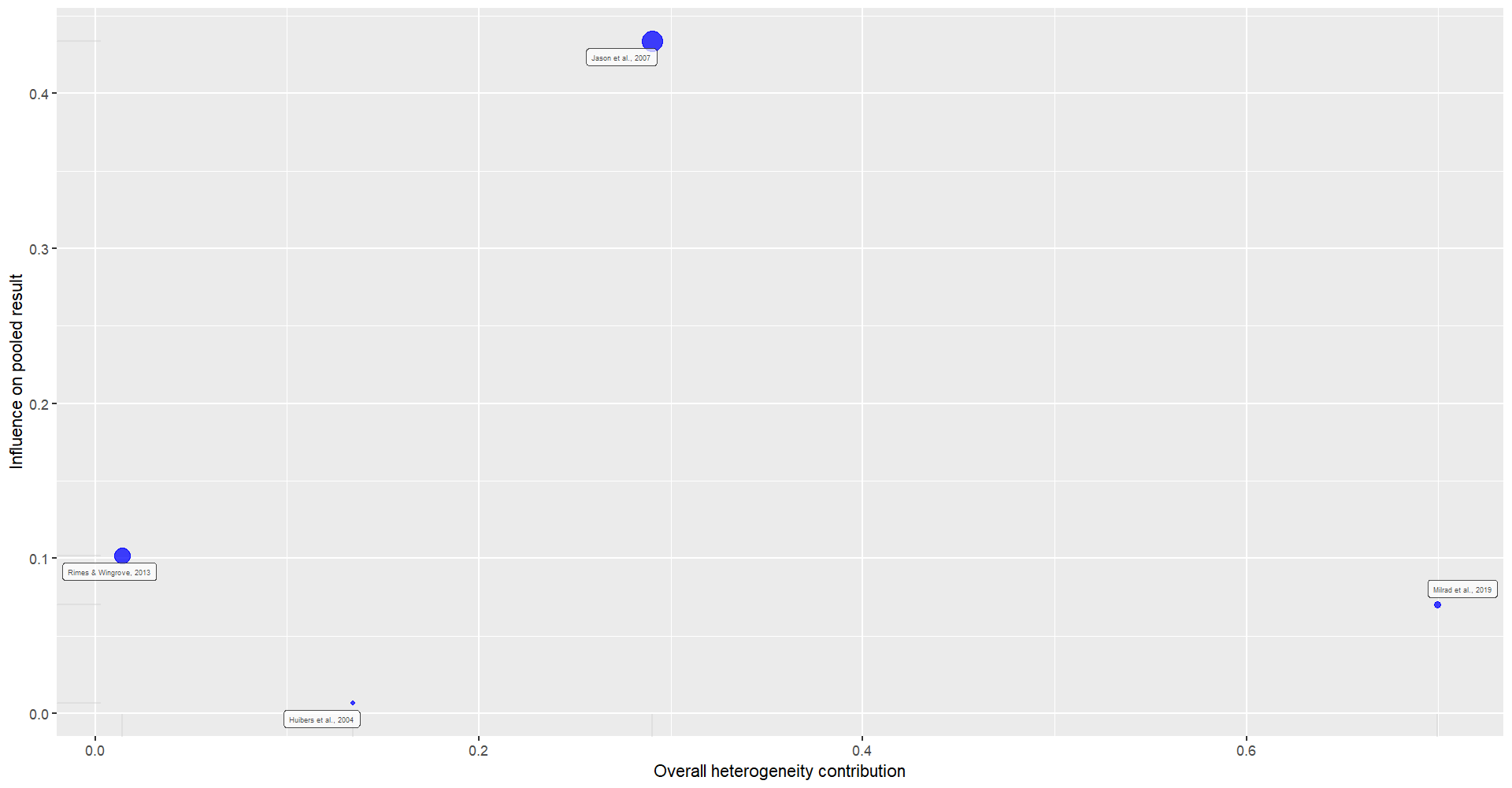


(A)


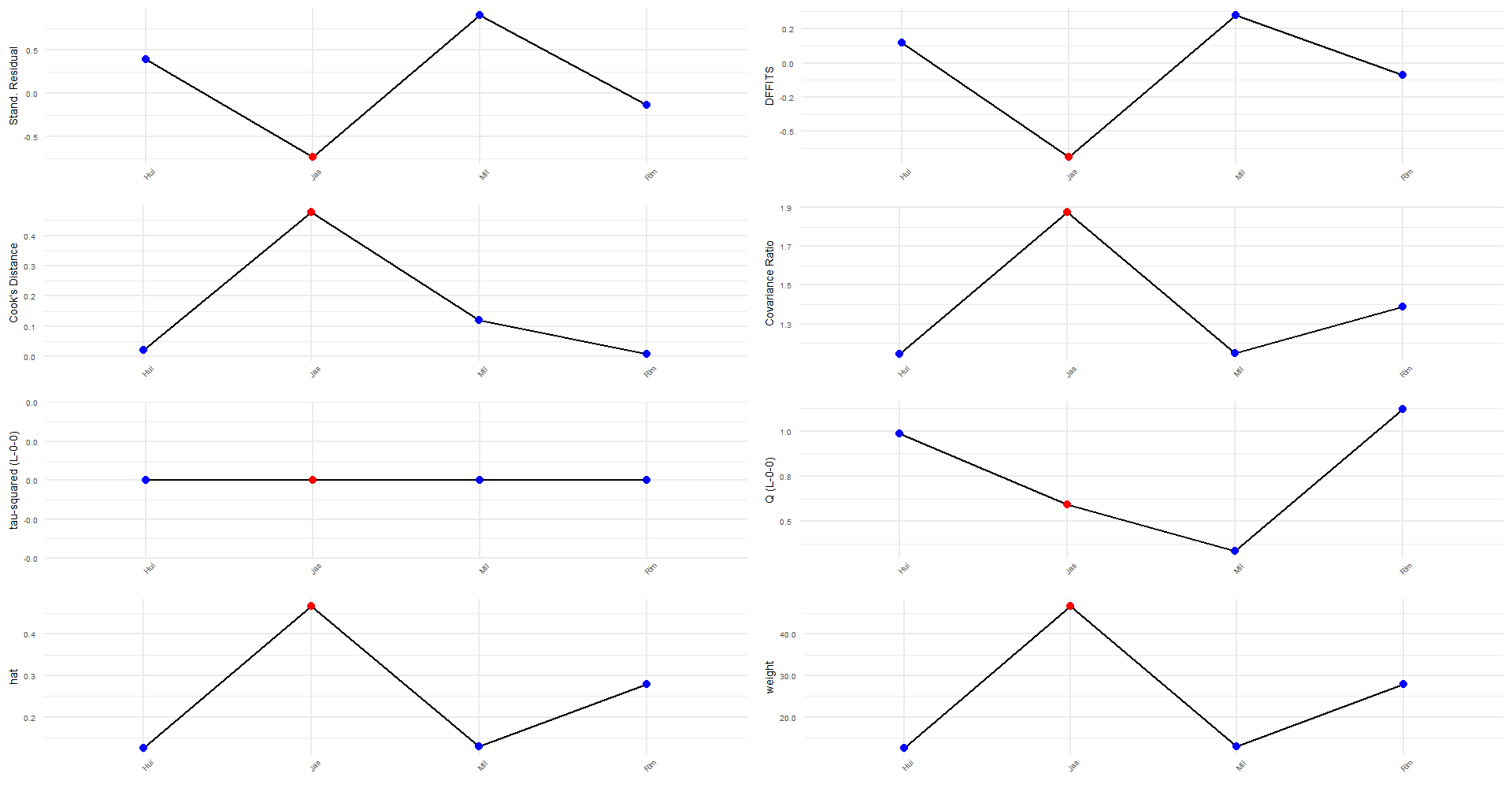


(B)


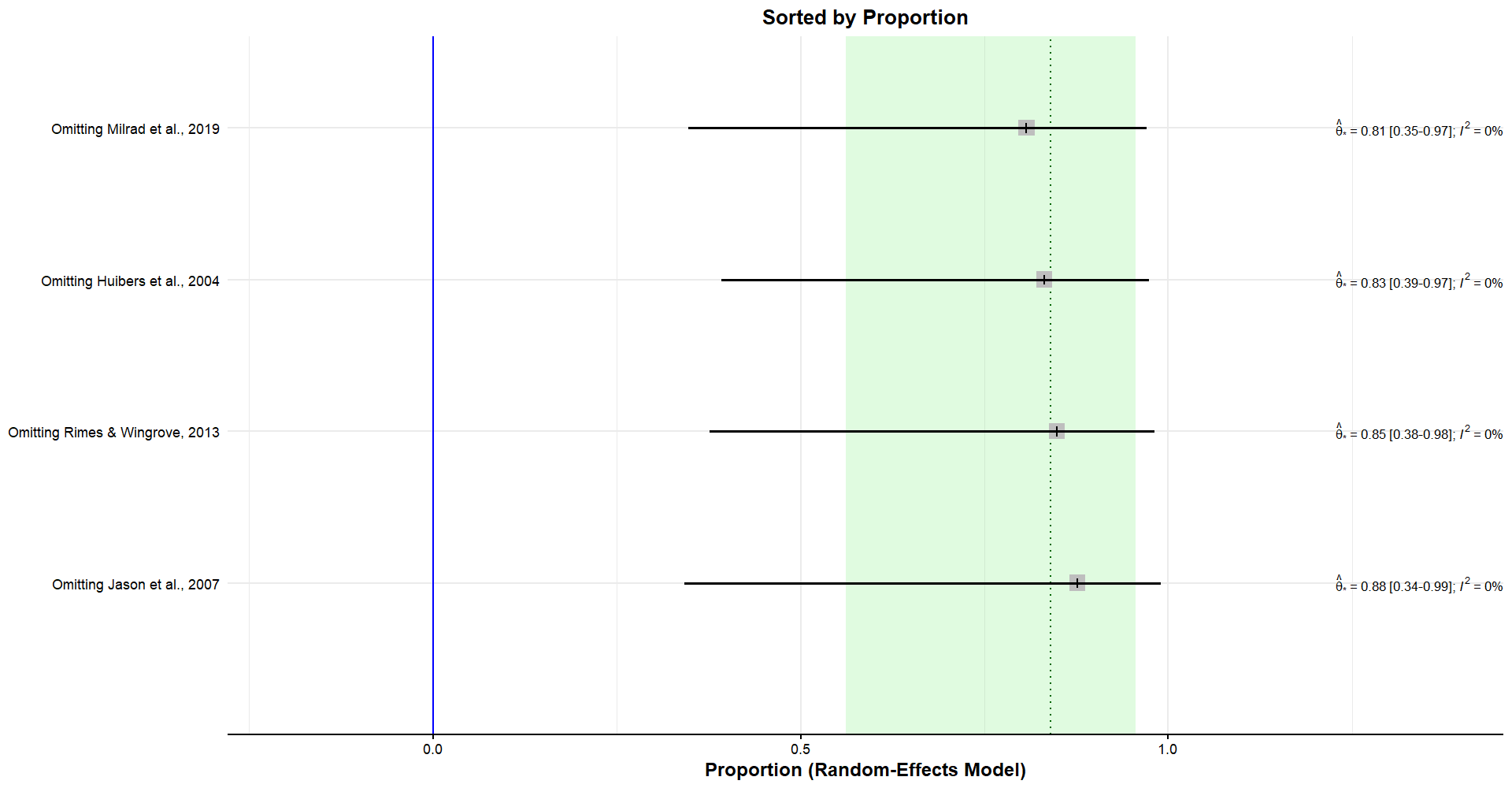


(C)


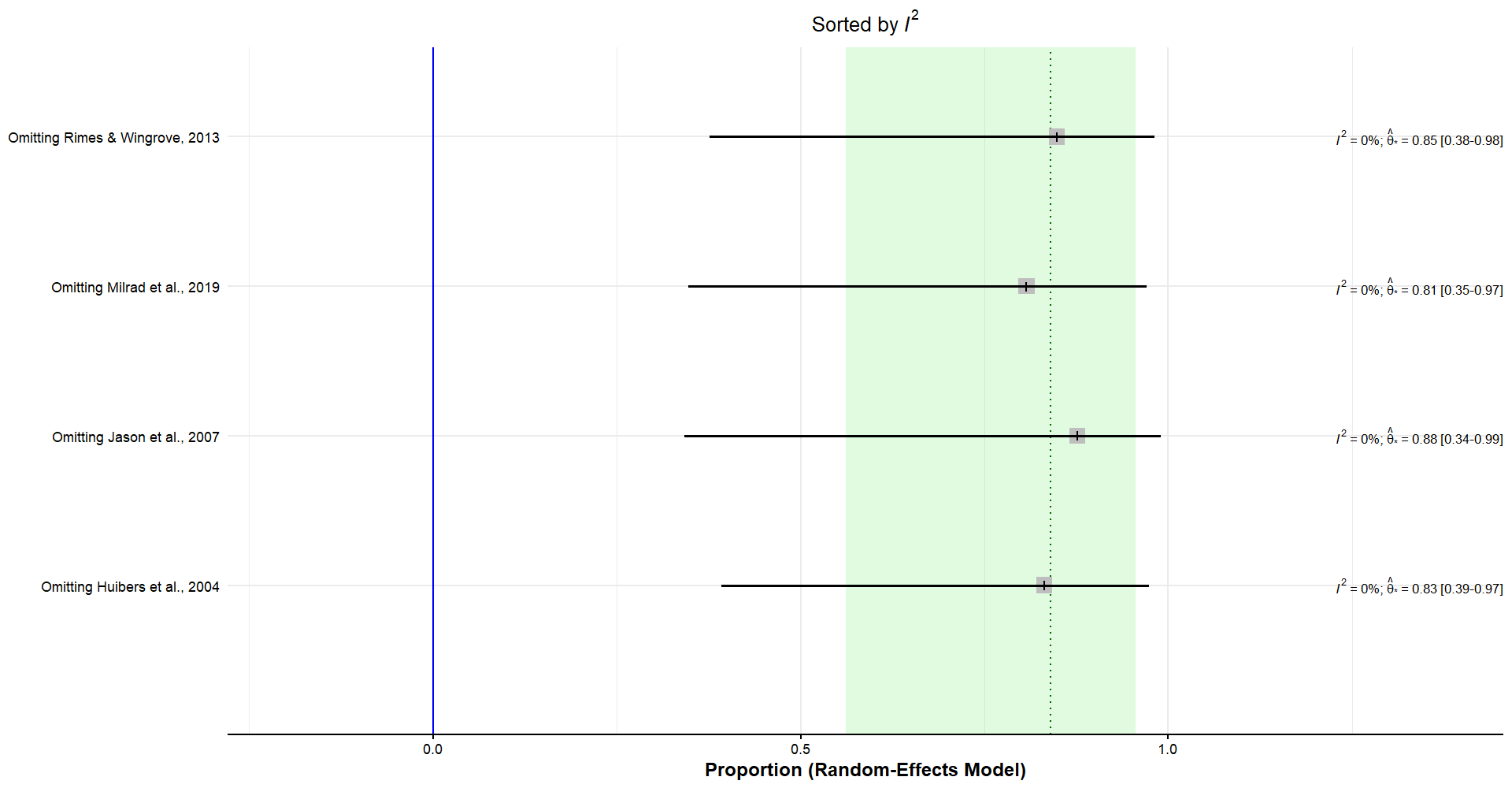


(D)

*Note.* A: Baujat plot; B: influence plot; C: leave one out method, influence on effect size; D: leave one out method, influence on heterogeneity.

# **Publication Bias**

**Figure S22**

*Publication bias*


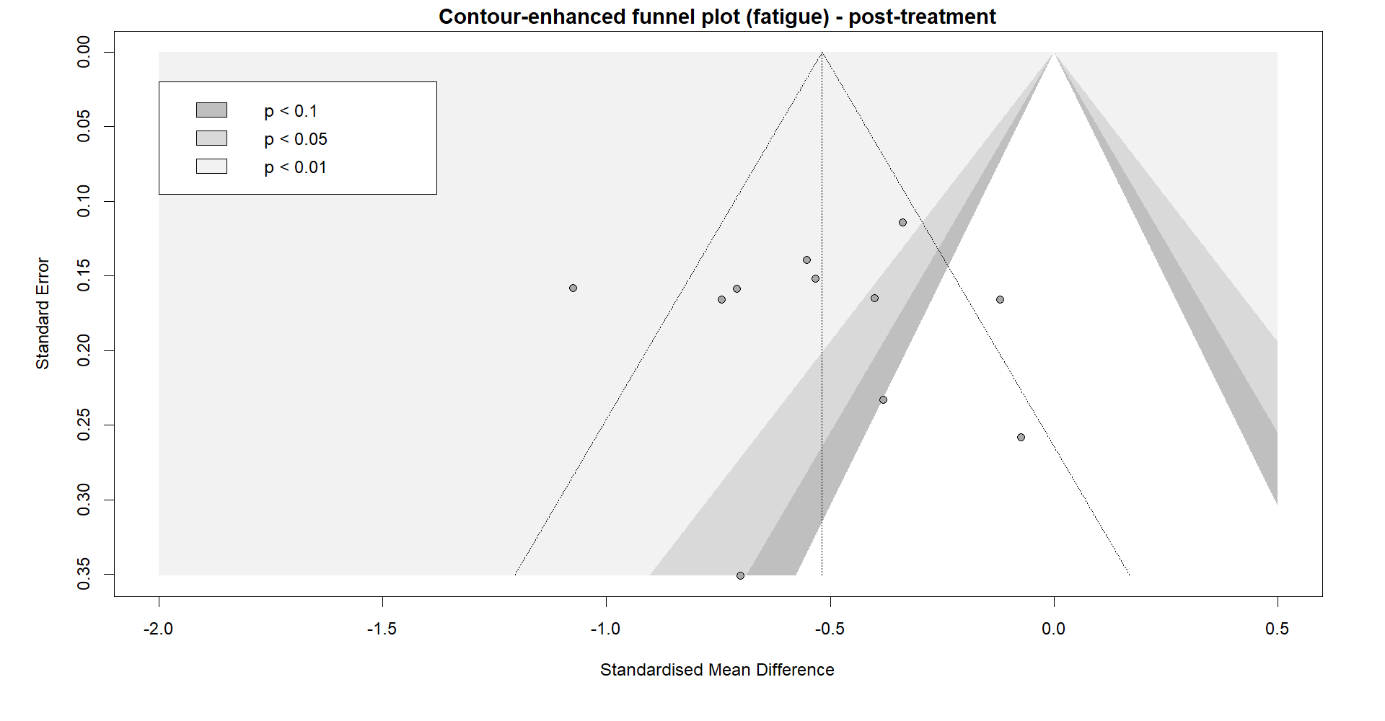


(A)


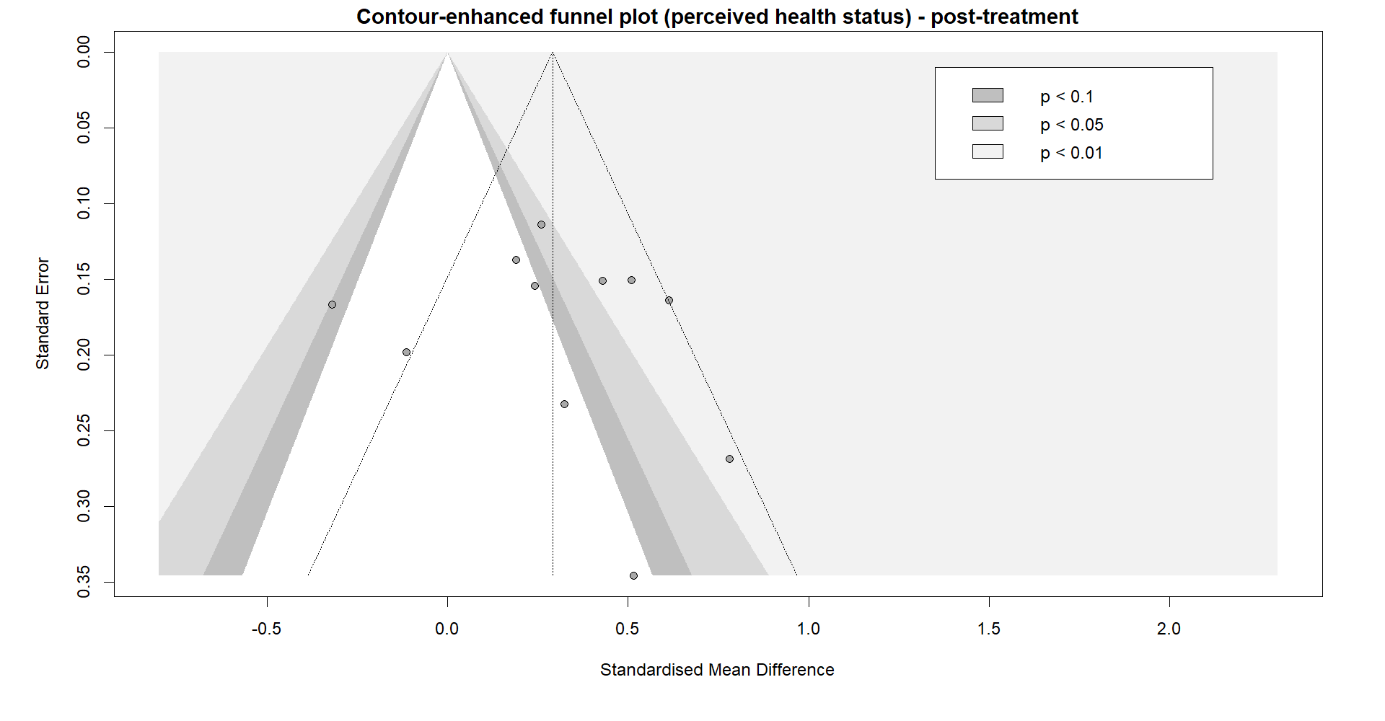


(B)


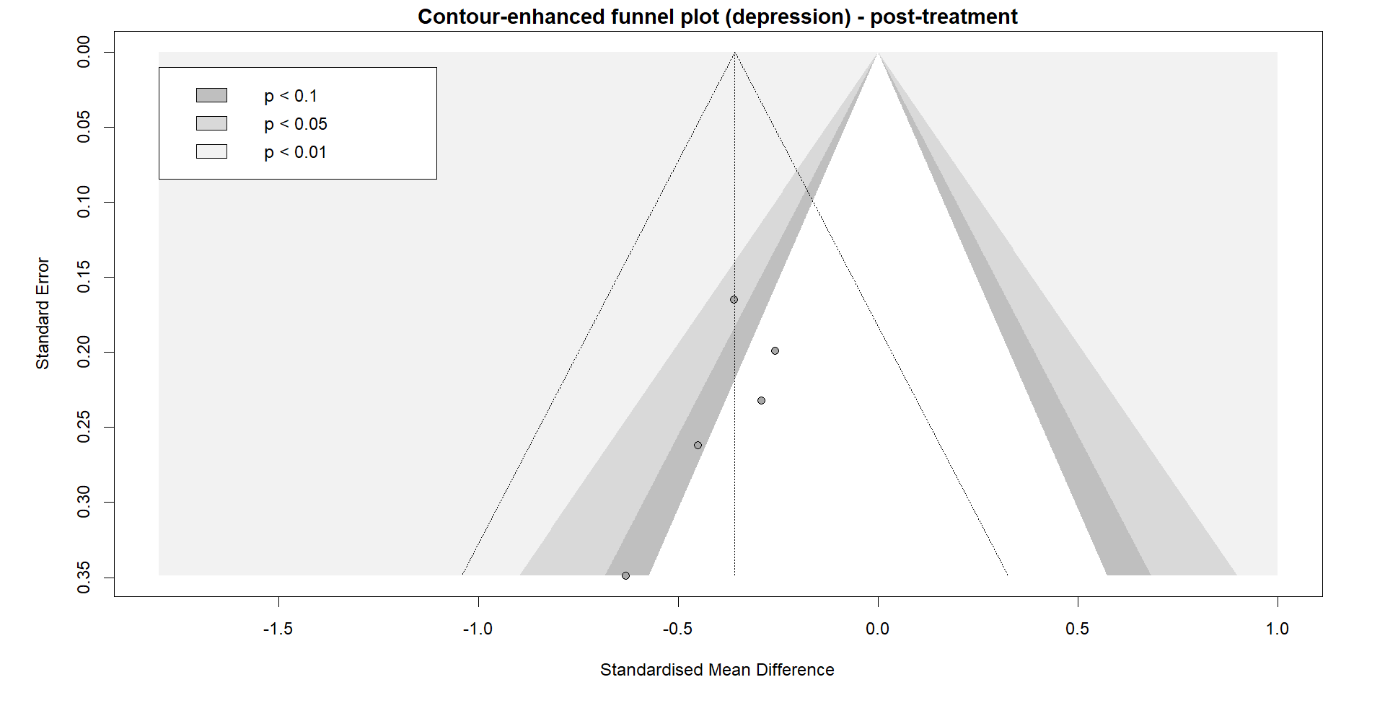


(C)


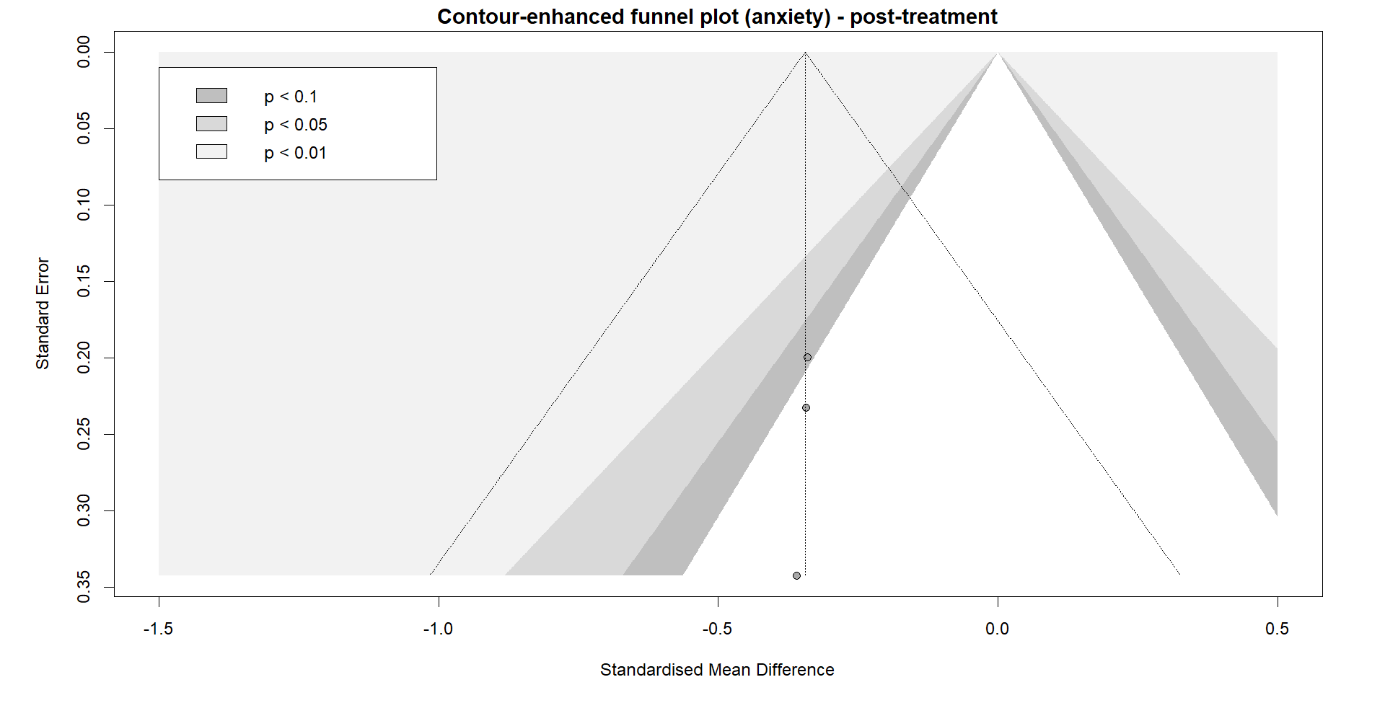


(D)


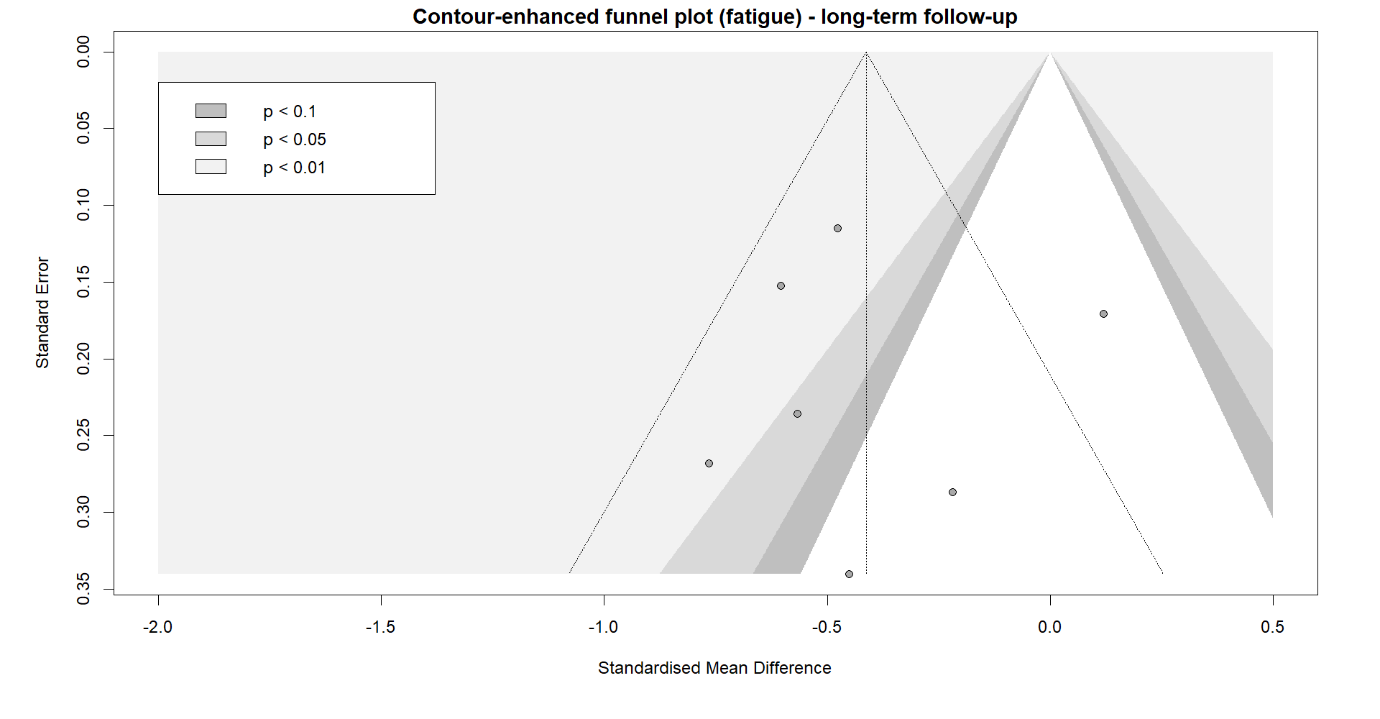


(E)


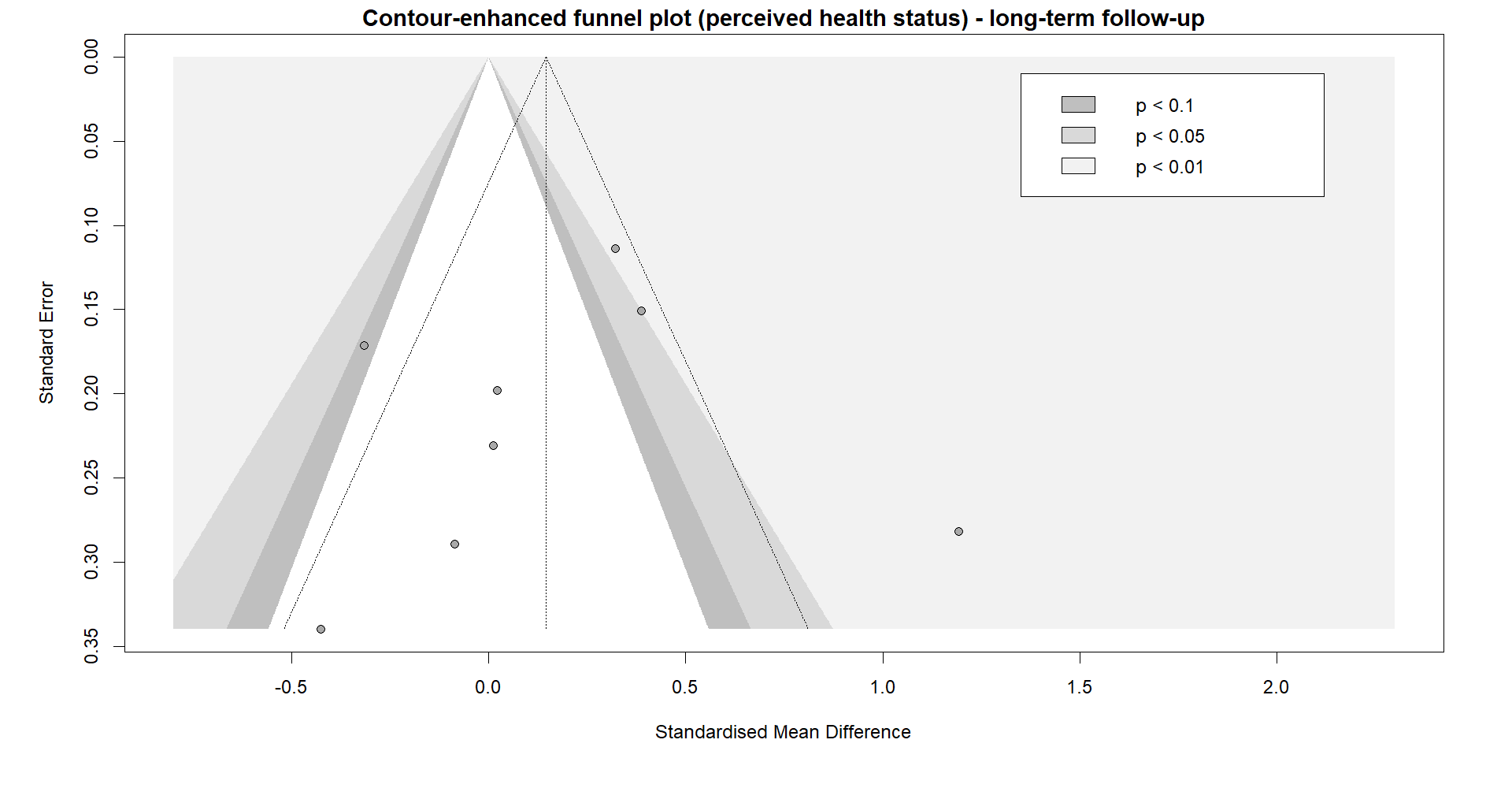


(F)


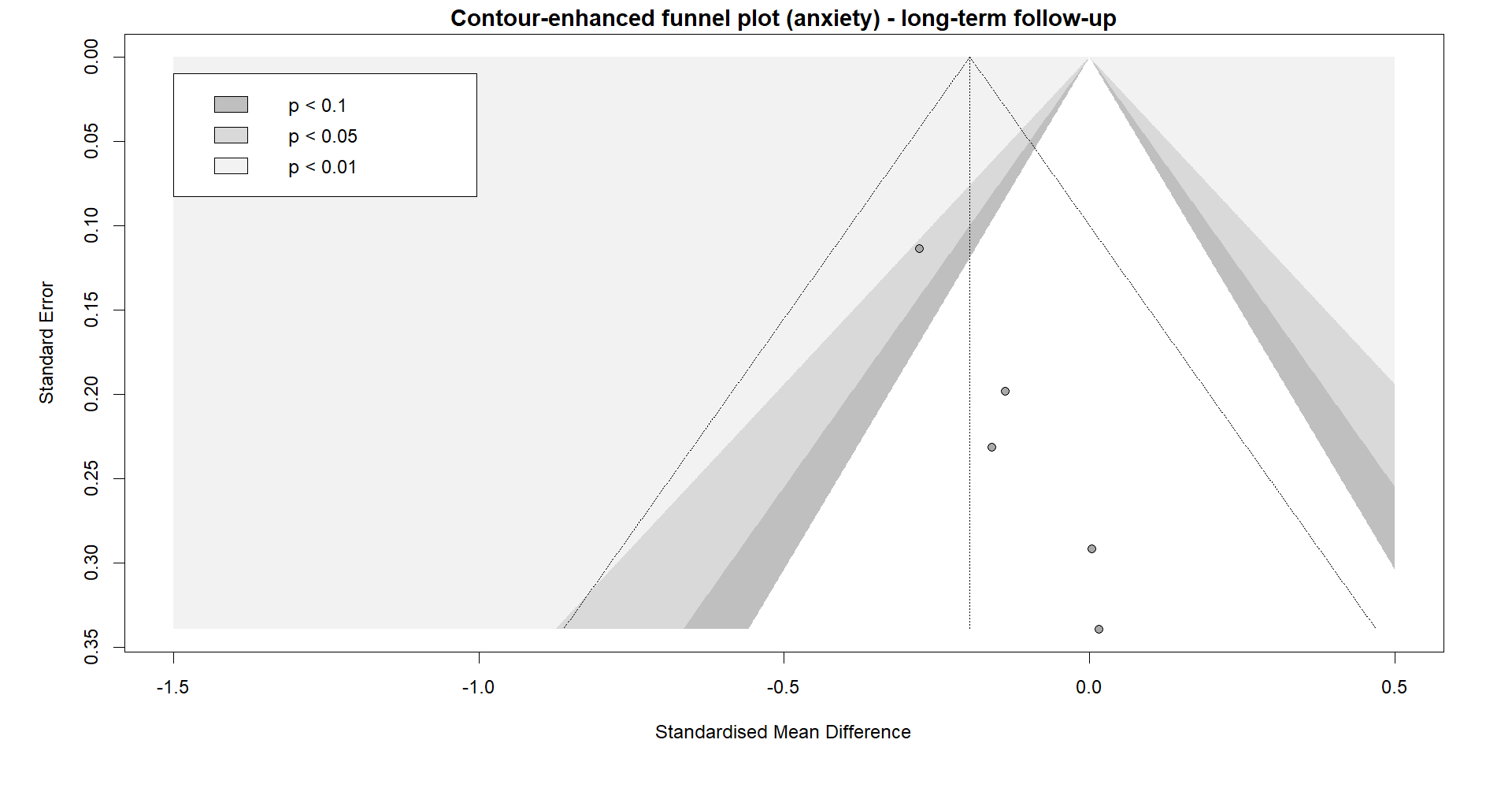


(G)

*Note.* A: fatigue at post-treatment; B: perceived health status at post-treatment; C: depression at post-treatment; D: anxiety at post-treatment; E: fatigue at long-term follow-up; F: perceived health status at long-term follow-up; G: anxiety at long-term follow-up.

**Table S11**

*Results of Egger’s regression tests*

| Outcome | Intercept | *t* | *p* |
| --- | --- | --- | --- |
| Post-treatment |  |  |  |
| Fatigue | 0.10 | 0.05 | 0.96 |
| Perceived Health Status | 0.94 | 0.47 | 0.65 |
| Depression^a^ | -1.33 | -1.62 | 0.20 |
| Anxiety^a^ | -0.14 | -6.26 | 0.10 |
| Long-term follow-up |  |  |  |
| Fatigue^a^ | -0.03 | -0.02 | 0.99 |
| Perceived Health Status^a^ | -1.04 | -0.46 | 0.66 |
| Depression^a^ | 1.30 | 2.24 | 0.09 |
| Anxiety^a^ | 1.37 | 7.87 | 0.01 |

^a^ *k* < 10

## **P-curve Analyses**

**Figure S23**

*P-curve analyses*
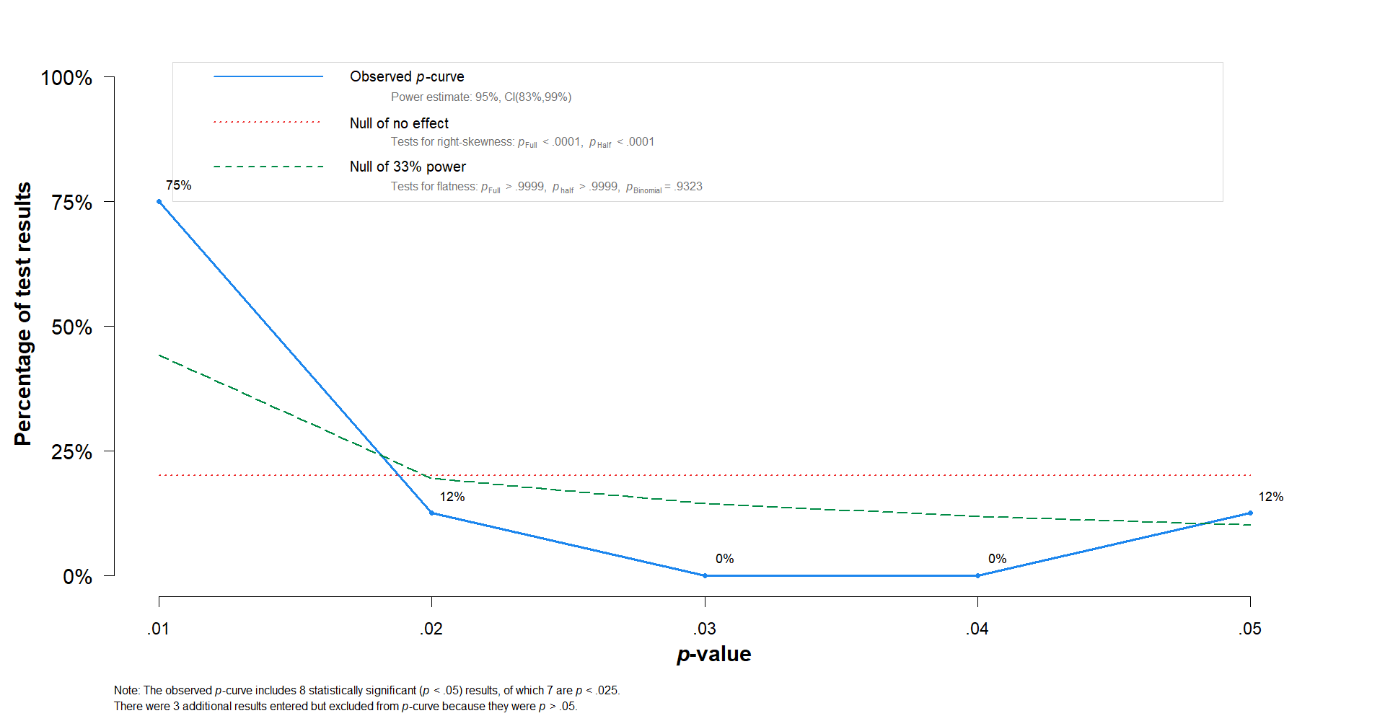


(A)


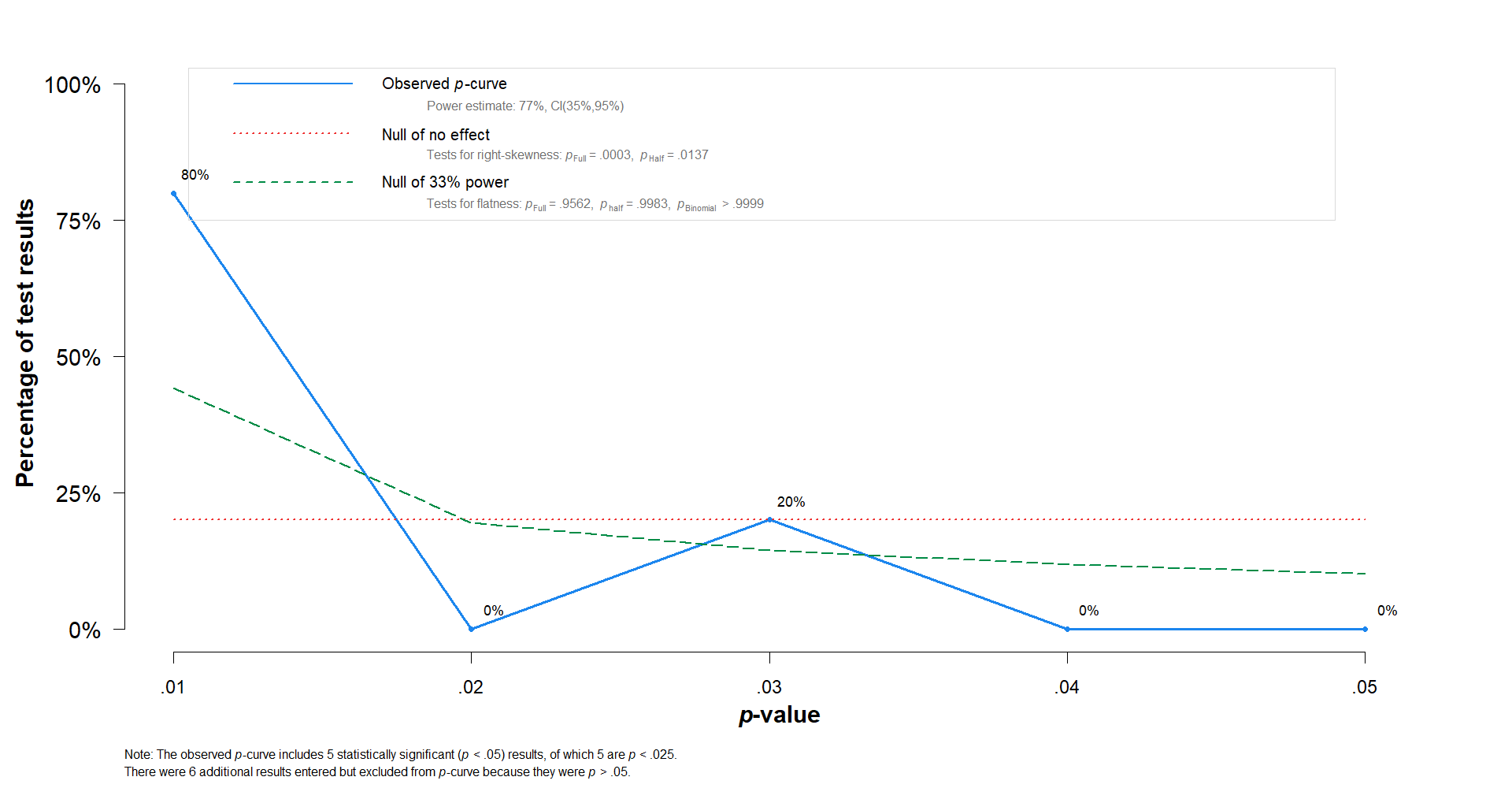


(B)


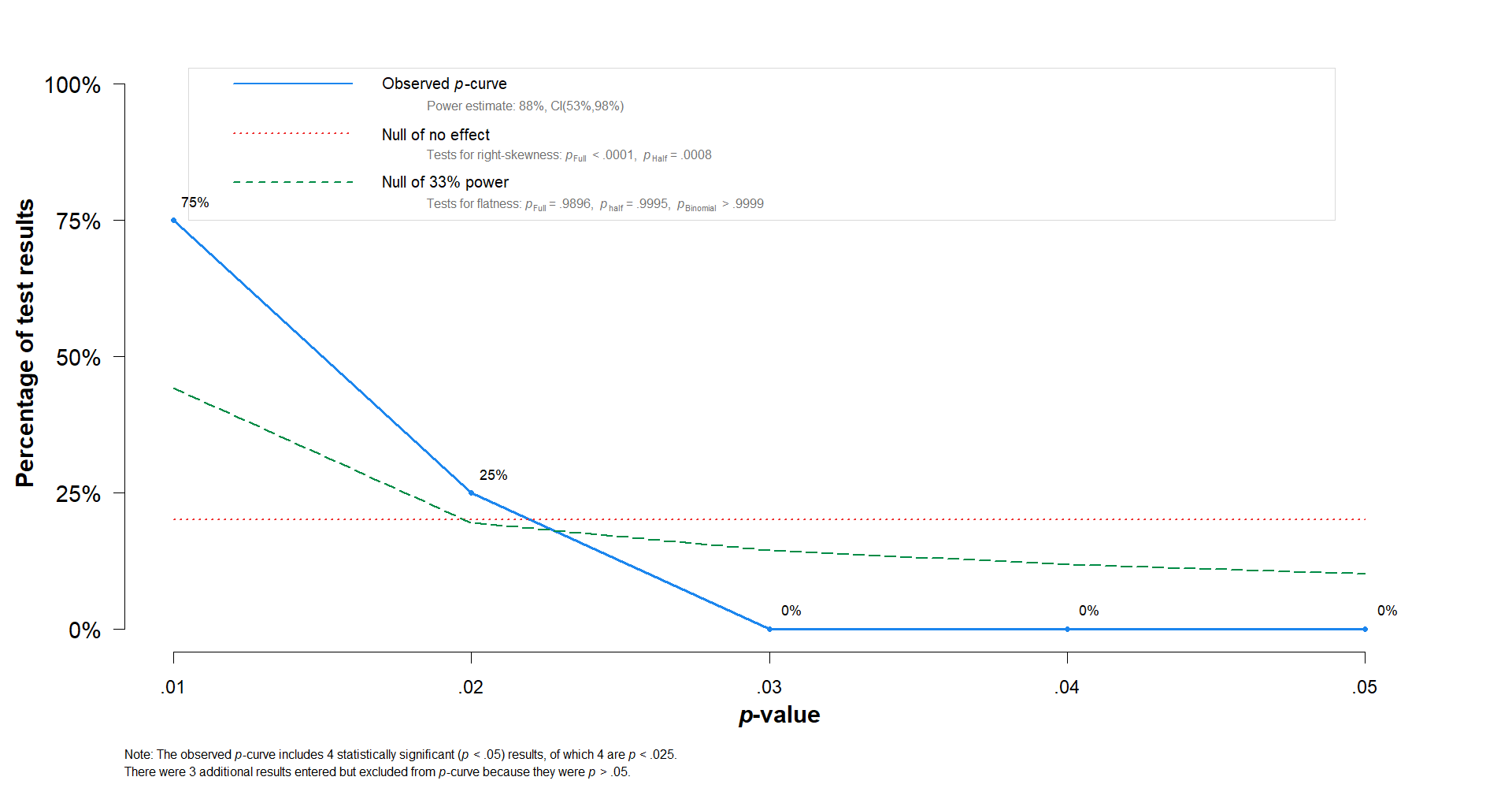


(C)

*Note.* A: *p*-curve for somatic symptom severity at post-treatment; B: *p*-curve for perceived health status at post-treatment; C: *p*-curve for somatic symptom severity at long-term follow-up. Note: *p*-curve could not be conducted for depression and anxiety at post-treatment and long-term follow-up as two or less significant (*p* < 0.05) effect sizes were detected; *p*-curve could not be conducted for perceived health status at long-term follow-up due to missing values.

# **References**

1. Holmes GP, Kaplan JE, Gantz NM, et al. Chronic fatigue syndrome: a working case definition. *Ann Intern Med*. 1988;108:387–9. doi:10.7326/0003-4819-108-3-387.
2. Sharpe MC, Archard LC, Banatvala JE, et al. A report - chronic fatigue syndrome: guidelines for research. *J R Soc Med*. 1991;84:118–21.
3. Fukuda K, Straus SE, Hickie I, Sharpe M, Dobbins JG, Komaroff A. The chronic fatigue syndrome: a comprehensive approach to its definition and study. International Chronic Fatigue Syndrome Study Group. *Ann Intern Med*. 1994;121:953–9. doi:10.7326/0003-4819-121-12-199412150-00009.
4. Carruthers BM, Jain AK, Meirleir KL de, et al. Myalgic Encephalomyelitis/Chronic Fatigue Syndrome: Clinical Working Case Definition, Diagnostic and Treatment Protocols*. J. Chronic Fatigue Syndr*. 2003;11:7–115. doi:10.1300/J092v11n01_02.
5. Carruthers BM, van de Sande MI, Meirleir KL de, et al. Myalgic encephalomyelitis: International Consensus Criteria. *J Intern Med*. 2011;270:327–38. doi:10.1111/j.1365-2796.2011.02428.x.
6. Turnbull N, Shaw EJ, Baker R, et al. (2007*). Chronic fatigue syndrome/myalgic encephalomyelitis (or encephalopathy): diagnosis and management of chronic fatigue syndrome/myalgic encephalomyelitis (or encephalopathy) in adults and children*. London: Royal College of General Practitioners; 2007.
7. World Health Organization. *The ICD-10 classification of mental and behavioural disorders: Clinical descriptions and diagnostic guidelines*. Geneva: World Health Organization; 2009.
8. Institute of Medicine (US). *Beyond myalgic encephalomyelitis/chronic fatigue syndrome: Redefining an illness.* Washington, District of Columbia: The National Academies Press; 2015.
9. Clayton EW. Beyond myalgic encephalomyelitis/chronic fatigue syndrome: an IOM report on redefining an illness. *JAMA*. 2015;313:1101–2. doi:10.1001/jama.2015.1346.
10. Schwarzer G, Carpenter J, Rücker G. *Meta-Analysis with R.* New York: Springer; 2015.
11. Viechtbauer W. Conducting Meta-Analyses in R with the metafor Package. *J Stat Softw.* 2010. doi:10.18637/jss.v036.i03.
12. Harrer M, Cuijpers P, Furukawa T, Ebert DD. *dmetar: Companion R Package For The Guide 'Doing Meta-Analysis in R*. R package version 0.0.9000. München: Protect Lab; 2019.
13. Wickham H, Averick M, Bryan J, et al. Welcome to the Tidyverse. *JOSS.* 2019;4:1686. doi:10.21105/joss.01686.
14. R Core Team. R: *A language and environment for statistical computing*. Vienna, Austria: R Foundation for Statistical Computing; 2022.
15. Lüdecke D. *Effect Size Computation for Meta Analysis*. R package esc version 0.5.1. Vienna: Comprehensive R Archive Network (CRAN); 2019.
16. Fox J, Weisberg S. *An R Companion to Applied Regression*. Thousand Oaks, CA, USA: Sage; 2019.
17. Wickham H, Bryan J, Posit PBC, et al. *readxl: Read Excel Files*. Version 1.4.0. Vienna: Comprehensive R Archive Network (CRAN); 2022
18. McGuinness LA, Higgins JPT. Risk-of-bias VISualization (robvis): An R package and Shiny web app for visualizing risk-of-bias assessments. *Res Synth Methods*. 2021; 12(1):55–61.
